# Expression signature of ten small nuclear RNAs serves as novel biomarker for prognosis prediction of acute myeloid leukemia

**DOI:** 10.1038/s41598-023-45626-x

**Published:** 2023-10-28

**Authors:** Zhongming Zhang, Rui Huang, Yongrong Lai

**Affiliations:** Department of Hematology, The First Affiliated Hospital of Guangxi Medical University, Shuang Yong Road 6, Nanning, 530021 Guangxi People’s Republic of China

**Keywords:** Haematological cancer, Genetics

## Abstract

This study aimed to screen for small nuclear RNAs (snRNAs) associated with the prognosis of acute myeloid leukemia (AML) by using The Cancer Genome Atlas (TCGA) whole-transcriptome sequencing dataset. A total of 130 AML patients from TCGA cohort with complete prognostic information and transcriptome data were enrolled in the current study. Comprehensive survival and functional enrichment analyses were performed to explore the prognostic value and potential biological functions of prognostic snRNAs in AML patients. In the current study, we screened 72 snRNAs that were notably associated with the clinical outcome of AML and developed an expression signature consist of ten snRNAs, that can be accurately applied to assess the overall survival of AML patients. Functional mechanism analysis revealed that this expression signature may be strongly linked to some classical tumor-associated pathways, such as Notch and Wnt pathways, as well as being closely related to B and T cell receptor pathways. Furthermore, we screened six compounds (chicago sky blue 6 B, 5230742, clorsulon, nefopam, nicardipine, and streptomycin) that may serve as targeted therapeutic drugs for AML using connectivity maps. Tumor immunoassays indicated significant differences in the immune microenvironment of the bone marrow tissue between high-risk and low-risk AML patients. Immune infiltration analysis also revealed significant differences in the abundance of multiple immune cells in the bone marrow of the two groups of AML patients groups. In conclusion, our results revealed a novel prognostic expression signature of AML consisting of ten snRNAs, and we conducted a preliminary exploration of its potential biological functions and tumor immunity.

## Introduction

Acute myeloid leukemia (AML) is a hematological malignancy. Currently, there is no effective treatment for most types of AML that mainly relies on chemotherapy. However, the prognosis of many AML patients remains poor^1^. According to cell morphology and histochemical characteristics, AML can be divided into different types based on cell morphology and histochemical characteristics. With the use of immunology, cytogenetics, and other technologies, in-depth knowledge of the biological parameters of AML cells has provided the basis for the accurate classification, clinical outcome, and treatment of AML^2^. At the same time, genomics is widely used for the diagnosis, treatment selection, and prognostic evaluation of AML. Even so, accurate and efficient prognostic prediction models and biomarkers for AML that can guide clinical diagnosis and treatment are yet to be developed. Therefore, efficient and objective biomarkers are needed to accurately evaluate the diagnosis, treatment, and prognosis of AML. Small nuclear RNA (snRNAs) are intracellular RNA molecules. It is an ingredient of the RNA spliceosome in the post-transcriptional processing of eukaryotes, and plays a role in the treatment of mRNA precursors^3^. Abnormal variants of snRNAs have been widely perceived as strongly linked to malignant cancers, including hepatocellular carcinoma (HCC), chronic lymphocytic leukemia (CLL), sonic hedgehog medulloblastoma (Shh-MB), melanoma, and many other malignant tumors and blood diseases^4–6^. By analyzing the snRNA data set of TCGA, Qin et al.^7^ built a prognostic risk scoring system based on three snRNAs to predict the prognosis of patients with gastrointestinal malignancies through computational biology and bioinformatics analysis methods. Based on a similar method, Zhang et al. constructed a prognostic risk score system based on Eight m6A-associated snRNAs to predict the prognosis of HCC patients^8^. Reviewing previous studies, we have not found any reports on the value of snRNAs in AML prognosis. To address this research gap, we conducted an integrated investigation of the clinical value and molecular mechanisms of snRNAs in AML. The objective of the current study was to investigate the snRNAs associated with AML prognosis using The Cancer Genome Atlas (TCGA) whole transcriptome sequencing dataset.

## Results

### SnRNA risk score modeling and survival analysis in AML

**Figure 1:**
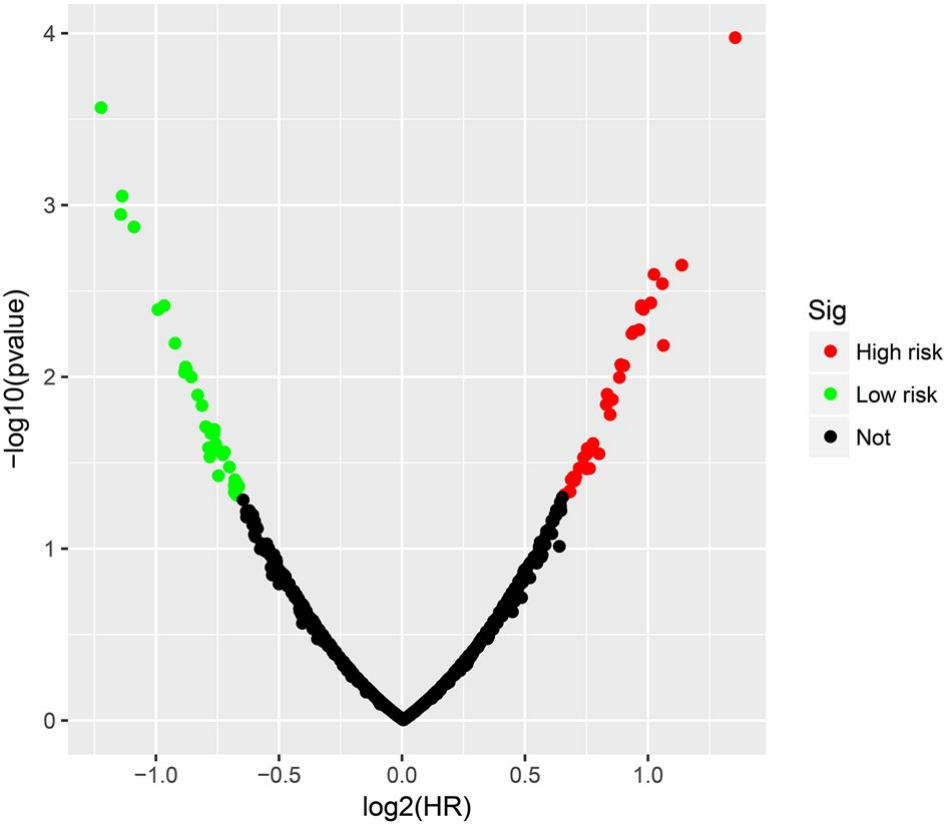
Distribution map of prognostic snRNAs in AML. *Notes* Figure was drawn by R4.0.2 version: https://www.r-project.org.

**Figure 2:**
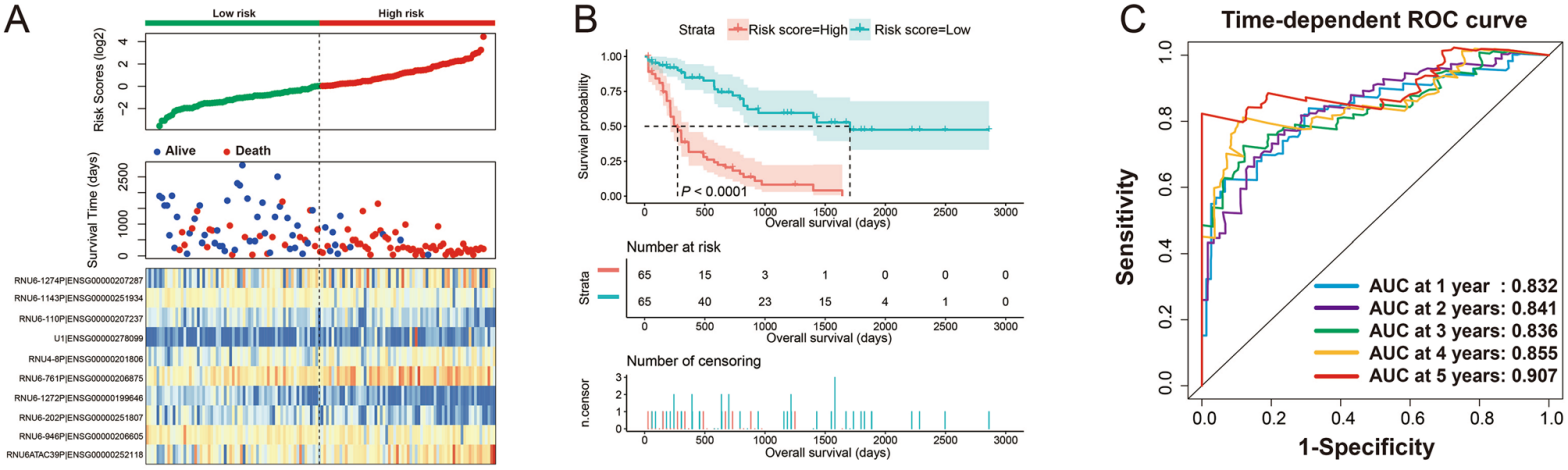
The prognostic risk prediction expression signature of AML composed of ten prognostic snRNAs. (**A**) Distribution map of expression signature and patients' survival time; (**B**) Kaplan–Meier curve of AML patients with different risk scores; (**C**) SurvivalROC curve of ten snRNAs expression signature. *Notes* All figures were drawn by R4.0.2 version: https://www.r-project.org.

**Figure 3:**
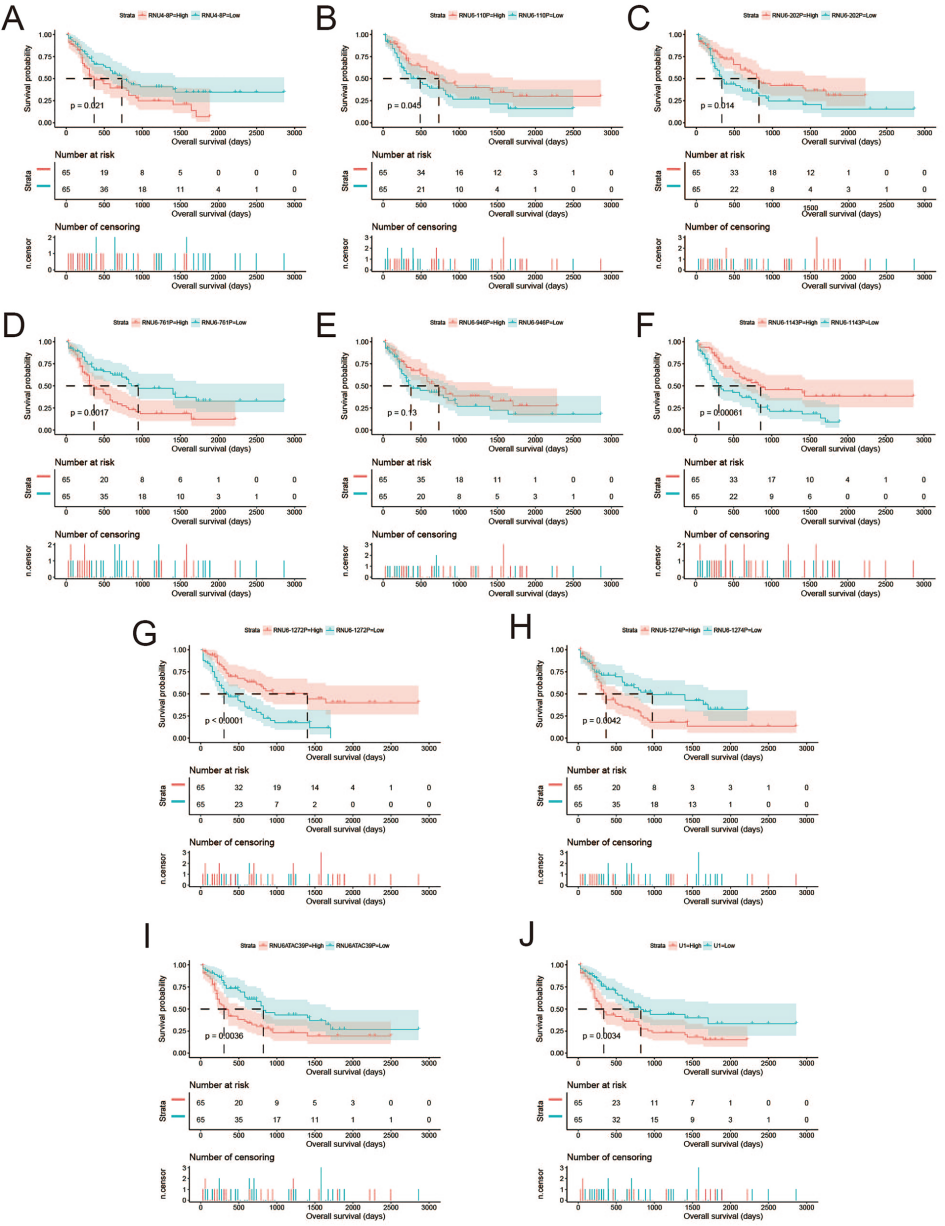
Kaplan–Meier curve of ten prognostic snRNAs in AML. (**A**) Kaplan–Meier curve of RNU4-8P; (**B**) Kaplan–Meier curve of RNU6-110P; (**C**) Kaplan–Meier curve of RNU6-202P; (**D**) Kaplan–Meier curve of RNU6-761P; (**E**) Kaplan–Meier curve of RNU6-946P; (**F**) Kaplan–Meier curve of RNU6-1143P; (**G**) Kaplan–Meier curve of RNU6-1272P; (**H**) Kaplan–Meier curve of RNU6-1274P; (**I**) Kaplan–Meier curve of RNU6ATAC39P; (**J**) Kaplan–Meier curve of U1. *Notes* All figures were drawn by R4.0.2 version: https://www.r-project.org.

**Figure 4:**
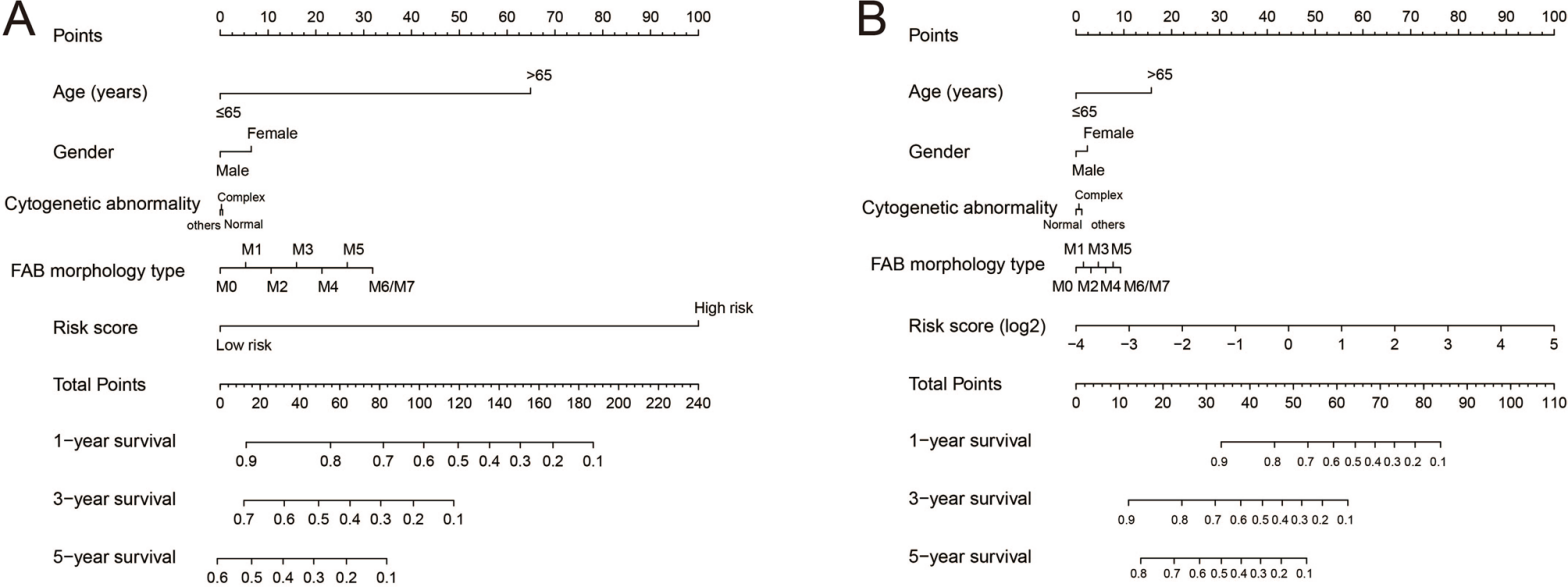
Nomogram of ten prognostic snRNAs expression signature in AML. (**A**) Risk score was divided into two groups; (**B**) Risk score is grouped according to scores. *Notes* All figures were drawn by R4.0.2 version: https://www.r-project.org.

The flow chart of this study was shown in Fig. S1. The AML patient records are shown in Table S1. French-American-British (FAB) morphology type and age were notably associated with the overall survival (OS) of patients with AML in the TCGA cohort. In the multivariate Cox proportional hazard regression model, we incorporated FAB morphology type and age into the model for adjustment. Altogether, 1872 snRNAs were obtained from the RNA sequencing data of TCGA, and snRNAs with an average value of < 1 were removed. Finally, 694 snRNAs were obtained by edge R-normalization for subsequent analyses. Using a multivariate Cox proportional hazard regression model in the R platform to screen for prognostic snRNAs, we identified 72 snRNAs that were notably associated with AML OS (Fig. 1, Table S2). We then included 72 prognostic snRNAs into the R platform for optimal prognostic model screening and finally obtained an expression signature containing 10 snRNAs. The expression signature was as follows: risk score = Exp_RNU6-1274P_ × 0.1409 + Exp_RNU6-1143P_ × (− 0.2353) + Exp_RNU6-110P_ × (− 0.2212) + Exp_U1_ × 0.1877 + Exp_RNU4-8P_ × 0.2214 + Exp_RNU6-761P_ × 0.3087 + Exp_RNU6-1272P_ × (− 0.5222) + Exp_RNU6-202P_ × (− 0.1926) + Exp_RNU6-946P_ × (− 0.2896) + Exp_RNU6ATAC39P_ × 0.3445. The high- and low-risk groups were divided according to the median value of the risk score. We observed that the OS of high-risk AML patients was notably shorter than that of low-risk patients [log-rank *P* < 0.0001, adjusted *P* < 0.0001, adjusted hazard ratio (HR) = 5.524, 95% confidence interval (CI) = 3.180–9.598, Fig. 2A,B]. Subsequently, *survivalROC* analysis showed that the risk score had high accuracy in predicting AML OS, and the area under the ROC curve (AUC) for predicting the 5-years OS of AML was 0.907 (Fig. 2C). Multivariate survival analysis results for these ten prognostic snRNAs are presented in Table 1 and Fig. 3A–J. We also used A nomogram model was used to predict the individual prognosis of this snRNA signature. Our study observed that this snRNA signature contributed the most to the death of AML patients in TCGA cohort (Fig. 4A,B).

**Table 1:** Multivariate survival analysis results of the ten snRNAs included in the risk score model in AML.

ID	Ensemble ID	Adjusted P§	HR	Low 95%CI	High 95%CI
RNU6-1143P	ENSG00000251934	0.000272814	0.426713992	0.269758037	0.674993163
RNU6-1272P	ENSG00000199646	0.001142804	0.451039966	0.279165911	0.728731709
RNU6-202P	ENSG00000251807	0.001349364	0.468147755	0.294325617	0.744625366
RNU6-1274P	ENSG00000207287	0.002548141	2.026689935	1.280923906	3.206648007
U1	ENSG00000278099	0.003877337	1.955643381	1.240514241	3.083028722
RNU6ATAC39P	ENSG00000252118	0.005643496	1.903128765	1.206614834	3.001702776
RNU6-761P	ENSG00000206875	0.008652609	1.861213512	1.170547774	2.959397141
RNU4-8P	ENSG00000201806	0.008699737	1.848034	1.168063504	2.923839031
RNU6-110P	ENSG00000207237	0.00908825	0.542894557	0.343086512	0.859067581
RNU6-946P	ENSG00000206605	0.009464659	0.539917887	0.338955868	0.860027371

*AML* acute myeloid leukemia, *snRNA* small nuclear RNA, *FAB* French-American-British, *HR* hazard ratio, *CI* confidence interval.

^§^Adjusted for age and FAB morphology type in multivariate Cox proportional hazards regression model.

### Molecular mechanism exploration of risk score in AML

**Figure 5:**
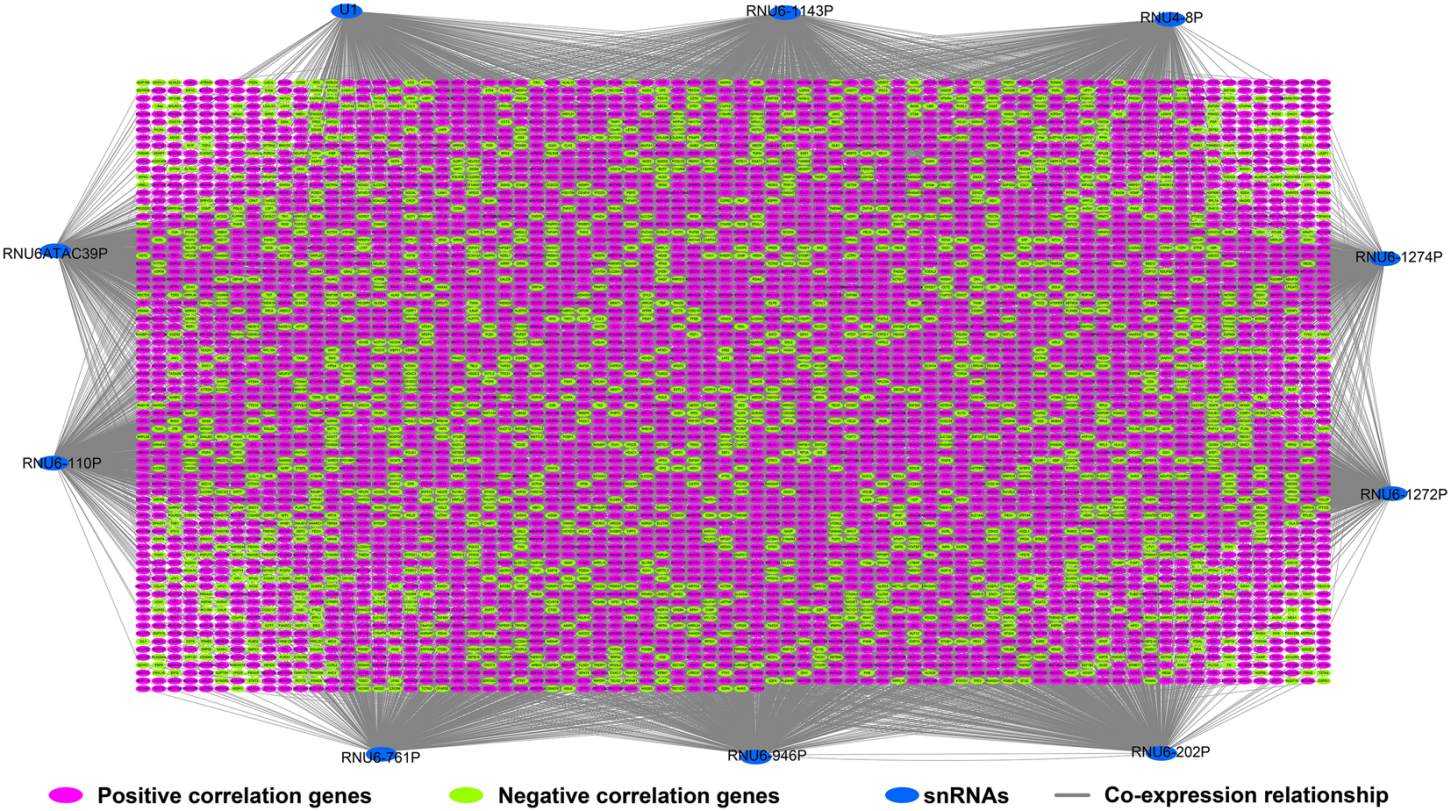
Interaction network plot of ten prognostic snRNAs and their co-expressed genes. *Notes* Figure was drawn by Cytoscape 3.6.1 version: https://cytoscape.org.

**Figure 6:**
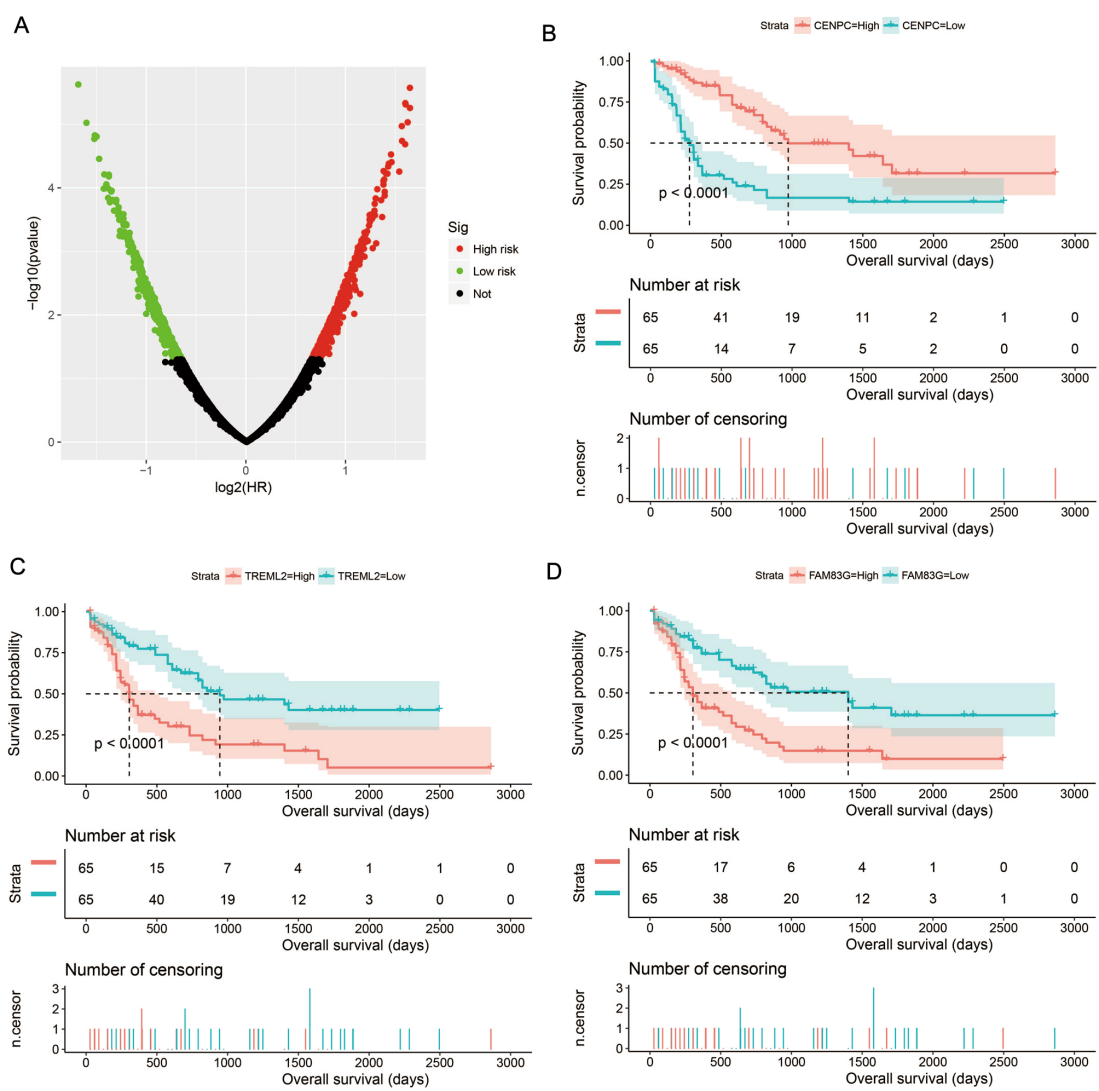
Survival analysis results of co-expressed genes of ten prognostic snRNAs. (**A**) Survival analysis results distribution map of snRNA co-expressed genes in AML; (**B**) Kaplan–Meier curve of centromere protein C (CENPC); (**C**) Kaplan–Meier curve of triggering receptor expressed on myeloid cells like 2 (TREML2); (**D**) Kaplan–Meier curve of family with sequence similarity 83 member G (FAM83G). *Notes* All figures were drawn by R4.0.2 version: https://www.r-project.org.

It is well known that snRNA is mainly involved in the post-transcriptional regulation of PCG mRNA, and functions mainly by regulating protein-coding gene (PCG) mRNA expression. Therefore, mRNA regulated by a specific snRNA exhibits a co-expression interaction with this snRNA at the expression level. A total of 8106 pairs of interactions between these ten snRNAs and PCGs (Fig. 5, Table S3) were identified by co-expression analysis. We carried out gene ontology (GO) and Kyoto Encyclopedia of Genes and Genomes (KEGG) functional enrichment of PCGs and found that these PCGs were significantly enriched in DNA repair, NIK(NF-kappaB-Inducing Kinase)/NF(nuclear factor)-kappa B signaling, planar cell polarity pathway, regulation of signal transduction by p53 class mediator, Wnt signaling pathway, T cell receptor (TCR) signaling pathway, cell division, cell–cell adherens junction, regulation of mitotic cell cycle phase transition, and the Fanconi anemia pathway (Table S4). The results of the BiNGO enrichment analysis also verified these results (Fig. S2). We also performed a survival analysis for these PCGs (Table S5) and screened 1017 PCGs that were associated with AML OS (Fig. 6A, Table S5). The top three significant PCGs were centromere protein C (CENPC, adjusted *P* < 0.0001, adjusted HR = 0.310, 95% CI 0.191–0.504, Fig. 6B), triggering receptor expressed on myeloid cells such as 2 (TREML2, adjusted *P* < 0.0001, adjusted HR = 3.114, 95% CI 1.938–5.004, Fig. 6C) and a family with sequence similarity 83 member G (FAM83G, adjusted *P* < 0.0001, adjusted HR = 3.015, 95% CI 1.880–4.837, Fig. 6D).

**Figure 7:**
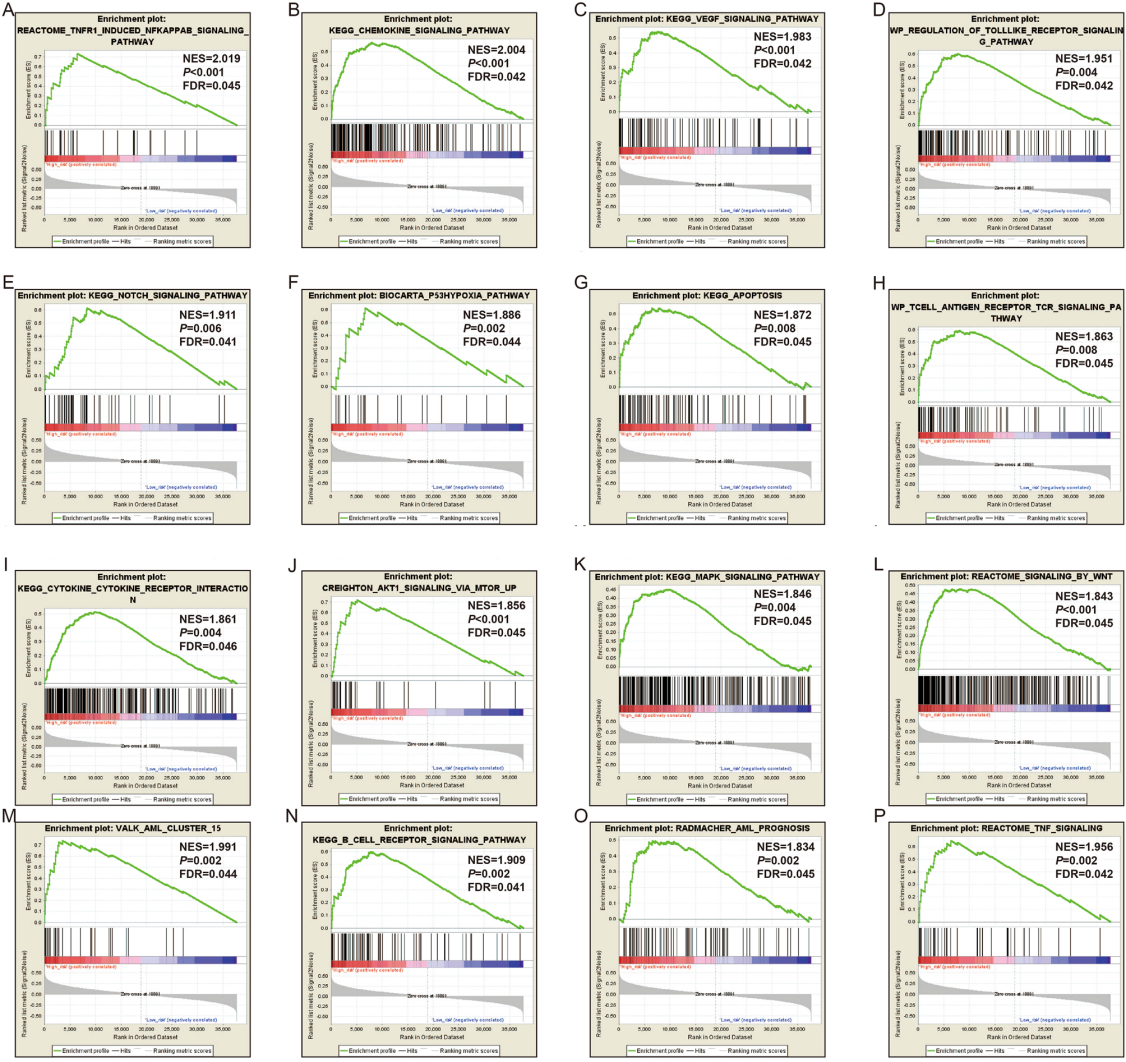
GSEA analysis results of the low- and high-risk score phenotypes in AML using c2 reference gene set (**A**–**P**). *Notes* All figures were drawn by GSEA 2.2.3 version: https://www.gsea-msigdb.org/gsea/index.jsp.

**Figure 8:**
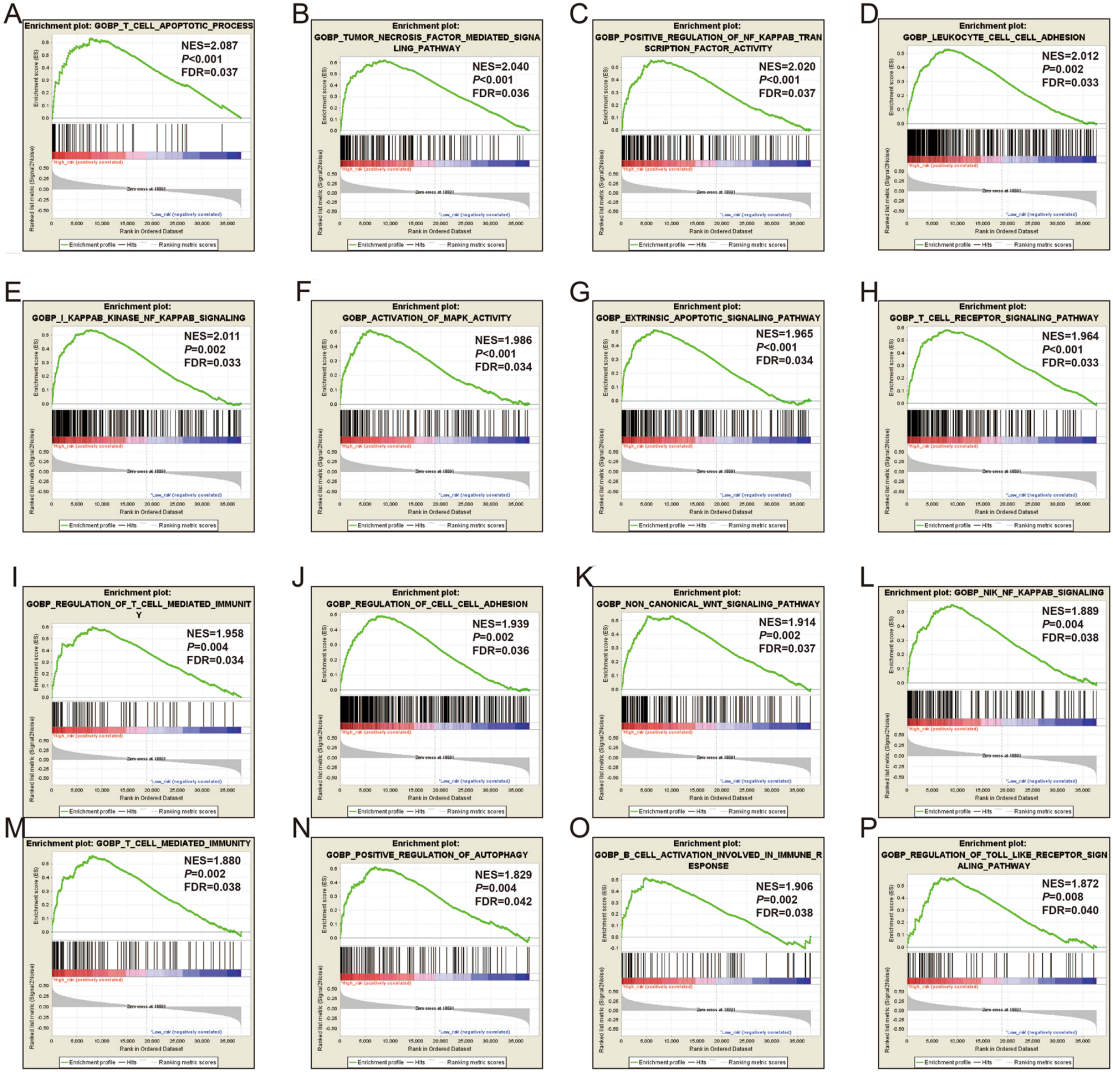
GSEA analysis results of the low- and high-risk score phenotypes in AML using c5 reference gene set (**A**–**P**). *Notes* All figures were drawn by GSEA 2.2.3 version: https://www.gsea-msigdb.org/gsea/index.jsp.

In gene set enrichment analysis (GSEA), our results verified the above-mentioned Database for Annotation, Visualization, and Integrated Discovery v6.8 (DAVID v6.8) enrichment results for these PCGs. In GSEA analysis using the c2 gene set, we found that high-risk AML patients were notably enriched in the following signaling pathways: NF/kappa B, VEGF, Toll-like receptor (TLR) pathway, Notch, tumor protein 53 (TP53), apoptosis, TCR, Akt1, mitogen-activated protein kinase (MAPK), Wnt, tumor necrosis factor (TNF), AML cluster 15, B-cell receptor (BCR) signaling pathway, and cytokine-cytokine pathway (Fig. 7A–P, Table S6). By Using the c5 gene set, we found that AML patients with high-risk phenotypes were notably enriched in the T cell apoptotic process, TNF pathway, NF/kappa B, cell–cell adhesion, MAPK pathway, TCR, T cell-mediated immunity, B cell activation involved in the immune response, and TLR signaling pathway (Fig. 8A–P, Table S7).

**Figure 9:**
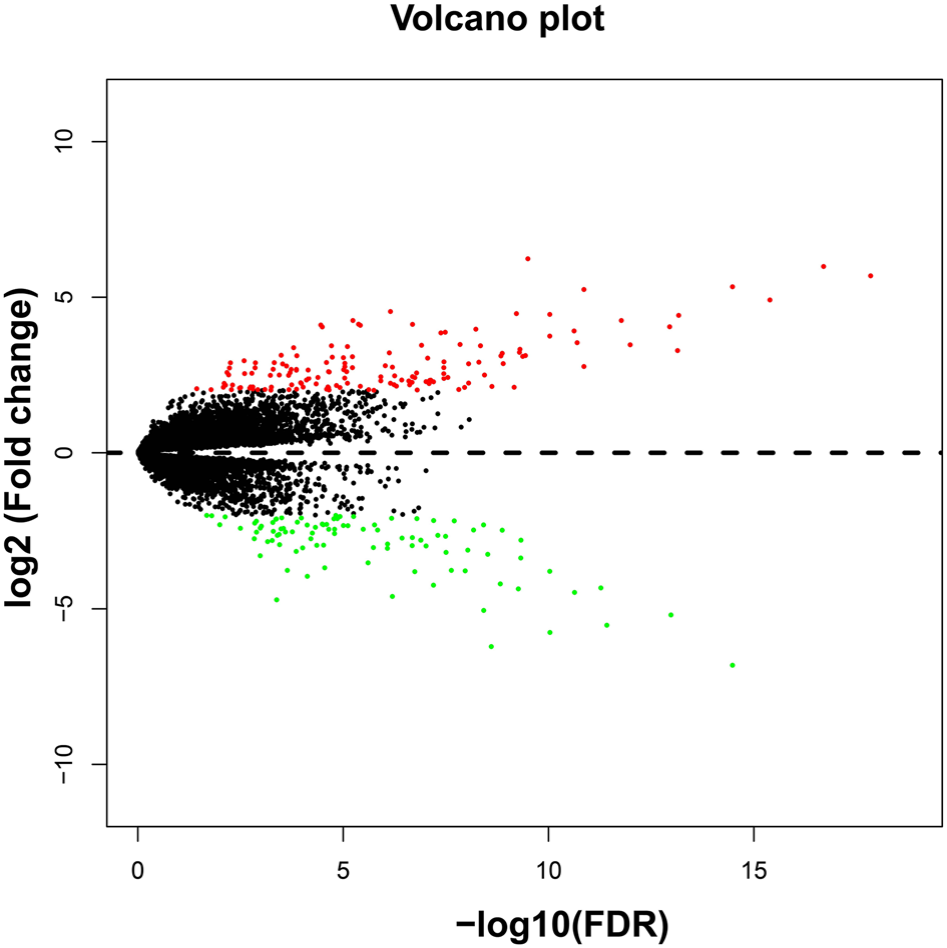
Volcano map of differentially expressed genes between low- and high-risk score phenotypes. *Notes* Figure was drawn by R4.0.2 version: https://www.r-project.org.

**Figure 10:**
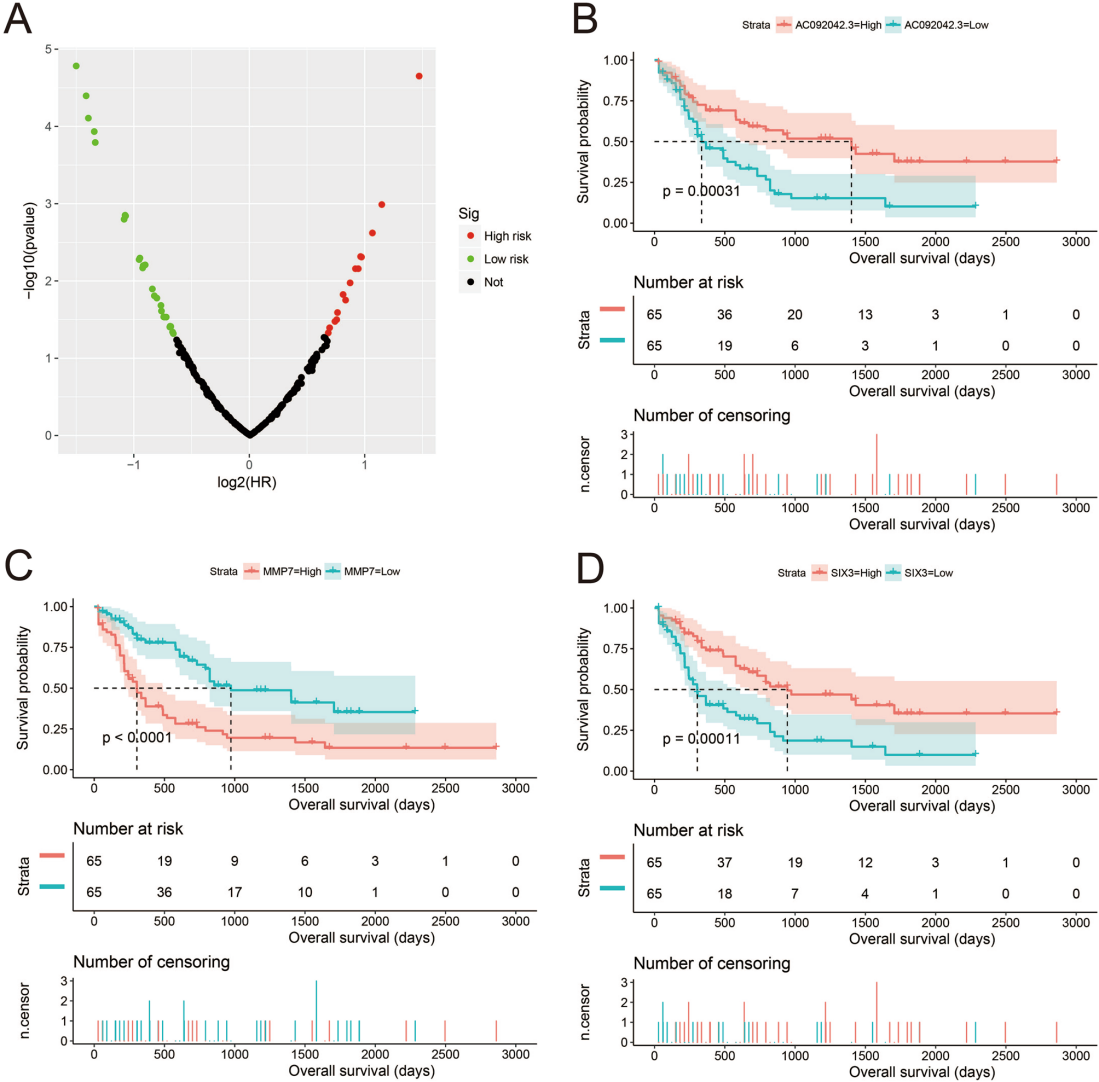
Survival analysis results of differentially expressed genes (DEGs) between low- and high-risk score phenotypes. (**A**) Survival analysis results distribution map of DEGs; (**B**) Kaplan–Meier curve of ACO92042.3; (**C**) Kaplan–Meier curve of matrix metallopeptidase 7 (MMP7); (**D**) Kaplan–Meier curve of SIX homeobox 3 (SIX3). *Notes* All figures were drawn by R4.0.2 version: https://www.r-project.org.

**Figure 11:**
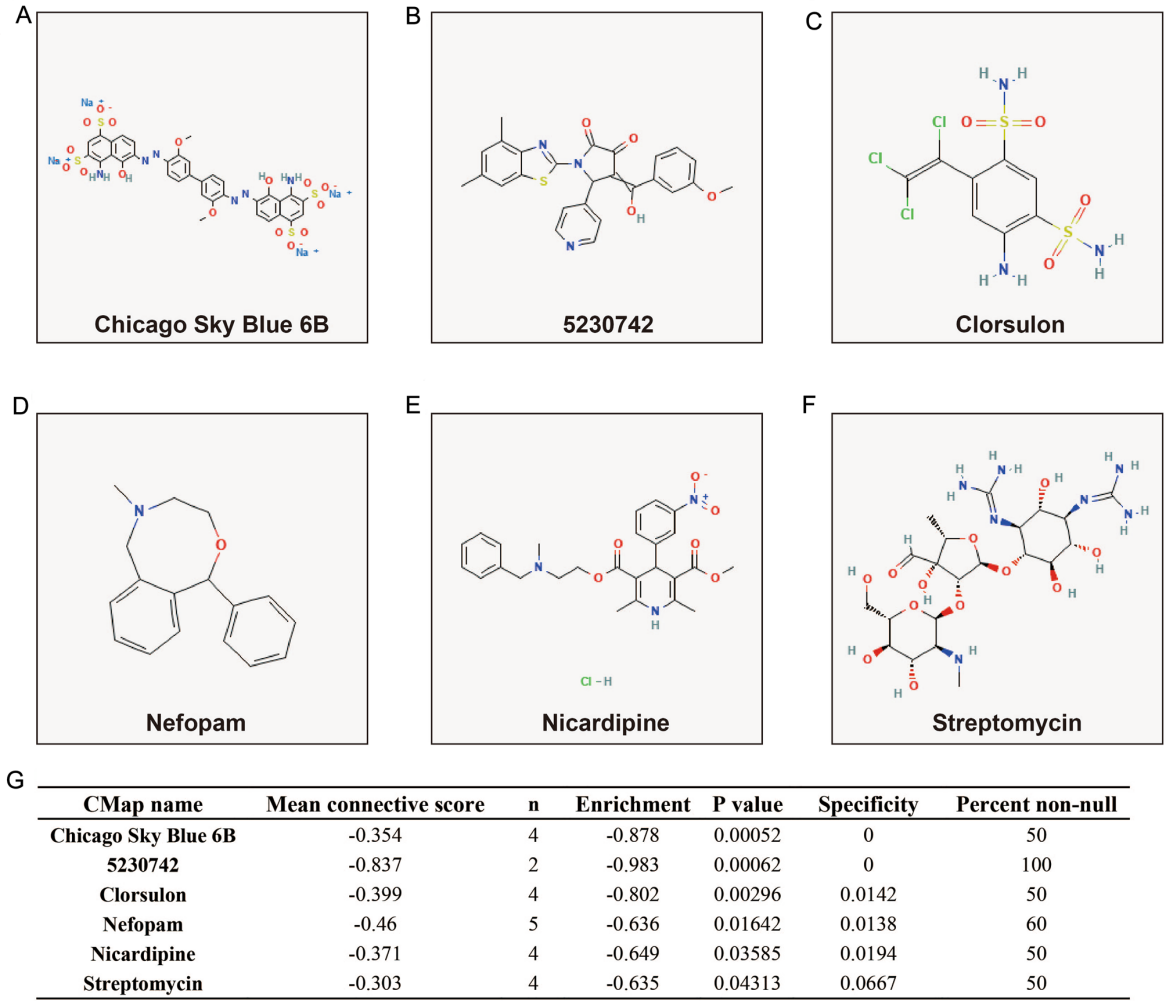
CMap results of DEGs between low- and high-risk score phenotypes. (**A**) Chemical formula of chicago sky blue 6B; (**B**) Chemical formula of 5230742; (**C**) Chemical formula of clorsulon; (**D**) Chemical formula of nefopam; (E) Chemical formula of nicardipine; (**F**) Chemical formula of streptomycin; (**G**) CMap results list.

We also explored functional enrichment by comparing the differentially expressed genes (DEGs) between patients with high- and low-risk AML. Through *edgeR* screening, 244 DEGs between these two groups were obtained, of which 96 were downregulated and 148 were upregulated (Fig. 9, Fig. S3, Table S7). The enrichment analysis of GO and KEGG revealed that these DEGs may be involved in the following biological molecular mechanisms: positive regulation of leukocyte chemotaxis, cell junctions, transcriptional activator activity, RNA polymerase II core promoter proximal region sequence-specific binding, positive regulation of transcription from the RNA polymerase II promoter, ephrin receptor signaling pathway, positive regulation of cell proliferation, cell–cell signaling, focal adhesion, ECM-receptor interaction, PI3K-Akt signaling pathway, and Notch signaling pathway (Table S9). BiNGO enrichment analysis also verified these results (Fig. S4). Prognostic analysis revealed that 42 DEGs were significantly associated with the OS of AML patients (Fig. 10A, Table S10), and the top three significant DEGs were AC092042.3 (adjusted *P* < 0.0001, adjusted HR = 0.352, 95% CI 0.219–0.566, Fig. 10B), matrix metallopeptidase 7 (MMP7, adjusted *P* < 0.0001, adjusted HR = 2.761, 95% CI 1.726–4.415, Fig. 10C), and SIX homeobox 3 (SIX3, adjusted P < 0.0001, adjusted HR = 0.374, 95% CI 0.234–0.598, Fig. 10D). These DEGs were imported into the online analysis tool connectivity map (CMap) to screen for potential small-molecule targeted therapies. We obtained six compounds (chicago sky blue 6 B, 5230742, clorsulon, nefopam, nicardipine, and streptomycin) that may be targeted therapeutic drugs for this risk score signature; their chemical structures are shown in Fig. 11A–F. The detailed list of CMap results is shown in Fig. 11G.

### Immune microenvironment and immune infiltration analysis

**Figure 12:**
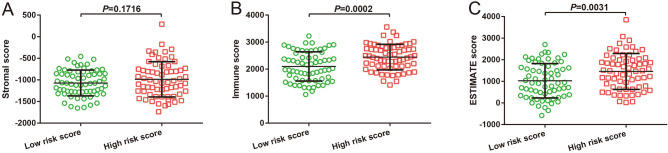
Comparison of immune microenvironment score between AML patients with different risk score phenotypes. (**A**) Stromal score between low- and high-risk score phenotypes; (**B**) Immune score between low- and high-risk score phenotypes; (**C**) ESTIMATE score between low- and high-risk score phenotypes. *Notes* All figures were drawn by GraphPad 6.01 version: https://www.graphpad.com.

**Figure 13:**
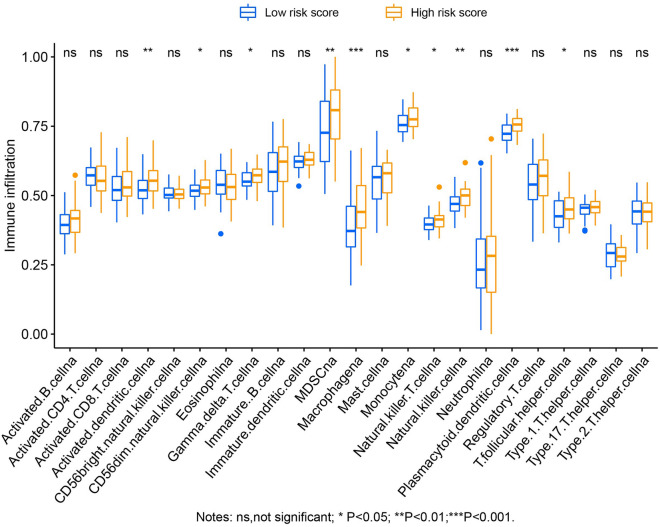
The abundance of immune infiltration between AML patients with different risk score phenotypes was compared by ssGSEA method. *Notes* All figures were drawn by R4.0.2 version: https://www.r-project.org.

In the functional enrichment analysis of this risk score signature, we found that the risk score model was markedly related to immune-associated pathway mechanisms such as the TCR and BCR Signaling pathways. To this end, we investigated this signature from two aspects: the immune microenvironment and immune infiltration. Immunohistochemical analysis revealed no significant differences in the stromal scores between the two groups (Fig. 12A, *P* = 0.1716). Nevertheless, significant differences were observed in immune (Fig. 12B, *P* = 0.0002) and ESTIMATE scores (Fig. 12C, *P* = 0.0031). Single-sample gene set enrichment analysis (ssGSEA) revealed significant differences in the abundance of ten immune cells in immune infiltration between low- and high-risk AML. The abundance of immune infiltrates was higher in high-risk AML patients than that in low-risk patients (Fig. 13).

## Discussion

The advent of high-throughput sequencing technology has greatly expanded our understanding of snRNA. Dysregulation of snRNA is closely associated with cancer progression, and there is growing evidence of snRNA's potential as a cancer diagnostic and prognostic biomarker, or therapeutic target^9^. By reviewing the literature, we have not found any reports on the comprehensive exploration of snRNAs in tumors, including hematological malignancies. A review of published multi-omics analyses based on TCGA dataset showed that snRNAs are rarely reported. We searched for 10 snRNA-related reports included in the prognostic signature. We only found that U1 has been reported in cancer, and the remaining nine snRNAs are new prognostic markers for AML that have not been reported before^10^. Shuai et al.^5^ showed that mutations in U1 were significantly associated with poor outcomes in patients with CLL, and they also suggested that mutations in U1 are strongly associated with alcohol consumption in HCC patients. Zhang et al.^11^ extracted U1 related snRNA from lung cancer patients for the first time and proposed that this snRNA might be a marker of lung cancer. A subsequent study by Dong et al.^12^ confirmed that U1 serves as a diagnostic marker for lung cancer. After silencing or overexpressing U1 in HeLa and other malignant tumor cell lines, Oh et al. found that changing the level of U1 can notably affect the migration and invasion capabilities of malignant tumor cells^13^. Suzuki et al.^6^ revealed that mutations in U1 in SHH medulloblastoma can notably suppress the tumor suppressor gene patched 1 (PTCH1) and activate oncogenes GLI family zinc finger 2 (GLI2) and cyclin D2 (CCND2), suggesting that U1 may serve as a therapeutic target for this tumor. Cheng et al. upregulated U1 in PC-12 pheochromocytoma cell lines and sequenced overexpressed U1 in cancer cell lines using whole-genome sequencing. Subsequently, differentially expressed genes were screened using bioinformatics analysis to determine the activation of oncogene-related functions and pathways^14^. By reviewing the abovementioned literature, we found that U1 was defined as an oncogene in previous reports. In the current study, we discovered that AML patients with high U1 expression had poorer clinical outcomes than those with low U1 expression, which is consistent with the results of previous studies.

Theoretical modeling of gene/protein signaling networks plays an important role in understanding regulatory mechanisms and discovering potential therapeutic targets for diseases. Li et al.^15^ verified the regulatory mechanism of caspase-1 or GSDMD and apoptosis and pyrodeath by using mathematical modeling combined with quantitative western blot analysis. Their team also used a phase-field model to explore how protein phase separation are controlled by mRNAs, and these computational models were eventually confirmed experimentally^16^. Therefore, the exploration of biological mechanisms through the use of computer simulation models can help to further understand functional mechanisms and discover potential therapeutic targets for diseases. These computational biology deep learning predictive models include, but are not limited to, fivefold cross-validation experiments. Graph convolutional network with graph attention network and so on^17,18^. It is still difficult to identify the association between genetic markers and noncoding RNAs in large-scale experiments, and computational biological methods to predict the association between genes and noncoding RNAs remain the potential effective way to explore disease markers and molecular mechanisms. However, the current prediction methods still have some limitations and need to be verified by experiments. In the field of computational biology, Zhao et al. have also published a variety of programs and research strategies for predicting the association of genetic markers and noncoding RNAs as well as the correlation of single cell sequencing data in computational biology^19–23^. In this study, the computational biology analysis method adopted by us is similar to theirs. Our current study also identified signaling pathways closely related to AML through bioinformatics enrichment analysis, including the TCR, BCR, Wnt, and Notch pathways. The T-cell receptor signaling pathway plays an important role in cancer immunotherapy^24,25^, and can also be used for the immunotherapy of hematological malignancies^26^. TCR can be used as PD-1 blockade therapy, and the effect of PD-1 blockade therapy in AML patients can be determined by the different states of T cells^27^. T-cell receptor gene therapy can significantly prevent AML recurrence after bone marrow transplantation^28^. B-cell receptors are widely used in lymphatic malignancies, and the targeted BCR signaling pathway can be used in the treatment of CNL^29–31^.

The Wnt signaling pathway has been shown to be closely related to cancer. Studies have suggested that the deregulation of Wnt signaling is beneficial for malignant transformation, tumor progression, and resistance to conventional cancer therapy^32–34^. There is growing evidence that dysregulated Wnt signaling may also disrupt cancer immune surveillance, thus promoting immune evasion and resistance to multiple immunotherapies, including immune checkpoint blockers^35,36^. The Wnt signaling pathway is involved in regulating the immune microenvironment in leukemia^37^. Wnt signaling may be disrupted in acute leukemia in several ways, including changes in gene expression and protein levels, epigenetic regulation, and mutations. Dysregulation of these Wnt signaling pathways may have different effects on the diagnosis and progression of AML, and is also a potential target for AML treatment^38^. Our current study found that the risk score may play a role in AML by regulating the Wnt signaling pathway, and we also observed that this risk score was significantly related to the immune microenvironment and immune cell infiltration. Through a literature search, we found that the Wnt signaling pathway plays an indispensable role in AML. Dai et al. explored the prognostic value of the Wnt gene family using TCGA AML cohort and found that Wnt family genes are closely related to the clinical outcome of AML^39^. Multiple genes in the Wnt signaling pathway have also been identified to be associated with AML^40^.

The present study also identified another classic cancer-related signaling pathway, the Notch signaling pathway, which is notably associated with risk score and tumor immunity. The Notch signaling pathway plays an indispensable role in the occurrence, progression, prognosis, and treatment of hematological malignancies and solid cancers, and serves as a target for malignant tumors treatment^41–44^. Although reports have found that Notch plays an oncogenic role in most solid tumors, it is still controversial whether Notch is carcinogenic or tumor suppressor in AML. Kannan et al. observed that activated Notch1 and Notch2 could inhibit the growth of AML cells in vivo and otherwise promote their growth of AML cells^45^. Lobry et al. observed similar results in their study^46^, whereas mutations in Notch1 were often associated with poor prognosis in AML^47^. By reviewing the literature, we have summarized the potential molecular mechanisms explored by expression signatures through functional enrichment. These signaling pathways are not only classic malignant tumor-related pathways but are also closely related to tumor immunity. Our current study also developed the tumor immunity expression signature, and we observed that this expression signature is notably associated with tumor immunity.

Among the screened drugs, Chicago Sky Blue 6 B was not found to be associated with AML or cancer in our review of previous studies. It is an allosteric inhibitor of macrophage migration (MIF), which inhibits osteoclast formation and promotes osteogenesis by inhibiting the NF-Kappa B signaling pathway^48,49^. In previous studies, nicardipine was found to enhance sensitivity to tumor chemotherapy agents. Shi et al. revealed that nicardipine could enhance the antitumor effect of temozolomide in glioblastoma multiforme (GBM) by inhibiting stem cell autophagy and promoting apoptosis^50^. Nicardipine has also been reported to significantly enhance the inhibitory effects of this drug on breast cancer cell proliferation^51^. Chen et al. used nicardipine to interfere with breast cancer cell lines and found that nicardipine significantly inhibited the migration and colony-forming abilities of breast cancer cells. By exploring the molecular mechanism, it was found that nicardipine achieves this phenomenon by regulating the Nrf2/ Ho-1 Axis and matrix metalloproteinase-9 in breast cancer cells^52^. Nicardipine significantly inhibited the growth of prostate cancer xenografts in nude mice in vivo^53^. A review of the literature revealed that the remaining four drugs were not associated with cancer or AML. Our findings are the first to reveal that these drugs may be targeted therapies for AML. However, these results need to be confirmed in future studies.

Although we found many interesting results in this study, our results still have certain limitations, because we were a small-sample study with a single cohort. First, it was a single-cohort study and lacked an additional validation cohort. Second, our study mainly used bioinformatics analysis tools to explore functional mechanisms and still needs in vivo and in vitro experimental verification. However, our findings provide a theoretical basis for exploring the potential clinical applications of snRNAs in AML and lay a foundation for the subsequent development of snRNA-related AML biomarkers.

## Conclusions

Our current study preliminarily identified 72 snRNAs that are associated with AML prognosis. Our results revealed a novel prognostic expression signature of AML consisting of ten prognostic snRNAs, and we conducted a preliminary exploration of its potential biological functions and tumor immunity. Functional enrichment analysis revealed that this prognostic signature is notably associated with biological functions and pathways such as cell adhesion, BCR, TCR, Wnt, and Notch signaling pathways. We also used CMap to screen six potential targeted drugs for this risk score of AML: Chicago sky blue 6 B, 5230742, clorsulon, nefopam, nicardipine, and streptomycin. Immunological analysis revealed significant differences in the immune microenvironments of AML patients with high- and low-risk phenotypes. The immune infiltration abundance of ten immune cells in the high-risk score phenotype was significantly higher than that in the low-risk score phenotype. However, because our study was a single-center, small-cohort study, these results require additional verification in future studies.

## Materials and methods

### Data preparation

Original RNA sequencing (RNA-Seq) count dataset and demographic information were obtained from TCGA website (https://portal.gdc.cancer.gov)^54^. The normalization of the raw sequencing data was performed using edgeR on the R platform^55^. We excluded patients with no prognostic or RNA sequencing data, and 130 patients will be included in the follow-up study. The patient inclusion and exclusion criteria can be found in our previous study^56^. The data used in this study met the requirements of TCGA. This study was approved by the Ethics Committee of the First Affiliated Hospital of the Guangxi Medical University. The approval number is 2021(KY-E-325).

### SnRNA risk score modeling and survival analysis in AML

A multivariate Cox proportional hazard regression model was used to screen for snRNAs related to the OS of AML patients. We performed a *step* function in the R platform to construct the best OS risk-score model for prognostic-related snRNAs. The calculation formula was as follows: risk score = ExpsnRNA_1_ × βsnRNA_1_ + ExpsnRNA_2_ × βsnRNA_2_ +···ExpsnRNA_n_ × βsnRNA_n_ (Exp: expression value)^57^. The nomogram model was executed using the *rms* package in the R software. The efficacy of the risk score model in predicting the OS of AML was determined using *SurvivalROC*^58^.

### Functional mechanism of risk score in AML

To evaluate the potential functional mechanism of snRNAs in the risk score model, we explored three approaches: snRNA-co-expressed genes, GSEA (https://www.gsea-msigdb.org/gsea/index.jsp), and DEGs. We extracted the mRNA expression profile matrix from TCGA RNA-Seq dataset and evaluated the co-expressed PCGs associated with snRNAs using the Pearson correlation coefficient. Subsequently, the DAVID v6.8 (https://david.ncifcrf.gov/home.jsp) was used to screen for pathways and biological functional mechanisms related to snRNA co-expression of PCGs, including Gene Ontology (GO) term and Kyoto Encyclopedia of Genes and Genomes (KEGG)^59–62^. We also used the BiNGO app in cytoscape v3.6.1 software (https://cytoscape.org) to further verify the enrichment analysis results of DAVID^63,64^. We also conducted functional enrichment analysis of the AML genome-wide dataset in GSEA v2.2.3 software using reference gene sets, including C2 (c2.all.v7.4. symbols.gmt), and C5 (c5.all.v7.4. symbols.gmt)^65–67^. We defined the GSEA results as |normalized enrichment score (NES)|> 1, *P* < 0.05, and false discovery rate (FDR) < 0.25 as reaching statistical significance. We also used risk scores to group patients into high- and low-risk phenotypes, screened DEGs between the two phenotypes using *edgeR*, and used DEGs for functional enrichment to explore the mechanism. DEGs were defined as |log2 fold change (FC)|> 2 and both P and FDR values were less than 0.05. We also used DEG to screen potential AML risk score therapeutic small-molecule compounds in the CMap ( https://portals.broadinstitute.org/cmap/)^68^ and the chemical structure was obtained from PubChem (https://pubchem.ncbi.nlm.nih.gov)^69,70^.

### Immune microenvironment and immune infiltration analysis

We used the Estimation of STromal and Immune cells in MAlignant Tumor tissues using Expression data (ESTIMATE) package to score the immune microenvironment in the R platform and calculated the stromal score, immune score, and ESTIMATE score in the bone marrow tissue of AML patients^71^. We used the gene set variation analysis (GSVA) package to perform ssGSEA in the R platform to evaluate the abundance of immune cell infiltration in the bone marrow tissues of AML patients^72^.

### Statistical analysis

Pearson correlation coefficient (*r*) was used to evaluate co-expressed PCGs, and *P* < 0.05 considered that there is a co-expression interaction relationship. Kaplan–Meier curves were evaluated using the log-rank test. We included demographic data related to AML prognosis of AML in a multivariate Cox proportional hazards regression model for adjustment. The R platform adopts R4.0.2 version. All statistical analyses were performed using the SPSS version 22.0. Differences were considered statistically significant at *P* < 0.05.

### Ethics approval and consent to participate

All raw dataset of AML included in the present study were downloaded from open access public database. The authors were not involved in any animal or human experiments and according with the ethical guidelines of the Helsinki Declaration. This study was approved by the Ethics Committee of the First Affiliated Hospital of the Guangxi Medical University. The approval number is 2021(KY-E-325).

### Supplementary Information

Supplementary Figures.

Supplementary Tables.

## Data Availability

The datasets used in this study are available from the corresponding authors upon request. The raw RNA sequencing dataset for AML was obtained from The Cancer Genome Atlas Data Portal (https://portal.gdc.cancer.gov).

## Abbreviations

AML: Acute myeloid leukemia
snRNA: Small nuclear RNA
GSEA: Gene set enrichment analysis
DEG: Differentially expressed gene
CMap: Connectivity map
ESTIMATE: Estimation of STromal and Immune cells in MAlignant Tumor tissues using expression data
MMP7: Matrix metallopeptidase 7
SIX3: SIX homeobox 3
CENPC: Centromere protein C
TREML2: Triggering receptor expressed on myeloid cells like 2
FAM83G: Family with sequence similarity 83 member G
TCGA: The Cancer Genome Atlas
HR: Hazard ratio
CI: Confidence interval
AUC: Area under the ROC curve
GO: Gene ontology
KEGG: Kyoto Encyclopedia of Genes and Genomes
OS: Overall survival
NES: Normalized enrichment score
FDR: False discovery rate
FC: Fold change
GSVA: Gene set variation analysis
ssGSEA: Single-sample gene set enrichment analysis

## References

[1] Pelcovits A, Niroula R (2020). Acute myeloid leukemia: A review. R I Med. J..

[2] Khwaja A (2016). Acute myeloid leukaemia. Nat. Rev. Dis. Primers.

[3] Karijolich J, Yu YT (2010). Spliceosomal snRNA modifications and their function. RNA Biol..

[4] Inoue D (2019). Spliceosomal disruption of the non-canonical BAF complex in cancer. Nature.

[5] Shuai S (2019). The U1 spliceosomal RNA is recurrently mutated in multiple cancers. Nature.

[6] Suzuki H (2019). Recurrent noncoding U1 snRNA mutations drive cryptic splicing in SHH medulloblastoma. Nature.

[7] Qin XG (2019). Prognostic value of small nuclear RNAs (snRNAs) for digestive tract pan- adenocarcinomas identified by RNA sequencing data. Pathol. Res. Pract..

[8] Zhang C (2022). Implications of m6A-associated snRNAs in the prognosis and immunotherapeutic responses of hepatocellular carcinoma. Front. Immunol..

[9] Xiao L, Wang J, Ju S, Cui M, Jing R (2022). Disorders and roles of tsRNA, snoRNA, snRNA and piRNA in cancer. J. Med. Genet..

[10] Spraggon L, Cartegni L (2013). U1 snRNP-dependent suppression of polyadenylation: Physiological role and therapeutic opportunities in cancer. Int J Cell Biol..

[11] Zhang L (2005). Discovery and identification of anti-U1-A snRNP antibody in lung cancer. Sci. China C Life Sci..

[12] Dong X (2020). Small nuclear RNAs (U1, U2, U5) in tumor-educated platelets are downregulated and act as promising biomarkers in lung cancer. Front. Oncol..

[13] Oh JM (2020). U1 snRNP regulates cancer cell migration and invasion in vitro. Nat. Commun..

[14] Cheng Z (2017). Gene expression profiling reveals U1 snRNA regulates cancer gene expression. Oncotarget.

[15] Li X (2022). Caspase-1 and gasdermin d afford the optimal targets with distinct switching strategies in NLRP1b inflammasome-induced cell death. Research.

[16] Xu F (2023). Specificity and competition of mRNAs dominate droplet pattern in protein phase separation. Phys. Rev. Res..

[17] Sun F, Sun J, Zhao Q (2022). A deep learning method for predicting metabolite-disease associations via graph neural network. Brief Bioinform..

[18] Wang T, Sun J, Zhao Q (2023). Investigating cardiotoxicity related with hERG channel blockers using molecular fingerprints and graph attention mechanism. Comput. Biol. Med..

[19] Hu H (2023). Gene function and cell surface protein association analysis based on single-cell multiomics data. Comput. Biol. Med..

[20] Wang W, Zhang L, Sun J, Zhao Q, Shuai J (2022). Predicting the potential human lncRNA-miRNA interactions based on graph convolution network with conditional random field. Brief Bioinform..

[21] Zhang L, Yang P, Feng H, Zhao Q, Liu H (2021). Using network distance analysis to predict lncRNA-miRNA interactions. Interdiscip. Sci..

[22] Meng R, Yin S, Sun J, Hu H, Zhao Q (2023). scAAGA: Single cell data analysis framework using asymmetric autoencoder with gene attention. Comput. Biol. Med..

[23] Gao H (2023). Predicting metabolite-disease associations based on auto-encoder and non-negative matrix factorization. Brief Bioinform..

[24] Chandran SS, Klebanoff CA (2019). T cell receptor-based cancer immunotherapy: Emerging efficacy and pathways of resistance. Immunol. Rev..

[25] Hoffmann MM, Slansky JE (2020). T-cell receptor affinity in the age of cancer immunotherapy. Mol Carcinog.

[26] Biernacki MA, Brault M, Bleakley M (2019). T-cell receptor-based immunotherapy for hematologic malignancies. Cancer J..

[27] Abbas HA (2021). Single cell T cell landscape and T cell receptor repertoire profiling of AML in context of PD-1 blockade therapy. Nat. Commun..

[28] Chapuis AG (2019). T cell receptor gene therapy targeting WT1 prevents acute myeloid leukemia relapse post-transplant. Nat. Med..

[29] Young RM, Phelan JD, Wilson WH, Staudt LM (2019). Pathogenic B-cell receptor signaling in lymphoid malignancies: New insights to improve treatment. Immunol. Rev..

[30] Burger JA, Chiorazzi N (2013). B cell receptor signaling in chronic lymphocytic leukemia. Trends Immunol..

[31] Burger JA, Wiestner A (2018). Targeting B cell receptor signalling in cancer: Preclinical and clinical advances. Nat. Rev. Cancer.

[32] Duchartre Y, Kim YM, Kahn M (2016). The Wnt signaling pathway in cancer. Crit. Rev. Oncol. Hematol..

[33] Zhan T, Rindtorff N, Boutros M (2017). Wnt signaling in cancer. Oncogene.

[34] Zhong Z, Virshup DM (2020). Wnt signaling and drug resistance in cancer. Mol. Pharmacol..

[35] Haseeb M, Pirzada RH, Ain QU, Choi S (2019). Wnt signaling in the regulation of immune cell and cancer therapeutics. Cells.

[36] Galluzzi L, Spranger S, Fuchs E, Lopez-Soto A (2019). WNT signaling in cancer immunosurveillance. Trends Cell Biol..

[37] Staal FJ, Famili F, Garcia Perez L, Pike-Overzet K (2016). Aberrant Wnt Signaling in Leukemia. Cancers.

[38] Soares-Lima SC, Pombo-de-Oliveira MS, Carneiro FRG (2020). The multiple ways Wnt signaling contributes to acute leukemia pathogenesis. J. Leukoc Biol..

[39] Dai Y (2021). Prognostic role of Wnt and Fzd gene families in acute myeloid leukaemia. J Cell Mol Med.

[40] Gruszka AM, Valli D, Alcalay M (2019). Wnt signalling in acute myeloid leukaemia. Cells.

[41] Meurette O, Mehlen P (2018). Notch signaling in the tumor microenvironment. Cancer Cell.

[42] Aster JC, Pear WS, Blacklow SC (2017). The varied roles of notch in cancer. Annu. Rev. Pathol..

[43] McCarter AC, Wang Q, Chiang M (2018). Notch in Leukemia. Adv. Exp. Med. Biol..

[44] Bellavia D, Palermo R, Felli MP, Screpanti I, Checquolo S (2018). Notch signaling as a therapeutic target for acute lymphoblastic leukemia. Expert Opin. Ther. Targets.

[45] Kannan S (2013). Notch activation inhibits AML growth and survival: A potential therapeutic approach. J. Exp. Med..

[46] Lobry C (2013). Notch pathway activation targets AML-initiating cell homeostasis and differentiation. J. Exp. Med..

[47] Aref S (2020). NOTCH-1 gene mutations influence survival in acute myeloid Leukemia patients. Asian Pac. J. Cancer Prev..

[48] He Z (2013). Chicago sky blue 6B, a vesicular glutamate transporters inhibitor, attenuates methamphetamine-induced hyperactivity and behavioral sensitization in mice. Behav. Brain Res..

[49] Baddireddy K, Poojary S (2019). A novel contrast stain for the rapid diagnosis of dermatophytoses: A cross-sectional comparative study of chicago sky blue 6b stain, potassium hydroxide mount and culture. Indian J. Dermatol..

[50] Shi J (2021). Nicardipine sensitizes temozolomide by inhibiting autophagy and promoting cell apoptosis in glioma stem cells. Aging.

[51] Deshmukh RR, Kim S, Elghoul Y, Dou QP (2017). P-Glycoprotein Inhibition sensitizes human breast cancer cells to proteasome inhibitors. J. Cell Biochem..

[52] Chen YC (2021). Nicardipine inhibits breast cancer migration via Nrf2/HO-1 axis and matrix metalloproteinase-9 regulation. Front. Pharmacol..

[53] Arisawa C, Kageyama Y, Kawakami S, Kihara K (2002). TNP-470 combined with nicardipine suppresses in vivo growth of PC-3, a human prostate cancer cell line. Urol. Oncol..

[54] Cancer Genome Atlas Research N (2013). Genomic and epigenomic landscapes of adult de novo acute myeloid leukemia. New England J. Med..

[55] Robinson MD, McCarthy DJ, Smyth GK (2010). edgeR: A Bioconductor package for differential expression analysis of digital gene expression data. Bioinformatics.

[56] Huang R, Liao X, Wang X, Li Q (2021). Comprehensive investigation of the clinical significance of long non-coding RNA HOXA-AS2 in acute myeloid leukemia using genome-wide RNA sequencing dataset. J. Cancer.

[57] Huang R, Liao X, Li Q (2017). Identification and validation of potential prognostic gene biomarkers for predicting survival in patients with acute myeloid leukemia. OncoTargets Therapy.

[58] Heagerty PJ, Zheng Y (2005). Survival model predictive accuracy and ROC curves. Biometrics.

[59] Dennis G (2003). DAVID: Database for annotation, visualization, and integrated discovery. Genome Biol..

[60] Kanehisa M, Furumichi M, Sato Y, Kawashima M, Ishiguro-Watanabe M (2023). KEGG for taxonomy-based analysis of pathways and genomes. Nucleic Acids Res..

[61] Kanehisa M (2019). Toward understanding the origin and evolution of cellular organisms. Protein Sci..

[62] Kanehisa M, Goto S (2000). KEGG: Kyoto encyclopedia of genes and genomes. Nucleic Acids Res..

[63] Maere S, Heymans K, Kuiper M (2005). BiNGO: A Cytoscape plugin to assess overrepresentation of gene ontology categories in biological networks. Bioinformatics.

[64] Shannon P (2003). Cytoscape: A software environment for integrated models of biomolecular interaction networks. Genome Res..

[65] Mootha VK (2003). PGC-1alpha-responsive genes involved in oxidative phosphorylation are coordinately downregulated in human diabetes. Nat. Genet..

[66] Liberzon A (2011). Molecular signatures database (MSigDB) 3.0. Bioinformatics.

[67] Subramanian A (2005). Gene set enrichment analysis: A knowledge-based approach for interpreting genome-wide expression profiles. Proc. Nat.l Acad. Sci. USA.

[68] Lamb J (2006). The Connectivity Map: Using gene-expression signatures to connect small molecules, genes, and disease. Science.

[69] Kim S (2021). Exploring chemical information in PubChem. Curr. Protoc.

[70] Zaslavsky L (2021). Discovering and summarizing relationships between chemicals, genes, proteins, and diseases in PubChem. Front. Res. Metr. Anal..

[71] Yoshihara K (2013). Inferring tumour purity and stromal and immune cell admixture from expression data. Nat. Commun..

[72] Hanzelmann S, Castelo R, Guinney J (2013). GSVA: Gene set variation analysis for microarray and RNA-seq data. BMC Bioinform..

