# Supplementary material for: Expression signature of ten small nuclear RNAs serves as novel biomarker for prognosis prediction of acute myeloid leukemia
Source: Sci Rep. 2023 Oct 28;13:18489. doi: 10.1038/s41598-023-45626-x (PMC10613265; doi:10.1038/s41598-023-45626-x)
Supplement: Supplementary file 1 — Supplementary Figures. [file 41598_2023_45626_MOESM1_ESM.pdf]

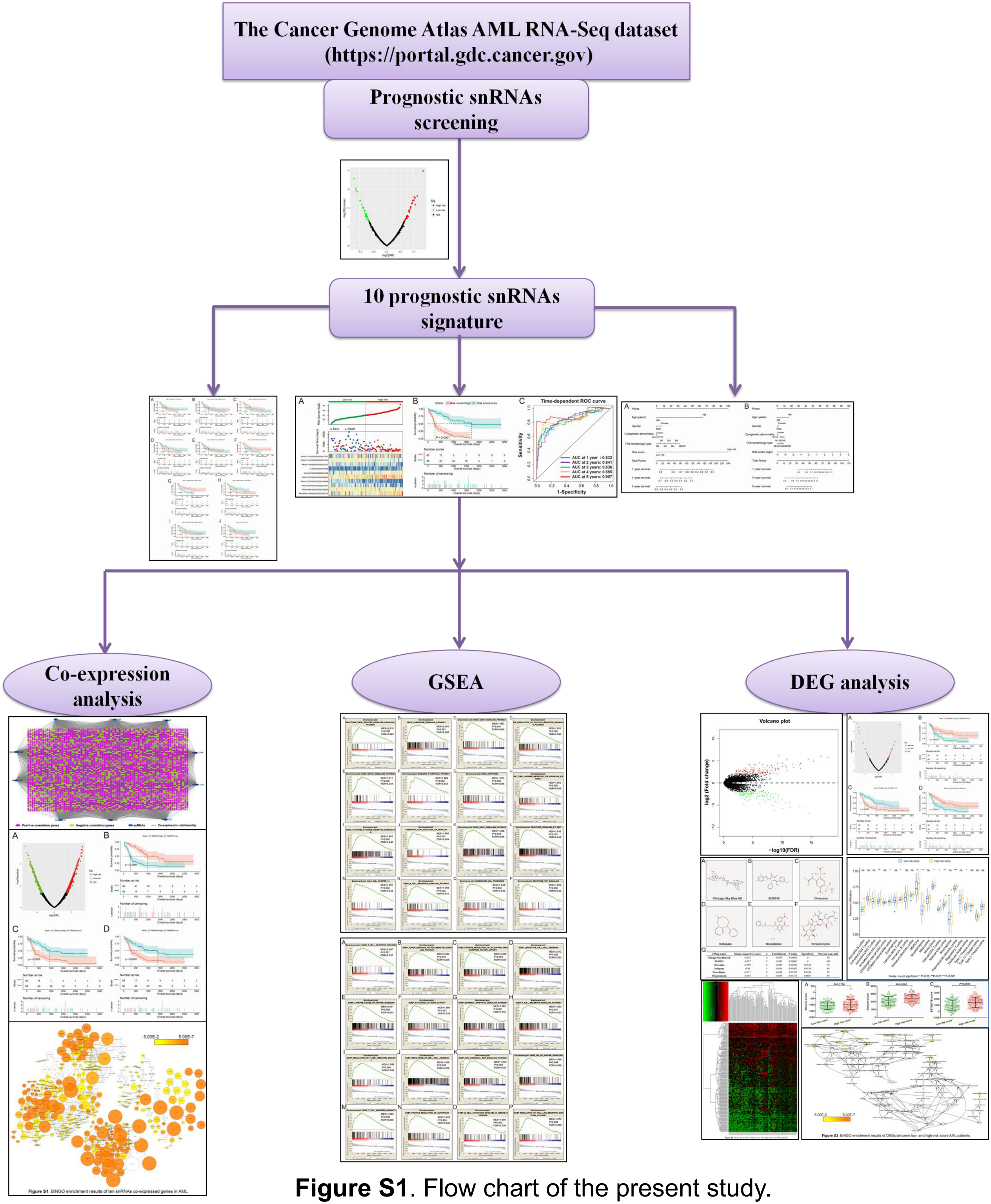

Figure S1. Flow chart of the present study.

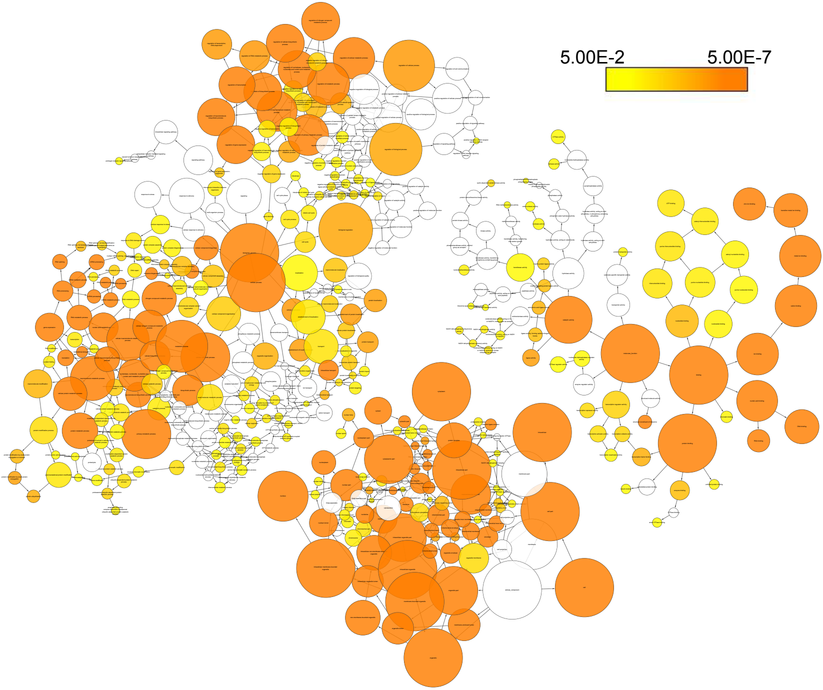

Figure S2. BiNGO enrichment results of ten snRNAs co-expressed genes in AML.

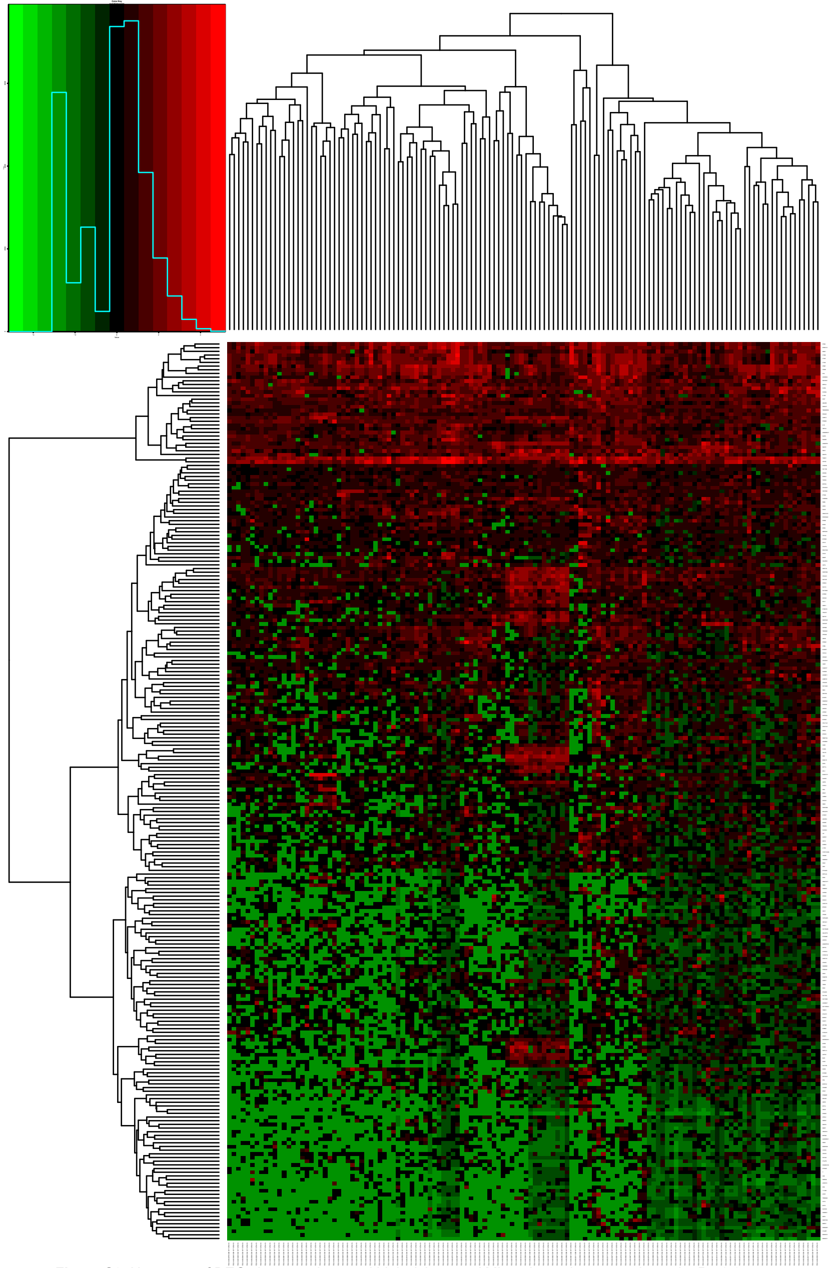

Figure S3. Heat map of DEGs between low- and high-risk score AML patients (heat map drawn by R4.0.2 version:https://www.r-project.org).

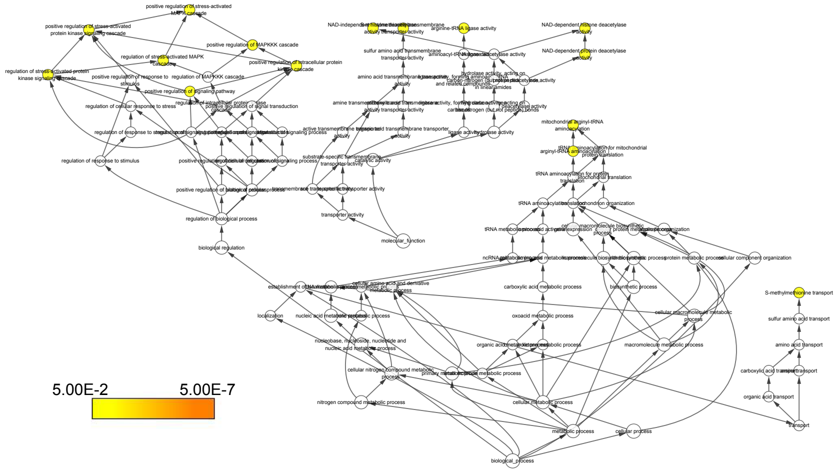

Figure S4. BiNGO enrichment results of DEGs between low- and high-risk score AML patients.
