# Supplementary material for: Expression signature of ten small nuclear RNAs serves as novel biomarker for prognosis prediction of acute myeloid leukemia
Source: Sci Rep. 2023 Oct 28;13:18489. doi: 10.1038/s41598-023-45626-x (PMC10613265; doi:10.1038/s41598-023-45626-x)
Supplement: Supplementary file 2 — Supplementary Tables. [file 41598_2023_45626_MOESM2_ESM.docx]

| **Table S1. Clinical parameters of 130 AML patients in TCGA cohort.** | | | | |
| --- | --- | --- | --- | --- |
| **Variables** | **Events/total(n=130)** | **MST (days)** | **HR (95% CI)** | **Log-rank *P*** |
| **Age (years)** |  |  |  | <0.0001 |
| ≤65 | 48/96 | 822 | 1 |  |
| >65 | 30/34 | 214 | 3.236(2.022-5.180) |  |
| **Gender** |  |  |  | 0.916 |
| Female | 36/60 | 671 | 1 |  |
| Male | 42/70 | 577 | 0.976(0.625-1.526) |  |
| **Cytogenetics abnormality** |  |  |  | 0.19 |
| Normal | 42/61 | 518 | 1 |  |
| Complex | 8/15 | 366 | 0.984（0.460-2.105） |  |
| Others | 21/42 | 945 | 0.625(0.369-1.056) |  |
| **FAB morphology type** |  |  |  | 0.029 |
| M0 | 7/12 | 792 | 1 |  |
| M1 | 19/30 | 731 | 1.438(0.604-3.424) |  |
| M2 | 19/32 | 486 | 1.240(0.520-2.955) |  |
| M3 | 3/14 | NA | 0.278(0.072-1.083) |  |
| M4 | 19/27 | 580 | 1.451(0.609-3.458) |  |
| M5 | 8/12 | 214 | 2.027(0.728-5.644) |  |
| M6+M7 | 3/3 | 304 | 3.361(0.851-13.272) |  |
| **Risk score** |  |  |  | <0.0001 |
| Low risk | 23/65 | 1706 | 1 |  |
| High risk | 55/65 | 273 | 5.392(3.210-9.058) |  |

**Notes**: §Information of cytogenetics abnormality was unavailable in 12 patients.

**Abbreviations**: MST, median survival time ; HR, hazard ratio; 95%CI, 95% confidence interval; FAB, French–American–British.

Table S2. Survival analysis results of the snRNAs in AML.

| ID | P | HR | Low 95%CI | High 95%CI |
| --- | --- | --- | --- | --- |
| RNU6-450P|ENSG00000201524 | 0.000107 | 2.547464 | 1.587420996 | 4.088122931 |
| RNU6-1143P|ENSG00000251934 | 0.000273 | 0.426714 | 0.269758037 | 0.674993163 |
| RNU6-26P|ENSG00000206712 | 0.000891 | 0.452836 | 0.283786571 | 0.722588345 |
| RNU6-1272P|ENSG00000199646 | 0.001143 | 0.45104 | 0.279165911 | 0.728731709 |
| RNU6-202P|ENSG00000251807 | 0.001349 | 0.468148 | 0.294325617 | 0.744625366 |
| RNU6ATAC6P|ENSG00000221059 | 0.002249 | 2.191158 | 1.324718999 | 3.624296786 |
| RNU6-1274P|ENSG00000207287 | 0.002548 | 2.02669 | 1.280923906 | 3.206648007 |
| RNU2-22P|ENSG00000223198 | 0.002887 | 2.074979 | 1.283734399 | 3.353914817 |
| RNU1-32P|ENSG00000200807 | 0.003733 | 2.008408 | 1.253611288 | 3.21766484 |
| U1|ENSG00000278099 | 0.003877 | 1.955643 | 1.240514241 | 3.083028722 |
| RNU6-613P|ENSG00000222344 | 0.003879 | 0.509788 | 0.322696254 | 0.805350395 |
| RNU4-14P|ENSG00000222790 | 0.003991 | 1.955237 | 1.238655831 | 3.086371086 |
| RNU6-529P|ENSG00000200253 | 0.004071 | 1.966874 | 1.239752676 | 3.120454295 |
| RNU6-766P|ENSG00000199348 | 0.004089 | 0.501004 | 0.312569532 | 0.803037843 |
| RNU6-9|ENSG00000207507 | 0.005352 | 1.943594 | 1.21758316 | 3.102503855 |
| RNU4-23P|ENSG00000199709 | 0.005479 | 1.91404 | 1.210562373 | 3.026320332 |
| RNU6ATAC39P|ENSG00000252118 | 0.005643 | 1.903129 | 1.206614834 | 3.001702776 |
| RNU6-260P|ENSG00000206819 | 0.006413 | 0.525597 | 0.330975771 | 0.834659794 |
| RNU6-1004P|ENSG00000252393 | 0.006605 | 2.080141 | 1.226189565 | 3.528808787 |
| RNU6-6P|ENSG00000272055 | 0.008537 | 1.845672 | 1.168990619 | 2.914056671 |
| RNU6-761P|ENSG00000206875 | 0.008653 | 1.861214 | 1.170547774 | 2.959397141 |
| RNU4-8P|ENSG00000201806 | 0.0087 | 1.848034 | 1.168063504 | 2.923839031 |
| RNU6-1096P|ENSG00000252898 | 0.008818 | 0.541205 | 0.341838521 | 0.856846338 |
| RNU6-110P|ENSG00000207237 | 0.009088 | 0.542895 | 0.343086512 | 0.859067581 |
| RNU6-946P|ENSG00000206605 | 0.009465 | 0.539918 | 0.338955868 | 0.860027371 |
| RNU6-522P|ENSG00000201367 | 0.010086 | 0.550097 | 0.348907787 | 0.867296386 |
| RNU6-1092P|ENSG00000252764 | 0.010147 | 1.837923 | 1.155593603 | 2.923139488 |
| RNU6-1242P|ENSG00000212354 | 0.012714 | 1.775448 | 1.130321165 | 2.78877836 |
| RNU6-430P|ENSG00000252887 | 0.012851 | 0.559947 | 0.354597134 | 0.884215085 |
| RNU5F-3P|ENSG00000200637 | 0.013658 | 1.80091 | 1.128340559 | 2.874377924 |
| RNU6-878P|ENSG00000207308 | 0.01458 | 1.771145 | 1.1195823 | 2.801896022 |
| RNU4-5P|ENSG00000272160 | 0.01479 | 0.567011 | 0.359299419 | 0.894801048 |
| RNU6-542P|ENSG00000252214 | 0.016718 | 1.790169 | 1.111095376 | 2.884274725 |
| RNU11-2P|ENSG00000252707 | 0.019616 | 0.573201 | 0.359179848 | 0.914748007 |
| RNU6-780P|ENSG00000212332 | 0.020092 | 0.576932 | 0.362845425 | 0.917333923 |
| RNU6-1025P|ENSG00000222561 | 0.020378 | 0.587387 | 0.374671713 | 0.920869843 |
| RNU6-677P|ENSG00000252723 | 0.020465 | 0.583891 | 0.370451397 | 0.920305544 |
| RNU6-1318P|ENSG00000202259 | 0.021408 | 0.581066 | 0.365907139 | 0.922741593 |
| RNU6-373P|ENSG00000223179 | 0.021537 | 0.586491 | 0.37209035 | 0.924430226 |
| RNU6-45P|ENSG00000207200 | 0.02461 | 1.706896 | 1.070791434 | 2.720879184 |
| RNU6-60P|ENSG00000201662 | 0.024631 | 0.589381 | 0.371650618 | 0.934669146 |
| RNU4ATAC11P|ENSG00000251748 | 0.026086 | 0.577423 | 0.355952899 | 0.93669024 |
| RNU6-725P|ENSG00000252371 | 0.026301 | 1.678947 | 1.062954755 | 2.651911012 |
| RNU4ATAC|ENSG00000264229 | 0.026873 | 0.593864 | 0.374354686 | 0.942087462 |
| RNU6-531P|ENSG00000252503 | 0.027541 | 0.604248 | 0.386042175 | 0.945792426 |
| U6|ENSG00000278757 | 0.028237 | 1.735505 | 1.060564148 | 2.839975864 |
| RNU6-1105P|ENSG00000207505 | 0.028378 | 1.676373 | 1.056215992 | 2.660655703 |
| RNU6-1169P|ENSG00000200407 | 0.028591 | 0.601718 | 0.38183524 | 0.948222461 |
| RNU6-118P|ENSG00000252361 | 0.029466 | 0.579834 | 0.354996741 | 0.947073109 |
| RNU4-51P|ENSG00000201076 | 0.029698 | 1.661222 | 1.051259679 | 2.625097052 |
| RNU6-370P|ENSG00000222940 | 0.033711 | 0.612867 | 0.390040903 | 0.962992674 |
| RNU6-1136P|ENSG00000252643 | 0.034269 | 1.640997 | 1.037400305 | 2.595789456 |
| RNU6-834P|ENSG00000199237 | 0.034363 | 1.689939 | 1.03939742 | 2.747645006 |
| RNU4ATAC18P|ENSG00000251988 | 0.034527 | 1.676207 | 1.038321387 | 2.70597178 |
| RNU6-1162P|ENSG00000207172 | 0.037863 | 0.59377 | 0.363015337 | 0.971207765 |
| RNU6-584P|ENSG00000222282 | 0.038577 | 1.614187 | 1.025480872 | 2.540855885 |
| RNU6-516P|ENSG00000223313 | 0.038579 | 1.625728 | 1.025859631 | 2.576367621 |
| RNU7-186P|ENSG00000238419 | 0.039004 | 1.619318 | 1.024627044 | 2.559166117 |
| RNU2-39P|ENSG00000222536 | 0.039935 | 0.621894 | 0.395291438 | 0.978397177 |
| U1|ENSG00000274428 | 0.039962 | 1.604677 | 1.021918243 | 2.519760668 |
| RNU4-62P|ENSG00000222057 | 0.040394 | 1.620418 | 1.021357191 | 2.570848488 |
| RNU1-67P|ENSG00000207175 | 0.040738 | 1.616808 | 1.020451807 | 2.561676224 |
| RNU6-82P|ENSG00000200840 | 0.041345 | 0.624316 | 0.397046354 | 0.981675993 |
| RNU4-53P|ENSG00000222760 | 0.041849 | 0.622038 | 0.39376583 | 0.982643385 |
| RNU6-198P|ENSG00000206985 | 0.043122 | 0.620923 | 0.391270304 | 0.985367131 |
| RNU6-536P|ENSG00000200254 | 0.043741 | 0.629076 | 0.400918082 | 0.987074662 |
| RNU6-1322P|ENSG00000201179 | 0.043893 | 0.624469 | 0.395019382 | 0.987196386 |
| RNU6-1251P|ENSG00000201372 | 0.046413 | 1.598262 | 1.007481642 | 2.53547187 |
| RNU1-56P|ENSG00000212605 | 0.046947 | 1.599533 | 1.006360445 | 2.542335155 |
| RNU6-487P|ENSG00000202300 | 0.047184 | 0.621053 | 0.387998889 | 0.994092784 |
| RNU7-125P|ENSG00000238964 | 0.048902 | 1.574844 | 1.00218884 | 2.474717644 |
| RNU6-223P|ENSG00000199700 | 0.049124 | 0.625153 | 0.391521555 | 0.998198938 |
| RNU4-39P|ENSG00000199325 | 0.050518 | 1.565035 | 0.998990397 | 2.451808542 |
| RNU4-78P|ENSG00000222872 | 0.052382 | 0.636736 | 0.403551939 | 1.004661284 |
| RNU6-564P|ENSG00000222915 | 0.053822 | 1.556226 | 0.992756253 | 2.439509833 |
| RNU6-91P|ENSG00000272439 | 0.057179 | 1.557174 | 0.986580048 | 2.457772997 |
| RNU2-68P|ENSG00000222810 | 0.059872 | 1.538843 | 0.982241143 | 2.410852195 |
| RNU6-341P|ENSG00000252782 | 0.060345 | 0.647764 | 0.411747247 | 1.019066993 |
| RNU6-322P|ENSG00000251819 | 0.060366 | 1.559042 | 0.980831645 | 2.478112365 |
| RNU6-455P|ENSG00000207412 | 0.060966 | 0.647837 | 0.411391798 | 1.020177873 |
| RNU6-307P|ENSG00000252552 | 0.060973 | 0.642428 | 0.404390198 | 1.020584302 |
| RNU6-343P|ENSG00000201579 | 0.062856 | 0.651599 | 0.414939625 | 1.02323551 |
| RNU6-479P|ENSG00000252157 | 0.063132 | 0.650171 | 0.412878656 | 1.023841449 |
| RNU6-1128P|ENSG00000199695 | 0.063737 | 1.533725 | 0.975854474 | 2.410516048 |
| RNU6-1199P|ENSG00000223181 | 0.064033 | 0.654599 | 0.418039538 | 1.025022523 |
| RNU6-1079P|ENSG00000199731 | 0.064372 | 1.53733 | 0.974665413 | 2.424816427 |
| RNU6-123P|ENSG00000251972 | 0.065054 | 0.648214 | 0.408971522 | 1.027410378 |
| RNU4-49P|ENSG00000251889 | 0.065136 | 0.653286 | 0.415541102 | 1.027052839 |
| RNU6-1112P|ENSG00000200455 | 0.065302 | 0.653311 | 0.415456929 | 1.027338639 |
| RNU4-24P|ENSG00000201435 | 0.065912 | 0.642731 | 0.401266662 | 1.029497108 |
| RNU6-481P|ENSG00000206921 | 0.069113 | 1.519822 | 0.967765048 | 2.386798707 |
| RNU1-85P|ENSG00000200997 | 0.069632 | 1.52318 | 0.966779199 | 2.399801464 |
| RNU6-519P|ENSG00000222522 | 0.069718 | 1.525309 | 0.966535522 | 2.407120108 |
| RNU6-378P|ENSG00000207052 | 0.069739 | 0.657786 | 0.418300391 | 1.034382852 |
| RNU6-553P|ENSG00000200917 | 0.073342 | 0.654437 | 0.411462024 | 1.040891393 |
| RNU6-1214P|ENSG00000200520 | 0.075048 | 0.661087 | 0.419144255 | 1.042685124 |
| RNU1-83P|ENSG00000200296 | 0.076846 | 0.662974 | 0.420490276 | 1.045291351 |
| RNU6-238P|ENSG00000200183 | 0.07765 | 1.508174 | 0.955499633 | 2.38052304 |
| RNU6-879P|ENSG00000201198 | 0.079567 | 1.49709 | 0.953526161 | 2.350516976 |
| RNU6-729P|ENSG00000207128 | 0.082326 | 1.519371 | 0.947812192 | 2.435595915 |
| RNU6-527P|ENSG00000200295 | 0.08327 | 0.657446 | 0.409025249 | 1.056745694 |
| RNU2-11P|ENSG00000239122 | 0.084867 | 1.494545 | 0.946266998 | 2.360502815 |
| U1|ENSG00000273768 | 0.08577 | 0.658673 | 0.409089398 | 1.060525655 |
| RNU6-1165P|ENSG00000222051 | 0.091964 | 1.470004 | 0.939097877 | 2.301049111 |
| RNU6-628P|ENSG00000222607 | 0.093963 | 0.670756 | 0.420343597 | 1.070345798 |
| RNU2-5P|ENSG00000222465 | 0.094386 | 0.680348 | 0.43324781 | 1.068380548 |
| RNU6-147P|ENSG00000206710 | 0.095567 | 1.490858 | 0.932153309 | 2.384433108 |
| RNU5E-3P|ENSG00000251722 | 0.096736 | 1.467871 | 0.93322107 | 2.308826963 |
| RNU6-1141P|ENSG00000201390 | 0.097471 | 1.551759 | 0.922874282 | 2.609190958 |
| RNU4-21P|ENSG00000199672 | 0.09783 | 1.465929 | 0.932064557 | 2.305577432 |
| RNU6-218P|ENSG00000252929 | 0.100008 | 0.683919 | 0.434907519 | 1.075506467 |
| RNU2-7P|ENSG00000222726 | 0.100845 | 1.467079 | 0.928151026 | 2.318932374 |
| RNU6-245P|ENSG00000206747 | 0.101016 | 0.668452 | 0.413051781 | 1.081771965 |
| RNU6-79P|ENSG00000199381 | 0.103187 | 0.678797 | 0.425959535 | 1.08171089 |
| RNU6-820P|ENSG00000207000 | 0.104019 | 0.677388 | 0.423535681 | 1.083390349 |
| RNU6-833P|ENSG00000200356 | 0.105299 | 0.684795 | 0.433086538 | 1.082795732 |
| U7|ENSG00000272215 | 0.106988 | 0.684336 | 0.431485167 | 1.085359139 |
| RNU7-172P|ENSG00000252934 | 0.109141 | 0.694413 | 0.444480844 | 1.084881053 |
| RNU6-444P|ENSG00000206715 | 0.109432 | 1.456369 | 0.919097868 | 2.307708962 |
| RNU6-1228P|ENSG00000212316 | 0.10964 | 1.478898 | 0.915679767 | 2.388542215 |
| RNVU1-1|ENSG00000207340 | 0.109742 | 0.686948 | 0.433589555 | 1.08835121 |
| RNU6-893P|ENSG00000238444 | 0.112383 | 1.450826 | 0.91641145 | 2.296890499 |
| RNU6-987P|ENSG00000207490 | 0.112623 | 1.475631 | 0.912481244 | 2.386334822 |
| RNU4-48P|ENSG00000202429 | 0.112714 | 1.444868 | 0.9169128 | 2.276819357 |
| RNU7-124P|ENSG00000251745 | 0.113912 | 0.694072 | 0.441342243 | 1.09152432 |
| RNU6-1062P|ENSG00000206635 | 0.116399 | 0.698887 | 0.446864297 | 1.09304567 |
| RNU7-103P|ENSG00000238370 | 0.117166 | 0.69648 | 0.442991563 | 1.095020103 |
| RNU7-84P|ENSG00000251892 | 0.11784 | 1.453075 | 0.909705216 | 2.321002021 |
| RNU6-701P|ENSG00000206786 | 0.118055 | 1.448764 | 0.910141879 | 2.306143901 |
| RNU6-85P|ENSG00000271932 | 0.119287 | 0.693085 | 0.43698585 | 1.099274372 |
| RNU1-87P|ENSG00000200597 | 0.119879 | 0.699784 | 0.446259765 | 1.097337476 |
| RNU7-107P|ENSG00000238523 | 0.1203 | 1.43459 | 0.909902639 | 2.261832321 |
| RNU6-204P|ENSG00000199335 | 0.120855 | 1.431155 | 0.909858323 | 2.251124094 |
| RNU6-759P|ENSG00000252549 | 0.122184 | 1.456788 | 0.904074183 | 2.347408161 |
| RNU6-90P|ENSG00000272337 | 0.123395 | 1.426736 | 0.907812766 | 2.242284674 |
| RNU6-254P|ENSG00000200247 | 0.12771 | 0.700612 | 0.443247064 | 1.107411136 |
| RNU6-574P|ENSG00000206992 | 0.128104 | 0.699961 | 0.442096606 | 1.108231926 |
| RNU4-31P|ENSG00000206938 | 0.129531 | 0.68906 | 0.425736186 | 1.115252477 |
| RNU6-856P|ENSG00000252768 | 0.131371 | 1.413254 | 0.901716545 | 2.214982781 |
| RNU6-342P|ENSG00000207251 | 0.134093 | 1.406282 | 0.900236504 | 2.196788141 |
| RNU6-122P|ENSG00000252627 | 0.134553 | 0.704933 | 0.445915266 | 1.114405949 |
| RNU6ATAC34P|ENSG00000221806 | 0.135556 | 1.41458 | 0.897074063 | 2.230625687 |
| RNU6-807P|ENSG00000252614 | 0.136523 | 0.704143 | 0.443730402 | 1.11738396 |
| RNU6-565P|ENSG00000222869 | 0.137795 | 0.705907 | 0.445646194 | 1.118160941 |
| RNU5F-4P|ENSG00000252390 | 0.138023 | 1.403278 | 0.896810591 | 2.195769862 |
| RNU6-4P|ENSG00000206932 | 0.138196 | 1.406417 | 0.895989621 | 2.207625205 |
| RNU6-748P|ENSG00000207378 | 0.138922 | 0.709528 | 0.450385056 | 1.117776365 |
| RNU6-1176P|ENSG00000212257 | 0.139236 | 0.707786 | 0.44765591 | 1.119075646 |
| RNU6-331P|ENSG00000207455 | 0.143469 | 0.69099 | 0.4211275 | 1.13378365 |
| RNU6-1091P|ENSG00000200815 | 0.143776 | 0.715204 | 0.456311242 | 1.120982805 |
| RNU2-17P|ENSG00000222222 | 0.143876 | 1.407343 | 0.889994575 | 2.225424265 |
| RNU4-54P|ENSG00000252237 | 0.146201 | 0.717023 | 0.457795394 | 1.123038673 |
| RNU4-52P|ENSG00000206936 | 0.146317 | 1.418183 | 0.885139751 | 2.272230744 |
| RNU7-151P|ENSG00000252739 | 0.148614 | 1.395577 | 0.88784547 | 2.193665082 |
| RNU6-1337P|ENSG00000252334 | 0.148874 | 1.428566 | 0.880200764 | 2.318561327 |
| RNU6-979P|ENSG00000206623 | 0.149053 | 0.702375 | 0.434663966 | 1.134969733 |
| RNU6-1305P|ENSG00000201223 | 0.15026 | 1.399036 | 0.885425852 | 2.210576141 |
| RNU6-862P|ENSG00000199674 | 0.15292 | 0.714133 | 0.45005864 | 1.133154733 |
| RNU6-345P|ENSG00000252810 | 0.153492 | 0.716634 | 0.453516075 | 1.13240536 |
| RNU6-1054P|ENSG00000223225 | 0.15569 | 1.392623 | 0.881590198 | 2.199888037 |
| RNU6-950P|ENSG00000207381 | 0.156099 | 1.383437 | 0.883421816 | 2.166460596 |
| RNU6-680P|ENSG00000207333 | 0.156823 | 1.393819 | 0.880195902 | 2.207158099 |
| RNU6-608P|ENSG00000206654 | 0.157635 | 0.716617 | 0.451404905 | 1.13764747 |
| RNU2-46P|ENSG00000252847 | 0.157947 | 1.399319 | 0.877757913 | 2.230790666 |
| RNU6-1010P|ENSG00000199627 | 0.158191 | 0.712242 | 0.444574551 | 1.141065102 |
| RNU6-1309P|ENSG00000200175 | 0.161315 | 0.725327 | 0.46278254 | 1.136817156 |
| RNU6-10P|ENSG00000206763 | 0.161634 | 0.704326 | 0.431121858 | 1.150662226 |
| RNU7-181P|ENSG00000253043 | 0.1621 | 1.390824 | 0.875818706 | 2.208665875 |
| RNU6-1016P|ENSG00000252498 | 0.165597 | 0.72097 | 0.454011924 | 1.144899842 |
| RNU7-23P|ENSG00000239099 | 0.170562 | 1.370112 | 0.873304685 | 2.149543491 |
| RNU6-1020P|ENSG00000200554 | 0.171122 | 1.380591 | 0.869920696 | 2.191041135 |
| RNU6-1237P|ENSG00000207508 | 0.177545 | 0.734452 | 0.468952214 | 1.15026726 |
| RNU6-466P|ENSG00000212526 | 0.179371 | 0.731674 | 0.463723008 | 1.15445395 |
| RNU6-22P|ENSG00000207083 | 0.180807 | 0.729873 | 0.460221444 | 1.157518514 |
| RNU6-1050P|ENSG00000222320 | 0.180907 | 1.358732 | 0.867178651 | 2.128918307 |
| RNU6-1111P|ENSG00000212420 | 0.182465 | 1.366789 | 0.863385177 | 2.163707686 |
| RNU6-29P|ENSG00000207367 | 0.186555 | 0.735406 | 0.466090163 | 1.160338392 |
| RNU6-855P|ENSG00000222327 | 0.187746 | 0.739828 | 0.47250973 | 1.158377939 |
| RNU7-50P|ENSG00000238304 | 0.18789 | 1.370498 | 0.857337328 | 2.190813147 |
| RNU4-68P|ENSG00000201184 | 0.188637 | 0.740305 | 0.472842012 | 1.159059003 |
| RNU6-534P|ENSG00000201747 | 0.191775 | 0.739155 | 0.469507645 | 1.163664662 |
| RNU2-51P|ENSG00000222640 | 0.192252 | 1.34767 | 0.860609573 | 2.110382463 |
| RNU6-47P|ENSG00000206593 | 0.19351 | 0.736784 | 0.464916087 | 1.16763137 |
| RNU2-69P|ENSG00000251870 | 0.193737 | 1.395381 | 0.84426356 | 2.306256275 |
| RNU6-890P|ENSG00000206848 | 0.195947 | 1.34623 | 0.857876537 | 2.112581228 |
| RNU6-571P|ENSG00000206855 | 0.196698 | 1.350823 | 0.85571612 | 2.132393345 |
| RNU11|ENSG00000274978 | 0.197537 | 1.352931 | 0.854289853 | 2.142625213 |
| RNU1-73P|ENSG00000206698 | 0.197537 | 0.744621 | 0.475497835 | 1.166062905 |
| RNU11-3P|ENSG00000212413 | 0.199655 | 0.741501 | 0.469479686 | 1.171133151 |
| RNU7-70P|ENSG00000252750 | 0.200853 | 0.744553 | 0.473811572 | 1.170000281 |
| RNU6-883P|ENSG00000207327 | 0.201462 | 1.341159 | 0.854834051 | 2.104160385 |
| RNU6-917P|ENSG00000252103 | 0.203517 | 1.368037 | 0.843965108 | 2.217537893 |
| RNU1-77P|ENSG00000212170 | 0.204322 | 1.342656 | 0.851874876 | 2.116185411 |
| RNU6-1198P|ENSG00000252713 | 0.207924 | 0.746494 | 0.473593114 | 1.176651327 |
| RNU6-1306P|ENSG00000202089 | 0.208263 | 1.343991 | 0.848030358 | 2.130007839 |
| U1|ENSG00000275291 | 0.209858 | 1.348487 | 0.845071008 | 2.15179348 |
| RNU6-646P|ENSG00000201441 | 0.210208 | 0.747959 | 0.474891732 | 1.178043568 |
| RNU6-485P|ENSG00000200345 | 0.210348 | 1.334777 | 0.849534834 | 2.097183716 |
| RNU6-972P|ENSG00000212510 | 0.210985 | 0.748434 | 0.475296944 | 1.178532202 |
| U4|ENSG00000278374 | 0.211747 | 1.332988 | 0.849005377 | 2.092868157 |
| RNU6-936P|ENSG00000206732 | 0.212096 | 0.752386 | 0.481243696 | 1.176294588 |
| RNU6-964P|ENSG00000252333 | 0.212913 | 0.747376 | 0.472662605 | 1.181754518 |
| RNU1-36P|ENSG00000202000 | 0.215633 | 1.3263 | 0.848231154 | 2.073811053 |
| RNU6-681P|ENSG00000200882 | 0.215898 | 0.754598 | 0.483105635 | 1.178661487 |
| RNU6-1043P|ENSG00000222287 | 0.216143 | 0.75162 | 0.478078966 | 1.181671392 |
| RNU6-662P|ENSG00000206980 | 0.216311 | 1.334117 | 0.844761256 | 2.106947118 |
| RNU6-1208P|ENSG00000238482 | 0.2171 | 0.753189 | 0.480212722 | 1.18133752 |
| RNU6-415P|ENSG00000252061 | 0.219578 | 0.755504 | 0.482880951 | 1.182044282 |
| RNU5B-2P|ENSG00000199906 | 0.219878 | 1.324709 | 0.845330002 | 2.075939802 |
| RNU6-1316P|ENSG00000206969 | 0.224444 | 1.338855 | 0.836104099 | 2.143911078 |
| RNU6-483P|ENSG00000206815 | 0.225072 | 1.32517 | 0.840872391 | 2.088396225 |
| RNU6-418P|ENSG00000206762 | 0.225173 | 0.746261 | 0.465037528 | 1.197548162 |
| RNU6-1082P|ENSG00000201444 | 0.226459 | 1.347755 | 0.830969486 | 2.185932323 |
| RNU4-47P|ENSG00000222808 | 0.226726 | 1.325797 | 0.839260894 | 2.094386491 |
| RNU6-611P|ENSG00000207003 | 0.230644 | 1.322939 | 0.837169781 | 2.090575324 |
| RNU5A-3P|ENSG00000254172 | 0.23101 | 1.316738 | 0.839374434 | 2.065585347 |
| RNU6-490P|ENSG00000199855 | 0.231899 | 0.760432 | 0.485356635 | 1.191404862 |
| RNU6-74P|ENSG00000206962 | 0.232522 | 0.76089 | 0.485838822 | 1.191658208 |
| RNU6-1024P|ENSG00000206926 | 0.232679 | 0.746899 | 0.462519275 | 1.206128515 |
| RNU1-100P|ENSG00000202125 | 0.23455 | 1.326782 | 0.832391052 | 2.114812488 |
| RNU1-134P|ENSG00000199805 | 0.234557 | 1.359959 | 0.819153087 | 2.257804649 |
| RNU6-197P|ENSG00000252489 | 0.235332 | 0.758472 | 0.480454287 | 1.197364836 |
| RNU6-1026P|ENSG00000207194 | 0.235676 | 1.310626 | 0.8381336 | 2.049481847 |
| RNU6-136P|ENSG00000207393 | 0.241543 | 1.313346 | 0.832271464 | 2.072494878 |
| RNU1-11P|ENSG00000206702 | 0.242317 | 1.323679 | 0.82725113 | 2.11800972 |
| RNU6-1017P|ENSG00000200483 | 0.244441 | 0.750698 | 0.463194609 | 1.216652871 |
| RNU6-1042P|ENSG00000206931 | 0.248176 | 1.320842 | 0.823640278 | 2.118185039 |
| RNU6-933P|ENSG00000207153 | 0.249657 | 0.76592 | 0.486405127 | 1.206059402 |
| RNU6ATAC23P|ENSG00000221375 | 0.250339 | 0.763085 | 0.481235175 | 1.210008287 |
| RNU7-193P|ENSG00000252532 | 0.250945 | 1.306694 | 0.827640985 | 2.063030441 |
| RNU6-126P|ENSG00000252494 | 0.251182 | 1.307168 | 0.827239376 | 2.065530568 |
| RNU6-1280P|ENSG00000202081 | 0.25444 | 1.307498 | 0.82448545 | 2.073475813 |
| RNU6-549P|ENSG00000207162 | 0.254781 | 0.757878 | 0.470307645 | 1.221282313 |
| RNU6-645P|ENSG00000201519 | 0.255564 | 0.769506 | 0.489839174 | 1.208845164 |
| RNU6-665P|ENSG00000207369 | 0.257632 | 0.770712 | 0.490970828 | 1.209840371 |
| RNU6-722P|ENSG00000223309 | 0.258381 | 1.298321 | 0.825585702 | 2.041747796 |
| RNU6-957P|ENSG00000200522 | 0.261157 | 0.772843 | 0.493052861 | 1.211405668 |
| U1|ENSG00000274210 | 0.261497 | 0.773505 | 0.49405443 | 1.211021374 |
| RNU6-407P|ENSG00000202150 | 0.263501 | 0.77527 | 0.496227247 | 1.211227529 |
| RNU6-59P|ENSG00000207518 | 0.264698 | 1.300875 | 0.819397204 | 2.06526977 |
| RNU6-958P|ENSG00000252804 | 0.265418 | 1.289976 | 0.8240785 | 2.019270992 |
| RNU6-928P|ENSG00000252685 | 0.265984 | 0.774623 | 0.493933365 | 1.214820645 |
| RNU6-1053P|ENSG00000251739 | 0.266316 | 0.774052 | 0.492775458 | 1.21588294 |
| RNU6-644P|ENSG00000212457 | 0.266957 | 0.769204 | 0.483993352 | 1.222486261 |
| RNU6-892P|ENSG00000222267 | 0.268407 | 1.297703 | 0.817991602 | 2.058742216 |
| RNU7-48P|ENSG00000238386 | 0.268463 | 0.77453 | 0.49261747 | 1.217775455 |
| RNU6-795P|ENSG00000252132 | 0.268888 | 0.774912 | 0.493092293 | 1.21780141 |
| RNU6-1034P|ENSG00000202205 | 0.271701 | 0.776858 | 0.495225176 | 1.218653089 |
| RNU6-634P|ENSG00000239189 | 0.272464 | 1.302907 | 0.812229318 | 2.090007973 |
| RNU6-291P|ENSG00000207451 | 0.272935 | 1.298865 | 0.813841174 | 2.072947488 |
| RNU6-1003P|ENSG00000252908 | 0.273637 | 0.752225 | 0.45181927 | 1.252365454 |
| RNU6-675P|ENSG00000206963 | 0.275465 | 0.779608 | 0.498374152 | 1.219541441 |
| RNU6-431P|ENSG00000206601 | 0.276122 | 0.777182 | 0.493742829 | 1.223333398 |
| RNU6-540P|ENSG00000207352 | 0.279433 | 0.779637 | 0.496581359 | 1.224037577 |
| RNU6-5P|ENSG00000206965 | 0.279684 | 1.28763 | 0.814205931 | 2.036327349 |
| RNU6-199P|ENSG00000199824 | 0.27973 | 0.780519 | 0.498023434 | 1.223254413 |
| RNU7-38P|ENSG00000238446 | 0.280339 | 0.780279 | 0.497309421 | 1.224258198 |
| RNU6-819P|ENSG00000200986 | 0.280448 | 1.289948 | 0.812419498 | 2.048159927 |
| RNU6-1281P|ENSG00000252032 | 0.280976 | 1.291177 | 0.811374348 | 2.054709456 |
| RNU1-1|ENSG00000206652 | 0.281182 | 1.283606 | 0.815127839 | 2.021333983 |
| RNU6-421P|ENSG00000201341 | 0.287127 | 1.277206 | 0.813959477 | 2.004098225 |
| RNU6-1095P|ENSG00000251957 | 0.289215 | 1.286609 | 0.807324058 | 2.050431397 |
| RNU6-501P|ENSG00000200942 | 0.289301 | 0.781146 | 0.494682637 | 1.233495012 |
| RNU6-1164P|ENSG00000201298 | 0.291837 | 0.783103 | 0.497037935 | 1.23381139 |
| RNU6-625P|ENSG00000238658 | 0.292229 | 0.782254 | 0.495314859 | 1.235420171 |
| RNU6-652P|ENSG00000202358 | 0.29354 | 0.78375 | 0.497415004 | 1.234912899 |
| RNU2-35P|ENSG00000252255 | 0.294443 | 0.774774 | 0.480833162 | 1.24840508 |
| RNU6-64P|ENSG00000252928 | 0.295769 | 0.7828 | 0.494641031 | 1.23882786 |
| RNU6-30P|ENSG00000207291 | 0.295909 | 0.787154 | 0.502525841 | 1.232992871 |
| RNU6-595P|ENSG00000200814 | 0.296062 | 1.287466 | 0.801497923 | 2.06809007 |
| RNU6-101P|ENSG00000222255 | 0.296803 | 0.782016 | 0.492731841 | 1.24113849 |
| RNU6-42P|ENSG00000206892 | 0.298728 | 0.788411 | 0.503522157 | 1.234488087 |
| RNU6-1158P|ENSG00000212469 | 0.300222 | 1.271451 | 0.807182496 | 2.002753152 |
| RNU6-190P|ENSG00000206881 | 0.301462 | 0.78221 | 0.490884752 | 1.246427958 |
| RNU6-908P|ENSG00000222092 | 0.301982 | 0.789231 | 0.503514949 | 1.237075761 |
| RNU6-1266P|ENSG00000199664 | 0.305769 | 1.272537 | 0.802334768 | 2.01829866 |
| RNU6-988P|ENSG00000253437 | 0.306902 | 1.262934 | 0.807061748 | 1.976307931 |
| RNU6-770P|ENSG00000200799 | 0.309827 | 0.791514 | 0.504074986 | 1.242859469 |
| RNU6-882P|ENSG00000212327 | 0.311139 | 0.791691 | 0.503787356 | 1.244123987 |
| RNU6-944P|ENSG00000200893 | 0.319035 | 0.792893 | 0.502311376 | 1.251571704 |
| RNU6-881P|ENSG00000252622 | 0.319298 | 0.796711 | 0.50939864 | 1.246074108 |
| RNU6-731P|ENSG00000253022 | 0.322899 | 0.796804 | 0.507877233 | 1.250099697 |
| RNU7-133P|ENSG00000238987 | 0.323815 | 0.79399 | 0.502110618 | 1.255541167 |
| RNU6-282P|ENSG00000202227 | 0.324885 | 1.26546 | 0.791924457 | 2.022147553 |
| RNU6-176P|ENSG00000253005 | 0.325257 | 1.271897 | 0.787662739 | 2.053824217 |
| RNU6-937P|ENSG00000207053 | 0.326277 | 1.263475 | 0.792093769 | 2.015378937 |
| RNU6-1013P|ENSG00000206907 | 0.32785 | 0.799108 | 0.509932357 | 1.252272201 |
| RNU6-831P|ENSG00000207278 | 0.331155 | 1.249553 | 0.797285818 | 1.958372541 |
| RNU4-38P|ENSG00000201342 | 0.331411 | 1.259686 | 0.790570524 | 2.007167622 |
| RNU7-175P|ENSG00000251724 | 0.331908 | 1.259266 | 0.790459902 | 2.006111366 |
| RNU6-50P|ENSG00000199226 | 0.332739 | 1.250092 | 0.79575926 | 1.963824022 |
| RNU6-1048P|ENSG00000200924 | 0.334807 | 1.252347 | 0.792743953 | 1.97841112 |
| RNU6-758P|ENSG00000252101 | 0.335411 | 1.249175 | 0.794373076 | 1.964364437 |
| RNU6-1188P|ENSG00000200665 | 0.335508 | 0.801994 | 0.511858848 | 1.256586697 |
| RNU6-1285P|ENSG00000200350 | 0.33613 | 1.246875 | 0.795357091 | 1.95471588 |
| RNU7-59P|ENSG00000238880 | 0.336427 | 1.267631 | 0.781615055 | 2.055856606 |
| RNU6-1152P|ENSG00000207306 | 0.337094 | 0.784812 | 0.478518113 | 1.287159891 |
| RNU6-640P|ENSG00000200563 | 0.338748 | 0.802967 | 0.512203276 | 1.258788693 |
| RNU6-672P|ENSG00000206638 | 0.339386 | 0.801006 | 0.508093463 | 1.262779375 |
| RNU6-1057P|ENSG00000252971 | 0.339955 | 1.2504 | 0.79016545 | 1.978698432 |
| RNU6-786P|ENSG00000252658 | 0.343923 | 0.801001 | 0.505906314 | 1.26822562 |
| RNU6-142P|ENSG00000206746 | 0.347839 | 0.797264 | 0.496788791 | 1.2794781 |
| RNU7-75P|ENSG00000251880 | 0.348202 | 0.805431 | 0.51249535 | 1.265803804 |
| RNU6-638P|ENSG00000252391 | 0.34984 | 0.799033 | 0.499226015 | 1.278887213 |
| RNU6-396P|ENSG00000202239 | 0.350706 | 1.238897 | 0.790025252 | 1.942807428 |
| U4|ENSG00000274197 | 0.353977 | 0.806937 | 0.51268319 | 1.270076927 |
| RNU6ATAC|ENSG00000221676 | 0.35425 | 1.237922 | 0.788096398 | 1.944496552 |
| RNU4-80P|ENSG00000200070 | 0.355603 | 1.242447 | 0.783879778 | 1.969276111 |
| RNU6-25P|ENSG00000206600 | 0.359787 | 0.811722 | 0.519418159 | 1.268519113 |
| RNU6-930P|ENSG00000212240 | 0.364824 | 0.810338 | 0.514196593 | 1.277037681 |
| RNU6-106P|ENSG00000207134 | 0.366095 | 1.233027 | 0.782877802 | 1.942008809 |
| RNU6-151P|ENSG00000201028 | 0.367143 | 0.814332 | 0.521130949 | 1.272495887 |
| RNU5A-5P|ENSG00000222986 | 0.370854 | 0.809138 | 0.508830908 | 1.286684419 |
| RNU6-1170P|ENSG00000251783 | 0.371937 | 1.230149 | 0.780700513 | 1.938345446 |
| RNU6ATAC16P|ENSG00000221518 | 0.372546 | 1.233853 | 0.777443616 | 1.95820453 |
| RNU6-548P|ENSG00000252892 | 0.372813 | 0.816241 | 0.522262844 | 1.275696656 |
| RNU6-425P|ENSG00000206604 | 0.373253 | 1.23511 | 0.775962616 | 1.965939697 |
| RNU7-60P|ENSG00000251747 | 0.373374 | 0.814637 | 0.518712792 | 1.279385847 |
| RNU6-623P|ENSG00000200013 | 0.374013 | 0.814761 | 0.518652515 | 1.279924652 |
| RNU1-94P|ENSG00000199497 | 0.374065 | 1.228807 | 0.780144557 | 1.935494853 |
| RNU1-106P|ENSG00000207110 | 0.376651 | 1.233146 | 0.774890485 | 1.962405647 |
| RNU6-19P|ENSG00000207449 | 0.379047 | 0.814965 | 0.516591795 | 1.285674089 |
| RNU2-33P|ENSG00000222276 | 0.384385 | 1.224982 | 0.775448403 | 1.935112816 |
| RNU6-262P|ENSG00000222249 | 0.385603 | 1.226699 | 0.773173904 | 1.946252205 |
| RNU6-313P|ENSG00000252126 | 0.386947 | 1.223984 | 0.774331915 | 1.934747753 |
| RNU1-2|ENSG00000207005 | 0.389416 | 1.220582 | 0.775235307 | 1.921764709 |
| RNU1-103P|ENSG00000252311 | 0.390768 | 1.220604 | 0.774213025 | 1.924372993 |
| RNU6-792P|ENSG00000200360 | 0.392807 | 1.217543 | 0.775207196 | 1.912278186 |
| RNU6-135P|ENSG00000252578 | 0.395882 | 0.823703 | 0.526407104 | 1.288899885 |
| RNU6-315P|ENSG00000199536 | 0.398779 | 1.215678 | 0.772332668 | 1.913519266 |
| RNU2-64P|ENSG00000223247 | 0.398989 | 0.819358 | 0.515707257 | 1.301799261 |
| RNU2-42P|ENSG00000222629 | 0.398989 | 1.214862 | 0.772863039 | 1.909639 |
| RNU1-132P|ENSG00000201170 | 0.401439 | 0.821577 | 0.519126188 | 1.300239407 |
| RNU6-268P|ENSG00000201044 | 0.402028 | 0.826177 | 0.52859179 | 1.2912966 |
| U1|ENSG00000273727 | 0.402709 | 1.217362 | 0.767974478 | 1.92971374 |
| RNU2-70P|ENSG00000222650 | 0.404628 | 0.824702 | 0.524134134 | 1.297630776 |
| RNU6-1085P|ENSG00000200446 | 0.406684 | 1.212637 | 0.769037901 | 1.912113417 |
| RNU6-1301P|ENSG00000199594 | 0.407784 | 1.216346 | 0.765013793 | 1.933949372 |
| RNU7-11P|ENSG00000252796 | 0.413304 | 0.823507 | 0.517159737 | 1.311322592 |
| RNU6-678P|ENSG00000252641 | 0.417694 | 1.204504 | 0.768031922 | 1.88902222 |
| U4|ENSG00000273744 | 0.418204 | 0.826695 | 0.521473347 | 1.310565928 |
| RNU2-18P|ENSG00000223156 | 0.418533 | 1.212562 | 0.760162657 | 1.934199957 |
| RNU6-308P|ENSG00000212259 | 0.421096 | 0.830821 | 0.528946744 | 1.304976516 |
| RNU7-41P|ENSG00000251726 | 0.421101 | 1.202363 | 0.767452885 | 1.883734464 |
| RNU6-1019P|ENSG00000201294 | 0.427764 | 0.834009 | 0.532528637 | 1.306165202 |
| RNU6-705P|ENSG00000222533 | 0.428203 | 1.209018 | 0.755972218 | 1.933569464 |
| RNU6-48P|ENSG00000206888 | 0.43108 | 0.835351 | 0.533795017 | 1.307263768 |
| RNU6-945P|ENSG00000206674 | 0.431164 | 1.201887 | 0.7603616 | 1.899796325 |
| RNU4-27P|ENSG00000222821 | 0.432687 | 1.205392 | 0.755904673 | 1.922158953 |
| RNU6-907P|ENSG00000212226 | 0.438694 | 1.193908 | 0.762363298 | 1.869733788 |
| RNU6-760P|ENSG00000251887 | 0.445304 | 1.190175 | 0.761119971 | 1.861094045 |
| RNU6-71P|ENSG00000207203 | 0.445515 | 0.837975 | 0.532118664 | 1.319635484 |
| RNU1-22P|ENSG00000200204 | 0.44829 | 0.839366 | 0.533804944 | 1.319837941 |
| RNU6-32P|ENSG00000206675 | 0.449384 | 0.837495 | 0.528963843 | 1.325983212 |
| RNU6-897P|ENSG00000252944 | 0.452019 | 0.840673 | 0.534803778 | 1.321478153 |
| U1|ENSG00000277678 | 0.452076 | 1.190382 | 0.75580816 | 1.874826856 |
| RNU6-403P|ENSG00000200033 | 0.452313 | 1.200486 | 0.745428722 | 1.933338621 |
| RNU1-93P|ENSG00000201574 | 0.456548 | 0.842378 | 0.536302215 | 1.323137053 |
| RNU6-236P|ENSG00000200756 | 0.45754 | 1.187478 | 0.754648315 | 1.868558022 |
| RNU1-124P|ENSG00000200731 | 0.458149 | 0.84508 | 0.541720379 | 1.318319518 |
| RNU6-821P|ENSG00000212297 | 0.458595 | 1.186083 | 0.755304257 | 1.862550698 |
| U7|ENSG00000275504 | 0.459551 | 0.845459 | 0.541854149 | 1.319176808 |
| RNU6-1256P|ENSG00000252348 | 0.460291 | 0.842365 | 0.534250232 | 1.328177849 |
| RNU4-29P|ENSG00000251752 | 0.462006 | 1.188407 | 0.750258342 | 1.882432302 |
| RNU4-34P|ENSG00000222363 | 0.462093 | 0.842488 | 0.533556769 | 1.330292729 |
| RNU6-1284P|ENSG00000200571 | 0.462199 | 0.845763 | 0.541140006 | 1.321866714 |
| RNU6-537P|ENSG00000253086 | 0.465823 | 1.182395 | 0.75372663 | 1.854862308 |
| RNU4-36P|ENSG00000201164 | 0.46629 | 1.189762 | 0.745503319 | 1.898762063 |
| RNU6-606P|ENSG00000207452 | 0.467687 | 0.847279 | 0.541735557 | 1.325151322 |
| RNU6-1300P|ENSG00000212348 | 0.468525 | 1.196656 | 0.736465151 | 1.944405202 |
| RNU6-388P|ENSG00000252821 | 0.468674 | 1.179947 | 0.754211204 | 1.845999902 |
| RNU1-91P|ENSG00000201616 | 0.469029 | 1.182294 | 0.751387466 | 1.860318271 |
| RNU6-854P|ENSG00000200906 | 0.471675 | 1.177881 | 0.754198697 | 1.839573297 |
| RNU7-61P|ENSG00000251706 | 0.473338 | 1.196466 | 0.732777401 | 1.953569277 |
| RNU6-387P|ENSG00000223263 | 0.476561 | 1.183218 | 0.744545647 | 1.88034722 |
| RNU1-122P|ENSG00000202408 | 0.478501 | 0.850154 | 0.542652133 | 1.33190544 |
| RNU1-72P|ENSG00000199846 | 0.47892 | 1.17742 | 0.749173842 | 1.850461512 |
| RNU6-1231P|ENSG00000252994 | 0.481274 | 0.849129 | 0.538648659 | 1.33857311 |
| RNU6-977P|ENSG00000252220 | 0.481624 | 0.844088 | 0.526452944 | 1.353368879 |
| RNU6-510P|ENSG00000212541 | 0.481677 | 1.174958 | 0.749839934 | 1.841095629 |
| RNU7-26P|ENSG00000238610 | 0.481885 | 0.850964 | 0.54272378 | 1.334268033 |
| RNU7-171P|ENSG00000238406 | 0.482252 | 1.190442 | 0.732047397 | 1.935873711 |
| RNU6-853P|ENSG00000201176 | 0.48276 | 0.849497 | 0.538700859 | 1.339603427 |
| RNU6-344P|ENSG00000252444 | 0.483678 | 0.85259 | 0.545666454 | 1.33215041 |
| RNU6-433P|ENSG00000200086 | 0.484592 | 0.848863 | 0.53614577 | 1.343977556 |
| RNU6ATAC36P|ENSG00000252684 | 0.485593 | 1.177292 | 0.744137768 | 1.862581586 |
| RNU6-1203P|ENSG00000222635 | 0.48598 | 1.175813 | 0.74553305 | 1.854425676 |
| RNU6-828P|ENSG00000201746 | 0.487787 | 0.852384 | 0.542865331 | 1.338378402 |
| RNU6-100P|ENSG00000252414 | 0.489212 | 1.172991 | 0.746287738 | 1.84366805 |
| RNU6-618P|ENSG00000200388 | 0.492843 | 0.853696 | 0.543214079 | 1.341637944 |
| RNU6-1038P|ENSG00000252325 | 0.493303 | 0.851251 | 0.536973869 | 1.349466384 |
| RNU6-181P|ENSG00000200095 | 0.495132 | 0.856238 | 0.548187818 | 1.337394554 |
| RNU6-88P|ENSG00000272507 | 0.496203 | 0.854135 | 0.542379561 | 1.345084959 |
| RNU6-1181P|ENSG00000206918 | 0.496716 | 0.854165 | 0.542160727 | 1.345723324 |
| RNU2-72P|ENSG00000223107 | 0.498592 | 0.857264 | 0.54877352 | 1.339171524 |
| RNU6-379P|ENSG00000200683 | 0.50084 | 0.856467 | 0.545499985 | 1.344703954 |
| RNU6-216P|ENSG00000200152 | 0.501353 | 0.855358 | 0.54244635 | 1.348774575 |
| RNU2-30P|ENSG00000252018 | 0.505432 | 1.163606 | 0.744966053 | 1.817505473 |
| RNU6-258P|ENSG00000212186 | 0.505506 | 1.163928 | 0.744509177 | 1.819624837 |
| RNU6-182P|ENSG00000252779 | 0.507748 | 0.858741 | 0.547208458 | 1.347632561 |
| RNU6-1206P|ENSG00000252148 | 0.509463 | 0.860457 | 0.550557675 | 1.344794214 |
| RNU4-89P|ENSG00000272359 | 0.510212 | 0.858836 | 0.546012636 | 1.350883208 |
| RNU6-87P|ENSG00000272028 | 0.510604 | 0.860941 | 0.551124231 | 1.344921089 |
| RNU6-196P|ENSG00000207042 | 0.512527 | 1.166734 | 0.735432839 | 1.850974827 |
| RNU6-1238P|ENSG00000253024 | 0.514611 | 0.853289 | 0.529495796 | 1.375086132 |
| RNU6-1255P|ENSG00000200304 | 0.515371 | 1.173705 | 0.724396213 | 1.901699279 |
| RNU6-875P|ENSG00000252297 | 0.522747 | 1.159418 | 0.736607976 | 1.824920083 |
| RNU6-824P|ENSG00000200594 | 0.524702 | 1.158274 | 0.736544784 | 1.821475834 |
| RNU6-264P|ENSG00000252943 | 0.524978 | 0.859191 | 0.53811538 | 1.371842701 |
| RNU1-88P|ENSG00000238554 | 0.525923 | 1.155296 | 0.739529156 | 1.804810319 |
| RNU7-71P|ENSG00000251868 | 0.529209 | 0.865939 | 0.553047918 | 1.355849064 |
| RNU6-323P|ENSG00000253739 | 0.530551 | 0.865842 | 0.551984074 | 1.358158285 |
| RNU6-80P|ENSG00000206922 | 0.531997 | 1.153676 | 0.736852525 | 1.806287215 |
| RNU7-96P|ENSG00000238719 | 0.534072 | 0.865427 | 0.548752046 | 1.364849296 |
| RNU6-354P|ENSG00000206589 | 0.534442 | 0.867501 | 0.554007561 | 1.358387995 |
| RNU6-310P|ENSG00000207385 | 0.53468 | 0.867866 | 0.554833482 | 1.357509113 |
| RNU6-838P|ENSG00000200269 | 0.535529 | 0.86647 | 0.550612601 | 1.363516612 |
| RNU2-6P|ENSG00000223336 | 0.535961 | 0.867857 | 0.554029483 | 1.359450863 |
| RNU7-63P|ENSG00000238417 | 0.536394 | 1.153774 | 0.733153392 | 1.815709675 |
| RNU7-73P|ENSG00000239105 | 0.537772 | 1.152137 | 0.734296548 | 1.807744646 |
| RNU7-79P|ENSG00000251891 | 0.541824 | 1.156828 | 0.724437967 | 1.847296683 |
| RNU6-375P|ENSG00000252859 | 0.545428 | 1.149847 | 0.73125385 | 1.808056048 |
| RNU6-969P|ENSG00000206627 | 0.549276 | 1.148774 | 0.729612636 | 1.808744197 |
| RNU6-874P|ENSG00000199260 | 0.549812 | 1.15293 | 0.723196067 | 1.838017391 |
| RNU7-160P|ENSG00000252469 | 0.55071 | 0.871296 | 0.554152097 | 1.369943256 |
| RNU6-1223P|ENSG00000252933 | 0.556097 | 1.145838 | 0.728226688 | 1.802934014 |
| RNU6-285P|ENSG00000222488 | 0.556183 | 0.872468 | 0.553892306 | 1.374273603 |
| RNU4-59P|ENSG00000201317 | 0.557676 | 0.864173 | 0.530468871 | 1.407801786 |
| RNU1-61P|ENSG00000251916 | 0.56353 | 0.874116 | 0.553729306 | 1.379876794 |
| RNU6-195P|ENSG00000223284 | 0.563724 | 0.874398 | 0.554389193 | 1.379124006 |
| RNU6-986P|ENSG00000200003 | 0.566763 | 0.873918 | 0.551070928 | 1.385904768 |
| RNU6-787P|ENSG00000206759 | 0.568911 | 1.139298 | 0.727394784 | 1.7844516 |
| RNU2-59P|ENSG00000222414 | 0.569252 | 0.877965 | 0.560833215 | 1.374425342 |
| RNU6-435P|ENSG00000206766 | 0.570355 | 0.875902 | 0.554276452 | 1.384156032 |
| RNU6-1011P|ENSG00000207399 | 0.570483 | 1.139877 | 0.725158779 | 1.791772762 |
| RNU6ATAC40P|ENSG00000221507 | 0.570975 | 0.876164 | 0.554605187 | 1.384161068 |
| RNU2-50P|ENSG00000222426 | 0.571605 | 1.139351 | 0.72502742 | 1.790442918 |
| RNU6-898P|ENSG00000202497 | 0.573714 | 1.137409 | 0.726294706 | 1.781231449 |
| RNU6-670P|ENSG00000201182 | 0.573977 | 1.136788 | 0.727057019 | 1.777422243 |
| RNU7-200P|ENSG00000251914 | 0.576061 | 1.140314 | 0.719677016 | 1.806806309 |
| RNU6-583P|ENSG00000251821 | 0.576369 | 0.879758 | 0.561284093 | 1.37893364 |
| RNU6-1262P|ENSG00000252026 | 0.576634 | 1.136906 | 0.724604306 | 1.783809802 |
| RNU6-1267P|ENSG00000222679 | 0.578222 | 0.880436 | 0.562011191 | 1.379273855 |
| RNU6ATAC24P|ENSG00000252620 | 0.581215 | 0.880383 | 0.559861359 | 1.384402972 |
| RNU6-1147P|ENSG00000200250 | 0.58138 | 0.878699 | 0.554890062 | 1.391467082 |
| RNU6-1|ENSG00000206625 | 0.58162 | 1.135018 | 0.72336736 | 1.780930081 |
| RNU6-312P|ENSG00000201499 | 0.583324 | 0.879222 | 0.55508438 | 1.39263898 |
| RNU6-288P|ENSG00000200560 | 0.584433 | 0.882096 | 0.562682869 | 1.382826185 |
| RNU6-298P|ENSG00000212190 | 0.585011 | 0.878417 | 0.5516128 | 1.398837073 |
| RNU6-358P|ENSG00000252373 | 0.586085 | 1.132105 | 0.72430964 | 1.76949503 |
| RNU6-89P|ENSG00000272262 | 0.591847 | 0.883048 | 0.560436433 | 1.391367251 |
| RNU2-62P|ENSG00000222800 | 0.592853 | 0.8842 | 0.563160529 | 1.388254707 |
| RNU6-922P|ENSG00000252562 | 0.593404 | 1.129248 | 0.722772345 | 1.764318396 |
| RNU1-13P|ENSG00000238825 | 0.593621 | 1.133298 | 0.715700712 | 1.794554583 |
| RNU6-1333P|ENSG00000251971 | 0.594356 | 1.1325 | 0.71639666 | 1.790287242 |
| RNU6-213P|ENSG00000206778 | 0.594452 | 0.879281 | 0.547567925 | 1.411942422 |
| RNU6-314P|ENSG00000252383 | 0.596021 | 1.13406 | 0.712261096 | 1.805647521 |
| RNU6-767P|ENSG00000206859 | 0.596052 | 1.131625 | 0.716375058 | 1.78757588 |
| RNU6-597P|ENSG00000202490 | 0.597234 | 1.13257 | 0.713702808 | 1.797268606 |
| RNU6-1283P|ENSG00000202184 | 0.598003 | 1.131679 | 0.714537296 | 1.792346526 |
| RNU4-72P|ENSG00000222686 | 0.598052 | 1.127984 | 0.720872629 | 1.765010734 |
| RNU6-653P|ENSG00000199944 | 0.598779 | 1.136347 | 0.705877975 | 1.829331243 |
| RNVU1-3|ENSG00000201183 | 0.600204 | 0.881183 | 0.54907642 | 1.414161389 |
| RNU1-120P|ENSG00000199879 | 0.602159 | 1.137223 | 0.70125963 | 1.844217588 |
| RNU5A-6P|ENSG00000206863 | 0.603224 | 1.126766 | 0.718417827 | 1.767219759 |
| RNU2-27P|ENSG00000251994 | 0.603847 | 0.887709 | 0.566072125 | 1.392096705 |
| RNU6-476P|ENSG00000207457 | 0.60446 | 0.887101 | 0.563789119 | 1.395819697 |
| RNU6-1310P|ENSG00000206880 | 0.605467 | 0.887744 | 0.565035326 | 1.394760692 |
| RNU6-107P|ENSG00000252452 | 0.612852 | 0.890019 | 0.566765683 | 1.397640509 |
| RNU6-975P|ENSG00000207023 | 0.612944 | 0.891323 | 0.570756144 | 1.391936185 |
| RNU7-77P|ENSG00000253054 | 0.613105 | 1.125067 | 0.712528988 | 1.776455681 |
| RNU6-150P|ENSG00000252046 | 0.613579 | 1.129857 | 0.703431092 | 1.814784806 |
| RNU6-942P|ENSG00000199872 | 0.617227 | 0.891444 | 0.568045337 | 1.398960843 |
| RNU6-859P|ENSG00000199598 | 0.61753 | 1.124837 | 0.70886321 | 1.784912959 |
| RNU5E-1|ENSG00000199347 | 0.619239 | 1.120784 | 0.714825617 | 1.757290772 |
| RNU6-31P|ENSG00000207116 | 0.620338 | 0.891777 | 0.566816763 | 1.403039934 |
| RNU6-1156P|ENSG00000222297 | 0.621285 | 0.892174 | 0.567371505 | 1.402914534 |
| RNU6-577P|ENSG00000252756 | 0.624191 | 1.119061 | 0.713526779 | 1.755081599 |
| RNU5A-1|ENSG00000199568 | 0.625433 | 1.121358 | 0.707982512 | 1.776095513 |
| RNU7-3P|ENSG00000252244 | 0.626399 | 1.119699 | 0.710246071 | 1.765200664 |
| RNU6-742P|ENSG00000202159 | 0.627327 | 1.119754 | 0.709270527 | 1.767799437 |
| RNU5E-4P|ENSG00000201801 | 0.629817 | 1.115701 | 0.714819825 | 1.741403256 |
| RNU6-1201P|ENSG00000206954 | 0.630276 | 0.894793 | 0.569039694 | 1.40702903 |
| RNU4-60P|ENSG00000253048 | 0.63069 | 1.117071 | 0.711316596 | 1.754279243 |
| RNU6-179P|ENSG00000200220 | 0.630737 | 0.893548 | 0.564685255 | 1.413935511 |
| RNU6-1099P|ENSG00000200403 | 0.632763 | 1.115986 | 0.711504132 | 1.750409905 |
| RNU5F-1|ENSG00000199377 | 0.641249 | 1.112474 | 0.710523102 | 1.741813045 |
| RNU7-8P|ENSG00000251767 | 0.642419 | 1.114638 | 0.704975881 | 1.762356612 |
| U7|ENSG00000273629 | 0.643741 | 0.897952 | 0.569035857 | 1.416989006 |
| RNU7-110P|ENSG00000252700 | 0.646895 | 0.899117 | 0.57044329 | 1.417164149 |
| RNU6-128P|ENSG00000206703 | 0.647232 | 0.899149 | 0.570283269 | 1.417661534 |
| RNU4-1|ENSG00000200795 | 0.648255 | 0.897581 | 0.564233675 | 1.427867964 |
| RNVU1-19|ENSG00000275538 | 0.64928 | 0.901712 | 0.577320868 | 1.408376415 |
| RNU6-789P|ENSG00000252914 | 0.649897 | 0.899214 | 0.568385164 | 1.422600731 |
| RNU4-4P|ENSG00000201458 | 0.651412 | 0.901443 | 0.574718577 | 1.413907758 |
| RNU6-419P|ENSG00000252839 | 0.651775 | 1.112221 | 0.700785275 | 1.765214282 |
| RNU6-1089P|ENSG00000252282 | 0.652642 | 0.900055 | 0.569026509 | 1.423659267 |
| RNU6-33P|ENSG00000207524 | 0.66524 | 1.110697 | 0.690335253 | 1.787027266 |
| RNU2-25P|ENSG00000222973 | 0.666232 | 1.10436 | 0.703418434 | 1.73383364 |
| RNU6-1115P|ENSG00000199360 | 0.668115 | 0.906985 | 0.580455734 | 1.417199126 |
| RNU6-494P|ENSG00000253063 | 0.669855 | 1.106133 | 0.695697533 | 1.758709921 |
| U4atac|ENSG00000275908 | 0.670849 | 1.10895 | 0.688310902 | 1.786647592 |
| RNU6-689P|ENSG00000206924 | 0.671445 | 0.907817 | 0.580656711 | 1.419309947 |
| RNU6-1005P|ENSG00000207248 | 0.67263 | 0.903956 | 0.565896669 | 1.443968894 |
| RNU6-377P|ENSG00000251774 | 0.676439 | 0.905799 | 0.569158147 | 1.44155265 |
| RNU6-762P|ENSG00000252916 | 0.676996 | 0.90841 | 0.578090754 | 1.42747286 |
| RNU4-82P|ENSG00000199313 | 0.678232 | 1.101034 | 0.69875537 | 1.734907447 |
| RNU6-720P|ENSG00000252172 | 0.678445 | 1.099319 | 0.702603637 | 1.720034751 |
| RNU6-686P|ENSG00000201709 | 0.679685 | 0.910385 | 0.583002996 | 1.421607053 |
| RNU6-360P|ENSG00000212246 | 0.681688 | 0.910619 | 0.58213995 | 1.424447231 |
| RNU6-422P|ENSG00000207362 | 0.68315 | 1.100611 | 0.69456682 | 1.744030144 |
| RNU2-31P|ENSG00000252763 | 0.685441 | 1.099123 | 0.695713732 | 1.736448614 |
| RNU6ATAC9P|ENSG00000252019 | 0.687344 | 1.096113 | 0.701070575 | 1.713756642 |
| RNU6-1321P|ENSG00000212207 | 0.688525 | 1.096118 | 0.699805374 | 1.716870943 |
| RNU6-446P|ENSG00000199697 | 0.688537 | 0.900251 | 0.538542251 | 1.50490055 |
| RNU6-409P|ENSG00000206841 | 0.689823 | 0.910504 | 0.574531224 | 1.442944959 |
| RNU4-61P|ENSG00000223175 | 0.691274 | 0.913284 | 0.58368816 | 1.42899463 |
| RNU6-2|ENSG00000207357 | 0.692762 | 1.094703 | 0.6988198 | 1.714853667 |
| RNU6-805P|ENSG00000202513 | 0.694099 | 1.095721 | 0.694798241 | 1.727990516 |
| RNU7-90P|ENSG00000238731 | 0.695429 | 1.095721 | 0.69334428 | 1.731614347 |
| RNU7-49P|ENSG00000251991 | 0.695784 | 0.914977 | 0.586111301 | 1.428368322 |
| RNU6-469P|ENSG00000252062 | 0.703635 | 0.916178 | 0.583572521 | 1.438350821 |
| RNU6-817P|ENSG00000212385 | 0.703735 | 1.091678 | 0.694635409 | 1.715664369 |
| RNU6-226P|ENSG00000200648 | 0.705228 | 1.099244 | 0.673240904 | 1.794808144 |
| RNU6-37P|ENSG00000199562 | 0.705776 | 1.093156 | 0.688439523 | 1.735793898 |
| RNU1-57P|ENSG00000207220 | 0.706247 | 1.091225 | 0.693031069 | 1.718207186 |
| RNU6-993P|ENSG00000252962 | 0.706568 | 0.915529 | 0.578269712 | 1.449484995 |
| RNU6-94P|ENSG00000271819 | 0.70882 | 0.917671 | 0.584592218 | 1.44052417 |
| RNU6ATAC26P|ENSG00000210841 | 0.709571 | 1.08884 | 0.695723043 | 1.704088263 |
| RNU4-2|ENSG00000202538 | 0.710578 | 1.088195 | 0.696352183 | 1.700531638 |
| RNU6-130P|ENSG00000223044 | 0.710863 | 0.918344 | 0.585335678 | 1.440805441 |
| RNU6-1189P|ENSG00000206685 | 0.711164 | 1.089252 | 0.692799504 | 1.712573143 |
| RNU6-539P|ENSG00000252474 | 0.712343 | 1.092692 | 0.682111789 | 1.75040952 |
| RNU6-125P|ENSG00000207234 | 0.712465 | 0.919349 | 0.587848728 | 1.437788626 |
| RNU6-724P|ENSG00000212260 | 0.714979 | 1.086701 | 0.695506983 | 1.697925896 |
| RNU6-171P|ENSG00000207082 | 0.716309 | 0.919637 | 0.585305466 | 1.444940939 |
| RNU6-28P|ENSG00000199248 | 0.719336 | 1.086015 | 0.692435357 | 1.703303815 |
| RNU2-28P|ENSG00000222389 | 0.720876 | 1.085353 | 0.69253141 | 1.700993995 |
| RNU6-57P|ENSG00000223280 | 0.722072 | 0.921501 | 0.587294425 | 1.445890184 |
| RNU6-1254P|ENSG00000212568 | 0.723901 | 0.920759 | 0.582388992 | 1.455721751 |
| RNU6-1297P|ENSG00000207144 | 0.72759 | 1.0885 | 0.675470354 | 1.754083896 |
| RNU5E-10P|ENSG00000200376 | 0.728685 | 0.922441 | 0.584562984 | 1.455614271 |
| RNU6-547P|ENSG00000252082 | 0.730634 | 1.082447 | 0.689498057 | 1.699339483 |
| RNU6-1289P|ENSG00000207160 | 0.734389 | 0.924608 | 0.58790688 | 1.45414248 |
| RNU6-402P|ENSG00000222610 | 0.736084 | 0.925549 | 0.590209906 | 1.45141705 |
| RNU6-615P|ENSG00000251874 | 0.737797 | 0.924932 | 0.585725871 | 1.460578254 |
| RNU4-25P|ENSG00000222501 | 0.739921 | 0.925697 | 0.586805542 | 1.460306347 |
| RNU6-212P|ENSG00000199512 | 0.74017 | 1.085685 | 0.667864952 | 1.764897005 |
| RNU6ATAC18P|ENSG00000221340 | 0.742681 | 1.078422 | 0.68713468 | 1.692527149 |
| RNU4-85P|ENSG00000201545 | 0.743938 | 1.078641 | 0.684863976 | 1.698829014 |
| RNU7-18P|ENSG00000252174 | 0.747233 | 0.929068 | 0.593925776 | 1.453324965 |
| RNU7-10P|ENSG00000271841 | 0.748459 | 0.928983 | 0.592203005 | 1.457287417 |
| RNU6-1245P|ENSG00000223062 | 0.748546 | 1.082735 | 0.665955274 | 1.760350837 |
| RNU6-1257P|ENSG00000207355 | 0.74856 | 0.927455 | 0.58519713 | 1.46988599 |
| RNU6-733P|ENSG00000222691 | 0.749061 | 1.079232 | 0.676416253 | 1.721928991 |
| RNU5D-1|ENSG00000200169 | 0.749584 | 1.0756 | 0.687543613 | 1.68267942 |
| RNU6-808P|ENSG00000212535 | 0.751294 | 0.930346 | 0.595300791 | 1.453959191 |
| RNU6-863P|ENSG00000251798 | 0.751543 | 1.075248 | 0.686202015 | 1.684867157 |
| RNU6-1212P|ENSG00000252832 | 0.751974 | 0.929007 | 0.588415217 | 1.46674434 |
| RNU6-541P|ENSG00000207440 | 0.75277 | 0.929332 | 0.58900685 | 1.466295691 |
| RNU6-589P|ENSG00000252294 | 0.76001 | 0.932645 | 0.596220599 | 1.458899655 |
| RNU7-28P|ENSG00000252568 | 0.762423 | 0.930586 | 0.583699583 | 1.483623502 |
| RNU6-97P|ENSG00000200257 | 0.763349 | 1.071126 | 0.684838705 | 1.675301552 |
| RNU6ATAC27P|ENSG00000221216 | 0.764973 | 1.070659 | 0.684316627 | 1.675118356 |
| RNU6-172P|ENSG00000222932 | 0.766028 | 1.071761 | 0.678982306 | 1.691755594 |
| RNU4-26P|ENSG00000222202 | 0.766708 | 1.071212 | 0.680003312 | 1.687485366 |
| RNU7-13P|ENSG00000252222 | 0.769897 | 0.935267 | 0.597297402 | 1.4644717 |
| RNU6-558P|ENSG00000201382 | 0.772499 | 0.935572 | 0.595659831 | 1.469453483 |
| RNU6-700P|ENSG00000212345 | 0.778205 | 1.066984 | 0.679544297 | 1.675320027 |
| RNU6-673P|ENSG00000201954 | 0.779205 | 0.932367 | 0.571439956 | 1.52126039 |
| RNU7-45P|ENSG00000238829 | 0.779504 | 1.066932 | 0.677891 | 1.679242417 |
| RNU4-50P|ENSG00000201231 | 0.779939 | 0.936752 | 0.592341465 | 1.481415272 |
| RNU6-884P|ENSG00000200759 | 0.781924 | 0.937101 | 0.591589419 | 1.484404678 |
| RNU6-233P|ENSG00000222777 | 0.78288 | 0.939075 | 0.600528671 | 1.468477096 |
| RNU6-346P|ENSG00000252097 | 0.788195 | 1.063531 | 0.678571664 | 1.6668801 |
| RNU6-177P|ENSG00000223189 | 0.790126 | 1.062819 | 0.678590502 | 1.6646035 |
| RNU6-1153P|ENSG00000252569 | 0.790371 | 0.940272 | 0.597103483 | 1.480666321 |
| RNU6-959P|ENSG00000207402 | 0.79144 | 0.93999 | 0.594183343 | 1.48705141 |
| RNVU1-7|ENSG00000206585 | 0.791968 | 0.941712 | 0.602702937 | 1.471406904 |
| RNU7-134P|ENSG00000238447 | 0.794627 | 0.93791 | 0.578831508 | 1.519744105 |
| RNU6-697P|ENSG00000200218 | 0.795682 | 0.94107 | 0.594240885 | 1.490327498 |
| RNU2-65P|ENSG00000222094 | 0.798754 | 0.943091 | 0.601089422 | 1.479681486 |
| RNU6-8|ENSG00000202337 | 0.802595 | 1.061409 | 0.665207962 | 1.693589793 |
| RNU6-14P|ENSG00000207360 | 0.803352 | 1.06099 | 0.665793294 | 1.690763744 |
| RNU1-63P|ENSG00000206629 | 0.803719 | 1.058265 | 0.677082847 | 1.654044613 |
| RNU6-602P|ENSG00000223087 | 0.8064 | 1.058228 | 0.672988846 | 1.663989098 |
| RNU6-610P|ENSG00000206991 | 0.807274 | 0.94511 | 0.600474111 | 1.487544511 |
| RNU7-40P|ENSG00000252206 | 0.809467 | 0.937078 | 0.552518987 | 1.589294476 |
| RNU6-302P|ENSG00000202119 | 0.810583 | 0.946899 | 0.606074164 | 1.479387667 |
| RNU6-237P|ENSG00000251794 | 0.811829 | 0.947319 | 0.606729602 | 1.479100364 |
| RNU6-484P|ENSG00000206743 | 0.812607 | 0.947062 | 0.604062106 | 1.484823488 |
| RNU6V|ENSG00000206832 | 0.812812 | 0.947417 | 0.605863725 | 1.481519494 |
| RNU1-74P|ENSG00000206835 | 0.813588 | 0.947358 | 0.604363522 | 1.485011071 |
| RNVU1-6|ENSG00000201558 | 0.814734 | 1.055492 | 0.671844915 | 1.658215235 |
| RNU6-858P|ENSG00000199306 | 0.815359 | 1.057209 | 0.662786683 | 1.686351287 |
| RNU12-2P|ENSG00000201659 | 0.818576 | 0.94836 | 0.602859509 | 1.491868275 |
| RNU6-906P|ENSG00000207431 | 0.818649 | 0.94594 | 0.58821769 | 1.521208482 |
| U1|ENSG00000277918 | 0.821059 | 1.052868 | 0.673744367 | 1.645328777 |
| RNU6-1215P|ENSG00000207170 | 0.825312 | 1.05199 | 0.670730176 | 1.649968769 |
| RNU7-141P|ENSG00000238777 | 0.829517 | 0.950583 | 0.599302018 | 1.507768956 |
| RNU6-335P|ENSG00000201433 | 0.830773 | 1.049695 | 0.672807407 | 1.637704178 |
| RNU4-9P|ENSG00000201821 | 0.830881 | 0.952448 | 0.609070331 | 1.48941415 |
| RNU7-19P|ENSG00000238959 | 0.832076 | 0.951515 | 0.601042251 | 1.506352238 |
| U1|ENSG00000206828 | 0.837378 | 1.048011 | 0.669710577 | 1.640003591 |
| RNU6-1278P|ENSG00000251939 | 0.837952 | 1.048571 | 0.665573711 | 1.651961529 |
| RNU6-44P|ENSG00000206772 | 0.838224 | 1.048344 | 0.666298428 | 1.649449978 |
| RNVU1-15|ENSG00000207205 | 0.838955 | 0.953136 | 0.599960337 | 1.514213124 |
| RNU6-132P|ENSG00000206590 | 0.839273 | 1.047215 | 0.670531993 | 1.635507736 |
| RNU4-87P|ENSG00000200974 | 0.844598 | 0.955425 | 0.605597938 | 1.507332969 |
| RNU7-180P|ENSG00000238374 | 0.844687 | 1.046933 | 0.661658345 | 1.656548337 |
| RNU6-514P|ENSG00000206935 | 0.846731 | 0.956706 | 0.610764379 | 1.49859174 |
| RNU2-63P|ENSG00000222724 | 0.850111 | 1.044516 | 0.664875348 | 1.640928223 |
| RNU6-915P|ENSG00000201813 | 0.851885 | 0.957026 | 0.603502793 | 1.517639527 |
| RNVU1-2|ENSG00000252135 | 0.85376 | 0.956703 | 0.597551136 | 1.531719471 |
| RNU6-790P|ENSG00000207208 | 0.853875 | 0.956805 | 0.598065944 | 1.530726412 |
| RNU6-482P|ENSG00000212370 | 0.855044 | 1.042879 | 0.664677162 | 1.636277028 |
| RNU1-133P|ENSG00000201737 | 0.857705 | 0.959321 | 0.609265513 | 1.510503192 |
| RNU1-16P|ENSG00000202347 | 0.85772 | 0.959298 | 0.609059438 | 1.510940698 |
| RNU6-1294P|ENSG00000251804 | 0.859044 | 1.041888 | 0.662432397 | 1.638703901 |
| RNU7-140P|ENSG00000238364 | 0.866623 | 0.962542 | 0.616490076 | 1.502840508 |
| RNU6-850P|ENSG00000252743 | 0.869253 | 1.040071 | 0.651471369 | 1.660469059 |
| RNU6-1121P|ENSG00000252611 | 0.869973 | 0.962897 | 0.612310207 | 1.514215967 |
| RNU6-840P|ENSG00000253084 | 0.876367 | 0.964438 | 0.611168229 | 1.521905712 |
| RNU6-62P|ENSG00000252335 | 0.877806 | 1.035828 | 0.661309027 | 1.62244922 |
| RNU6-188P|ENSG00000201077 | 0.878946 | 0.964709 | 0.607569976 | 1.53177873 |
| RNU6-1157P|ENSG00000207185 | 0.879204 | 0.965435 | 0.613343149 | 1.519645107 |
| RNU6-504P|ENSG00000252377 | 0.881743 | 0.966349 | 0.615561564 | 1.517039146 |
| RNU6-593P|ENSG00000201586 | 0.882945 | 1.035371 | 0.651840214 | 1.644565465 |
| RNU6-1279P|ENSG00000206644 | 0.882961 | 0.966801 | 0.616769249 | 1.515484251 |
| RNU6-750P|ENSG00000212248 | 0.887095 | 1.032786 | 0.661616577 | 1.612182148 |
| RNU6-399P|ENSG00000202034 | 0.887331 | 1.032908 | 0.659996964 | 1.616519466 |
| RNU7-128P|ENSG00000253056 | 0.8882 | 0.967796 | 0.613177695 | 1.527500728 |
| RNU6-920P|ENSG00000222858 | 0.891349 | 1.032266 | 0.654486705 | 1.628106309 |
| RNU6ATAC29P|ENSG00000221015 | 0.892041 | 1.031631 | 0.657987805 | 1.617450697 |
| RNU6-866P|ENSG00000274607 | 0.893047 | 0.969746 | 0.619669582 | 1.517593491 |
| RNU6-876P|ENSG00000212597 | 0.894496 | 1.032819 | 0.640855717 | 1.664515725 |
| RNU6-934P|ENSG00000202024 | 0.89469 | 1.031029 | 0.655803539 | 1.620945496 |
| RNU6-723P|ENSG00000206700 | 0.897293 | 0.970699 | 0.617984034 | 1.524726026 |
| RNU7-30P|ENSG00000252644 | 0.898578 | 1.029552 | 0.657882945 | 1.611195947 |
| RNU6-647P|ENSG00000201113 | 0.900849 | 1.02881 | 0.658086067 | 1.608377754 |
| RNU6-509P|ENSG00000200389 | 0.902584 | 1.02883 | 0.652656157 | 1.621820514 |
| RNU6-242P|ENSG00000207087 | 0.905073 | 0.973103 | 0.621649129 | 1.523252773 |
| RNU6-1154P|ENSG00000212303 | 0.905357 | 1.027596 | 0.656047927 | 1.609567619 |
| RNU2-37P|ENSG00000222627 | 0.910009 | 1.026826 | 0.648831742 | 1.625031312 |
| RNU6-137P|ENSG00000200550 | 0.910373 | 1.026269 | 0.653412681 | 1.611887801 |
| RNU6-306P|ENSG00000207347 | 0.912569 | 0.974491 | 0.614401382 | 1.545621495 |
| RNU5B-1|ENSG00000200156 | 0.915209 | 1.025105 | 0.649442356 | 1.618065173 |
| RNU6-272P|ENSG00000222960 | 0.917576 | 0.976016 | 0.616287403 | 1.545720055 |
| RNU6-998P|ENSG00000251703 | 0.91835 | 0.976725 | 0.622620319 | 1.532219814 |
| RNU6-769P|ENSG00000207136 | 0.919008 | 1.023657 | 0.652264957 | 1.606515604 |
| U2|ENSG00000278135 | 0.923751 | 1.022161 | 0.652501051 | 1.60124352 |
| RNU6-658P|ENSG00000207336 | 0.924548 | 0.978287 | 0.62110809 | 1.540868942 |
| RNU6-999P|ENSG00000251754 | 0.930371 | 1.020567 | 0.646424435 | 1.611259303 |
| RNU6-437P|ENSG00000238420 | 0.931939 | 0.980445 | 0.623153371 | 1.542593235 |
| RNU6-1190P|ENSG00000222743 | 0.9356 | 1.019696 | 0.635332383 | 1.636591675 |
| U4|ENSG00000276103 | 0.936367 | 1.01859 | 0.648068476 | 1.600949341 |
| RNU4ATAC12P|ENSG00000252269 | 0.939767 | 0.982742 | 0.625655562 | 1.543632078 |
| RNU6-194P|ENSG00000200732 | 0.941031 | 0.982205 | 0.610379157 | 1.580537492 |
| RNVU1-4|ENSG00000277610 | 0.943148 | 0.983667 | 0.625593156 | 1.546694548 |
| RNU6-891P|ENSG00000207007 | 0.943611 | 1.016575 | 0.644632069 | 1.603122263 |
| U1|ENSG00000270722 | 0.945311 | 0.984196 | 0.624316005 | 1.551524145 |
| RNU6-1209P|ENSG00000200525 | 0.947335 | 1.015319 | 0.646686982 | 1.594082178 |
| RNU1-109P|ENSG00000206687 | 0.94841 | 0.985291 | 0.628951031 | 1.543518296 |
| RNU6-711P|ENSG00000253064 | 0.951621 | 0.986005 | 0.62538171 | 1.55457981 |
| RNU6-255P|ENSG00000252766 | 0.952142 | 0.986358 | 0.629818712 | 1.544732307 |
| RNU6ATAC4P|ENSG00000210181 | 0.953526 | 0.986686 | 0.628661126 | 1.548607321 |
| RNU6-43P|ENSG00000207029 | 0.953728 | 0.986662 | 0.626883238 | 1.552922526 |
| RNU6-877P|ENSG00000206595 | 0.954308 | 0.986792 | 0.626179629 | 1.555077149 |
| U1|ENSG00000273516 | 0.955074 | 0.987131 | 0.629025288 | 1.549106778 |
| RNU6-319P|ENSG00000251834 | 0.958745 | 1.011904 | 0.646267 | 1.584407048 |
| RNU6-512P|ENSG00000201308 | 0.963163 | 1.010691 | 0.643604253 | 1.587151176 |
| RNU6-1327P|ENSG00000202172 | 0.964096 | 1.010425 | 0.643272399 | 1.587132305 |
| RNU7-115P|ENSG00000252242 | 0.964476 | 1.010295 | 0.643723738 | 1.585611127 |
| RNU7-170P|ENSG00000239082 | 0.964756 | 1.010221 | 0.643453617 | 1.586045025 |
| RNU4-40P|ENSG00000201221 | 0.965511 | 1.010094 | 0.640694133 | 1.59247527 |
| RNU2-58P|ENSG00000252212 | 0.966157 | 0.99041 | 0.634594258 | 1.545731495 |
| RNU7-169P|ENSG00000238457 | 0.966401 | 0.99021 | 0.626494269 | 1.565083077 |
| RNU1-68P|ENSG00000275229 | 0.967008 | 0.990599 | 0.633142418 | 1.549865491 |
| RNU6-1081P|ENSG00000207267 | 0.970459 | 1.008588 | 0.641441947 | 1.585879974 |
| RNU6-141P|ENSG00000222431 | 0.973387 | 0.992012 | 0.619275712 | 1.589094605 |
| RNU4-30P|ENSG00000222177 | 0.973554 | 1.007642 | 0.642429754 | 1.580473371 |
| RNVU1-18|ENSG00000206737 | 0.974952 | 1.007215 | 0.643037934 | 1.577638611 |
| RNU1-129P|ENSG00000206791 | 0.976011 | 1.007187 | 0.631545081 | 1.606259376 |
| RNU1-136P|ENSG00000206908 | 0.97619 | 1.006829 | 0.643968395 | 1.574151877 |
| RNU7-195P|ENSG00000239151 | 0.977501 | 0.993583 | 0.635187599 | 1.554197978 |
| RNU5A-8P|ENSG00000200972 | 0.980207 | 1.00579 | 0.637446812 | 1.58697582 |
| RNU4ATAC16P|ENSG00000221439 | 0.980333 | 0.994248 | 0.628517403 | 1.572796048 |
| RNU6-757P|ENSG00000222266 | 0.981357 | 1.005586 | 0.630251885 | 1.604442204 |
| RNU7-123P|ENSG00000251720 | 0.988035 | 1.003478 | 0.637414064 | 1.579771596 |
| RNU2-24P|ENSG00000252639 | 0.988504 | 0.996712 | 0.636823812 | 1.559984602 |
| RNU6-249P|ENSG00000199886 | 0.990783 | 0.997327 | 0.633254433 | 1.5707118 |
| RNU6-925P|ENSG00000207359 | 0.994616 | 0.998385 | 0.624245855 | 1.596761423 |
| RNU7-113P|ENSG00000238735 | 0.995999 | 1.001157 | 0.637121609 | 1.573192981 |
| RNU1-108P|ENSG00000199426 | 0.998399 | 0.999543 | 0.63939279 | 1.562554184 |

Table S3.Results of co-expression gene screening of the Ten Prognostic snRNAs.

| ID | ID | R | P value |
| --- | --- | --- | --- |
| U1 | PROSER1 | -0.325 | 0.003498597 |
| U1 | SPG20 | -0.276 | 0.012478151 |
| U1 | STAT3 | -0.254 | 0.020947255 |
| U1 | DGCR2 | -0.25 | 0.022936006 |
| U1 | CRCP | -0.237 | 0.03057307 |
| U1 | CUX1 | -0.232 | 0.034044691 |
| U1 | TMED4 | -0.23 | 0.035524858 |
| U1 | STAT5A | -0.229 | 0.036285304 |
| U1 | ZNF609 | -0.226 | 0.038650402 |
| U1 | SEC16A | -0.225 | 0.039467335 |
| U1 | RBM33 | -0.224 | 0.04029888 |
| U1 | NHLRC3 | -0.222 | 0.042006599 |
| U1 | TBC1D2B | -0.221 | 0.042883176 |
| U1 | POM121C | -0.218 | 0.045606203 |
| U1 | TNRC18 | -0.218 | 0.045606203 |
| U1 | CUL1 | -0.217 | 0.046545661 |
| U1 | MBOAT1 | -0.217 | 0.046545661 |
| U1 | NUP214 | -0.216 | 0.047501356 |
| U1 | MGAT5 | -0.216 | 0.047501356 |
| U1 | PTPRA | -0.216 | 0.047501356 |
| U1 | HK2 | -0.215 | 0.048473495 |
| U1 | GRK7 | 0.242 | 0.04988004 |
| U1 | KDF1 | 0.242 | 0.04988004 |
| U1 | RAB26 | 0.242 | 0.04988004 |
| U1 | ZPBP2 | 0.242 | 0.04988004 |
| U1 | OR2H2 | 0.242 | 0.04988004 |
| U1 | GDF5 | 0.242 | 0.04988004 |
| U1 | SLC38A9 | 0.242 | 0.04988004 |
| U1 | LRRC71 | 0.243 | 0.048884232 |
| U1 | RABGAP1L | 0.243 | 0.048884232 |
| U1 | GVQW2 | 0.243 | 0.048884232 |
| U1 | APITD1-CORT | 0.243 | 0.048884232 |
| U1 | LGALS4 | 0.243 | 0.048884232 |
| U1 | ZNF606 | 0.244 | 0.047905166 |
| U1 | SLC6A17 | 0.244 | 0.047905166 |
| U1 | CC2D2B | 0.244 | 0.047905166 |
| U1 | TAL2 | 0.244 | 0.047905166 |
| U1 | ACAT1 | 0.244 | 0.047905166 |
| U1 | EPHX2 | 0.244 | 0.047905166 |
| U1 | DNAJC27 | 0.244 | 0.047905166 |
| U1 | PPAN-P2RY11 | 0.244 | 0.047905166 |
| U1 | RDM1 | 0.244 | 0.047905166 |
| U1 | APOC2 | 0.244 | 0.047905166 |
| U1 | MPP2 | 0.244 | 0.047905166 |
| U1 | SLC4A5 | 0.245 | 0.046942632 |
| U1 | ZNF705E | 0.245 | 0.046942632 |
| U1 | MEIOB | 0.245 | 0.046942632 |
| U1 | CLDN23 | 0.245 | 0.046942632 |
| U1 | CRACR2A | 0.245 | 0.046942632 |
| U1 | TIAM2 | 0.246 | 0.04599642 |
| U1 | RNF112 | 0.246 | 0.04599642 |
| U1 | OR56B1 | 0.246 | 0.04599642 |
| U1 | NDUFAF4 | 0.246 | 0.04599642 |
| U1 | HAUS4 | 0.247 | 0.045066325 |
| U1 | LY6K | 0.247 | 0.045066325 |
| U1 | ARL14EP | 0.247 | 0.045066325 |
| U1 | SEC14L2 | 0.247 | 0.045066325 |
| U1 | HEATR4 | 0.248 | 0.044152137 |
| U1 | ABCC2 | 0.248 | 0.044152137 |
| U1 | WDR64 | 0.248 | 0.044152137 |
| U1 | SPACA5B | 0.248 | 0.044152137 |
| U1 | C5orf63 | 0.249 | 0.043253652 |
| U1 | C1orf226 | 0.249 | 0.043253652 |
| U1 | TMC5 | 0.249 | 0.043253652 |
| U1 | DNAI1 | 0.249 | 0.043253652 |
| U1 | TMEM99 | 0.25 | 0.042370667 |
| U1 | PNMA3 | 0.25 | 0.042370667 |
| U1 | NIPA1 | 0.25 | 0.042370667 |
| U1 | AC073610.3 | 0.25 | 0.042370667 |
| U1 | GPAT2 | 0.251 | 0.041502976 |
| U1 | FXYD4 | 0.251 | 0.041502976 |
| U1 | C1orf127 | 0.251 | 0.041502976 |
| U1 | TMEM41A | 0.251 | 0.041502976 |
| U1 | MS4A4E | 0.251 | 0.041502976 |
| U1 | UCP3 | 0.251 | 0.041502976 |
| U1 | FBXW10 | 0.251 | 0.041502976 |
| U1 | LINC00959 | 0.252 | 0.04065038 |
| U1 | ATAD3C | 0.252 | 0.04065038 |
| U1 | RNF43 | 0.252 | 0.04065038 |
| U1 | PSTK | 0.252 | 0.04065038 |
| U1 | FPGT-TNNI3K | 0.252 | 0.04065038 |
| U1 | AC079447.1 | 0.252 | 0.04065038 |
| U1 | MPV17L | 0.253 | 0.039812677 |
| U1 | TESMIN | 0.253 | 0.039812677 |
| U1 | WNK3 | 0.253 | 0.039812677 |
| U1 | FANCM | 0.253 | 0.039812677 |
| U1 | IDNK | 0.253 | 0.039812677 |
| U1 | GPR160 | 0.253 | 0.039812677 |
| U1 | SLC46A1 | 0.253 | 0.039812677 |
| U1 | EMB | 0.254 | 0.038989668 |
| U1 | DIRC3 | 0.254 | 0.038989668 |
| U1 | LGALS9B | 0.254 | 0.038989668 |
| U1 | COMMD3-BMI1 | 0.254 | 0.038989668 |
| U1 | PFN4 | 0.254 | 0.038989668 |
| U1 | ZNF215 | 0.254 | 0.038989668 |
| U1 | HEXIM1 | 0.254 | 0.038989668 |
| U1 | RNASE10 | 0.254 | 0.038989668 |
| U1 | KIAA1328 | 0.255 | 0.038181155 |
| U1 | PRDM8 | 0.255 | 0.038181155 |
| U1 | SLC17A5 | 0.255 | 0.038181155 |
| U1 | RAB3A | 0.255 | 0.038181155 |
| U1 | GNG3 | 0.255 | 0.038181155 |
| U1 | SMAD3 | 0.256 | 0.037386943 |
| U1 | CASC4 | 0.256 | 0.037386943 |
| U1 | CCDC141 | 0.256 | 0.037386943 |
| U1 | SLC9A5 | 0.256 | 0.037386943 |
| U1 | PPIL3 | 0.256 | 0.037386943 |
| U1 | KLRF2 | 0.256 | 0.037386943 |
| U1 | HELQ | 0.257 | 0.036606835 |
| U1 | GOLGA6L4 | 0.257 | 0.036606835 |
| U1 | CLECL1 | 0.257 | 0.036606835 |
| U1 | OSGEPL1 | 0.257 | 0.036606835 |
| U1 | PPFIA3 | 0.257 | 0.036606835 |
| U1 | ULBP3 | 0.258 | 0.035840638 |
| U1 | EXPH5 | 0.258 | 0.035840638 |
| U1 | NAE1 | 0.258 | 0.035840638 |
| U1 | COG2 | 0.259 | 0.03508816 |
| U1 | CXorf21 | 0.259 | 0.03508816 |
| U1 | ZNF251 | 0.259 | 0.03508816 |
| U1 | CFAP61 | 0.259 | 0.03508816 |
| U1 | C15orf40 | 0.259 | 0.03508816 |
| U1 | CFAP100 | 0.259 | 0.03508816 |
| U1 | NCR3LG1 | 0.26 | 0.03434921 |
| U1 | C8orf44-SGK3 | 0.26 | 0.03434921 |
| U1 | PPM1L | 0.26 | 0.03434921 |
| U1 | PPP1R1B | 0.26 | 0.03434921 |
| U1 | EID1 | 0.26 | 0.03434921 |
| U1 | TEAD2 | 0.26 | 0.03434921 |
| U1 | GOLGA8F | 0.261 | 0.033623599 |
| U1 | KCNQ3 | 0.261 | 0.033623599 |
| U1 | MAP6 | 0.261 | 0.033623599 |
| U1 | SNPH | 0.261 | 0.033623599 |
| U1 | UQCRH | 0.261 | 0.033623599 |
| U1 | RHOXF1 | 0.262 | 0.032911138 |
| U1 | COMMD3 | 0.262 | 0.032911138 |
| U1 | DNAH9 | 0.262 | 0.032911138 |
| U1 | BEX1 | 0.263 | 0.032211641 |
| U1 | RPL23 | 0.263 | 0.032211641 |
| U1 | FBXO22 | 0.263 | 0.032211641 |
| U1 | ALDOB | 0.263 | 0.032211641 |
| U1 | ST7L | 0.263 | 0.032211641 |
| U1 | BMI1 | 0.264 | 0.031524923 |
| U1 | VSIG8 | 0.264 | 0.031524923 |
| U1 | AL360181.3 | 0.264 | 0.031524923 |
| U1 | BRSK2 | 0.264 | 0.031524923 |
| U1 | PQLC2 | 0.264 | 0.031524923 |
| U1 | CCNG1 | 0.264 | 0.031524923 |
| U1 | MCHR1 | 0.265 | 0.0308508 |
| U1 | MTFR1 | 0.265 | 0.0308508 |
| U1 | LAMB3 | 0.265 | 0.0308508 |
| U1 | SELENOI | 0.265 | 0.0308508 |
| U1 | MYLIP | 0.265 | 0.0308508 |
| U1 | NLRP14 | 0.266 | 0.030189091 |
| U1 | BCAS4 | 0.266 | 0.030189091 |
| U1 | HIST1H4C | 0.266 | 0.030189091 |
| U1 | ACTN2 | 0.266 | 0.030189091 |
| U1 | ADAL | 0.266 | 0.030189091 |
| U1 | AIFM2 | 0.266 | 0.030189091 |
| U1 | ANOS1 | 0.266 | 0.030189091 |
| U1 | WDR49 | 0.267 | 0.029539616 |
| U1 | SLC28A1 | 0.267 | 0.029539616 |
| U1 | RMDN2 | 0.269 | 0.028276649 |
| U1 | TUBGCP5 | 0.269 | 0.028276649 |
| U1 | KLB | 0.269 | 0.028276649 |
| U1 | RUBCNL | 0.269 | 0.028276649 |
| U1 | PDZD9 | 0.269 | 0.028276649 |
| U1 | GFPT2 | 0.269 | 0.028276649 |
| U1 | CYP4X1 | 0.27 | 0.027662806 |
| U1 | ORC6 | 0.271 | 0.027060489 |
| U1 | IL17B | 0.271 | 0.027060489 |
| U1 | PNPLA1 | 0.271 | 0.027060489 |
| U1 | HADHA | 0.271 | 0.027060489 |
| U1 | NIF3L1 | 0.271 | 0.027060489 |
| U1 | PDZRN3 | 0.271 | 0.027060489 |
| U1 | SPRED3 | 0.272 | 0.026469526 |
| U1 | GALM | 0.272 | 0.026469526 |
| U1 | SCN3B | 0.273 | 0.025889746 |
| U1 | PDE1B | 0.273 | 0.025889746 |
| U1 | AC011195.2 | 0.273 | 0.025889746 |
| U1 | SEC11C | 0.274 | 0.02532098 |
| U1 | DZANK1 | 0.274 | 0.02532098 |
| U1 | ZFP90 | 0.274 | 0.02532098 |
| U1 | SRR | 0.274 | 0.02532098 |
| U1 | MTX2 | 0.274 | 0.02532098 |
| U1 | MEF2C | 0.275 | 0.024763059 |
| U1 | S100G | 0.275 | 0.024763059 |
| U1 | RFX5 | 0.275 | 0.024763059 |
| U1 | TRIM67 | 0.275 | 0.024763059 |
| U1 | FAAH | 0.276 | 0.024215818 |
| U1 | PLET1 | 0.276 | 0.024215818 |
| U1 | ASPA | 0.276 | 0.024215818 |
| U1 | ZBBX | 0.277 | 0.023679092 |
| U1 | TACC2 | 0.277 | 0.023679092 |
| U1 | AC015813.2 | 0.277 | 0.023679092 |
| U1 | ANKAR | 0.277 | 0.023679092 |
| U1 | NAPSA | 0.277 | 0.023679092 |
| U1 | ABHD12B | 0.277 | 0.023679092 |
| U1 | FASTKD1 | 0.278 | 0.023152718 |
| U1 | ZDHHC19 | 0.278 | 0.023152718 |
| U1 | GPR75-ASB3 | 0.278 | 0.023152718 |
| U1 | ATP8A2 | 0.279 | 0.022636534 |
| U1 | HPX | 0.28 | 0.02213038 |
| U1 | MYOZ1 | 0.28 | 0.02213038 |
| U1 | PLPP7 | 0.281 | 0.0216341 |
| U1 | TPH1 | 0.281 | 0.0216341 |
| U1 | PIWIL2 | 0.281 | 0.0216341 |
| U1 | FAM71D | 0.282 | 0.021147535 |
| U1 | SNX31 | 0.282 | 0.021147535 |
| U1 | ANKS4B | 0.283 | 0.020670531 |
| U1 | ILDR2 | 0.284 | 0.020202935 |
| U1 | UPK3A | 0.284 | 0.020202935 |
| U1 | OR2Z1 | 0.284 | 0.020202935 |
| U1 | BEST2 | 0.285 | 0.019744596 |
| U1 | TRIM61 | 0.285 | 0.019744596 |
| U1 | ABHD1 | 0.285 | 0.019744596 |
| U1 | PDE4C | 0.286 | 0.019295363 |
| U1 | PPP1R36 | 0.287 | 0.018855088 |
| U1 | CAMKV | 0.287 | 0.018855088 |
| U1 | RBFOX2 | 0.288 | 0.018423624 |
| U1 | NRG4 | 0.288 | 0.018423624 |
| U1 | HTR2A | 0.289 | 0.018000827 |
| U1 | MPHOSPH6 | 0.289 | 0.018000827 |
| U1 | YJEFN3 | 0.29 | 0.017586553 |
| U1 | AL162231.1 | 0.29 | 0.017586553 |
| U1 | ACCSL | 0.291 | 0.01718066 |
| U1 | DCST1 | 0.291 | 0.01718066 |
| U1 | NQO1 | 0.291 | 0.01718066 |
| U1 | PRUNE2 | 0.292 | 0.016783009 |
| U1 | SIRT4 | 0.292 | 0.016783009 |
| U1 | SMTNL2 | 0.292 | 0.016783009 |
| U1 | APOO | 0.293 | 0.01639346 |
| U1 | RAD9B | 0.294 | 0.016011877 |
| U1 | UVRAG | 0.295 | 0.015638125 |
| U1 | RPL7 | 0.296 | 0.01527207 |
| U1 | ENTPD3 | 0.296 | 0.01527207 |
| U1 | MAATS1 | 0.296 | 0.01527207 |
| U1 | LINC01125 | 0.296 | 0.01527207 |
| U1 | CACNA1E | 0.296 | 0.01527207 |
| U1 | CYP4A22 | 0.296 | 0.01527207 |
| U1 | CCDC38 | 0.296 | 0.01527207 |
| U1 | NPAS4 | 0.297 | 0.01491358 |
| U1 | ITIH1 | 0.298 | 0.014562525 |
| U1 | GPC4 | 0.298 | 0.014562525 |
| U1 | UQCRB | 0.298 | 0.014562525 |
| U1 | SATL1 | 0.298 | 0.014562525 |
| U1 | PDE7A | 0.298 | 0.014562525 |
| U1 | GRAMD3 | 0.299 | 0.014218777 |
| U1 | PSMA8 | 0.299 | 0.014218777 |
| U1 | IL23A | 0.299 | 0.014218777 |
| U1 | EMILIN3 | 0.299 | 0.014218777 |
| U1 | C22orf15 | 0.299 | 0.014218777 |
| U1 | ZC2HC1C | 0.299 | 0.014218777 |
| U1 | IQANK1 | 0.299 | 0.014218777 |
| U1 | PTCHD3 | 0.3 | 0.013882207 |
| U1 | SLC39A5 | 0.3 | 0.013882207 |
| U1 | IGFL4 | 0.3 | 0.013882207 |
| U1 | PDC | 0.301 | 0.013552691 |
| U1 | HHIPL1 | 0.301 | 0.013552691 |
| U1 | FAM135B | 0.301 | 0.013552691 |
| U1 | MYLK3 | 0.302 | 0.013230104 |
| U1 | LRRC75A | 0.302 | 0.013230104 |
| U1 | IGFBPL1 | 0.303 | 0.012914325 |
| U1 | ERICH6 | 0.303 | 0.012914325 |
| U1 | GFAP | 0.304 | 0.012605233 |
| U1 | TIFAB | 0.304 | 0.012605233 |
| U1 | OCSTAMP | 0.304 | 0.012605233 |
| U1 | ANKRD65 | 0.304 | 0.012605233 |
| U1 | INPP5J | 0.305 | 0.012302707 |
| U1 | EDEM1 | 0.306 | 0.012006632 |
| U1 | CCL25 | 0.307 | 0.01171689 |
| U1 | SMTN | 0.307 | 0.01171689 |
| U1 | TMEM255A | 0.307 | 0.01171689 |
| U1 | HNRNPU | 0.307 | 0.01171689 |
| U1 | TSSK3 | 0.308 | 0.011433368 |
| U1 | LCMT2 | 0.308 | 0.011433368 |
| U1 | BTK | 0.308 | 0.011433368 |
| U1 | ST5 | 0.308 | 0.011433368 |
| U1 | RTBDN | 0.309 | 0.011155952 |
| U1 | XIRP1 | 0.309 | 0.011155952 |
| U1 | PTGES3L-AARSD1 | 0.309 | 0.011155952 |
| U1 | TRPM8 | 0.309 | 0.011155952 |
| U1 | SMIM10L2A | 0.309 | 0.011155952 |
| U1 | ABCB7 | 0.309 | 0.011155952 |
| U1 | UNC79 | 0.31 | 0.010884531 |
| U1 | UBA3 | 0.311 | 0.010618995 |
| U1 | PRPH2 | 0.312 | 0.010359236 |
| U1 | TMEM231 | 0.312 | 0.010359236 |
| U1 | MORF4L2 | 0.312 | 0.010359236 |
| U1 | FOXR1 | 0.313 | 0.010105146 |
| U1 | RND1 | 0.313 | 0.010105146 |
| U1 | CTGF | 0.313 | 0.010105146 |
| U1 | C1QTNF7 | 0.313 | 0.010105146 |
| U1 | GOLGA6L7P | 0.313 | 0.010105146 |
| U1 | AHNAK2 | 0.314 | 0.009856621 |
| U1 | ADAMTS3 | 0.314 | 0.009856621 |
| U1 | C3orf22 | 0.314 | 0.009856621 |
| U1 | ARL13A | 0.314 | 0.009856621 |
| U1 | BAZ2B | 0.315 | 0.009613557 |
| U1 | CELF4 | 0.315 | 0.009613557 |
| U1 | TAF7L | 0.315 | 0.009613557 |
| U1 | SYPL2 | 0.316 | 0.009375851 |
| U1 | ENDOU | 0.316 | 0.009375851 |
| U1 | A4GNT | 0.316 | 0.009375851 |
| U1 | UQCRHL | 0.316 | 0.009375851 |
| U1 | ADCY9 | 0.316 | 0.009375851 |
| U1 | B3GNT7 | 0.317 | 0.009143403 |
| U1 | ABCG5 | 0.318 | 0.008916113 |
| U1 | MLANA | 0.318 | 0.008916113 |
| U1 | SLC2A10 | 0.318 | 0.008916113 |
| U1 | CNBD2 | 0.318 | 0.008916113 |
| U1 | DNHD1 | 0.32 | 0.008476615 |
| U1 | AGRP | 0.322 | 0.008056593 |
| U1 | LEKR1 | 0.322 | 0.008056593 |
| U1 | KLHL20 | 0.322 | 0.008056593 |
| U1 | SOCS2 | 0.322 | 0.008056593 |
| U1 | CCL5 | 0.323 | 0.007853651 |
| U1 | ACSBG2 | 0.323 | 0.007853651 |
| U1 | KIAA1161 | 0.323 | 0.007853651 |
| U1 | FAM205C | 0.325 | 0.007461453 |
| U1 | PDZD3 | 0.325 | 0.007461453 |
| U1 | ESRRG | 0.325 | 0.007461453 |
| U1 | BUB3 | 0.325 | 0.007461453 |
| U1 | EFCAB11 | 0.326 | 0.007272018 |
| U1 | SPSB2 | 0.326 | 0.007272018 |
| U1 | CELA2A | 0.326 | 0.007272018 |
| U1 | LIN28A | 0.327 | 0.007086911 |
| U1 | IZUMO2 | 0.327 | 0.007086911 |
| U1 | MANSC4 | 0.327 | 0.007086911 |
| U1 | P2RY2 | 0.328 | 0.006906045 |
| U1 | TNPO1 | 0.328 | 0.006906045 |
| U1 | LRRC58 | 0.328 | 0.006906045 |
| U1 | HOGA1 | 0.328 | 0.006906045 |
| U1 | PDZD7 | 0.329 | 0.006729337 |
| U1 | RBKS | 0.329 | 0.006729337 |
| U1 | C1orf54 | 0.329 | 0.006729337 |
| U1 | FOLR1 | 0.33 | 0.006556703 |
| U1 | TMEM107 | 0.33 | 0.006556703 |
| U1 | STAC2 | 0.331 | 0.006388063 |
| U1 | AC112484.1 | 0.331 | 0.006388063 |
| U1 | CLEC18A | 0.331 | 0.006388063 |
| U1 | HSF2BP | 0.332 | 0.006223336 |
| U1 | INTS6L | 0.332 | 0.006223336 |
| U1 | PLA2G2F | 0.333 | 0.006062443 |
| U1 | RPL21 | 0.333 | 0.006062443 |
| U1 | ZNF883 | 0.333 | 0.006062443 |
| U1 | KRTAP5-7 | 0.334 | 0.005905307 |
| U1 | TMEM212 | 0.334 | 0.005905307 |
| U1 | RIBC2 | 0.335 | 0.005751851 |
| U1 | CLEC1B | 0.335 | 0.005751851 |
| U1 | DCST2 | 0.335 | 0.005751851 |
| U1 | OR2C1 | 0.337 | 0.00545568 |
| U1 | DENND2C | 0.337 | 0.00545568 |
| U1 | LIMA1 | 0.337 | 0.00545568 |
| U1 | FBXO16 | 0.338 | 0.005312819 |
| U1 | CLEC2B | 0.339 | 0.005173346 |
| U1 | TSGA13 | 0.339 | 0.005173346 |
| U1 | GOLGA8M | 0.339 | 0.005173346 |
| U1 | RAB42 | 0.34 | 0.00503719 |
| U1 | HYPK | 0.34 | 0.00503719 |
| U1 | SERPINI2 | 0.341 | 0.004904281 |
| U1 | DNAJB5 | 0.341 | 0.004904281 |
| U1 | SLC25A23 | 0.342 | 0.004774553 |
| U1 | LIPI | 0.342 | 0.004774553 |
| U1 | ASCL3 | 0.342 | 0.004774553 |
| U1 | SH3KBP1 | 0.343 | 0.004647938 |
| U1 | SRRM4 | 0.343 | 0.004647938 |
| U1 | RND2 | 0.344 | 0.004524371 |
| U1 | RAB36 | 0.345 | 0.004403788 |
| U1 | SPIRE2 | 0.345 | 0.004403788 |
| U1 | MYOG | 0.345 | 0.004403788 |
| U1 | AC011498.5 | 0.346 | 0.004286124 |
| U1 | ANGPT4 | 0.347 | 0.004171319 |
| U1 | FAM174B | 0.348 | 0.00405931 |
| U1 | SCG5 | 0.349 | 0.003950039 |
| U1 | FRMD4A | 0.349 | 0.003950039 |
| U1 | B9D1 | 0.349 | 0.003950039 |
| U1 | RPS6KA5 | 0.35 | 0.003843445 |
| U1 | MMRN1 | 0.351 | 0.003739471 |
| U1 | WDR63 | 0.351 | 0.003739471 |
| U1 | PPP1R1A | 0.352 | 0.00363806 |
| U1 | SMIM8 | 0.354 | 0.003442704 |
| U1 | GPD1L | 0.354 | 0.003442704 |
| U1 | RHO | 0.354 | 0.003442704 |
| U1 | RPP14 | 0.355 | 0.003348651 |
| U1 | CDK20 | 0.355 | 0.003348651 |
| U1 | CCT6B | 0.355 | 0.003348651 |
| U1 | DDX4 | 0.356 | 0.003256944 |
| U1 | MCMDC2 | 0.356 | 0.003256944 |
| U1 | ANTXRL | 0.357 | 0.00316753 |
| U1 | PINLYP | 0.357 | 0.00316753 |
| U1 | HIPK4 | 0.358 | 0.003080359 |
| U1 | PPP1R27 | 0.358 | 0.003080359 |
| U1 | ZFP3 | 0.359 | 0.002995381 |
| U1 | ZNF521 | 0.36 | 0.002912547 |
| U1 | ZPBP | 0.361 | 0.002831809 |
| U1 | NLRP11 | 0.361 | 0.002831809 |
| U1 | PSORS1C1 | 0.362 | 0.00275312 |
| U1 | NOS1AP | 0.363 | 0.002676432 |
| U1 | KRTAP5-9 | 0.364 | 0.002601702 |
| U1 | SLC12A3 | 0.365 | 0.002528884 |
| U1 | MTX3 | 0.366 | 0.002457934 |
| U1 | AK2 | 0.367 | 0.00238881 |
| U1 | CLCNKB | 0.367 | 0.00238881 |
| U1 | CKM | 0.367 | 0.00238881 |
| U1 | BVES | 0.368 | 0.002321471 |
| U1 | NPPB | 0.369 | 0.002255873 |
| U1 | CCDC65 | 0.37 | 0.002191979 |
| U1 | ACADM | 0.371 | 0.002129746 |
| U1 | BDNF | 0.371 | 0.002129746 |
| U1 | PPP1R3G | 0.371 | 0.002129746 |
| U1 | NKAIN2 | 0.372 | 0.002069138 |
| U1 | COQ7 | 0.372 | 0.002069138 |
| U1 | KCTD19 | 0.374 | 0.001952643 |
| U1 | GPR179 | 0.375 | 0.001896681 |
| U1 | AQP4 | 0.375 | 0.001896681 |
| U1 | IGSF1 | 0.376 | 0.001842196 |
| U1 | WNT8B | 0.378 | 0.001737517 |
| U1 | PPP1R1C | 0.379 | 0.001687254 |
| U1 | MORN1 | 0.379 | 0.001687254 |
| U1 | AKR7A2 | 0.379 | 0.001687254 |
| U1 | AZIN2 | 0.379 | 0.001687254 |
| U1 | TTLL9 | 0.38 | 0.001638332 |
| U1 | ALPK2 | 0.38 | 0.001638332 |
| U1 | LRRC3C | 0.38 | 0.001638332 |
| U1 | G6PC | 0.381 | 0.001590718 |
| U1 | IRGC | 0.381 | 0.001590718 |
| U1 | HRASLS | 0.381 | 0.001590718 |
| U1 | ETV3L | 0.381 | 0.001590718 |
| U1 | NKAPL | 0.382 | 0.001544382 |
| U1 | CHPF | 0.383 | 0.001499291 |
| U1 | BRS3 | 0.383 | 0.001499291 |
| U1 | BTBD18 | 0.384 | 0.001455415 |
| U1 | SENP6 | 0.385 | 0.001412726 |
| U1 | ACOT4 | 0.385 | 0.001412726 |
| U1 | GPR156 | 0.387 | 0.001330791 |
| U1 | MPZL2 | 0.388 | 0.001291489 |
| U1 | COX20 | 0.389 | 0.00125326 |
| U1 | ALDH3A1 | 0.391 | 0.001179919 |
| U1 | GRIN2D | 0.392 | 0.001144754 |
| U1 | C17orf98 | 0.392 | 0.001144754 |
| U1 | ENPP7 | 0.392 | 0.001144754 |
| U1 | ATP13A5 | 0.393 | 0.001110561 |
| U1 | SEL1L3 | 0.393 | 0.001110561 |
| U1 | TMPRSS12 | 0.393 | 0.001110561 |
| U1 | LRIT3 | 0.394 | 0.001077314 |
| U1 | GALR3 | 0.394 | 0.001077314 |
| U1 | GYG2 | 0.395 | 0.00104499 |
| U1 | INPP5B | 0.397 | 0.000983017 |
| U1 | UCN2 | 0.397 | 0.000983017 |
| U1 | F11 | 0.397 | 0.000983017 |
| U1 | LRRN4CL | 0.397 | 0.000983017 |
| U1 | SLC52A1 | 0.397 | 0.000983017 |
| U1 | ACTRT3 | 0.398 | 0.000953323 |
| U1 | TNFSF14 | 0.399 | 0.000924462 |
| U1 | SLC39A2 | 0.399 | 0.000924462 |
| U1 | DEPDC5 | 0.4 | 0.000896412 |
| U1 | NDRG2 | 0.401 | 0.000869153 |
| U1 | TMEM78 | 0.401 | 0.000869153 |
| U1 | TNN | 0.401 | 0.000869153 |
| U1 | ZNF532 | 0.402 | 0.000842665 |
| U1 | SNCA | 0.402 | 0.000842665 |
| U1 | HSPA2 | 0.402 | 0.000842665 |
| U1 | CCL23 | 0.403 | 0.000816926 |
| U1 | SLC25A41 | 0.403 | 0.000816926 |
| U1 | PTGES3L | 0.405 | 0.000767624 |
| U1 | CDNF | 0.407 | 0.000721096 |
| U1 | PLA2G10 | 0.407 | 0.000721096 |
| U1 | POLN | 0.407 | 0.000721096 |
| U1 | MDH1 | 0.407 | 0.000721096 |
| U1 | BTN2A2 | 0.409 | 0.000677199 |
| U1 | C19orf57 | 0.409 | 0.000677199 |
| U1 | TEDDM1 | 0.41 | 0.000656195 |
| U1 | AP1S3 | 0.41 | 0.000656195 |
| U1 | OFD1 | 0.411 | 0.000635798 |
| U1 | PER3 | 0.411 | 0.000635798 |
| U1 | LYG2 | 0.411 | 0.000635798 |
| U1 | SYT17 | 0.412 | 0.000615992 |
| U1 | RUFY4 | 0.412 | 0.000615992 |
| U1 | C9orf24 | 0.413 | 0.000596761 |
| U1 | WDR93 | 0.413 | 0.000596761 |
| U1 | NREP | 0.415 | 0.000559964 |
| U1 | CHST5 | 0.416 | 0.000542369 |
| U1 | ACTG2 | 0.417 | 0.00052529 |
| U1 | FBXO15 | 0.417 | 0.00052529 |
| U1 | CLDN18 | 0.417 | 0.00052529 |
| U1 | DNAH3 | 0.417 | 0.00052529 |
| U1 | SLC4A9 | 0.419 | 0.000492625 |
| U1 | BSND | 0.421 | 0.000461863 |
| U1 | PIGM | 0.423 | 0.0004329 |
| U1 | C14orf132 | 0.423 | 0.0004329 |
| U1 | C1orf194 | 0.423 | 0.0004329 |
| U1 | CUX2 | 0.426 | 0.000392619 |
| U1 | CAPN9 | 0.427 | 0.00037999 |
| U1 | MYOCD | 0.429 | 0.000355863 |
| U1 | RNF175 | 0.43 | 0.000344344 |
| U1 | RET | 0.43 | 0.000344344 |
| U1 | CDC20B | 0.43 | 0.000344344 |
| U1 | PTPRB | 0.431 | 0.000333174 |
| U1 | SPATA46 | 0.431 | 0.000333174 |
| U1 | ANXA10 | 0.433 | 0.000311844 |
| U1 | MCOLN3 | 0.433 | 0.000311844 |
| U1 | CFAP65 | 0.434 | 0.000301666 |
| U1 | FGF7 | 0.434 | 0.000301666 |
| U1 | NMNAT3 | 0.437 | 0.000272965 |
| U1 | SCUBE1 | 0.437 | 0.000272965 |
| U1 | C22orf42 | 0.439 | 0.000255275 |
| U1 | GPD2 | 0.439 | 0.000255275 |
| U1 | ANKFN1 | 0.439 | 0.000255275 |
| U1 | SLC35F1 | 0.44 | 0.000246839 |
| U1 | XKRX | 0.441 | 0.000238664 |
| U1 | DACT3 | 0.442 | 0.000230745 |
| U1 | CCDC62 | 0.443 | 0.000223072 |
| U1 | BTN1A1 | 0.444 | 0.00021564 |
| U1 | GRK4 | 0.445 | 0.00020844 |
| U1 | KRTAP5-8 | 0.445 | 0.00020844 |
| U1 | NLRP9 | 0.446 | 0.000201467 |
| U1 | CDKL3 | 0.446 | 0.000201467 |
| U1 | LHFPL4 | 0.446 | 0.000201467 |
| U1 | PLA2G2C | 0.446 | 0.000201467 |
| U1 | APOC4-APOC2 | 0.447 | 0.000194713 |
| U1 | REEP3 | 0.447 | 0.000194713 |
| U1 | PLIN5 | 0.449 | 0.000181839 |
| U1 | KIAA1755 | 0.452 | 0.000164019 |
| U1 | ATP23 | 0.453 | 0.000158454 |
| U1 | NOTO | 0.454 | 0.000153067 |
| U1 | DCANP1 | 0.455 | 0.000147853 |
| U1 | TEKT5 | 0.456 | 0.000142806 |
| U1 | CCNB3 | 0.457 | 0.000137922 |
| U1 | KIAA1257 | 0.458 | 0.000133195 |
| U1 | TIMM8A | 0.46 | 0.000124196 |
| U1 | BMP10 | 0.46 | 0.000124196 |
| U1 | TDRD7 | 0.46 | 0.000124196 |
| U1 | ZNF311 | 0.462 | 0.000115772 |
| U1 | DZIP1L | 0.462 | 0.000115772 |
| U1 | THEM4 | 0.465 | 0.000104141 |
| U1 | CLEC2A | 0.465 | 0.000104141 |
| U1 | MAP3K13 | 0.465 | 0.000104141 |
| U1 | FOXN4 | 0.465 | 0.000104141 |
| U1 | BHLHB9 | 0.466 | 0.000100515 |
| U1 | MESP2 | 0.468 | 9.36E-05 |
| U1 | KRTAP5-10 | 0.47 | 8.72E-05 |
| U1 | SEC16B | 0.471 | 8.41E-05 |
| U1 | SMYD3 | 0.471 | 8.41E-05 |
| U1 | NEO1 | 0.471 | 8.41E-05 |
| U1 | SAGE1 | 0.472 | 8.11E-05 |
| U1 | CYP4Z1 | 0.473 | 7.83E-05 |
| U1 | REN | 0.474 | 7.55E-05 |
| U1 | PRDM6 | 0.475 | 7.28E-05 |
| U1 | C19orf18 | 0.476 | 7.03E-05 |
| U1 | CFAP206 | 0.478 | 6.53E-05 |
| U1 | CDHR1 | 0.48 | 6.08E-05 |
| U1 | RRH | 0.483 | 5.44E-05 |
| U1 | LRRC31 | 0.483 | 5.44E-05 |
| U1 | GPR62 | 0.484 | 5.25E-05 |
| U1 | TRPM4 | 0.485 | 5.06E-05 |
| U1 | PACSIN1 | 0.489 | 4.36E-05 |
| U1 | LRMP | 0.495 | 3.49E-05 |
| U1 | HAPLN2 | 0.497 | 3.24E-05 |
| U1 | NFASC | 0.499 | 3.00E-05 |
| U1 | CYB5R2 | 0.501 | 2.78E-05 |
| U1 | EFCAB2 | 0.501 | 2.78E-05 |
| U1 | CACNB1 | 0.503 | 2.58E-05 |
| U1 | C19orf84 | 0.507 | 2.21E-05 |
| U1 | AADAC | 0.508 | 2.13E-05 |
| U1 | PCLO | 0.509 | 2.05E-05 |
| U1 | CLSTN2 | 0.511 | 1.90E-05 |
| U1 | PHF24 | 0.516 | 1.56E-05 |
| U1 | HOXC4 | 0.519 | 1.39E-05 |
| U1 | EBF1 | 0.52 | 1.34E-05 |
| U1 | SRL | 0.521 | 1.28E-05 |
| U1 | GPR20 | 0.522 | 1.23E-05 |
| U1 | SYT12 | 0.525 | 1.10E-05 |
| U1 | TRPC5OS | 0.53 | 8.98E-06 |
| U1 | THBS4 | 0.53 | 8.98E-06 |
| U1 | CLASP2 | 0.531 | 8.63E-06 |
| U1 | HEATR9 | 0.532 | 8.29E-06 |
| U1 | PLXDC1 | 0.534 | 7.65E-06 |
| U1 | MAGIX | 0.536 | 7.06E-06 |
| U1 | TDRD5 | 0.537 | 6.78E-06 |
| U1 | DES | 0.537 | 6.78E-06 |
| U1 | CRADD | 0.544 | 5.10E-06 |
| U1 | MSX2 | 0.544 | 5.10E-06 |
| U1 | COL7A1 | 0.545 | 4.89E-06 |
| U1 | ARSF | 0.545 | 4.89E-06 |
| U1 | NMUR1 | 0.546 | 4.69E-06 |
| U1 | ITIH3 | 0.546 | 4.69E-06 |
| U1 | WSCD1 | 0.548 | 4.32E-06 |
| U1 | BFSP1 | 0.552 | 3.66E-06 |
| U1 | KCNC3 | 0.554 | 3.37E-06 |
| U1 | CES3 | 0.555 | 3.23E-06 |
| U1 | PLCD4 | 0.555 | 3.23E-06 |
| U1 | SFTPC | 0.556 | 3.10E-06 |
| U1 | AGT | 0.556 | 3.10E-06 |
| U1 | SCN2B | 0.559 | 2.73E-06 |
| U1 | TNFSF9 | 0.565 | 2.12E-06 |
| U1 | CHST4 | 0.568 | 1.87E-06 |
| U1 | POPDC3 | 0.571 | 1.64E-06 |
| U1 | TTBK1 | 0.578 | 1.21E-06 |
| U1 | NINL | 0.579 | 1.16E-06 |
| U1 | CHST6 | 0.581 | 1.06E-06 |
| U1 | KLHDC9 | 0.581 | 1.06E-06 |
| U1 | DCDC1 | 0.581 | 1.06E-06 |
| U1 | TCF23 | 0.582 | 1.02E-06 |
| U1 | NTNG1 | 0.583 | 9.75E-07 |
| U1 | FILIP1 | 0.588 | 7.83E-07 |
| U1 | ZBTB33 | 0.594 | 6.00E-07 |
| U1 | AKR7A3 | 0.594 | 6.00E-07 |
| U1 | LAMC2 | 0.597 | 5.25E-07 |
| U1 | CAPN6 | 0.597 | 5.25E-07 |
| U1 | C2CD6 | 0.598 | 5.02E-07 |
| U1 | ASMT | 0.601 | 4.38E-07 |
| U1 | AADACL2 | 0.603 | 4.00E-07 |
| U1 | ASIC2 | 0.605 | 3.66E-07 |
| U1 | ADAM11 | 0.609 | 3.05E-07 |
| U1 | KANK4 | 0.61 | 2.91E-07 |
| U1 | TMEM198 | 0.623 | 1.60E-07 |
| U1 | RHBDL3 | 0.627 | 1.33E-07 |
| U1 | CNTNAP1 | 0.642 | 6.50E-08 |
| U1 | LMCD1 | 0.644 | 5.90E-08 |
| U1 | NR1I2 | 0.647 | 5.11E-08 |
| U1 | TPTE2 | 0.661 | 2.58E-08 |
| U1 | USP2 | 0.662 | 2.45E-08 |
| U1 | KCNN2 | 0.668 | 1.82E-08 |
| U1 | C8orf48 | 0.673 | 1.42E-08 |
| U1 | KLKB1 | 0.685 | 7.71E-09 |
| U1 | FAM107B | 0.687 | 6.96E-09 |
| U1 | IL23R | 0.691 | 5.67E-09 |
| U1 | SMIM9 | 0.698 | 3.94E-09 |
| U1 | TERB1 | 0.701 | 3.37E-09 |
| U1 | ZNF556 | 0.705 | 2.73E-09 |
| U1 | NECTIN4 | 0.708 | 2.33E-09 |
| U1 | MOBP | 0.708 | 2.33E-09 |
| U1 | SLC22A7 | 0.71 | 2.10E-09 |
| U1 | ACOT6 | 0.712 | 1.89E-09 |
| U1 | SOX5 | 0.713 | 1.79E-09 |
| U1 | SYNPO2 | 0.714 | 1.70E-09 |
| U1 | ACSM5 | 0.717 | 1.45E-09 |
| U1 | LYZL6 | 0.717 | 1.45E-09 |
| U1 | P2RY6 | 0.722 | 1.11E-09 |
| U1 | CA7 | 0.736 | 5.18E-10 |
| U1 | ANKRD45 | 0.743 | 3.52E-10 |
| U1 | PNPLA3 | 0.745 | 3.15E-10 |
| U1 | ASF1A | 0.751 | 2.26E-10 |
| U1 | PLA2G3 | 0.757 | 1.61E-10 |
| U1 | BABAM2 | 0.758 | 1.52E-10 |
| U1 | CD70 | 0.758 | 1.52E-10 |
| U1 | HMSD | 0.763 | 1.15E-10 |
| U1 | GTSF1L | 0.764 | 1.08E-10 |
| U1 | CADM1 | 0.765 | 1.02E-10 |
| U1 | MAP3K20 | 0.771 | 7.27E-11 |
| U1 | DKKL1 | 0.773 | 6.48E-11 |
| U1 | AKT3 | 0.774 | 6.12E-11 |
| U1 | ADGRF3 | 0.776 | 5.45E-11 |
| U1 | LINC00371 | 0.778 | 4.86E-11 |
| U1 | KLK13 | 0.782 | 3.85E-11 |
| U1 | DACH1 | 0.785 | 3.23E-11 |
| U1 | RSPH6A | 0.792 | 2.15E-11 |
| U1 | CCDC155 | 0.795 | 1.80E-11 |
| U1 | SCARA5 | 0.815 | 5.43E-12 |
| U1 | CCR10 | 0.818 | 4.53E-12 |
| U1 | GIPR | 0.819 | 4.26E-12 |
| U1 | OTOF | 0.825 | 2.95E-12 |
| U1 | CRIP3 | 0.827 | 2.61E-12 |
| U1 | PDYN | 0.831 | 2.04E-12 |
| U1 | ATP2A1 | 0.837 | 1.41E-12 |
| U1 | TXNDC8 | 0.838 | 1.32E-12 |
| U1 | FRK | 0.838 | 1.32E-12 |
| U1 | MCOLN2 | 0.84 | 1.17E-12 |
| U1 | SLC22A9 | 0.841 | 1.10E-12 |
| U1 | TGM5 | 0.841 | 1.10E-12 |
| U1 | SLC9C2 | 0.844 | 9.10E-13 |
| U1 | SEL1L2 | 0.845 | 8.55E-13 |
| U1 | PAH | 0.853 | 5.16E-13 |
| U1 | PKHD1 | 0.86 | 3.31E-13 |
| U1 | FBXO27 | 0.862 | 2.91E-13 |
| U1 | CCDC182 | 0.869 | 1.86E-13 |
| U1 | KCNMA1 | 0.874 | 1.34E-13 |
| U1 | SERPINB13 | 0.875 | 1.26E-13 |
| U1 | HMX3 | 0.881 | 8.52E-14 |
| U1 | CYP11A1 | 0.882 | 7.98E-14 |
| U1 | SLC10A2 | 0.894 | 3.62E-14 |
| U1 | TKTL1 | 0.895 | 3.39E-14 |
| U1 | KIAA1210 | 0.898 | 2.78E-14 |
| U1 | PRICKLE2 | 0.901 | 2.27E-14 |
| U1 | KCNF1 | 0.906 | 1.63E-14 |
| U1 | HMX2 | 0.908 | 1.42E-14 |
| U1 | CLCNKA | 0.91 | 1.24E-14 |
| U1 | SHROOM3 | 0.91 | 1.24E-14 |
| U1 | DNAJC5B | 0.911 | 1.16E-14 |
| U1 | SLC2A14 | 0.927 | 3.90E-15 |
| U1 | FSTL5 | 0.93 | 3.18E-15 |
| U1 | HSPB7 | 0.93 | 3.18E-15 |
| U1 | SNAP25 | 0.931 | 2.96E-15 |
| U1 | HRC | 0.931 | 2.96E-15 |
| U1 | PRL | 0.936 | 2.10E-15 |
| U1 | MLIP | 0.94 | 1.59E-15 |
| U1 | U1 | 1 | 2.13E-17 |
| RNU6ATAC39P | MAT2A | -0.401 | 0.000633309 |
| RNU6ATAC39P | PAN2 | -0.377 | 0.001287268 |
| RNU6ATAC39P | IKBKAP | -0.371 | 0.00152822 |
| RNU6ATAC39P | IFT27 | -0.341 | 0.003482289 |
| RNU6ATAC39P | SUGP2 | -0.337 | 0.003869771 |
| RNU6ATAC39P | FBXW8 | -0.33 | 0.004643188 |
| RNU6ATAC39P | SNRNP200 | -0.328 | 0.004888537 |
| RNU6ATAC39P | ABCC5 | -0.328 | 0.004888537 |
| RNU6ATAC39P | WDR27 | -0.327 | 0.005015558 |
| RNU6ATAC39P | MBD1 | -0.325 | 0.00527859 |
| RNU6ATAC39P | KDM4A | -0.321 | 0.005842354 |
| RNU6ATAC39P | AC010522.1 | -0.319 | 0.006144113 |
| RNU6ATAC39P | AL133352.1 | -0.318 | 0.006300195 |
| RNU6ATAC39P | METTL3 | -0.316 | 0.006623108 |
| RNU6ATAC39P | RIMBP3 | -0.315 | 0.006790081 |
| RNU6ATAC39P | BRPF3 | -0.313 | 0.00713542 |
| RNU6ATAC39P | MTOR | -0.312 | 0.007313934 |
| RNU6ATAC39P | PTCD3 | -0.31 | 0.007683029 |
| RNU6ATAC39P | TSTD2 | -0.306 | 0.008471679 |
| RNU6ATAC39P | MTMR4 | -0.305 | 0.008679823 |
| RNU6ATAC39P | TSGA10 | -0.305 | 0.008679823 |
| RNU6ATAC39P | ZNF45 | -0.305 | 0.008679823 |
| RNU6ATAC39P | ANKRD27 | -0.304 | 0.008892527 |
| RNU6ATAC39P | EARS2 | -0.301 | 0.009558846 |
| RNU6ATAC39P | ADAT2 | -0.301 | 0.009558846 |
| RNU6ATAC39P | ANAPC5 | -0.299 | 0.010027444 |
| RNU6ATAC39P | ERMARD | -0.299 | 0.010027444 |
| RNU6ATAC39P | NEU3 | -0.298 | 0.010269329 |
| RNU6ATAC39P | OCRL | -0.297 | 0.010516393 |
| RNU6ATAC39P | ZBTB40 | -0.295 | 0.011026438 |
| RNU6ATAC39P | ERCC3 | -0.295 | 0.011026438 |
| RNU6ATAC39P | TUBE1 | -0.295 | 0.011026438 |
| RNU6ATAC39P | AARS2 | -0.293 | 0.011558343 |
| RNU6ATAC39P | L3MBTL1 | -0.293 | 0.011558343 |
| RNU6ATAC39P | MBTPS1 | -0.292 | 0.011832738 |
| RNU6ATAC39P | RFT1 | -0.292 | 0.011832738 |
| RNU6ATAC39P | ZNF473 | -0.291 | 0.012112894 |
| RNU6ATAC39P | ZBED4 | -0.291 | 0.012112894 |
| RNU6ATAC39P | UBE3B | -0.291 | 0.012112894 |
| RNU6ATAC39P | EPG5 | -0.29 | 0.012398914 |
| RNU6ATAC39P | ZNF202 | -0.289 | 0.012690899 |
| RNU6ATAC39P | TXNDC5 | -0.289 | 0.012690899 |
| RNU6ATAC39P | NCBP3 | -0.288 | 0.012988954 |
| RNU6ATAC39P | ZC3H14 | -0.288 | 0.012988954 |
| RNU6ATAC39P | B3GNTL1 | -0.288 | 0.012988954 |
| RNU6ATAC39P | ERLIN1 | -0.283 | 0.014574014 |
| RNU6ATAC39P | GAS8 | -0.282 | 0.014910749 |
| RNU6ATAC39P | NIF3L1 | -0.282 | 0.014910749 |
| RNU6ATAC39P | COQ10A | -0.281 | 0.015254321 |
| RNU6ATAC39P | PIGO | -0.281 | 0.015254321 |
| RNU6ATAC39P | GEMIN4 | -0.279 | 0.015962433 |
| RNU6ATAC39P | SUPT7L | -0.279 | 0.015962433 |
| RNU6ATAC39P | CAPRIN2 | -0.279 | 0.015962433 |
| RNU6ATAC39P | USP49 | -0.278 | 0.016327208 |
| RNU6ATAC39P | GOLGA8O | -0.277 | 0.016699287 |
| RNU6ATAC39P | TECTA | -0.276 | 0.01707879 |
| RNU6ATAC39P | HECTD4 | -0.276 | 0.01707879 |
| RNU6ATAC39P | NFRKB | -0.274 | 0.017860558 |
| RNU6ATAC39P | XPOT | -0.274 | 0.017860558 |
| RNU6ATAC39P | FAN1 | -0.273 | 0.01826307 |
| RNU6ATAC39P | EP400NL | -0.273 | 0.01826307 |
| RNU6ATAC39P | QSOX2 | -0.272 | 0.018673502 |
| RNU6ATAC39P | OGT | -0.272 | 0.018673502 |
| RNU6ATAC39P | HENMT1 | -0.272 | 0.018673502 |
| RNU6ATAC39P | DDX23 | -0.271 | 0.019091981 |
| RNU6ATAC39P | POMGNT2 | -0.271 | 0.019091981 |
| RNU6ATAC39P | MRRF | -0.27 | 0.019518636 |
| RNU6ATAC39P | PLCG2 | -0.27 | 0.019518636 |
| RNU6ATAC39P | SMYD4 | -0.269 | 0.019953597 |
| RNU6ATAC39P | ZNF596 | -0.269 | 0.019953597 |
| RNU6ATAC39P | AGAP6 | -0.268 | 0.020396995 |
| RNU6ATAC39P | PGBD2 | -0.268 | 0.020396995 |
| RNU6ATAC39P | CDK5RAP2 | -0.268 | 0.020396995 |
| RNU6ATAC39P | SLC7A5 | -0.268 | 0.020396995 |
| RNU6ATAC39P | POLR1A | -0.267 | 0.020848963 |
| RNU6ATAC39P | KIZ | -0.267 | 0.020848963 |
| RNU6ATAC39P | PIWIL4 | -0.267 | 0.020848963 |
| RNU6ATAC39P | GCN1 | -0.267 | 0.020848963 |
| RNU6ATAC39P | TTC8 | -0.266 | 0.021309635 |
| RNU6ATAC39P | TUBGCP5 | -0.266 | 0.021309635 |
| RNU6ATAC39P | AC004076.1 | -0.266 | 0.021309635 |
| RNU6ATAC39P | AC020915.1 | -0.265 | 0.021779147 |
| RNU6ATAC39P | CFAP44 | -0.265 | 0.021779147 |
| RNU6ATAC39P | KBTBD11 | -0.265 | 0.021779147 |
| RNU6ATAC39P | DBF4B | -0.264 | 0.022257637 |
| RNU6ATAC39P | CYB5RL | -0.264 | 0.022257637 |
| RNU6ATAC39P | CUL9 | -0.264 | 0.022257637 |
| RNU6ATAC39P | TTI1 | -0.263 | 0.022745241 |
| RNU6ATAC39P | PCCB | -0.263 | 0.022745241 |
| RNU6ATAC39P | SLC15A2 | -0.263 | 0.022745241 |
| RNU6ATAC39P | PRDM10 | -0.263 | 0.022745241 |
| RNU6ATAC39P | CNOT1 | -0.262 | 0.023242102 |
| RNU6ATAC39P | PEX1 | -0.262 | 0.023242102 |
| RNU6ATAC39P | RANBP10 | -0.261 | 0.023748358 |
| RNU6ATAC39P | ATG2B | -0.261 | 0.023748358 |
| RNU6ATAC39P | SLC14A2 | -0.261 | 0.023748358 |
| RNU6ATAC39P | ZNF778 | -0.26 | 0.024264153 |
| RNU6ATAC39P | POLE | -0.26 | 0.024264153 |
| RNU6ATAC39P | REV1 | -0.26 | 0.024264153 |
| RNU6ATAC39P | C4B | -0.259 | 0.024789632 |
| RNU6ATAC39P | JMJD7-PLA2G4B | -0.259 | 0.024789632 |
| RNU6ATAC39P | DNAH1 | -0.259 | 0.024789632 |
| RNU6ATAC39P | TBP | -0.258 | 0.025324938 |
| RNU6ATAC39P | CNNM3 | -0.257 | 0.025870219 |
| RNU6ATAC39P | KBTBD4 | -0.257 | 0.025870219 |
| RNU6ATAC39P | HSDL1 | -0.257 | 0.025870219 |
| RNU6ATAC39P | NEMP1 | -0.257 | 0.025870219 |
| RNU6ATAC39P | TRMT13 | -0.257 | 0.025870219 |
| RNU6ATAC39P | SF3B1 | -0.257 | 0.025870219 |
| RNU6ATAC39P | HPS4 | -0.257 | 0.025870219 |
| RNU6ATAC39P | SEC22B | -0.256 | 0.026425624 |
| RNU6ATAC39P | CENPJ | -0.256 | 0.026425624 |
| RNU6ATAC39P | HUWE1 | -0.256 | 0.026425624 |
| RNU6ATAC39P | TMEM56-RWDD3 | -0.256 | 0.026425624 |
| RNU6ATAC39P | ZNF221 | -0.256 | 0.026425624 |
| RNU6ATAC39P | GTF2H2C | -0.256 | 0.026425624 |
| RNU6ATAC39P | RNF123 | -0.255 | 0.0269913 |
| RNU6ATAC39P | PHF7 | -0.255 | 0.0269913 |
| RNU6ATAC39P | L3HYPDH | -0.255 | 0.0269913 |
| RNU6ATAC39P | TSR1 | -0.255 | 0.0269913 |
| RNU6ATAC39P | SH3YL1 | -0.255 | 0.0269913 |
| RNU6ATAC39P | DCAF5 | -0.253 | 0.028154073 |
| RNU6ATAC39P | C12orf71 | -0.253 | 0.028154073 |
| RNU6ATAC39P | EIF2B5 | -0.253 | 0.028154073 |
| RNU6ATAC39P | GOLGA6L9 | -0.252 | 0.028751476 |
| RNU6ATAC39P | POM121 | -0.252 | 0.028751476 |
| RNU6ATAC39P | CCDC39 | -0.251 | 0.029359762 |
| RNU6ATAC39P | ANAPC1 | -0.251 | 0.029359762 |
| RNU6ATAC39P | C12orf29 | -0.25 | 0.029979087 |
| RNU6ATAC39P | POLR3E | -0.25 | 0.029979087 |
| RNU6ATAC39P | ZNF488 | -0.25 | 0.029979087 |
| RNU6ATAC39P | TRMU | -0.249 | 0.030609608 |
| RNU6ATAC39P | FITM2 | -0.249 | 0.030609608 |
| RNU6ATAC39P | GOLGA3 | -0.249 | 0.030609608 |
| RNU6ATAC39P | LHX4 | -0.249 | 0.030609608 |
| RNU6ATAC39P | PRPF8 | -0.248 | 0.031251485 |
| RNU6ATAC39P | C17orf97 | -0.248 | 0.031251485 |
| RNU6ATAC39P | SAP130 | -0.248 | 0.031251485 |
| RNU6ATAC39P | ZNF77 | -0.248 | 0.031251485 |
| RNU6ATAC39P | POGZ | -0.248 | 0.031251485 |
| RNU6ATAC39P | ZNF337 | -0.247 | 0.031904877 |
| RNU6ATAC39P | ACOX3 | -0.246 | 0.032569945 |
| RNU6ATAC39P | GOLGA6L10 | -0.246 | 0.032569945 |
| RNU6ATAC39P | ZC3H11A | -0.246 | 0.032569945 |
| RNU6ATAC39P | PTPN22 | -0.246 | 0.032569945 |
| RNU6ATAC39P | OGFOD2 | -0.246 | 0.032569945 |
| RNU6ATAC39P | C4A | -0.246 | 0.032569945 |
| RNU6ATAC39P | TAS2R5 | -0.246 | 0.032569945 |
| RNU6ATAC39P | BAHCC1 | -0.246 | 0.032569945 |
| RNU6ATAC39P | ZNF169 | -0.246 | 0.032569945 |
| RNU6ATAC39P | SLC37A4 | -0.244 | 0.033935763 |
| RNU6ATAC39P | PDIA6 | -0.244 | 0.033935763 |
| RNU6ATAC39P | WRN | -0.244 | 0.033935763 |
| RNU6ATAC39P | KLHL21 | -0.244 | 0.033935763 |
| RNU6ATAC39P | CRYGS | -0.244 | 0.033935763 |
| RNU6ATAC39P | FECH | -0.244 | 0.033935763 |
| RNU6ATAC39P | TBC1D24 | -0.244 | 0.033935763 |
| RNU6ATAC39P | ZNF155 | -0.243 | 0.034636841 |
| RNU6ATAC39P | EMC1 | -0.243 | 0.034636841 |
| RNU6ATAC39P | RFX8 | -0.243 | 0.034636841 |
| RNU6ATAC39P | BHMT2 | -0.243 | 0.034636841 |
| RNU6ATAC39P | KANK1 | -0.243 | 0.034636841 |
| RNU6ATAC39P | MTRR | -0.242 | 0.035350253 |
| RNU6ATAC39P | FAM86B2 | -0.242 | 0.035350253 |
| RNU6ATAC39P | AC233992.2 | -0.242 | 0.035350253 |
| RNU6ATAC39P | ZNF324B | -0.242 | 0.035350253 |
| RNU6ATAC39P | PPT2-EGFL8 | -0.242 | 0.035350253 |
| RNU6ATAC39P | DRC3 | -0.242 | 0.035350253 |
| RNU6ATAC39P | P2RY8 | -0.242 | 0.035350253 |
| RNU6ATAC39P | BBS4 | -0.241 | 0.036076166 |
| RNU6ATAC39P | DDX3Y | -0.241 | 0.036076166 |
| RNU6ATAC39P | NEURL4 | -0.24 | 0.03681475 |
| RNU6ATAC39P | KANSL3 | -0.24 | 0.03681475 |
| RNU6ATAC39P | GOPC | -0.24 | 0.03681475 |
| RNU6ATAC39P | FBXO10 | -0.24 | 0.03681475 |
| RNU6ATAC39P | EDEM3 | -0.24 | 0.03681475 |
| RNU6ATAC39P | NFYA | -0.239 | 0.037566174 |
| RNU6ATAC39P | RGS9BP | -0.239 | 0.037566174 |
| RNU6ATAC39P | TRMT12 | -0.239 | 0.037566174 |
| RNU6ATAC39P | C16orf58 | -0.239 | 0.037566174 |
| RNU6ATAC39P | GALNT3 | -0.238 | 0.03833061 |
| RNU6ATAC39P | CREBZF | -0.238 | 0.03833061 |
| RNU6ATAC39P | KRTAP5-7 | -0.238 | 0.03833061 |
| RNU6ATAC39P | SLC22A16 | -0.238 | 0.03833061 |
| RNU6ATAC39P | MIEF1 | -0.238 | 0.03833061 |
| RNU6ATAC39P | ZNF649 | -0.238 | 0.03833061 |
| RNU6ATAC39P | POGLUT1 | -0.238 | 0.03833061 |
| RNU6ATAC39P | ALAD | -0.237 | 0.03910823 |
| RNU6ATAC39P | CCDC14 | -0.237 | 0.03910823 |
| RNU6ATAC39P | MLEC | -0.237 | 0.03910823 |
| RNU6ATAC39P | HERC2 | -0.237 | 0.03910823 |
| RNU6ATAC39P | METAP1D | -0.236 | 0.039899206 |
| RNU6ATAC39P | ABCB10 | -0.236 | 0.039899206 |
| RNU6ATAC39P | PRSS51 | -0.236 | 0.039899206 |
| RNU6ATAC39P | ZNF10 | -0.236 | 0.039899206 |
| RNU6ATAC39P | BCS1L | -0.236 | 0.039899206 |
| RNU6ATAC39P | NUP188 | -0.235 | 0.040703715 |
| RNU6ATAC39P | FILIP1 | -0.235 | 0.040703715 |
| RNU6ATAC39P | INCA1 | -0.235 | 0.040703715 |
| RNU6ATAC39P | ZNF285 | -0.234 | 0.041521931 |
| RNU6ATAC39P | WDR19 | -0.234 | 0.041521931 |
| RNU6ATAC39P | TRMT44 | -0.234 | 0.041521931 |
| RNU6ATAC39P | ZNF749 | -0.234 | 0.041521931 |
| RNU6ATAC39P | GOLGA1 | -0.234 | 0.041521931 |
| RNU6ATAC39P | FBXO9 | -0.233 | 0.042354031 |
| RNU6ATAC39P | RPS4Y1 | -0.233 | 0.042354031 |
| RNU6ATAC39P | NCAPD3 | -0.233 | 0.042354031 |
| RNU6ATAC39P | TTC14 | -0.233 | 0.042354031 |
| RNU6ATAC39P | SZT2 | -0.233 | 0.042354031 |
| RNU6ATAC39P | EDRF1 | -0.233 | 0.042354031 |
| RNU6ATAC39P | ODF2 | -0.232 | 0.043200194 |
| RNU6ATAC39P | SLC25A37 | -0.232 | 0.043200194 |
| RNU6ATAC39P | PEX3 | -0.231 | 0.044060598 |
| RNU6ATAC39P | FBXO16 | -0.23 | 0.044935424 |
| RNU6ATAC39P | ZNF223 | -0.23 | 0.044935424 |
| RNU6ATAC39P | SPTBN2 | -0.23 | 0.044935424 |
| RNU6ATAC39P | LHFP | -0.23 | 0.044935424 |
| RNU6ATAC39P | DHX15 | -0.229 | 0.045824852 |
| RNU6ATAC39P | LRP3 | -0.229 | 0.045824852 |
| RNU6ATAC39P | IARS | -0.229 | 0.045824852 |
| RNU6ATAC39P | ZDHHC23 | -0.229 | 0.045824852 |
| RNU6ATAC39P | SETD4 | -0.229 | 0.045824852 |
| RNU6ATAC39P | ZNF384 | -0.228 | 0.046729064 |
| RNU6ATAC39P | TMEM27 | -0.228 | 0.046729064 |
| RNU6ATAC39P | RNF146 | -0.228 | 0.046729064 |
| RNU6ATAC39P | UGGT1 | -0.228 | 0.046729064 |
| RNU6ATAC39P | URB1 | -0.228 | 0.046729064 |
| RNU6ATAC39P | SCRN3 | -0.227 | 0.047648245 |
| RNU6ATAC39P | SLC14A1 | -0.227 | 0.047648245 |
| RNU6ATAC39P | CHRNA10 | -0.227 | 0.047648245 |
| RNU6ATAC39P | CCDC169 | -0.227 | 0.047648245 |
| RNU6ATAC39P | AP4B1 | -0.227 | 0.047648245 |
| RNU6ATAC39P | RAB9B | -0.226 | 0.048582577 |
| RNU6ATAC39P | MED12 | -0.226 | 0.048582577 |
| RNU6ATAC39P | SEMA4G | -0.226 | 0.048582577 |
| RNU6ATAC39P | IPPK | -0.226 | 0.048582577 |
| RNU6ATAC39P | NOXRED1 | -0.226 | 0.048582577 |
| RNU6ATAC39P | TBC1D20 | -0.226 | 0.048582577 |
| RNU6ATAC39P | MASP2 | -0.226 | 0.048582577 |
| RNU6ATAC39P | DIDO1 | -0.226 | 0.048582577 |
| RNU6ATAC39P | C2orf16 | -0.225 | 0.049532246 |
| RNU6ATAC39P | TBCEL | -0.225 | 0.049532246 |
| RNU6ATAC39P | SFMBT1 | -0.225 | 0.049532246 |
| RNU6ATAC39P | PIGV | -0.225 | 0.049532246 |
| RNU6ATAC39P | RCCD1 | -0.225 | 0.049532246 |
| RNU6ATAC39P | C19orf68 | 0.251 | 0.049345774 |
| RNU6ATAC39P | C3orf38 | 0.251 | 0.049345774 |
| RNU6ATAC39P | RNASE8 | 0.251 | 0.049345774 |
| RNU6ATAC39P | PNRC1 | 0.251 | 0.049345774 |
| RNU6ATAC39P | RSPH10B2 | 0.251 | 0.049345774 |
| RNU6ATAC39P | ACTR10 | 0.251 | 0.049345774 |
| RNU6ATAC39P | GMEB1 | 0.251 | 0.049345774 |
| RNU6ATAC39P | GPATCH3 | 0.251 | 0.049345774 |
| RNU6ATAC39P | OTOG | 0.252 | 0.048399112 |
| RNU6ATAC39P | FUOM | 0.252 | 0.048399112 |
| RNU6ATAC39P | RND1 | 0.252 | 0.048399112 |
| RNU6ATAC39P | RIPK2 | 0.252 | 0.048399112 |
| RNU6ATAC39P | TNIP1 | 0.252 | 0.048399112 |
| RNU6ATAC39P | PRKAG2 | 0.252 | 0.048399112 |
| RNU6ATAC39P | FAM81B | 0.252 | 0.048399112 |
| RNU6ATAC39P | PLGRKT | 0.252 | 0.048399112 |
| RNU6ATAC39P | KPNB1 | 0.252 | 0.048399112 |
| RNU6ATAC39P | NSMCE3 | 0.252 | 0.048399112 |
| RNU6ATAC39P | MYL6 | 0.252 | 0.048399112 |
| RNU6ATAC39P | PPP2R5E | 0.253 | 0.04746775 |
| RNU6ATAC39P | SYP | 0.253 | 0.04746775 |
| RNU6ATAC39P | OR6V1 | 0.253 | 0.04746775 |
| RNU6ATAC39P | ANP32A | 0.253 | 0.04746775 |
| RNU6ATAC39P | CHGB | 0.253 | 0.04746775 |
| RNU6ATAC39P | RPL19 | 0.254 | 0.046551504 |
| RNU6ATAC39P | CSNK2B | 0.254 | 0.046551504 |
| RNU6ATAC39P | RASA4B | 0.254 | 0.046551504 |
| RNU6ATAC39P | LIPF | 0.254 | 0.046551504 |
| RNU6ATAC39P | USP27X | 0.254 | 0.046551504 |
| RNU6ATAC39P | PAF1 | 0.254 | 0.046551504 |
| RNU6ATAC39P | MRPL40 | 0.254 | 0.046551504 |
| RNU6ATAC39P | RALA | 0.254 | 0.046551504 |
| RNU6ATAC39P | UCP2 | 0.255 | 0.045650189 |
| RNU6ATAC39P | CYTH1 | 0.255 | 0.045650189 |
| RNU6ATAC39P | ARPC1B | 0.255 | 0.045650189 |
| RNU6ATAC39P | SDF2 | 0.255 | 0.045650189 |
| RNU6ATAC39P | SMNDC1 | 0.255 | 0.045650189 |
| RNU6ATAC39P | SPATA2L | 0.255 | 0.045650189 |
| RNU6ATAC39P | ATP6V1H | 0.255 | 0.045650189 |
| RNU6ATAC39P | CSF2RA | 0.255 | 0.045650189 |
| RNU6ATAC39P | PRPF38B | 0.255 | 0.045650189 |
| RNU6ATAC39P | TLE6 | 0.256 | 0.044763624 |
| RNU6ATAC39P | SARAF | 0.256 | 0.044763624 |
| RNU6ATAC39P | DNAJC8 | 0.256 | 0.044763624 |
| RNU6ATAC39P | CFP | 0.256 | 0.044763624 |
| RNU6ATAC39P | SNX2 | 0.256 | 0.044763624 |
| RNU6ATAC39P | NAXE | 0.256 | 0.044763624 |
| RNU6ATAC39P | SLC16A6 | 0.256 | 0.044763624 |
| RNU6ATAC39P | PDAP1 | 0.256 | 0.044763624 |
| RNU6ATAC39P | SWSAP1 | 0.257 | 0.043891626 |
| RNU6ATAC39P | RPP38 | 0.257 | 0.043891626 |
| RNU6ATAC39P | COTL1 | 0.257 | 0.043891626 |
| RNU6ATAC39P | GNAI3 | 0.257 | 0.043891626 |
| RNU6ATAC39P | RSBN1L | 0.257 | 0.043891626 |
| RNU6ATAC39P | RPL12 | 0.257 | 0.043891626 |
| RNU6ATAC39P | RAP1B | 0.257 | 0.043891626 |
| RNU6ATAC39P | H3F3C | 0.258 | 0.043034014 |
| RNU6ATAC39P | HSPA1L | 0.258 | 0.043034014 |
| RNU6ATAC39P | RPS14 | 0.258 | 0.043034014 |
| RNU6ATAC39P | NOP53 | 0.258 | 0.043034014 |
| RNU6ATAC39P | SMIM20 | 0.258 | 0.043034014 |
| RNU6ATAC39P | TFPT | 0.258 | 0.043034014 |
| RNU6ATAC39P | RPS24 | 0.258 | 0.043034014 |
| RNU6ATAC39P | DAP3 | 0.258 | 0.043034014 |
| RNU6ATAC39P | SETSIP | 0.258 | 0.043034014 |
| RNU6ATAC39P | GLTP | 0.259 | 0.042190609 |
| RNU6ATAC39P | NKX2-5 | 0.259 | 0.042190609 |
| RNU6ATAC39P | NABP2 | 0.259 | 0.042190609 |
| RNU6ATAC39P | AATF | 0.259 | 0.042190609 |
| RNU6ATAC39P | ANKHD1 | 0.259 | 0.042190609 |
| RNU6ATAC39P | VAMP2 | 0.259 | 0.042190609 |
| RNU6ATAC39P | RNF34 | 0.259 | 0.042190609 |
| RNU6ATAC39P | RSL1D1 | 0.26 | 0.041361231 |
| RNU6ATAC39P | FEZ2 | 0.26 | 0.041361231 |
| RNU6ATAC39P | CMC2 | 0.26 | 0.041361231 |
| RNU6ATAC39P | APOPT1 | 0.26 | 0.041361231 |
| RNU6ATAC39P | COX6B1 | 0.26 | 0.041361231 |
| RNU6ATAC39P | AC023055.1 | 0.26 | 0.041361231 |
| RNU6ATAC39P | SLAMF9 | 0.26 | 0.041361231 |
| RNU6ATAC39P | RPL14 | 0.26 | 0.041361231 |
| RNU6ATAC39P | CDC42 | 0.26 | 0.041361231 |
| RNU6ATAC39P | TMEM41A | 0.26 | 0.041361231 |
| RNU6ATAC39P | GPANK1 | 0.261 | 0.040545703 |
| RNU6ATAC39P | AC114490.2 | 0.261 | 0.040545703 |
| RNU6ATAC39P | RPL23 | 0.261 | 0.040545703 |
| RNU6ATAC39P | SLC6A7 | 0.261 | 0.040545703 |
| RNU6ATAC39P | ATP5EP2 | 0.261 | 0.040545703 |
| RNU6ATAC39P | MYL12B | 0.261 | 0.040545703 |
| RNU6ATAC39P | SHPK | 0.261 | 0.040545703 |
| RNU6ATAC39P | MEIKIN | 0.261 | 0.040545703 |
| RNU6ATAC39P | BTF3 | 0.262 | 0.039743848 |
| RNU6ATAC39P | PPIL4 | 0.262 | 0.039743848 |
| RNU6ATAC39P | FBXL3 | 0.262 | 0.039743848 |
| RNU6ATAC39P | NFKBIB | 0.262 | 0.039743848 |
| RNU6ATAC39P | C7orf31 | 0.262 | 0.039743848 |
| RNU6ATAC39P | HMGA1 | 0.262 | 0.039743848 |
| RNU6ATAC39P | TMEM213 | 0.262 | 0.039743848 |
| RNU6ATAC39P | NFATC1 | 0.262 | 0.039743848 |
| RNU6ATAC39P | SERPINB6 | 0.262 | 0.039743848 |
| RNU6ATAC39P | ZNRD1 | 0.263 | 0.03895549 |
| RNU6ATAC39P | RHOB | 0.263 | 0.03895549 |
| RNU6ATAC39P | KIAA1522 | 0.263 | 0.03895549 |
| RNU6ATAC39P | SSU72 | 0.263 | 0.03895549 |
| RNU6ATAC39P | XIAP | 0.263 | 0.03895549 |
| RNU6ATAC39P | SERGEF | 0.263 | 0.03895549 |
| RNU6ATAC39P | OLA1 | 0.263 | 0.03895549 |
| RNU6ATAC39P | NDUFS4 | 0.263 | 0.03895549 |
| RNU6ATAC39P | BAG1 | 0.264 | 0.038180456 |
| RNU6ATAC39P | BORCS6 | 0.264 | 0.038180456 |
| RNU6ATAC39P | RPL41 | 0.264 | 0.038180456 |
| RNU6ATAC39P | ARL4C | 0.264 | 0.038180456 |
| RNU6ATAC39P | STYXL1 | 0.265 | 0.037418572 |
| RNU6ATAC39P | SULT1A1 | 0.265 | 0.037418572 |
| RNU6ATAC39P | CNPY2 | 0.265 | 0.037418572 |
| RNU6ATAC39P | C14orf166 | 0.265 | 0.037418572 |
| RNU6ATAC39P | ZNF101 | 0.265 | 0.037418572 |
| RNU6ATAC39P | RPL28 | 0.265 | 0.037418572 |
| RNU6ATAC39P | DGUOK | 0.265 | 0.037418572 |
| RNU6ATAC39P | F11R | 0.265 | 0.037418572 |
| RNU6ATAC39P | ERBB3 | 0.266 | 0.036669666 |
| RNU6ATAC39P | SLC2A6 | 0.266 | 0.036669666 |
| RNU6ATAC39P | ARPC3 | 0.266 | 0.036669666 |
| RNU6ATAC39P | RPL35A | 0.266 | 0.036669666 |
| RNU6ATAC39P | PLN | 0.266 | 0.036669666 |
| RNU6ATAC39P | UBE2Q1 | 0.266 | 0.036669666 |
| RNU6ATAC39P | TMCC3 | 0.266 | 0.036669666 |
| RNU6ATAC39P | HEXIM1 | 0.266 | 0.036669666 |
| RNU6ATAC39P | NOP58 | 0.267 | 0.035933567 |
| RNU6ATAC39P | 11-Sep | 0.267 | 0.035933567 |
| RNU6ATAC39P | YIPF4 | 0.267 | 0.035933567 |
| RNU6ATAC39P | APOBEC3C | 0.267 | 0.035933567 |
| RNU6ATAC39P | RNF145 | 0.267 | 0.035933567 |
| RNU6ATAC39P | ATP5E | 0.267 | 0.035933567 |
| RNU6ATAC39P | NUBP1 | 0.267 | 0.035933567 |
| RNU6ATAC39P | RNF149 | 0.268 | 0.035210106 |
| RNU6ATAC39P | ETS2 | 0.268 | 0.035210106 |
| RNU6ATAC39P | KLF6 | 0.268 | 0.035210106 |
| RNU6ATAC39P | TBCA | 0.268 | 0.035210106 |
| RNU6ATAC39P | OARD1 | 0.268 | 0.035210106 |
| RNU6ATAC39P | MED4 | 0.269 | 0.034499113 |
| RNU6ATAC39P | ESD | 0.269 | 0.034499113 |
| RNU6ATAC39P | PTPRH | 0.269 | 0.034499113 |
| RNU6ATAC39P | ASCC1 | 0.27 | 0.033800422 |
| RNU6ATAC39P | GABRR3 | 0.27 | 0.033800422 |
| RNU6ATAC39P | CDHR4 | 0.27 | 0.033800422 |
| RNU6ATAC39P | NDUFC1 | 0.27 | 0.033800422 |
| RNU6ATAC39P | AC090527.2 | 0.271 | 0.033113866 |
| RNU6ATAC39P | PSMA4 | 0.271 | 0.033113866 |
| RNU6ATAC39P | RBM3 | 0.271 | 0.033113866 |
| RNU6ATAC39P | PTMA | 0.271 | 0.033113866 |
| RNU6ATAC39P | SMIM11B | 0.271 | 0.033113866 |
| RNU6ATAC39P | APOBEC2 | 0.271 | 0.033113866 |
| RNU6ATAC39P | SOX4 | 0.271 | 0.033113866 |
| RNU6ATAC39P | LMBRD1 | 0.272 | 0.032439281 |
| RNU6ATAC39P | HLA-DMA | 0.272 | 0.032439281 |
| RNU6ATAC39P | CNTD1 | 0.272 | 0.032439281 |
| RNU6ATAC39P | AL159163.1 | 0.272 | 0.032439281 |
| RNU6ATAC39P | SLC9A3R2 | 0.272 | 0.032439281 |
| RNU6ATAC39P | RASL10B | 0.272 | 0.032439281 |
| RNU6ATAC39P | NME2 | 0.272 | 0.032439281 |
| RNU6ATAC39P | RAB34 | 0.273 | 0.031776503 |
| RNU6ATAC39P | LPP | 0.273 | 0.031776503 |
| RNU6ATAC39P | PPP3R1 | 0.273 | 0.031776503 |
| RNU6ATAC39P | POU2F2 | 0.273 | 0.031776503 |
| RNU6ATAC39P | NTAN1 | 0.273 | 0.031776503 |
| RNU6ATAC39P | PIGX | 0.273 | 0.031776503 |
| RNU6ATAC39P | RPL36AL | 0.273 | 0.031776503 |
| RNU6ATAC39P | HIST1H4B | 0.273 | 0.031776503 |
| RNU6ATAC39P | TINF2 | 0.274 | 0.03112537 |
| RNU6ATAC39P | MRPL48 | 0.274 | 0.03112537 |
| RNU6ATAC39P | NR3C1 | 0.274 | 0.03112537 |
| RNU6ATAC39P | GCC1 | 0.274 | 0.03112537 |
| RNU6ATAC39P | LRRFIP2 | 0.274 | 0.03112537 |
| RNU6ATAC39P | ENSA | 0.274 | 0.03112537 |
| RNU6ATAC39P | RPS16 | 0.275 | 0.030485721 |
| RNU6ATAC39P | EIF4EBP3 | 0.275 | 0.030485721 |
| RNU6ATAC39P | SIAH1 | 0.275 | 0.030485721 |
| RNU6ATAC39P | SEC11A | 0.275 | 0.030485721 |
| RNU6ATAC39P | C9orf66 | 0.275 | 0.030485721 |
| RNU6ATAC39P | DDX19B | 0.275 | 0.030485721 |
| RNU6ATAC39P | SS18L2 | 0.275 | 0.030485721 |
| RNU6ATAC39P | L1CAM | 0.275 | 0.030485721 |
| RNU6ATAC39P | KDSR | 0.276 | 0.029857396 |
| RNU6ATAC39P | DEDD | 0.276 | 0.029857396 |
| RNU6ATAC39P | FFAR2 | 0.276 | 0.029857396 |
| RNU6ATAC39P | RPL36A-HNRNPH2 | 0.276 | 0.029857396 |
| RNU6ATAC39P | ANTXR2 | 0.276 | 0.029857396 |
| RNU6ATAC39P | TMSB10 | 0.276 | 0.029857396 |
| RNU6ATAC39P | EMP3 | 0.276 | 0.029857396 |
| RNU6ATAC39P | RNF125 | 0.277 | 0.029240237 |
| RNU6ATAC39P | RPL27A | 0.277 | 0.029240237 |
| RNU6ATAC39P | MRPS7 | 0.277 | 0.029240237 |
| RNU6ATAC39P | DNPH1 | 0.277 | 0.029240237 |
| RNU6ATAC39P | RAP2B | 0.277 | 0.029240237 |
| RNU6ATAC39P | PARVB | 0.277 | 0.029240237 |
| RNU6ATAC39P | ATP6V1G1 | 0.277 | 0.029240237 |
| RNU6ATAC39P | RPS26 | 0.278 | 0.028634087 |
| RNU6ATAC39P | RRAGC | 0.278 | 0.028634087 |
| RNU6ATAC39P | PXN | 0.278 | 0.028634087 |
| RNU6ATAC39P | PYM1 | 0.278 | 0.028634087 |
| RNU6ATAC39P | UBXN1 | 0.278 | 0.028634087 |
| RNU6ATAC39P | CA5B | 0.278 | 0.028634087 |
| RNU6ATAC39P | VIM | 0.279 | 0.028038789 |
| RNU6ATAC39P | MIEN1 | 0.279 | 0.028038789 |
| RNU6ATAC39P | CLEC2B | 0.279 | 0.028038789 |
| RNU6ATAC39P | SLC35D2 | 0.279 | 0.028038789 |
| RNU6ATAC39P | ARHGEF38 | 0.279 | 0.028038789 |
| RNU6ATAC39P | PTX3 | 0.279 | 0.028038789 |
| RNU6ATAC39P | WASHC3 | 0.279 | 0.028038789 |
| RNU6ATAC39P | SNU13 | 0.28 | 0.02745419 |
| RNU6ATAC39P | S100A10 | 0.28 | 0.02745419 |
| RNU6ATAC39P | RNF7 | 0.28 | 0.02745419 |
| RNU6ATAC39P | MBD2 | 0.28 | 0.02745419 |
| RNU6ATAC39P | C1QTNF2 | 0.28 | 0.02745419 |
| RNU6ATAC39P | TNFAIP8L1 | 0.28 | 0.02745419 |
| RNU6ATAC39P | LCN8 | 0.28 | 0.02745419 |
| RNU6ATAC39P | RAP1A | 0.28 | 0.02745419 |
| RNU6ATAC39P | BCL10 | 0.28 | 0.02745419 |
| RNU6ATAC39P | AL139353.1 | 0.281 | 0.026880136 |
| RNU6ATAC39P | SPECC1L-ADORA2A | 0.281 | 0.026880136 |
| RNU6ATAC39P | PRELID2 | 0.281 | 0.026880136 |
| RNU6ATAC39P | EIF3K | 0.282 | 0.026316475 |
| RNU6ATAC39P | TOMM7 | 0.283 | 0.025763057 |
| RNU6ATAC39P | DYNLL2 | 0.283 | 0.025763057 |
| RNU6ATAC39P | FAM222B | 0.283 | 0.025763057 |
| RNU6ATAC39P | RPF1 | 0.283 | 0.025763057 |
| RNU6ATAC39P | PTP4A2 | 0.283 | 0.025763057 |
| RNU6ATAC39P | ANXA4 | 0.284 | 0.025219733 |
| RNU6ATAC39P | ADRB1 | 0.284 | 0.025219733 |
| RNU6ATAC39P | ASB3 | 0.284 | 0.025219733 |
| RNU6ATAC39P | AIF1 | 0.284 | 0.025219733 |
| RNU6ATAC39P | DARS | 0.284 | 0.025219733 |
| RNU6ATAC39P | PSMA7 | 0.285 | 0.024686356 |
| RNU6ATAC39P | GTF3C6 | 0.285 | 0.024686356 |
| RNU6ATAC39P | TGFBI | 0.285 | 0.024686356 |
| RNU6ATAC39P | MRPL21 | 0.285 | 0.024686356 |
| RNU6ATAC39P | SCRT2 | 0.285 | 0.024686356 |
| RNU6ATAC39P | S100A4 | 0.286 | 0.024162778 |
| RNU6ATAC39P | BORCS7 | 0.286 | 0.024162778 |
| RNU6ATAC39P | GPAT3 | 0.286 | 0.024162778 |
| RNU6ATAC39P | RPS9 | 0.286 | 0.024162778 |
| RNU6ATAC39P | TIMM9 | 0.286 | 0.024162778 |
| RNU6ATAC39P | FAM117B | 0.287 | 0.023648854 |
| RNU6ATAC39P | KRT15 | 0.287 | 0.023648854 |
| RNU6ATAC39P | ZNF564 | 0.288 | 0.023144442 |
| RNU6ATAC39P | LGALS9 | 0.288 | 0.023144442 |
| RNU6ATAC39P | SFXN3 | 0.289 | 0.022649399 |
| RNU6ATAC39P | PLCD3 | 0.289 | 0.022649399 |
| RNU6ATAC39P | MT-ND2 | 0.29 | 0.022163583 |
| RNU6ATAC39P | SERTAD1 | 0.29 | 0.022163583 |
| RNU6ATAC39P | CCNY | 0.29 | 0.022163583 |
| RNU6ATAC39P | GDPD1 | 0.29 | 0.022163583 |
| RNU6ATAC39P | NENF | 0.29 | 0.022163583 |
| RNU6ATAC39P | TRIM33 | 0.291 | 0.021686856 |
| RNU6ATAC39P | BLOC1S2 | 0.291 | 0.021686856 |
| RNU6ATAC39P | MT-ATP8 | 0.291 | 0.021686856 |
| RNU6ATAC39P | IER5 | 0.291 | 0.021686856 |
| RNU6ATAC39P | RAB11A | 0.291 | 0.021686856 |
| RNU6ATAC39P | SRC | 0.291 | 0.021686856 |
| RNU6ATAC39P | TTC1 | 0.292 | 0.021219078 |
| RNU6ATAC39P | PEX11B | 0.292 | 0.021219078 |
| RNU6ATAC39P | RPL35 | 0.292 | 0.021219078 |
| RNU6ATAC39P | C1orf158 | 0.292 | 0.021219078 |
| RNU6ATAC39P | NOL7 | 0.293 | 0.020760115 |
| RNU6ATAC39P | STOML3 | 0.293 | 0.020760115 |
| RNU6ATAC39P | NSA2 | 0.293 | 0.020760115 |
| RNU6ATAC39P | PRKAR2A | 0.293 | 0.020760115 |
| RNU6ATAC39P | CALCOCO2 | 0.294 | 0.020309829 |
| RNU6ATAC39P | TPPP | 0.294 | 0.020309829 |
| RNU6ATAC39P | MT-ND1 | 0.294 | 0.020309829 |
| RNU6ATAC39P | NCOR1 | 0.294 | 0.020309829 |
| RNU6ATAC39P | RPS20 | 0.294 | 0.020309829 |
| RNU6ATAC39P | DYNLRB1 | 0.294 | 0.020309829 |
| RNU6ATAC39P | DUSP11 | 0.295 | 0.019868088 |
| RNU6ATAC39P | RPLP2 | 0.295 | 0.019868088 |
| RNU6ATAC39P | PLEKHO1 | 0.295 | 0.019868088 |
| RNU6ATAC39P | MRPS16 | 0.295 | 0.019868088 |
| RNU6ATAC39P | BRK1 | 0.296 | 0.019434757 |
| RNU6ATAC39P | ZDHHC11B | 0.296 | 0.019434757 |
| RNU6ATAC39P | LRRC19 | 0.296 | 0.019434757 |
| RNU6ATAC39P | SERP1 | 0.296 | 0.019434757 |
| RNU6ATAC39P | CDKN2AIPNL | 0.296 | 0.019434757 |
| RNU6ATAC39P | NEMF | 0.297 | 0.019009708 |
| RNU6ATAC39P | YWHAB | 0.297 | 0.019009708 |
| RNU6ATAC39P | RAB13 | 0.297 | 0.019009708 |
| RNU6ATAC39P | PRR13 | 0.297 | 0.019009708 |
| RNU6ATAC39P | GLRX2 | 0.298 | 0.018592809 |
| RNU6ATAC39P | RPS8 | 0.298 | 0.018592809 |
| RNU6ATAC39P | RPS25 | 0.298 | 0.018592809 |
| RNU6ATAC39P | VDAC2 | 0.299 | 0.018183931 |
| RNU6ATAC39P | PPIG | 0.299 | 0.018183931 |
| RNU6ATAC39P | SNF8 | 0.299 | 0.018183931 |
| RNU6ATAC39P | UBE2D2 | 0.299 | 0.018183931 |
| RNU6ATAC39P | RPS27A | 0.299 | 0.018183931 |
| RNU6ATAC39P | KLF3 | 0.299 | 0.018183931 |
| RNU6ATAC39P | AGXT | 0.3 | 0.017782949 |
| RNU6ATAC39P | ARID4A | 0.3 | 0.017782949 |
| RNU6ATAC39P | GINM1 | 0.3 | 0.017782949 |
| RNU6ATAC39P | HNRNPUL2 | 0.301 | 0.017389737 |
| RNU6ATAC39P | CCDC71L | 0.301 | 0.017389737 |
| RNU6ATAC39P | ANXA11 | 0.301 | 0.017389737 |
| RNU6ATAC39P | TUBA1A | 0.301 | 0.017389737 |
| RNU6ATAC39P | TPPP3 | 0.301 | 0.017389737 |
| RNU6ATAC39P | C11orf1 | 0.302 | 0.017004169 |
| RNU6ATAC39P | NQO1 | 0.302 | 0.017004169 |
| RNU6ATAC39P | C9orf78 | 0.302 | 0.017004169 |
| RNU6ATAC39P | ZCCHC17 | 0.303 | 0.016626124 |
| RNU6ATAC39P | CATIP | 0.304 | 0.016255479 |
| RNU6ATAC39P | DCTPP1 | 0.304 | 0.016255479 |
| RNU6ATAC39P | SMS | 0.304 | 0.016255479 |
| RNU6ATAC39P | RPS12 | 0.304 | 0.016255479 |
| RNU6ATAC39P | TPT1 | 0.305 | 0.015892115 |
| RNU6ATAC39P | NDFIP1 | 0.305 | 0.015892115 |
| RNU6ATAC39P | ZNF511 | 0.305 | 0.015892115 |
| RNU6ATAC39P | ABCC11 | 0.306 | 0.015535913 |
| RNU6ATAC39P | IGF2BP2 | 0.306 | 0.015535913 |
| RNU6ATAC39P | CLTA | 0.307 | 0.015186756 |
| RNU6ATAC39P | TRIOBP | 0.307 | 0.015186756 |
| RNU6ATAC39P | PTPRA | 0.307 | 0.015186756 |
| RNU6ATAC39P | NAGS | 0.308 | 0.014844527 |
| RNU6ATAC39P | NCR3 | 0.308 | 0.014844527 |
| RNU6ATAC39P | BCAS2 | 0.309 | 0.014509112 |
| RNU6ATAC39P | ATP5H | 0.309 | 0.014509112 |
| RNU6ATAC39P | ATP5C1 | 0.31 | 0.014180397 |
| RNU6ATAC39P | PSMD1 | 0.31 | 0.014180397 |
| RNU6ATAC39P | IGSF9 | 0.31 | 0.014180397 |
| RNU6ATAC39P | GSTO1 | 0.31 | 0.014180397 |
| RNU6ATAC39P | LENG1 | 0.311 | 0.013858272 |
| RNU6ATAC39P | ARID4B | 0.311 | 0.013858272 |
| RNU6ATAC39P | RPL39 | 0.311 | 0.013858272 |
| RNU6ATAC39P | LRRFIP1 | 0.312 | 0.013542624 |
| RNU6ATAC39P | F2RL2 | 0.312 | 0.013542624 |
| RNU6ATAC39P | EID2 | 0.313 | 0.013233346 |
| RNU6ATAC39P | MRPS18B | 0.313 | 0.013233346 |
| RNU6ATAC39P | DCTN2 | 0.313 | 0.013233346 |
| RNU6ATAC39P | OAZ2 | 0.313 | 0.013233346 |
| RNU6ATAC39P | UXT | 0.313 | 0.013233346 |
| RNU6ATAC39P | CDKN1C | 0.314 | 0.012930329 |
| RNU6ATAC39P | SH3BGRL3 | 0.314 | 0.012930329 |
| RNU6ATAC39P | AFTPH | 0.314 | 0.012930329 |
| RNU6ATAC39P | MRFAP1 | 0.315 | 0.012633466 |
| RNU6ATAC39P | AC040162.1 | 0.315 | 0.012633466 |
| RNU6ATAC39P | GGCT | 0.315 | 0.012633466 |
| RNU6ATAC39P | BTBD19 | 0.315 | 0.012633466 |
| RNU6ATAC39P | POLR1D | 0.315 | 0.012633466 |
| RNU6ATAC39P | LBX1 | 0.315 | 0.012633466 |
| RNU6ATAC39P | CLIC1 | 0.316 | 0.012342653 |
| RNU6ATAC39P | TMEM203 | 0.316 | 0.012342653 |
| RNU6ATAC39P | CAMTA1 | 0.317 | 0.012057785 |
| RNU6ATAC39P | NT5C3A | 0.317 | 0.012057785 |
| RNU6ATAC39P | LST1 | 0.317 | 0.012057785 |
| RNU6ATAC39P | SCNM1 | 0.318 | 0.01177876 |
| RNU6ATAC39P | C2orf69 | 0.318 | 0.01177876 |
| RNU6ATAC39P | GTF2F2 | 0.319 | 0.011505478 |
| RNU6ATAC39P | MRPL10 | 0.319 | 0.011505478 |
| RNU6ATAC39P | TMEM51 | 0.32 | 0.011237837 |
| RNU6ATAC39P | TMEM256 | 0.321 | 0.010975739 |
| RNU6ATAC39P | THOC7 | 0.321 | 0.010975739 |
| RNU6ATAC39P | FAAP24 | 0.321 | 0.010975739 |
| RNU6ATAC39P | BRS3 | 0.321 | 0.010975739 |
| RNU6ATAC39P | TALDO1 | 0.321 | 0.010975739 |
| RNU6ATAC39P | FAM76A | 0.322 | 0.010719087 |
| RNU6ATAC39P | FAM19A1 | 0.322 | 0.010719087 |
| RNU6ATAC39P | MTFP1 | 0.323 | 0.010467785 |
| RNU6ATAC39P | RPL26 | 0.324 | 0.010221739 |
| RNU6ATAC39P | PRKX | 0.325 | 0.009980854 |
| RNU6ATAC39P | MEMO1 | 0.326 | 0.009745038 |
| RNU6ATAC39P | MINOS1 | 0.326 | 0.009745038 |
| RNU6ATAC39P | RTN4 | 0.326 | 0.009745038 |
| RNU6ATAC39P | STX8 | 0.326 | 0.009745038 |
| RNU6ATAC39P | RPL27 | 0.327 | 0.009514202 |
| RNU6ATAC39P | EXOSC1 | 0.327 | 0.009514202 |
| RNU6ATAC39P | PSME1 | 0.327 | 0.009514202 |
| RNU6ATAC39P | MUC12 | 0.328 | 0.009288253 |
| RNU6ATAC39P | LITAF | 0.328 | 0.009288253 |
| RNU6ATAC39P | ANKRD12 | 0.328 | 0.009288253 |
| RNU6ATAC39P | CCNI | 0.329 | 0.009067106 |
| RNU6ATAC39P | PPP2R5A | 0.329 | 0.009067106 |
| RNU6ATAC39P | TRNAU1AP | 0.33 | 0.008850671 |
| RNU6ATAC39P | C4orf36 | 0.33 | 0.008850671 |
| RNU6ATAC39P | WASF2 | 0.331 | 0.008638864 |
| RNU6ATAC39P | PIN4 | 0.331 | 0.008638864 |
| RNU6ATAC39P | FRMPD2 | 0.332 | 0.008431598 |
| RNU6ATAC39P | RPL21 | 0.332 | 0.008431598 |
| RNU6ATAC39P | BUD31 | 0.332 | 0.008431598 |
| RNU6ATAC39P | ZFAND3 | 0.332 | 0.008431598 |
| RNU6ATAC39P | KLF2 | 0.333 | 0.008228792 |
| RNU6ATAC39P | CRK | 0.333 | 0.008228792 |
| RNU6ATAC39P | TERF2 | 0.333 | 0.008228792 |
| RNU6ATAC39P | MED6 | 0.335 | 0.007836227 |
| RNU6ATAC39P | GABARAP | 0.335 | 0.007836227 |
| RNU6ATAC39P | C12orf50 | 0.335 | 0.007836227 |
| RNU6ATAC39P | TRAPPC3 | 0.336 | 0.007646307 |
| RNU6ATAC39P | SLC35E4 | 0.336 | 0.007646307 |
| RNU6ATAC39P | LYSMD2 | 0.336 | 0.007646307 |
| RNU6ATAC39P | PAFAH1B2 | 0.337 | 0.007460523 |
| RNU6ATAC39P | FRG1 | 0.337 | 0.007460523 |
| RNU6ATAC39P | CHRNG | 0.337 | 0.007460523 |
| RNU6ATAC39P | RP9 | 0.338 | 0.007278798 |
| RNU6ATAC39P | ITM2B | 0.338 | 0.007278798 |
| RNU6ATAC39P | KRT81 | 0.338 | 0.007278798 |
| RNU6ATAC39P | EAPP | 0.339 | 0.007101055 |
| RNU6ATAC39P | ZCCHC9 | 0.339 | 0.007101055 |
| RNU6ATAC39P | RBM26 | 0.34 | 0.006927218 |
| RNU6ATAC39P | RBPJL | 0.34 | 0.006927218 |
| RNU6ATAC39P | SULT1A4 | 0.34 | 0.006927218 |
| RNU6ATAC39P | PRDX6 | 0.34 | 0.006927218 |
| RNU6ATAC39P | NSRP1 | 0.341 | 0.006757214 |
| RNU6ATAC39P | RPS29 | 0.342 | 0.006590969 |
| RNU6ATAC39P | COX5A | 0.342 | 0.006590969 |
| RNU6ATAC39P | IMP3 | 0.342 | 0.006590969 |
| RNU6ATAC39P | PSMA2 | 0.343 | 0.006428411 |
| RNU6ATAC39P | TM4SF19 | 0.343 | 0.006428411 |
| RNU6ATAC39P | PDZD11 | 0.343 | 0.006428411 |
| RNU6ATAC39P | KHDRBS1 | 0.343 | 0.006428411 |
| RNU6ATAC39P | PABPN1 | 0.343 | 0.006428411 |
| RNU6ATAC39P | RASA4 | 0.343 | 0.006428411 |
| RNU6ATAC39P | PBDC1 | 0.343 | 0.006428411 |
| RNU6ATAC39P | MPHOSPH8 | 0.344 | 0.006269469 |
| RNU6ATAC39P | LYRM4 | 0.344 | 0.006269469 |
| RNU6ATAC39P | AC005726.2 | 0.344 | 0.006269469 |
| RNU6ATAC39P | DNAJC15 | 0.345 | 0.006114074 |
| RNU6ATAC39P | CWF19L2 | 0.345 | 0.006114074 |
| RNU6ATAC39P | NDUFAF2 | 0.345 | 0.006114074 |
| RNU6ATAC39P | PLLP | 0.345 | 0.006114074 |
| RNU6ATAC39P | PTRHD1 | 0.346 | 0.005962156 |
| RNU6ATAC39P | NDUFB5 | 0.346 | 0.005962156 |
| RNU6ATAC39P | UBE2R2 | 0.347 | 0.005813648 |
| RNU6ATAC39P | NDUFB1 | 0.347 | 0.005813648 |
| RNU6ATAC39P | CD52 | 0.347 | 0.005813648 |
| RNU6ATAC39P | RAPGEF6 | 0.348 | 0.005668483 |
| RNU6ATAC39P | EEF1G | 0.348 | 0.005668483 |
| RNU6ATAC39P | LUC7L3 | 0.351 | 0.005252397 |
| RNU6ATAC39P | RPL24 | 0.352 | 0.005119959 |
| RNU6ATAC39P | TMOD3 | 0.352 | 0.005119959 |
| RNU6ATAC39P | CFDP1 | 0.353 | 0.004990547 |
| RNU6ATAC39P | RPLP1 | 0.354 | 0.0048641 |
| RNU6ATAC39P | DENR | 0.355 | 0.004740559 |
| RNU6ATAC39P | PYURF | 0.356 | 0.004619864 |
| RNU6ATAC39P | DNAJC1 | 0.356 | 0.004619864 |
| RNU6ATAC39P | DHFR2 | 0.357 | 0.004501959 |
| RNU6ATAC39P | SLC18A1 | 0.357 | 0.004501959 |
| RNU6ATAC39P | ZNF277 | 0.357 | 0.004501959 |
| RNU6ATAC39P | CCDC91 | 0.358 | 0.004386787 |
| RNU6ATAC39P | TAF12 | 0.359 | 0.004274291 |
| RNU6ATAC39P | STMND1 | 0.359 | 0.004274291 |
| RNU6ATAC39P | AC093155.3 | 0.36 | 0.004164418 |
| RNU6ATAC39P | C11orf98 | 0.36 | 0.004164418 |
| RNU6ATAC39P | SLC35E3 | 0.361 | 0.004057114 |
| RNU6ATAC39P | PPP3CA | 0.361 | 0.004057114 |
| RNU6ATAC39P | RHOC | 0.361 | 0.004057114 |
| RNU6ATAC39P | HIGD2A | 0.362 | 0.003952326 |
| RNU6ATAC39P | SLU7 | 0.363 | 0.003850001 |
| RNU6ATAC39P | FOXP1 | 0.363 | 0.003850001 |
| RNU6ATAC39P | MT1A | 0.364 | 0.003750088 |
| RNU6ATAC39P | UBE2L3 | 0.365 | 0.003652538 |
| RNU6ATAC39P | ASB8 | 0.367 | 0.003464328 |
| RNU6ATAC39P | CXorf56 | 0.368 | 0.003373572 |
| RNU6ATAC39P | C18orf21 | 0.368 | 0.003373572 |
| RNU6ATAC39P | DNAJC30 | 0.368 | 0.003373572 |
| RNU6ATAC39P | RPS10 | 0.368 | 0.003373572 |
| RNU6ATAC39P | PI3 | 0.369 | 0.003284986 |
| RNU6ATAC39P | IL3RA | 0.369 | 0.003284986 |
| RNU6ATAC39P | NAP1L1 | 0.37 | 0.003198524 |
| RNU6ATAC39P | ZNF581 | 0.371 | 0.003114141 |
| RNU6ATAC39P | CHMP3 | 0.371 | 0.003114141 |
| RNU6ATAC39P | OTP | 0.372 | 0.003031792 |
| RNU6ATAC39P | RHOA | 0.372 | 0.003031792 |
| RNU6ATAC39P | MT-ND6 | 0.374 | 0.002873024 |
| RNU6ATAC39P | ZMAT2 | 0.374 | 0.002873024 |
| RNU6ATAC39P | CYP20A1 | 0.375 | 0.00279652 |
| RNU6ATAC39P | RDH11 | 0.376 | 0.002721881 |
| RNU6ATAC39P | DYNC1LI1 | 0.378 | 0.002578036 |
| RNU6ATAC39P | BRD3 | 0.378 | 0.002578036 |
| RNU6ATAC39P | MT-ND3 | 0.379 | 0.002508752 |
| RNU6ATAC39P | C12orf45 | 0.381 | 0.002375267 |
| RNU6ATAC39P | DNAJC7 | 0.381 | 0.002375267 |
| RNU6ATAC39P | IL27RA | 0.382 | 0.002310992 |
| RNU6ATAC39P | RAC1 | 0.383 | 0.002248314 |
| RNU6ATAC39P | PHF20 | 0.383 | 0.002248314 |
| RNU6ATAC39P | CRIP2 | 0.384 | 0.002187197 |
| RNU6ATAC39P | TAOK3 | 0.386 | 0.002069508 |
| RNU6ATAC39P | UBE2I | 0.386 | 0.002069508 |
| RNU6ATAC39P | SLC36A2 | 0.387 | 0.002012868 |
| RNU6ATAC39P | SNRNP35 | 0.387 | 0.002012868 |
| RNU6ATAC39P | FAM71D | 0.388 | 0.001957654 |
| RNU6ATAC39P | TAGLN2 | 0.389 | 0.001903833 |
| RNU6ATAC39P | FUNDC1 | 0.39 | 0.001851375 |
| RNU6ATAC39P | MAGEB17 | 0.391 | 0.001800247 |
| RNU6ATAC39P | POLR3GL | 0.393 | 0.001701864 |
| RNU6ATAC39P | UPF3B | 0.394 | 0.00165455 |
| RNU6ATAC39P | TCOF1 | 0.395 | 0.001608448 |
| RNU6ATAC39P | ZRSR2 | 0.398 | 0.001477145 |
| RNU6ATAC39P | RPL36 | 0.398 | 0.001477145 |
| RNU6ATAC39P | RPL38 | 0.399 | 0.001435621 |
| RNU6ATAC39P | PEBP1 | 0.399 | 0.001435621 |
| RNU6ATAC39P | CAMLG | 0.399 | 0.001435621 |
| RNU6ATAC39P | ANP32B | 0.399 | 0.001435621 |
| RNU6ATAC39P | RPS4X | 0.399 | 0.001435621 |
| RNU6ATAC39P | SARNP | 0.403 | 0.001280058 |
| RNU6ATAC39P | AL033529.1 | 0.406 | 0.001173765 |
| RNU6ATAC39P | EIF5B | 0.407 | 0.001140187 |
| RNU6ATAC39P | PHACTR4 | 0.407 | 0.001140187 |
| RNU6ATAC39P | ATP2B1 | 0.409 | 0.00107568 |
| RNU6ATAC39P | ACSM2B | 0.41 | 0.001044708 |
| RNU6ATAC39P | HSPA1A | 0.411 | 0.001014563 |
| RNU6ATAC39P | SFR1 | 0.412 | 0.000985225 |
| RNU6ATAC39P | FBXO33 | 0.413 | 0.000956674 |
| RNU6ATAC39P | S100A2 | 0.419 | 0.000800852 |
| RNU6ATAC39P | LINC00694 | 0.423 | 0.000710431 |
| RNU6ATAC39P | ISY1 | 0.423 | 0.000710431 |
| RNU6ATAC39P | HSCB | 0.425 | 0.000668867 |
| RNU6ATAC39P | AL031708.1 | 0.428 | 0.000610742 |
| RNU6ATAC39P | ZBTB7A | 0.43 | 0.000574641 |
| RNU6ATAC39P | DYNC1I2 | 0.431 | 0.000557346 |
| RNU6ATAC39P | NACA2 | 0.433 | 0.000524199 |
| RNU6ATAC39P | ZBTB8B | 0.434 | 0.000508324 |
| RNU6ATAC39P | KPNA6 | 0.435 | 0.000492898 |
| RNU6ATAC39P | RWDD1 | 0.438 | 0.000449199 |
| RNU6ATAC39P | C12orf43 | 0.438 | 0.000449199 |
| RNU6ATAC39P | RPA4 | 0.44 | 0.000422104 |
| RNU6ATAC39P | PPP1R2 | 0.44 | 0.000422104 |
| RNU6ATAC39P | TAX1BP3 | 0.441 | 0.000409137 |
| RNU6ATAC39P | NFIC | 0.441 | 0.000409137 |
| RNU6ATAC39P | LARP7 | 0.446 | 0.000349698 |
| RNU6ATAC39P | RPS13 | 0.447 | 0.000338824 |
| RNU6ATAC39P | PHF14 | 0.455 | 0.000262552 |
| RNU6ATAC39P | FAM177A1 | 0.456 | 0.000254241 |
| RNU6ATAC39P | CIR1 | 0.461 | 0.000216257 |
| RNU6ATAC39P | CELF1 | 0.465 | 0.000189778 |
| RNU6ATAC39P | DCP1A | 0.477 | 0.000127461 |
| RNU6ATAC39P | TRIM56 | 0.478 | 0.00012325 |
| RNU6ATAC39P | WBP4 | 0.485 | 9.72E-05 |
| RNU6ATAC39P | RTF1 | 0.489 | 8.48E-05 |
| RNU6ATAC39P | SYF2 | 0.494 | 7.14E-05 |
| RNU6ATAC39P | SNRPB2 | 0.495 | 6.89E-05 |
| RNU6ATAC39P | MED19 | 0.498 | 6.21E-05 |
| RNU6ATAC39P | LGALS14 | 0.499 | 6.00E-05 |
| RNU6ATAC39P | TAT | 0.5 | 5.79E-05 |
| RNU6ATAC39P | SPOP | 0.505 | 4.86E-05 |
| RNU6ATAC39P | ANKRD1 | 0.51 | 4.07E-05 |
| RNU6ATAC39P | WIPF2 | 0.513 | 3.65E-05 |
| RNU6ATAC39P | DUSP18 | 0.525 | 2.36E-05 |
| RNU6ATAC39P | HSPA1B | 0.528 | 2.12E-05 |
| RNU6ATAC39P | SMIM11A | 0.528 | 2.12E-05 |
| RNU6ATAC39P | ZCRB1 | 0.533 | 1.76E-05 |
| RNU6ATAC39P | TAF3 | 0.542 | 1.25E-05 |
| RNU6ATAC39P | SMAP1 | 0.543 | 1.21E-05 |
| RNU6ATAC39P | RBMX2 | 0.551 | 8.90E-06 |
| RNU6ATAC39P | LDB3 | 0.574 | 3.62E-06 |
| RNU6ATAC39P | CAMK1D | 0.582 | 2.63E-06 |
| RNU6ATAC39P | CFAP99 | 0.621 | 5.16E-07 |
| RNU6ATAC39P | RNU6ATAC39P | 1 | 3.67E-16 |
| RNU6-1274P | CDC42EP4 | -0.437 | 0.002965994 |
| RNU6-1274P | LONRF3 | -0.416 | 0.00470904 |
| RNU6-1274P | ATXN1 | -0.415 | 0.004811516 |
| RNU6-1274P | IRS2 | -0.414 | 0.004916005 |
| RNU6-1274P | MAP4K4 | -0.403 | 0.006208508 |
| RNU6-1274P | NEK3 | -0.397 | 0.007035759 |
| RNU6-1274P | EMILIN2 | -0.395 | 0.007332747 |
| RNU6-1274P | SMARCD3 | -0.389 | 0.008292358 |
| RNU6-1274P | INSR | -0.388 | 0.008462789 |
| RNU6-1274P | RTL8C | -0.387 | 0.008636345 |
| RNU6-1274P | SLC22A4 | -0.372 | 0.011649463 |
| RNU6-1274P | ST3GAL6 | -0.371 | 0.011880092 |
| RNU6-1274P | SLC36A1 | -0.37 | 0.01211476 |
| RNU6-1274P | CTNNA1 | -0.366 | 0.013095026 |
| RNU6-1274P | TADA2B | -0.366 | 0.013095026 |
| RNU6-1274P | SELENOK | -0.362 | 0.014144793 |
| RNU6-1274P | ELF3 | -0.361 | 0.014418568 |
| RNU6-1274P | NOL3 | -0.361 | 0.014418568 |
| RNU6-1274P | LIN7A | -0.359 | 0.014980171 |
| RNU6-1274P | ANXA2 | -0.358 | 0.015268132 |
| RNU6-1274P | IL17RA | -0.358 | 0.015268132 |
| RNU6-1274P | PARL | -0.357 | 0.015560956 |
| RNU6-1274P | ENTPD4 | -0.357 | 0.015560956 |
| RNU6-1274P | ZMYND10 | -0.355 | 0.016161462 |
| RNU6-1274P | PCOLCE | -0.355 | 0.016161462 |
| RNU6-1274P | FCGRT | -0.355 | 0.016161462 |
| RNU6-1274P | KIF1C | -0.352 | 0.01710041 |
| RNU6-1274P | GRN | -0.35 | 0.017752657 |
| RNU6-1274P | HEXB | -0.348 | 0.018426604 |
| RNU6-1274P | BTG1 | -0.346 | 0.019122838 |
| RNU6-1274P | P4HA2 | -0.346 | 0.019122838 |
| RNU6-1274P | RAF1 | -0.345 | 0.019479501 |
| RNU6-1274P | JAZF1 | -0.342 | 0.020584584 |
| RNU6-1274P | CDKN2D | -0.341 | 0.020964901 |
| RNU6-1274P | FAM114A1 | -0.34 | 0.021351327 |
| RNU6-1274P | GABARAPL1 | -0.34 | 0.021351327 |
| RNU6-1274P | AOAH | -0.339 | 0.021743941 |
| RNU6-1274P | KDM7A | -0.339 | 0.021743941 |
| RNU6-1274P | SIRPB2 | -0.339 | 0.021743941 |
| RNU6-1274P | ARHGEF11 | -0.339 | 0.021743941 |
| RNU6-1274P | WDFY3 | -0.339 | 0.021743941 |
| RNU6-1274P | HOMER3 | -0.339 | 0.021743941 |
| RNU6-1274P | NFAM1 | -0.338 | 0.022142824 |
| RNU6-1274P | ADAM9 | -0.338 | 0.022142824 |
| RNU6-1274P | RCN3 | -0.337 | 0.022548056 |
| RNU6-1274P | PLAUR | -0.337 | 0.022548056 |
| RNU6-1274P | SLC22A15 | -0.335 | 0.023377893 |
| RNU6-1274P | HERPUD1 | -0.335 | 0.023377893 |
| RNU6-1274P | TANK | -0.332 | 0.024672325 |
| RNU6-1274P | GLRX | -0.33 | 0.025569379 |
| RNU6-1274P | FAM32A | -0.33 | 0.025569379 |
| RNU6-1274P | TMEM63B | -0.33 | 0.025569379 |
| RNU6-1274P | STX2 | -0.329 | 0.026028392 |
| RNU6-1274P | SERPINB8 | -0.329 | 0.026028392 |
| RNU6-1274P | FAM131A | -0.329 | 0.026028392 |
| RNU6-1274P | S100A6 | -0.328 | 0.026494511 |
| RNU6-1274P | DVL3 | -0.328 | 0.026494511 |
| RNU6-1274P | AZI2 | -0.328 | 0.026494511 |
| RNU6-1274P | CHD7 | -0.327 | 0.026967825 |
| RNU6-1274P | COPZ2 | -0.327 | 0.026967825 |
| RNU6-1274P | TOX2 | -0.327 | 0.026967825 |
| RNU6-1274P | LYST | -0.325 | 0.027936386 |
| RNU6-1274P | ALDH3A2 | -0.325 | 0.027936386 |
| RNU6-1274P | UBTD1 | -0.325 | 0.027936386 |
| RNU6-1274P | ZNF438 | -0.325 | 0.027936386 |
| RNU6-1274P | ARHGEF40 | -0.324 | 0.028431811 |
| RNU6-1274P | MCUB | -0.324 | 0.028431811 |
| RNU6-1274P | CALM1 | -0.323 | 0.028934788 |
| RNU6-1274P | SRA1 | -0.323 | 0.028934788 |
| RNU6-1274P | PCBP4 | -0.323 | 0.028934788 |
| RNU6-1274P | DCBLD2 | -0.322 | 0.029445405 |
| RNU6-1274P | CD63 | -0.322 | 0.029445405 |
| RNU6-1274P | LMO4 | -0.322 | 0.029445405 |
| RNU6-1274P | SLC39A7 | -0.321 | 0.029963754 |
| RNU6-1274P | CTSK | -0.321 | 0.029963754 |
| RNU6-1274P | SELENOS | -0.32 | 0.030489928 |
| RNU6-1274P | MXD1 | -0.319 | 0.031024019 |
| RNU6-1274P | 9-Mar | -0.319 | 0.031024019 |
| RNU6-1274P | BBC3 | -0.318 | 0.03156612 |
| RNU6-1274P | ATP6V1E1 | -0.318 | 0.03156612 |
| RNU6-1274P | KCTD6 | -0.318 | 0.03156612 |
| RNU6-1274P | CNIH4 | -0.318 | 0.03156612 |
| RNU6-1274P | FAT2 | -0.318 | 0.03156612 |
| RNU6-1274P | ANKRD35 | -0.317 | 0.032116325 |
| RNU6-1274P | CST3 | -0.317 | 0.032116325 |
| RNU6-1274P | KIAA0355 | -0.316 | 0.032674729 |
| RNU6-1274P | MSANTD2 | -0.316 | 0.032674729 |
| RNU6-1274P | TGFA | -0.316 | 0.032674729 |
| RNU6-1274P | JMJD6 | -0.316 | 0.032674729 |
| RNU6-1274P | SMIM10 | -0.316 | 0.032674729 |
| RNU6-1274P | MRAS | -0.315 | 0.033241427 |
| RNU6-1274P | RCVRN | -0.315 | 0.033241427 |
| RNU6-1274P | NACC2 | -0.315 | 0.033241427 |
| RNU6-1274P | LGALS3 | -0.315 | 0.033241427 |
| RNU6-1274P | DCBLD1 | -0.315 | 0.033241427 |
| RNU6-1274P | TNS2 | -0.314 | 0.033816515 |
| RNU6-1274P | GPAT4 | -0.313 | 0.034400089 |
| RNU6-1274P | SLC35F6 | -0.312 | 0.034992246 |
| RNU6-1274P | NDUFB9 | -0.312 | 0.034992246 |
| RNU6-1274P | S100A10 | -0.312 | 0.034992246 |
| RNU6-1274P | SERGEF | -0.312 | 0.034992246 |
| RNU6-1274P | ZCCHC24 | -0.311 | 0.035593083 |
| RNU6-1274P | FAM129B | -0.311 | 0.035593083 |
| RNU6-1274P | LETM2 | -0.31 | 0.0362027 |
| RNU6-1274P | COX19 | -0.309 | 0.036821195 |
| RNU6-1274P | CELA1 | -0.309 | 0.036821195 |
| RNU6-1274P | SLC12A9 | -0.309 | 0.036821195 |
| RNU6-1274P | MYL12A | -0.309 | 0.036821195 |
| RNU6-1274P | MT1X | -0.309 | 0.036821195 |
| RNU6-1274P | CABP7 | -0.309 | 0.036821195 |
| RNU6-1274P | S100A11 | -0.309 | 0.036821195 |
| RNU6-1274P | MNDA | -0.309 | 0.036821195 |
| RNU6-1274P | GMPPA | -0.308 | 0.037448667 |
| RNU6-1274P | ANG | -0.308 | 0.037448667 |
| RNU6-1274P | STXBP1 | -0.307 | 0.038085216 |
| RNU6-1274P | MT1F | -0.307 | 0.038085216 |
| RNU6-1274P | SLC22A18 | -0.307 | 0.038085216 |
| RNU6-1274P | MYL12B | -0.307 | 0.038085216 |
| RNU6-1274P | SIMC1 | -0.307 | 0.038085216 |
| RNU6-1274P | RIN2 | -0.306 | 0.038730942 |
| RNU6-1274P | RBMS1 | -0.306 | 0.038730942 |
| RNU6-1274P | CCDC96 | -0.306 | 0.038730942 |
| RNU6-1274P | MGST3 | -0.305 | 0.039385948 |
| RNU6-1274P | RHOU | -0.305 | 0.039385948 |
| RNU6-1274P | ACSS3 | -0.305 | 0.039385948 |
| RNU6-1274P | PAPLN | -0.305 | 0.039385948 |
| RNU6-1274P | BRICD5 | -0.304 | 0.040050334 |
| RNU6-1274P | CTSH | -0.304 | 0.040050334 |
| RNU6-1274P | ERP27 | -0.304 | 0.040050334 |
| RNU6-1274P | SMPD1 | -0.304 | 0.040050334 |
| RNU6-1274P | SEMA4C | -0.304 | 0.040050334 |
| RNU6-1274P | CEBPD | -0.304 | 0.040050334 |
| RNU6-1274P | WWC2 | -0.304 | 0.040050334 |
| RNU6-1274P | MOSPD3 | -0.303 | 0.040724202 |
| RNU6-1274P | CATSPER1 | -0.303 | 0.040724202 |
| RNU6-1274P | PPARG | -0.302 | 0.041407657 |
| RNU6-1274P | ARHGAP12 | -0.302 | 0.041407657 |
| RNU6-1274P | SGCB | -0.302 | 0.041407657 |
| RNU6-1274P | PPARD | -0.302 | 0.041407657 |
| RNU6-1274P | MYL6 | -0.302 | 0.041407657 |
| RNU6-1274P | TM6SF1 | -0.301 | 0.0421008 |
| RNU6-1274P | SNX11 | -0.301 | 0.0421008 |
| RNU6-1274P | NBPF15 | -0.301 | 0.0421008 |
| RNU6-1274P | REEP5 | -0.3 | 0.042803735 |
| RNU6-1274P | SNX30 | -0.3 | 0.042803735 |
| RNU6-1274P | WIPI1 | -0.3 | 0.042803735 |
| RNU6-1274P | CBR3 | -0.3 | 0.042803735 |
| RNU6-1274P | PPFIBP2 | -0.299 | 0.043516568 |
| RNU6-1274P | RTL8B | -0.299 | 0.043516568 |
| RNU6-1274P | SPA17 | -0.299 | 0.043516568 |
| RNU6-1274P | MYO1C | -0.299 | 0.043516568 |
| RNU6-1274P | HECW2 | -0.299 | 0.043516568 |
| RNU6-1274P | ACVR1B | -0.298 | 0.044239403 |
| RNU6-1274P | CDA | -0.298 | 0.044239403 |
| RNU6-1274P | CLTCL1 | -0.298 | 0.044239403 |
| RNU6-1274P | HUS1B | -0.298 | 0.044239403 |
| RNU6-1274P | AGO4 | -0.298 | 0.044239403 |
| RNU6-1274P | P4HB | -0.298 | 0.044239403 |
| RNU6-1274P | HPS3 | -0.298 | 0.044239403 |
| RNU6-1274P | 1-Mar | -0.297 | 0.044972345 |
| RNU6-1274P | CAPN2 | -0.297 | 0.044972345 |
| RNU6-1274P | PHF13 | -0.297 | 0.044972345 |
| RNU6-1274P | CAST | -0.297 | 0.044972345 |
| RNU6-1274P | TRERF1 | -0.297 | 0.044972345 |
| RNU6-1274P | ATP6V0B | -0.296 | 0.045715501 |
| RNU6-1274P | DEF8 | -0.296 | 0.045715501 |
| RNU6-1274P | GORASP1 | -0.296 | 0.045715501 |
| RNU6-1274P | PDE8A | -0.296 | 0.045715501 |
| RNU6-1274P | PTPRJ | -0.295 | 0.046468977 |
| RNU6-1274P | MYDGF | -0.295 | 0.046468977 |
| RNU6-1274P | HAGH | -0.295 | 0.046468977 |
| RNU6-1274P | NDRG1 | -0.294 | 0.047232879 |
| RNU6-1274P | RNH1 | -0.294 | 0.047232879 |
| RNU6-1274P | RFX8 | -0.294 | 0.047232879 |
| RNU6-1274P | NTAN1 | -0.294 | 0.047232879 |
| RNU6-1274P | NOD2 | -0.294 | 0.047232879 |
| RNU6-1274P | CST6 | -0.294 | 0.047232879 |
| RNU6-1274P | SERTAD3 | -0.294 | 0.047232879 |
| RNU6-1274P | SIRT7 | -0.294 | 0.047232879 |
| RNU6-1274P | NR4A2 | -0.294 | 0.047232879 |
| RNU6-1274P | FBXO8 | -0.294 | 0.047232879 |
| RNU6-1274P | ZBTB18 | -0.293 | 0.048007315 |
| RNU6-1274P | C1orf162 | -0.293 | 0.048007315 |
| RNU6-1274P | PILRA | -0.292 | 0.048792393 |
| RNU6-1274P | CRAT | -0.292 | 0.048792393 |
| RNU6-1274P | ADCY9 | -0.292 | 0.048792393 |
| RNU6-1274P | ZFAND2A | -0.292 | 0.048792393 |
| RNU6-1274P | ARHGAP26 | -0.292 | 0.048792393 |
| RNU6-1274P | FAM20C | -0.292 | 0.048792393 |
| RNU6-1274P | S100A5 | -0.292 | 0.048792393 |
| RNU6-1274P | FCER1G | -0.291 | 0.049588221 |
| RNU6-1274P | ZDHHC3 | -0.291 | 0.049588221 |
| RNU6-1274P | NAALADL2 | 0.278 | 0.049537956 |
| RNU6-1274P | OR13C5 | 0.278 | 0.049537956 |
| RNU6-1274P | SF3B3 | 0.278 | 0.049537956 |
| RNU6-1274P | JPT2 | 0.278 | 0.049537956 |
| RNU6-1274P | ALDH1B1 | 0.278 | 0.049537956 |
| RNU6-1274P | TEX9 | 0.278 | 0.049537956 |
| RNU6-1274P | EIF2AK4 | 0.278 | 0.049537956 |
| RNU6-1274P | ERCC8 | 0.278 | 0.049537956 |
| RNU6-1274P | AMD1 | 0.278 | 0.049537956 |
| RNU6-1274P | MYOM2 | 0.279 | 0.048742806 |
| RNU6-1274P | SERHL2 | 0.279 | 0.048742806 |
| RNU6-1274P | NANP | 0.279 | 0.048742806 |
| RNU6-1274P | JRKL | 0.279 | 0.048742806 |
| RNU6-1274P | STX17 | 0.279 | 0.048742806 |
| RNU6-1274P | ZBED2 | 0.279 | 0.048742806 |
| RNU6-1274P | GDAP1 | 0.279 | 0.048742806 |
| RNU6-1274P | RRN3 | 0.28 | 0.047958399 |
| RNU6-1274P | CCDC138 | 0.28 | 0.047958399 |
| RNU6-1274P | TEX30 | 0.28 | 0.047958399 |
| RNU6-1274P | DAPK1 | 0.28 | 0.047958399 |
| RNU6-1274P | BBOF1 | 0.28 | 0.047958399 |
| RNU6-1274P | RBP5 | 0.28 | 0.047958399 |
| RNU6-1274P | DKC1 | 0.28 | 0.047958399 |
| RNU6-1274P | IPO7 | 0.28 | 0.047958399 |
| RNU6-1274P | ATP2C1 | 0.28 | 0.047958399 |
| RNU6-1274P | EXD2 | 0.28 | 0.047958399 |
| RNU6-1274P | C10orf95 | 0.281 | 0.047184627 |
| RNU6-1274P | NUFIP1 | 0.281 | 0.047184627 |
| RNU6-1274P | STON2 | 0.281 | 0.047184627 |
| RNU6-1274P | ERGIC1 | 0.281 | 0.047184627 |
| RNU6-1274P | TAF4B | 0.281 | 0.047184627 |
| RNU6-1274P | LSM14B | 0.281 | 0.047184627 |
| RNU6-1274P | ZNF793 | 0.281 | 0.047184627 |
| RNU6-1274P | HAS2 | 0.281 | 0.047184627 |
| RNU6-1274P | C22orf15 | 0.281 | 0.047184627 |
| RNU6-1274P | RBM12B | 0.282 | 0.046421382 |
| RNU6-1274P | SERF1B | 0.282 | 0.046421382 |
| RNU6-1274P | TRIM37 | 0.282 | 0.046421382 |
| RNU6-1274P | CAPRIN1 | 0.282 | 0.046421382 |
| RNU6-1274P | GAN | 0.282 | 0.046421382 |
| RNU6-1274P | FAM81B | 0.282 | 0.046421382 |
| RNU6-1274P | SIN3A | 0.282 | 0.046421382 |
| RNU6-1274P | RFXAP | 0.282 | 0.046421382 |
| RNU6-1274P | PLAGL1 | 0.282 | 0.046421382 |
| RNU6-1274P | POC1B | 0.283 | 0.045668558 |
| RNU6-1274P | MED17 | 0.283 | 0.045668558 |
| RNU6-1274P | NF1 | 0.283 | 0.045668558 |
| RNU6-1274P | TUBGCP4 | 0.283 | 0.045668558 |
| RNU6-1274P | TSTD2 | 0.283 | 0.045668558 |
| RNU6-1274P | HIST1H2BN | 0.283 | 0.045668558 |
| RNU6-1274P | LCLAT1 | 0.283 | 0.045668558 |
| RNU6-1274P | ADGRG1 | 0.284 | 0.044926046 |
| RNU6-1274P | RBM4B | 0.284 | 0.044926046 |
| RNU6-1274P | FAM186B | 0.284 | 0.044926046 |
| RNU6-1274P | ZMYM4 | 0.284 | 0.044926046 |
| RNU6-1274P | IFT74 | 0.284 | 0.044926046 |
| RNU6-1274P | BCR | 0.284 | 0.044926046 |
| RNU6-1274P | TTLL5 | 0.284 | 0.044926046 |
| RNU6-1274P | ZBTB46 | 0.284 | 0.044926046 |
| RNU6-1274P | TOGARAM1 | 0.285 | 0.044193741 |
| RNU6-1274P | C14orf159 | 0.285 | 0.044193741 |
| RNU6-1274P | NOL8 | 0.285 | 0.044193741 |
| RNU6-1274P | TMEM182 | 0.285 | 0.044193741 |
| RNU6-1274P | ZNF140 | 0.285 | 0.044193741 |
| RNU6-1274P | CHD4 | 0.285 | 0.044193741 |
| RNU6-1274P | ZMYND8 | 0.285 | 0.044193741 |
| RNU6-1274P | SH3PXD2A | 0.285 | 0.044193741 |
| RNU6-1274P | NFRKB | 0.286 | 0.043471537 |
| RNU6-1274P | ZNF346 | 0.286 | 0.043471537 |
| RNU6-1274P | ZNF792 | 0.286 | 0.043471537 |
| RNU6-1274P | HOMEZ | 0.286 | 0.043471537 |
| RNU6-1274P | SP7 | 0.286 | 0.043471537 |
| RNU6-1274P | ELFN2 | 0.286 | 0.043471537 |
| RNU6-1274P | SLC7A1 | 0.286 | 0.043471537 |
| RNU6-1274P | GLS2 | 0.287 | 0.042759329 |
| RNU6-1274P | SHQ1 | 0.287 | 0.042759329 |
| RNU6-1274P | LARP4B | 0.287 | 0.042759329 |
| RNU6-1274P | ZMYM1 | 0.287 | 0.042759329 |
| RNU6-1274P | SPACA9 | 0.287 | 0.042759329 |
| RNU6-1274P | IARS | 0.287 | 0.042759329 |
| RNU6-1274P | CYP7B1 | 0.287 | 0.042759329 |
| RNU6-1274P | DDO | 0.287 | 0.042759329 |
| RNU6-1274P | AKAP11 | 0.288 | 0.042057011 |
| RNU6-1274P | AL121594.3 | 0.288 | 0.042057011 |
| RNU6-1274P | LGSN | 0.288 | 0.042057011 |
| RNU6-1274P | GRAP | 0.288 | 0.042057011 |
| RNU6-1274P | TIMM10B | 0.288 | 0.042057011 |
| RNU6-1274P | DNAJC16 | 0.288 | 0.042057011 |
| RNU6-1274P | POP1 | 0.288 | 0.042057011 |
| RNU6-1274P | NEK11 | 0.288 | 0.042057011 |
| RNU6-1274P | FAM229B | 0.288 | 0.042057011 |
| RNU6-1274P | SMARCC1 | 0.288 | 0.042057011 |
| RNU6-1274P | CLEC16A | 0.288 | 0.042057011 |
| RNU6-1274P | SYTL4 | 0.288 | 0.042057011 |
| RNU6-1274P | CCDC171 | 0.288 | 0.042057011 |
| RNU6-1274P | TAS2R8 | 0.288 | 0.042057011 |
| RNU6-1274P | ZBTB8A | 0.289 | 0.041364479 |
| RNU6-1274P | PSPH | 0.289 | 0.041364479 |
| RNU6-1274P | PM20D2 | 0.289 | 0.041364479 |
| RNU6-1274P | KCNQ5 | 0.289 | 0.041364479 |
| RNU6-1274P | SMYD3 | 0.289 | 0.041364479 |
| RNU6-1274P | DCAF17 | 0.289 | 0.041364479 |
| RNU6-1274P | ROBO3 | 0.289 | 0.041364479 |
| RNU6-1274P | LIMS1 | 0.289 | 0.041364479 |
| RNU6-1274P | KIAA0391 | 0.29 | 0.040681629 |
| RNU6-1274P | B4GALT6 | 0.29 | 0.040681629 |
| RNU6-1274P | ERICH6 | 0.29 | 0.040681629 |
| RNU6-1274P | PCDHGA12 | 0.29 | 0.040681629 |
| RNU6-1274P | SLK | 0.29 | 0.040681629 |
| RNU6-1274P | TMEM136 | 0.29 | 0.040681629 |
| RNU6-1274P | DCAF16 | 0.291 | 0.040008359 |
| RNU6-1274P | PTPN11 | 0.291 | 0.040008359 |
| RNU6-1274P | C12orf66 | 0.291 | 0.040008359 |
| RNU6-1274P | TSPYL4 | 0.291 | 0.040008359 |
| RNU6-1274P | ZNF569 | 0.291 | 0.040008359 |
| RNU6-1274P | ZNF81 | 0.291 | 0.040008359 |
| RNU6-1274P | NUBPL | 0.291 | 0.040008359 |
| RNU6-1274P | ZKSCAN4 | 0.291 | 0.040008359 |
| RNU6-1274P | ITPR1 | 0.291 | 0.040008359 |
| RNU6-1274P | PRR5L | 0.291 | 0.040008359 |
| RNU6-1274P | ARHGEF35 | 0.291 | 0.040008359 |
| RNU6-1274P | MRPL19 | 0.292 | 0.039344564 |
| RNU6-1274P | MTAP | 0.292 | 0.039344564 |
| RNU6-1274P | MAP7 | 0.292 | 0.039344564 |
| RNU6-1274P | KLHL9 | 0.292 | 0.039344564 |
| RNU6-1274P | TRANK1 | 0.292 | 0.039344564 |
| RNU6-1274P | ARHGAP25 | 0.292 | 0.039344564 |
| RNU6-1274P | GCN1 | 0.292 | 0.039344564 |
| RNU6-1274P | PRKCQ | 0.292 | 0.039344564 |
| RNU6-1274P | SPON1 | 0.292 | 0.039344564 |
| RNU6-1274P | SPN | 0.292 | 0.039344564 |
| RNU6-1274P | MORC2 | 0.292 | 0.039344564 |
| RNU6-1274P | RAB40A | 0.293 | 0.038690144 |
| RNU6-1274P | GPR89A | 0.293 | 0.038690144 |
| RNU6-1274P | KIF2A | 0.293 | 0.038690144 |
| RNU6-1274P | RAD51D | 0.293 | 0.038690144 |
| RNU6-1274P | CCDC86 | 0.294 | 0.038044997 |
| RNU6-1274P | CDK6 | 0.294 | 0.038044997 |
| RNU6-1274P | GPR135 | 0.294 | 0.038044997 |
| RNU6-1274P | TTC36 | 0.294 | 0.038044997 |
| RNU6-1274P | LZTS2 | 0.294 | 0.038044997 |
| RNU6-1274P | C2orf80 | 0.294 | 0.038044997 |
| RNU6-1274P | TBXA2R | 0.294 | 0.038044997 |
| RNU6-1274P | ABHD2 | 0.295 | 0.03740902 |
| RNU6-1274P | C17orf78 | 0.295 | 0.03740902 |
| RNU6-1274P | MB | 0.295 | 0.03740902 |
| RNU6-1274P | MRM1 | 0.295 | 0.03740902 |
| RNU6-1274P | ACY3 | 0.295 | 0.03740902 |
| RNU6-1274P | PHTF1 | 0.295 | 0.03740902 |
| RNU6-1274P | TEX10 | 0.295 | 0.03740902 |
| RNU6-1274P | CEP83 | 0.295 | 0.03740902 |
| RNU6-1274P | RANBP2 | 0.295 | 0.03740902 |
| RNU6-1274P | TBC1D32 | 0.295 | 0.03740902 |
| RNU6-1274P | ORC3 | 0.295 | 0.03740902 |
| RNU6-1274P | ZNF770 | 0.295 | 0.03740902 |
| RNU6-1274P | MGA | 0.296 | 0.036782115 |
| RNU6-1274P | ZNF891 | 0.296 | 0.036782115 |
| RNU6-1274P | MRPS27 | 0.296 | 0.036782115 |
| RNU6-1274P | ZNF280C | 0.296 | 0.036782115 |
| RNU6-1274P | ASB11 | 0.296 | 0.036782115 |
| RNU6-1274P | NIP7 | 0.296 | 0.036782115 |
| RNU6-1274P | MBNL3 | 0.296 | 0.036782115 |
| RNU6-1274P | AHI1 | 0.296 | 0.036782115 |
| RNU6-1274P | RGPD4 | 0.296 | 0.036782115 |
| RNU6-1274P | CHST10 | 0.297 | 0.03616418 |
| RNU6-1274P | TOMM20L | 0.297 | 0.03616418 |
| RNU6-1274P | SLC9B2 | 0.297 | 0.03616418 |
| RNU6-1274P | NPAT | 0.298 | 0.035555117 |
| RNU6-1274P | HIVEP3 | 0.298 | 0.035555117 |
| RNU6-1274P | PRMT5 | 0.298 | 0.035555117 |
| RNU6-1274P | ZNF594 | 0.298 | 0.035555117 |
| RNU6-1274P | BANK1 | 0.298 | 0.035555117 |
| RNU6-1274P | POLR3H | 0.298 | 0.035555117 |
| RNU6-1274P | TBCK | 0.298 | 0.035555117 |
| RNU6-1274P | LAIR2 | 0.299 | 0.034954827 |
| RNU6-1274P | IFT80 | 0.299 | 0.034954827 |
| RNU6-1274P | ALDH6A1 | 0.299 | 0.034954827 |
| RNU6-1274P | FAM161A | 0.299 | 0.034954827 |
| RNU6-1274P | CDS2 | 0.299 | 0.034954827 |
| RNU6-1274P | ADK | 0.299 | 0.034954827 |
| RNU6-1274P | C11orf57 | 0.299 | 0.034954827 |
| RNU6-1274P | IL15RA | 0.299 | 0.034954827 |
| RNU6-1274P | FOXL2 | 0.299 | 0.034954827 |
| RNU6-1274P | UBTF | 0.3 | 0.034363212 |
| RNU6-1274P | ZMYM3 | 0.3 | 0.034363212 |
| RNU6-1274P | TRPC4 | 0.3 | 0.034363212 |
| RNU6-1274P | ZNF551 | 0.3 | 0.034363212 |
| RNU6-1274P | OR2G2 | 0.301 | 0.033780173 |
| RNU6-1274P | PIGW | 0.301 | 0.033780173 |
| RNU6-1274P | CSN1S1 | 0.301 | 0.033780173 |
| RNU6-1274P | G6PC | 0.301 | 0.033780173 |
| RNU6-1274P | SLC5A5 | 0.301 | 0.033780173 |
| RNU6-1274P | SIK2 | 0.301 | 0.033780173 |
| RNU6-1274P | GART | 0.301 | 0.033780173 |
| RNU6-1274P | CD2AP | 0.301 | 0.033780173 |
| RNU6-1274P | 6-Sep | 0.301 | 0.033780173 |
| RNU6-1274P | KLHL33 | 0.301 | 0.033780173 |
| RNU6-1274P | ZNF334 | 0.301 | 0.033780173 |
| RNU6-1274P | DFFA | 0.301 | 0.033780173 |
| RNU6-1274P | ACOX3 | 0.302 | 0.033205615 |
| RNU6-1274P | PRPF19 | 0.302 | 0.033205615 |
| RNU6-1274P | AC015688.5 | 0.302 | 0.033205615 |
| RNU6-1274P | ZNHIT6 | 0.302 | 0.033205615 |
| RNU6-1274P | LARP4 | 0.302 | 0.033205615 |
| RNU6-1274P | LGALS3BP | 0.302 | 0.033205615 |
| RNU6-1274P | CASS4 | 0.302 | 0.033205615 |
| RNU6-1274P | CACNG6 | 0.302 | 0.033205615 |
| RNU6-1274P | PEX26 | 0.302 | 0.033205615 |
| RNU6-1274P | TIGD1 | 0.303 | 0.03263944 |
| RNU6-1274P | AK3 | 0.303 | 0.03263944 |
| RNU6-1274P | TDRD1 | 0.303 | 0.03263944 |
| RNU6-1274P | LRTM2 | 0.303 | 0.03263944 |
| RNU6-1274P | CCNG1 | 0.303 | 0.03263944 |
| RNU6-1274P | ULK4 | 0.304 | 0.032081553 |
| RNU6-1274P | UTP20 | 0.304 | 0.032081553 |
| RNU6-1274P | THAP12 | 0.304 | 0.032081553 |
| RNU6-1274P | TIGD2 | 0.304 | 0.032081553 |
| RNU6-1274P | GSPT1 | 0.304 | 0.032081553 |
| RNU6-1274P | NUDT21 | 0.305 | 0.03153186 |
| RNU6-1274P | MAPRE2 | 0.305 | 0.03153186 |
| RNU6-1274P | E2F5 | 0.305 | 0.03153186 |
| RNU6-1274P | C17orf80 | 0.305 | 0.03153186 |
| RNU6-1274P | CD72 | 0.307 | 0.030456673 |
| RNU6-1274P | SMIM17 | 0.307 | 0.030456673 |
| RNU6-1274P | ZNF827 | 0.307 | 0.030456673 |
| RNU6-1274P | NFX1 | 0.308 | 0.029930993 |
| RNU6-1274P | PRKACB | 0.308 | 0.029930993 |
| RNU6-1274P | ZNF772 | 0.308 | 0.029930993 |
| RNU6-1274P | UBAP2L | 0.308 | 0.029930993 |
| RNU6-1274P | KIAA0586 | 0.308 | 0.029930993 |
| RNU6-1274P | COA4 | 0.308 | 0.029930993 |
| RNU6-1274P | STRBP | 0.308 | 0.029930993 |
| RNU6-1274P | KCNV2 | 0.308 | 0.029930993 |
| RNU6-1274P | GTF3C4 | 0.309 | 0.029413132 |
| RNU6-1274P | WDR4 | 0.309 | 0.029413132 |
| RNU6-1274P | NUDCD2 | 0.309 | 0.029413132 |
| RNU6-1274P | RBM45 | 0.31 | 0.028902997 |
| RNU6-1274P | CYP2A7 | 0.31 | 0.028902997 |
| RNU6-1274P | ADCY3 | 0.31 | 0.028902997 |
| RNU6-1274P | RPUSD4 | 0.31 | 0.028902997 |
| RNU6-1274P | ZNF888 | 0.311 | 0.028400497 |
| RNU6-1274P | CEP104 | 0.311 | 0.028400497 |
| RNU6-1274P | MSH2 | 0.311 | 0.028400497 |
| RNU6-1274P | KNG1 | 0.311 | 0.028400497 |
| RNU6-1274P | AC131097.2 | 0.311 | 0.028400497 |
| RNU6-1274P | FASTKD2 | 0.311 | 0.028400497 |
| RNU6-1274P | SKP2 | 0.312 | 0.027905542 |
| RNU6-1274P | MACF1 | 0.312 | 0.027905542 |
| RNU6-1274P | CCDC180 | 0.312 | 0.027905542 |
| RNU6-1274P | SMARCC2 | 0.312 | 0.027905542 |
| RNU6-1274P | TMEM163 | 0.312 | 0.027905542 |
| RNU6-1274P | SFXN2 | 0.313 | 0.027418042 |
| RNU6-1274P | ATXN7L3B | 0.313 | 0.027418042 |
| RNU6-1274P | MIPEP | 0.313 | 0.027418042 |
| RNU6-1274P | ERICH3 | 0.313 | 0.027418042 |
| RNU6-1274P | NKRF | 0.313 | 0.027418042 |
| RNU6-1274P | ZCCHC7 | 0.313 | 0.027418042 |
| RNU6-1274P | TMEM177 | 0.313 | 0.027418042 |
| RNU6-1274P | AGPAT5 | 0.313 | 0.027418042 |
| RNU6-1274P | WDR43 | 0.313 | 0.027418042 |
| RNU6-1274P | ABCE1 | 0.313 | 0.027418042 |
| RNU6-1274P | AGAP2 | 0.313 | 0.027418042 |
| RNU6-1274P | FASTKD1 | 0.314 | 0.026937906 |
| RNU6-1274P | IQCH | 0.314 | 0.026937906 |
| RNU6-1274P | NOLC1 | 0.314 | 0.026937906 |
| RNU6-1274P | DNAJA3 | 0.314 | 0.026937906 |
| RNU6-1274P | S100A1 | 0.314 | 0.026937906 |
| RNU6-1274P | DNMT3A | 0.314 | 0.026937906 |
| RNU6-1274P | ATP8A1 | 0.315 | 0.026465047 |
| RNU6-1274P | AC017083.4 | 0.315 | 0.026465047 |
| RNU6-1274P | PAICS | 0.316 | 0.025999376 |
| RNU6-1274P | VAV3 | 0.316 | 0.025999376 |
| RNU6-1274P | IVD | 0.316 | 0.025999376 |
| RNU6-1274P | C2orf49 | 0.316 | 0.025999376 |
| RNU6-1274P | HMG20A | 0.316 | 0.025999376 |
| RNU6-1274P | TMEM120B | 0.316 | 0.025999376 |
| RNU6-1274P | URB1 | 0.316 | 0.025999376 |
| RNU6-1274P | TFDP2 | 0.316 | 0.025999376 |
| RNU6-1274P | KRBOX4 | 0.317 | 0.025540806 |
| RNU6-1274P | POU2F3 | 0.317 | 0.025540806 |
| RNU6-1274P | PCP4L1 | 0.317 | 0.025540806 |
| RNU6-1274P | LYRM1 | 0.318 | 0.02508925 |
| RNU6-1274P | HEATR5B | 0.318 | 0.02508925 |
| RNU6-1274P | REXO5 | 0.318 | 0.02508925 |
| RNU6-1274P | RC3H2 | 0.318 | 0.02508925 |
| RNU6-1274P | ACSS1 | 0.319 | 0.024644622 |
| RNU6-1274P | SCML2 | 0.319 | 0.024644622 |
| RNU6-1274P | DNAAF4 | 0.319 | 0.024644622 |
| RNU6-1274P | MSANTD4 | 0.319 | 0.024644622 |
| RNU6-1274P | TCAF1 | 0.319 | 0.024644622 |
| RNU6-1274P | NOP14 | 0.319 | 0.024644622 |
| RNU6-1274P | WARS2 | 0.319 | 0.024644622 |
| RNU6-1274P | MTHFD1 | 0.319 | 0.024644622 |
| RNU6-1274P | HSF5 | 0.32 | 0.024206837 |
| RNU6-1274P | GRIK5 | 0.32 | 0.024206837 |
| RNU6-1274P | UBFD1 | 0.32 | 0.024206837 |
| RNU6-1274P | COG8 | 0.32 | 0.024206837 |
| RNU6-1274P | C5orf42 | 0.32 | 0.024206837 |
| RNU6-1274P | AL033529.1 | 0.32 | 0.024206837 |
| RNU6-1274P | PHLPP1 | 0.32 | 0.024206837 |
| RNU6-1274P | FAM185A | 0.32 | 0.024206837 |
| RNU6-1274P | ZCCHC11 | 0.32 | 0.024206837 |
| RNU6-1274P | REST | 0.32 | 0.024206837 |
| RNU6-1274P | NIPSNAP1 | 0.32 | 0.024206837 |
| RNU6-1274P | PPP2R1B | 0.321 | 0.023775809 |
| RNU6-1274P | WRNIP1 | 0.321 | 0.023775809 |
| RNU6-1274P | LPIN1 | 0.321 | 0.023775809 |
| RNU6-1274P | LRTOMT | 0.321 | 0.023775809 |
| RNU6-1274P | PLAC1 | 0.321 | 0.023775809 |
| RNU6-1274P | ZRANB3 | 0.321 | 0.023775809 |
| RNU6-1274P | CPXM1 | 0.322 | 0.023351455 |
| RNU6-1274P | PROB1 | 0.322 | 0.023351455 |
| RNU6-1274P | BACE1 | 0.322 | 0.023351455 |
| RNU6-1274P | GBP6 | 0.322 | 0.023351455 |
| RNU6-1274P | NAA35 | 0.322 | 0.023351455 |
| RNU6-1274P | ZNF584 | 0.322 | 0.023351455 |
| RNU6-1274P | SCARB1 | 0.322 | 0.023351455 |
| RNU6-1274P | FAM83B | 0.322 | 0.023351455 |
| RNU6-1274P | TREML2 | 0.322 | 0.023351455 |
| RNU6-1274P | LBHD1 | 0.322 | 0.023351455 |
| RNU6-1274P | RAI1 | 0.322 | 0.023351455 |
| RNU6-1274P | CNNM1 | 0.323 | 0.022933691 |
| RNU6-1274P | CARMIL2 | 0.323 | 0.022933691 |
| RNU6-1274P | ZC3HAV1L | 0.323 | 0.022933691 |
| RNU6-1274P | CBX5 | 0.323 | 0.022933691 |
| RNU6-1274P | FAM216A | 0.323 | 0.022933691 |
| RNU6-1274P | RBM20 | 0.324 | 0.022522435 |
| RNU6-1274P | GPAM | 0.324 | 0.022522435 |
| RNU6-1274P | OR11L1 | 0.324 | 0.022522435 |
| RNU6-1274P | KLHL23 | 0.324 | 0.022522435 |
| RNU6-1274P | TTC30B | 0.324 | 0.022522435 |
| RNU6-1274P | ATIC | 0.325 | 0.022117604 |
| RNU6-1274P | KBTBD6 | 0.325 | 0.022117604 |
| RNU6-1274P | MRE11 | 0.325 | 0.022117604 |
| RNU6-1274P | FARSB | 0.325 | 0.022117604 |
| RNU6-1274P | SMARCA5 | 0.326 | 0.021719117 |
| RNU6-1274P | MOCS3 | 0.326 | 0.021719117 |
| RNU6-1274P | LNX2 | 0.326 | 0.021719117 |
| RNU6-1274P | ZNF790 | 0.326 | 0.021719117 |
| RNU6-1274P | GSTCD | 0.326 | 0.021719117 |
| RNU6-1274P | SV2A | 0.326 | 0.021719117 |
| RNU6-1274P | THNSL1 | 0.326 | 0.021719117 |
| RNU6-1274P | STXBP5 | 0.326 | 0.021719117 |
| RNU6-1274P | DDB1 | 0.327 | 0.021326894 |
| RNU6-1274P | C11orf1 | 0.327 | 0.021326894 |
| RNU6-1274P | CCDC148 | 0.327 | 0.021326894 |
| RNU6-1274P | GTF3C1 | 0.327 | 0.021326894 |
| RNU6-1274P | GJC2 | 0.328 | 0.020940853 |
| RNU6-1274P | ZC3H7B | 0.328 | 0.020940853 |
| RNU6-1274P | SCN4A | 0.328 | 0.020940853 |
| RNU6-1274P | FAM196A | 0.328 | 0.020940853 |
| RNU6-1274P | ANGPT1 | 0.329 | 0.020560917 |
| RNU6-1274P | EARS2 | 0.329 | 0.020560917 |
| RNU6-1274P | ATAD1 | 0.329 | 0.020560917 |
| RNU6-1274P | SYNJ2 | 0.33 | 0.020187005 |
| RNU6-1274P | CLDN20 | 0.33 | 0.020187005 |
| RNU6-1274P | PHOSPHO2 | 0.33 | 0.020187005 |
| RNU6-1274P | C7orf72 | 0.331 | 0.019819041 |
| RNU6-1274P | OTUD4 | 0.331 | 0.019819041 |
| RNU6-1274P | GLS | 0.332 | 0.019456947 |
| RNU6-1274P | NUP153 | 0.332 | 0.019456947 |
| RNU6-1274P | REV3L | 0.332 | 0.019456947 |
| RNU6-1274P | DNAH6 | 0.332 | 0.019456947 |
| RNU6-1274P | PRMT3 | 0.332 | 0.019456947 |
| RNU6-1274P | ARHGEF9 | 0.333 | 0.019100646 |
| RNU6-1274P | ANGPTL6 | 0.333 | 0.019100646 |
| RNU6-1274P | CD34 | 0.333 | 0.019100646 |
| RNU6-1274P | HPDL | 0.334 | 0.018750062 |
| RNU6-1274P | WDR88 | 0.335 | 0.018405119 |
| RNU6-1274P | CYP2C8 | 0.335 | 0.018405119 |
| RNU6-1274P | MLH3 | 0.335 | 0.018405119 |
| RNU6-1274P | DCAF7 | 0.336 | 0.018065744 |
| RNU6-1274P | OR13D1 | 0.336 | 0.018065744 |
| RNU6-1274P | NLE1 | 0.336 | 0.018065744 |
| RNU6-1274P | ALKBH8 | 0.336 | 0.018065744 |
| RNU6-1274P | SMG7 | 0.337 | 0.017731863 |
| RNU6-1274P | HIBCH | 0.338 | 0.017403401 |
| RNU6-1274P | OR52H1 | 0.338 | 0.017403401 |
| RNU6-1274P | LRPPRC | 0.338 | 0.017403401 |
| RNU6-1274P | GATC | 0.338 | 0.017403401 |
| RNU6-1274P | CEP68 | 0.338 | 0.017403401 |
| RNU6-1274P | ANKEF1 | 0.339 | 0.017080287 |
| RNU6-1274P | KLF17 | 0.339 | 0.017080287 |
| RNU6-1274P | GNL3L | 0.339 | 0.017080287 |
| RNU6-1274P | WDR3 | 0.339 | 0.017080287 |
| RNU6-1274P | ZDHHC15 | 0.34 | 0.016762448 |
| RNU6-1274P | ADGRB1 | 0.34 | 0.016762448 |
| RNU6-1274P | MTR | 0.34 | 0.016762448 |
| RNU6-1274P | TARBP1 | 0.341 | 0.016449814 |
| RNU6-1274P | BAG5 | 0.341 | 0.016449814 |
| RNU6-1274P | ACSL5 | 0.342 | 0.016142313 |
| RNU6-1274P | TBC1D29 | 0.342 | 0.016142313 |
| RNU6-1274P | PDCD11 | 0.342 | 0.016142313 |
| RNU6-1274P | AIF1L | 0.342 | 0.016142313 |
| RNU6-1274P | RRP1B | 0.342 | 0.016142313 |
| RNU6-1274P | CLNS1A | 0.342 | 0.016142313 |
| RNU6-1274P | MAP7D2 | 0.342 | 0.016142313 |
| RNU6-1274P | SPATA24 | 0.343 | 0.015839876 |
| RNU6-1274P | GUF1 | 0.343 | 0.015839876 |
| RNU6-1274P | CSTF3 | 0.344 | 0.015542434 |
| RNU6-1274P | MMACHC | 0.344 | 0.015542434 |
| RNU6-1274P | RBM12 | 0.345 | 0.015249917 |
| RNU6-1274P | POLR3A | 0.345 | 0.015249917 |
| RNU6-1274P | CENPP | 0.345 | 0.015249917 |
| RNU6-1274P | DHTKD1 | 0.345 | 0.015249917 |
| RNU6-1274P | ZBTB26 | 0.345 | 0.015249917 |
| RNU6-1274P | PUS7 | 0.346 | 0.014962259 |
| RNU6-1274P | PARP11 | 0.346 | 0.014962259 |
| RNU6-1274P | LANCL1 | 0.346 | 0.014962259 |
| RNU6-1274P | SLX4IP | 0.346 | 0.014962259 |
| RNU6-1274P | FOXD4L5 | 0.346 | 0.014962259 |
| RNU6-1274P | ZNF320 | 0.346 | 0.014962259 |
| RNU6-1274P | RFX7 | 0.347 | 0.014679392 |
| RNU6-1274P | NFIX | 0.347 | 0.014679392 |
| RNU6-1274P | IPO11 | 0.348 | 0.014401249 |
| RNU6-1274P | FLRT1 | 0.348 | 0.014401249 |
| RNU6-1274P | CIPC | 0.349 | 0.014127765 |
| RNU6-1274P | TP53TG3D | 0.349 | 0.014127765 |
| RNU6-1274P | LARS2 | 0.349 | 0.014127765 |
| RNU6-1274P | NARS2 | 0.349 | 0.014127765 |
| RNU6-1274P | IPO5 | 0.349 | 0.014127765 |
| RNU6-1274P | PRPF8 | 0.35 | 0.013858874 |
| RNU6-1274P | TMEM25 | 0.35 | 0.013858874 |
| RNU6-1274P | METTL8 | 0.35 | 0.013858874 |
| RNU6-1274P | TMEM241 | 0.351 | 0.013594512 |
| RNU6-1274P | DLAT | 0.351 | 0.013594512 |
| RNU6-1274P | TERF2 | 0.351 | 0.013594512 |
| RNU6-1274P | LRRC34 | 0.352 | 0.013334615 |
| RNU6-1274P | DHX33 | 0.352 | 0.013334615 |
| RNU6-1274P | TGFBRAP1 | 0.353 | 0.01307912 |
| RNU6-1274P | SETDB2 | 0.354 | 0.012827963 |
| RNU6-1274P | TSR1 | 0.354 | 0.012827963 |
| RNU6-1274P | HUWE1 | 0.356 | 0.012338421 |
| RNU6-1274P | ZNF749 | 0.356 | 0.012338421 |
| RNU6-1274P | CKLF-CMTM1 | 0.356 | 0.012338421 |
| RNU6-1274P | PDLIM1 | 0.357 | 0.012099912 |
| RNU6-1274P | PFAS | 0.357 | 0.012099912 |
| RNU6-1274P | ADNP | 0.358 | 0.011865499 |
| RNU6-1274P | EXOSC2 | 0.358 | 0.011865499 |
| RNU6-1274P | TRMT61B | 0.359 | 0.011635122 |
| RNU6-1274P | GUCY1A3 | 0.359 | 0.011635122 |
| RNU6-1274P | METTL16 | 0.36 | 0.011408723 |
| RNU6-1274P | SH3D21 | 0.36 | 0.011408723 |
| RNU6-1274P | NEK1 | 0.36 | 0.011408723 |
| RNU6-1274P | ANGPT2 | 0.362 | 0.010967623 |
| RNU6-1274P | ROS1 | 0.362 | 0.010967623 |
| RNU6-1274P | SLC9A7 | 0.363 | 0.010752809 |
| RNU6-1274P | POLR1B | 0.364 | 0.010541744 |
| RNU6-1274P | FAM83G | 0.364 | 0.010541744 |
| RNU6-1274P | NUP214 | 0.365 | 0.010334372 |
| RNU6-1274P | PPAT | 0.365 | 0.010334372 |
| RNU6-1274P | TRUB2 | 0.365 | 0.010334372 |
| RNU6-1274P | SLC5A10 | 0.366 | 0.010130638 |
| RNU6-1274P | FIP1L1 | 0.367 | 0.009930488 |
| RNU6-1274P | ATP8B2 | 0.368 | 0.009733869 |
| RNU6-1274P | INTS7 | 0.368 | 0.009733869 |
| RNU6-1274P | ZNF496 | 0.368 | 0.009733869 |
| RNU6-1274P | PANK1 | 0.369 | 0.009540727 |
| RNU6-1274P | ASAP2 | 0.369 | 0.009540727 |
| RNU6-1274P | GTF2I | 0.369 | 0.009540727 |
| RNU6-1274P | IGSF10 | 0.369 | 0.009540727 |
| RNU6-1274P | CYP2C18 | 0.369 | 0.009540727 |
| RNU6-1274P | XPO5 | 0.37 | 0.009351009 |
| RNU6-1274P | TAF1D | 0.371 | 0.009164665 |
| RNU6-1274P | ZNF33B | 0.371 | 0.009164665 |
| RNU6-1274P | FKTN | 0.372 | 0.008981642 |
| RNU6-1274P | BTBD3 | 0.372 | 0.008981642 |
| RNU6-1274P | ANKRD17 | 0.373 | 0.008801891 |
| RNU6-1274P | FOXR1 | 0.374 | 0.00862536 |
| RNU6-1274P | SYNGAP1 | 0.374 | 0.00862536 |
| RNU6-1274P | TTC5 | 0.374 | 0.00862536 |
| RNU6-1274P | C2CD3 | 0.374 | 0.00862536 |
| RNU6-1274P | FAM120C | 0.375 | 0.008452001 |
| RNU6-1274P | CAD | 0.375 | 0.008452001 |
| RNU6-1274P | DZIP3 | 0.376 | 0.008281765 |
| RNU6-1274P | RGP1 | 0.377 | 0.008114602 |
| RNU6-1274P | C10orf128 | 0.377 | 0.008114602 |
| RNU6-1274P | USP10 | 0.378 | 0.007950467 |
| RNU6-1274P | TMEM135 | 0.378 | 0.007950467 |
| RNU6-1274P | UNC119B | 0.378 | 0.007950467 |
| RNU6-1274P | SULT1E1 | 0.378 | 0.007950467 |
| RNU6-1274P | ACTR3C | 0.379 | 0.007789311 |
| RNU6-1274P | PALB2 | 0.379 | 0.007789311 |
| RNU6-1274P | SPRYD4 | 0.379 | 0.007789311 |
| RNU6-1274P | ZPBP | 0.38 | 0.007631089 |
| RNU6-1274P | RPP40 | 0.381 | 0.007475753 |
| RNU6-1274P | PRDM16 | 0.381 | 0.007475753 |
| RNU6-1274P | SPAG16 | 0.382 | 0.007323259 |
| RNU6-1274P | TMEM156 | 0.383 | 0.007173561 |
| RNU6-1274P | CACNG8 | 0.384 | 0.007026617 |
| RNU6-1274P | HEATR1 | 0.386 | 0.00674081 |
| RNU6-1274P | RNF214 | 0.388 | 0.006465495 |
| RNU6-1274P | CMTM1 | 0.388 | 0.006465495 |
| RNU6-1274P | URB2 | 0.388 | 0.006465495 |
| RNU6-1274P | KCNIP1 | 0.39 | 0.006200338 |
| RNU6-1274P | FANCF | 0.39 | 0.006200338 |
| RNU6-1274P | DROSHA | 0.391 | 0.006071467 |
| RNU6-1274P | DOCK1 | 0.392 | 0.005945013 |
| RNU6-1274P | LDLRAD2 | 0.393 | 0.005820937 |
| RNU6-1274P | ARHGEF5 | 0.393 | 0.005820937 |
| RNU6-1274P | HERC2 | 0.393 | 0.005820937 |
| RNU6-1274P | AMER1 | 0.393 | 0.005820937 |
| RNU6-1274P | DDX10 | 0.394 | 0.005699201 |
| RNU6-1274P | LIPC | 0.394 | 0.005699201 |
| RNU6-1274P | FOXL2NB | 0.395 | 0.005579766 |
| RNU6-1274P | L2HGDH | 0.396 | 0.005462594 |
| RNU6-1274P | CYP2C9 | 0.396 | 0.005462594 |
| RNU6-1274P | ADGRG5 | 0.398 | 0.00523489 |
| RNU6-1274P | PHGR1 | 0.398 | 0.00523489 |
| RNU6-1274P | KMT2A | 0.398 | 0.00523489 |
| RNU6-1274P | MARS2 | 0.399 | 0.005124284 |
| RNU6-1274P | UBIAD1 | 0.4 | 0.005015795 |
| RNU6-1274P | A2ML1 | 0.401 | 0.004909387 |
| RNU6-1274P | UBAP2 | 0.401 | 0.004909387 |
| RNU6-1274P | AC090360.1 | 0.401 | 0.004909387 |
| RNU6-1274P | PAAF1 | 0.401 | 0.004909387 |
| RNU6-1274P | FAM208B | 0.402 | 0.004805025 |
| RNU6-1274P | ZNF70 | 0.403 | 0.004702675 |
| RNU6-1274P | DISP2 | 0.405 | 0.004503873 |
| RNU6-1274P | LRBA | 0.407 | 0.004312717 |
| RNU6-1274P | EP400 | 0.407 | 0.004312717 |
| RNU6-1274P | ALG9 | 0.409 | 0.004128945 |
| RNU6-1274P | TWNK | 0.409 | 0.004128945 |
| RNU6-1274P | ARRB1 | 0.41 | 0.00403975 |
| RNU6-1274P | DDX31 | 0.412 | 0.003866588 |
| RNU6-1274P | AP002360.1 | 0.412 | 0.003866588 |
| RNU6-1274P | TMEM217 | 0.412 | 0.003866588 |
| RNU6-1274P | FTCDNL1 | 0.414 | 0.003700195 |
| RNU6-1274P | DNMT3B | 0.414 | 0.003700195 |
| RNU6-1274P | AP002495.2 | 0.416 | 0.003540337 |
| RNU6-1274P | TFB1M | 0.417 | 0.003462788 |
| RNU6-1274P | NME7 | 0.42 | 0.003239326 |
| RNU6-1274P | SHANK2 | 0.423 | 0.003029079 |
| RNU6-1274P | STN1 | 0.424 | 0.002961812 |
| RNU6-1274P | CCDC113 | 0.426 | 0.002831351 |
| RNU6-1274P | MDN1 | 0.428 | 0.002706157 |
| RNU6-1274P | KDM4D | 0.429 | 0.002645476 |
| RNU6-1274P | MACC1 | 0.431 | 0.002527829 |
| RNU6-1274P | ANGEL1 | 0.438 | 0.002152757 |
| RNU6-1274P | PARP1 | 0.44 | 0.00205538 |
| RNU6-1274P | CNST | 0.441 | 0.002008223 |
| RNU6-1274P | S100Z | 0.443 | 0.001916874 |
| RNU6-1274P | KCNQ2 | 0.443 | 0.001916874 |
| RNU6-1274P | HESX1 | 0.444 | 0.001872644 |
| RNU6-1274P | MCCC2 | 0.445 | 0.001829354 |
| RNU6-1274P | ZMIZ1 | 0.446 | 0.001786985 |
| RNU6-1274P | SLC7A6 | 0.449 | 0.001665232 |
| RNU6-1274P | NR6A1 | 0.449 | 0.001665232 |
| RNU6-1274P | SLC52A3 | 0.451 | 0.001588354 |
| RNU6-1274P | IFT46 | 0.454 | 0.001479147 |
| RNU6-1274P | MPI | 0.454 | 0.001479147 |
| RNU6-1274P | KLLN | 0.455 | 0.001444311 |
| RNU6-1274P | ANAPC1 | 0.457 | 0.001376896 |
| RNU6-1274P | R3HDM1 | 0.459 | 0.001312394 |
| RNU6-1274P | POLR1A | 0.46 | 0.0012812 |
| RNU6-1274P | FAM86C1 | 0.462 | 0.001220854 |
| RNU6-1274P | ABCC1 | 0.469 | 0.001029717 |
| RNU6-1274P | ACACA | 0.478 | 0.000824607 |
| RNU6-1274P | YLPM1 | 0.479 | 0.000804324 |
| RNU6-1274P | ACSM6 | 0.479 | 0.000804324 |
| RNU6-1274P | LCT | 0.488 | 0.000641522 |
| RNU6-1274P | ALDH5A1 | 0.495 | 0.000536696 |
| RNU6-1274P | SCFD2 | 0.515 | 0.000318478 |
| RNU6-1274P | NAT10 | 0.524 | 0.000250344 |
| RNU6-1274P | CPT1A | 0.536 | 0.000180584 |
| RNU6-1274P | RNU6-1274P | 1 | 3.72E-12 |
| RNU6-1272P | RPS6KA1 | -0.544 | 0.001684242 |
| RNU6-1272P | IRAK1 | -0.496 | 0.004216779 |
| RNU6-1272P | TRIM8 | -0.481 | 0.005534637 |
| RNU6-1272P | VAV1 | -0.475 | 0.006158531 |
| RNU6-1272P | CCND3 | -0.469 | 0.006845061 |
| RNU6-1272P | SIPA1 | -0.461 | 0.007867197 |
| RNU6-1272P | ABCA7 | -0.452 | 0.009178783 |
| RNU6-1272P | BLOC1S1 | -0.452 | 0.009178783 |
| RNU6-1272P | ADCY7 | -0.451 | 0.009335945 |
| RNU6-1272P | RMND5B | -0.447 | 0.009988867 |
| RNU6-1272P | SH2D3C | -0.444 | 0.010504935 |
| RNU6-1272P | SH3BP2 | -0.443 | 0.010682155 |
| RNU6-1272P | ARHGEF1 | -0.442 | 0.010862028 |
| RNU6-1272P | UCK1 | -0.44 | 0.011229869 |
| RNU6-1272P | TFEB | -0.439 | 0.011417906 |
| RNU6-1272P | SH3BP1 | -0.439 | 0.011417906 |
| RNU6-1272P | SELPLG | -0.436 | 0.011998892 |
| RNU6-1272P | LIMK1 | -0.436 | 0.011998892 |
| RNU6-1272P | PREX1 | -0.43 | 0.013240011 |
| RNU6-1272P | PSMD2 | -0.429 | 0.013457549 |
| RNU6-1272P | SIRT2 | -0.427 | 0.013902122 |
| RNU6-1272P | CYTH4 | -0.426 | 0.014129232 |
| RNU6-1272P | NTMT1 | -0.424 | 0.014593295 |
| RNU6-1272P | ITPK1 | -0.422 | 0.015070744 |
| RNU6-1272P | TEX261 | -0.422 | 0.015070744 |
| RNU6-1272P | FAM53B | -0.422 | 0.015070744 |
| RNU6-1272P | PLBD2 | -0.422 | 0.015070744 |
| RNU6-1272P | FIBP | -0.422 | 0.015070744 |
| RNU6-1272P | IKBKG | -0.42 | 0.015561897 |
| RNU6-1272P | CSK | -0.419 | 0.015812715 |
| RNU6-1272P | PRELID1 | -0.419 | 0.015812715 |
| RNU6-1272P | LSM4 | -0.417 | 0.016325037 |
| RNU6-1272P | TECPR1 | -0.416 | 0.016586625 |
| RNU6-1272P | IQCE | -0.416 | 0.016586625 |
| RNU6-1272P | RAPGEF1 | -0.414 | 0.017120865 |
| RNU6-1272P | LSP1 | -0.413 | 0.017393602 |
| RNU6-1272P | ARHGAP45 | -0.412 | 0.017670142 |
| RNU6-1272P | WRAP73 | -0.41 | 0.018234803 |
| RNU6-1272P | IQSEC1 | -0.41 | 0.018234803 |
| RNU6-1272P | LTB4R | -0.41 | 0.018234803 |
| RNU6-1272P | FERMT3 | -0.409 | 0.018523013 |
| RNU6-1272P | EHBP1L1 | -0.408 | 0.018815201 |
| RNU6-1272P | RHBDF2 | -0.408 | 0.018815201 |
| RNU6-1272P | FLNA | -0.405 | 0.019716086 |
| RNU6-1272P | UNC93B1 | -0.405 | 0.019716086 |
| RNU6-1272P | HCLS1 | -0.404 | 0.020024641 |
| RNU6-1272P | SASH3 | -0.404 | 0.020024641 |
| RNU6-1272P | ETFB | -0.404 | 0.020024641 |
| RNU6-1272P | NCOR2 | -0.403 | 0.020337401 |
| RNU6-1272P | ITGB2 | -0.403 | 0.020337401 |
| RNU6-1272P | RABGGTA | -0.402 | 0.020654415 |
| RNU6-1272P | LAPTM5 | -0.402 | 0.020654415 |
| RNU6-1272P | SNX8 | -0.402 | 0.020654415 |
| RNU6-1272P | NUDT22 | -0.401 | 0.020975728 |
| RNU6-1272P | STK10 | -0.401 | 0.020975728 |
| RNU6-1272P | NAPA | -0.4 | 0.021301389 |
| RNU6-1272P | CCDC22 | -0.4 | 0.021301389 |
| RNU6-1272P | SCYL1 | -0.399 | 0.021631444 |
| RNU6-1272P | GPR132 | -0.399 | 0.021631444 |
| RNU6-1272P | BATF | -0.399 | 0.021631444 |
| RNU6-1272P | PLEKHJ1 | -0.399 | 0.021631444 |
| RNU6-1272P | C6orf89 | -0.398 | 0.021965942 |
| RNU6-1272P | PEPD | -0.398 | 0.021965942 |
| RNU6-1272P | PPARD | -0.398 | 0.021965942 |
| RNU6-1272P | RIPK3 | -0.397 | 0.022304931 |
| RNU6-1272P | ATP13A2 | -0.397 | 0.022304931 |
| RNU6-1272P | CD300A | -0.396 | 0.02264846 |
| RNU6-1272P | SLC8B1 | -0.395 | 0.022996578 |
| RNU6-1272P | GPR108 | -0.395 | 0.022996578 |
| RNU6-1272P | C15orf39 | -0.395 | 0.022996578 |
| RNU6-1272P | TOR2A | -0.395 | 0.022996578 |
| RNU6-1272P | PLXNB2 | -0.394 | 0.023349334 |
| RNU6-1272P | ARHGAP27 | -0.393 | 0.023706777 |
| RNU6-1272P | PLEKHM1 | -0.393 | 0.023706777 |
| RNU6-1272P | PI4KB | -0.393 | 0.023706777 |
| RNU6-1272P | PRKCD | -0.392 | 0.024068958 |
| RNU6-1272P | ABR | -0.392 | 0.024068958 |
| RNU6-1272P | FBXW5 | -0.392 | 0.024068958 |
| RNU6-1272P | MTCH1 | -0.392 | 0.024068958 |
| RNU6-1272P | RNPEPL1 | -0.391 | 0.024435928 |
| RNU6-1272P | SLC22A18 | -0.391 | 0.024435928 |
| RNU6-1272P | CYB561A3 | -0.39 | 0.024807736 |
| RNU6-1272P | POLR2G | -0.39 | 0.024807736 |
| RNU6-1272P | MIEN1 | -0.389 | 0.025184434 |
| RNU6-1272P | RASA3 | -0.389 | 0.025184434 |
| RNU6-1272P | TK2 | -0.389 | 0.025184434 |
| RNU6-1272P | TFE3 | -0.388 | 0.025566073 |
| RNU6-1272P | MED27 | -0.388 | 0.025566073 |
| RNU6-1272P | DHRS4L2 | -0.388 | 0.025566073 |
| RNU6-1272P | TUBG2 | -0.388 | 0.025566073 |
| RNU6-1272P | EVI5L | -0.387 | 0.025952706 |
| RNU6-1272P | VSIR | -0.386 | 0.026344383 |
| RNU6-1272P | POLR2E | -0.386 | 0.026344383 |
| RNU6-1272P | ZNF335 | -0.386 | 0.026344383 |
| RNU6-1272P | LSM10 | -0.386 | 0.026344383 |
| RNU6-1272P | KCNQ1 | -0.385 | 0.026741158 |
| RNU6-1272P | STX16-NPEPL1 | -0.385 | 0.026741158 |
| RNU6-1272P | G6PD | -0.385 | 0.026741158 |
| RNU6-1272P | DHX16 | -0.384 | 0.027143082 |
| RNU6-1272P | C7orf50 | -0.383 | 0.02755021 |
| RNU6-1272P | TLN1 | -0.383 | 0.02755021 |
| RNU6-1272P | SH3GLB2 | -0.382 | 0.027962595 |
| RNU6-1272P | WDR1 | -0.382 | 0.027962595 |
| RNU6-1272P | ABHD11 | -0.382 | 0.027962595 |
| RNU6-1272P | SH3TC1 | -0.382 | 0.027962595 |
| RNU6-1272P | SMPD1 | -0.382 | 0.027962595 |
| RNU6-1272P | EPN1 | -0.381 | 0.028380289 |
| RNU6-1272P | OAZ1 | -0.381 | 0.028380289 |
| RNU6-1272P | MAPKAP1 | -0.381 | 0.028380289 |
| RNU6-1272P | CCDC88B | -0.38 | 0.028803348 |
| RNU6-1272P | S100A6 | -0.38 | 0.028803348 |
| RNU6-1272P | CISD3 | -0.38 | 0.028803348 |
| RNU6-1272P | RAB24 | -0.38 | 0.028803348 |
| RNU6-1272P | EFHD2 | -0.379 | 0.029231826 |
| RNU6-1272P | FGD3 | -0.379 | 0.029231826 |
| RNU6-1272P | ARAP1 | -0.379 | 0.029231826 |
| RNU6-1272P | BRAT1 | -0.379 | 0.029231826 |
| RNU6-1272P | PDLIM2 | -0.379 | 0.029231826 |
| RNU6-1272P | TADA3 | -0.379 | 0.029231826 |
| RNU6-1272P | RGS14 | -0.379 | 0.029231826 |
| RNU6-1272P | MCRS1 | -0.378 | 0.029665777 |
| RNU6-1272P | CREB3 | -0.378 | 0.029665777 |
| RNU6-1272P | STK16 | -0.377 | 0.030105256 |
| RNU6-1272P | BCKDK | -0.377 | 0.030105256 |
| RNU6-1272P | TRIM62 | -0.377 | 0.030105256 |
| RNU6-1272P | CAMTA2 | -0.376 | 0.03055032 |
| RNU6-1272P | KRI1 | -0.375 | 0.031001022 |
| RNU6-1272P | HSD17B10 | -0.375 | 0.031001022 |
| RNU6-1272P | FBXW4 | -0.375 | 0.031001022 |
| RNU6-1272P | PORCN | -0.374 | 0.03145742 |
| RNU6-1272P | RUBCN | -0.374 | 0.03145742 |
| RNU6-1272P | GRK6 | -0.374 | 0.03145742 |
| RNU6-1272P | PEF1 | -0.374 | 0.03145742 |
| RNU6-1272P | WAS | -0.374 | 0.03145742 |
| RNU6-1272P | DGKQ | -0.373 | 0.031919569 |
| RNU6-1272P | SNRNP25 | -0.373 | 0.031919569 |
| RNU6-1272P | TWF2 | -0.373 | 0.031919569 |
| RNU6-1272P | RANGAP1 | -0.372 | 0.032387527 |
| RNU6-1272P | TRABD | -0.372 | 0.032387527 |
| RNU6-1272P | RER1 | -0.371 | 0.03286135 |
| RNU6-1272P | BLVRB | -0.371 | 0.03286135 |
| RNU6-1272P | TMEM229B | -0.371 | 0.03286135 |
| RNU6-1272P | RXRA | -0.371 | 0.03286135 |
| RNU6-1272P | NKG7 | -0.37 | 0.033341096 |
| RNU6-1272P | PSD4 | -0.37 | 0.033341096 |
| RNU6-1272P | SMAD3 | -0.369 | 0.033826822 |
| RNU6-1272P | TMEM80 | -0.369 | 0.033826822 |
| RNU6-1272P | MCOLN1 | -0.369 | 0.033826822 |
| RNU6-1272P | E2F4 | -0.368 | 0.034318585 |
| RNU6-1272P | MICAL1 | -0.368 | 0.034318585 |
| RNU6-1272P | ACTN4 | -0.367 | 0.034816445 |
| RNU6-1272P | CLCN5 | -0.367 | 0.034816445 |
| RNU6-1272P | FAM96B | -0.367 | 0.034816445 |
| RNU6-1272P | HPS1 | -0.366 | 0.03532046 |
| RNU6-1272P | ITGAL | -0.366 | 0.03532046 |
| RNU6-1272P | TNFAIP2 | -0.366 | 0.03532046 |
| RNU6-1272P | PANK4 | -0.366 | 0.03532046 |
| RNU6-1272P | TSPAN17 | -0.366 | 0.03532046 |
| RNU6-1272P | NPRL2 | -0.366 | 0.03532046 |
| RNU6-1272P | TNNI2 | -0.366 | 0.03532046 |
| RNU6-1272P | PUSL1 | -0.366 | 0.03532046 |
| RNU6-1272P | MED29 | -0.365 | 0.035830687 |
| RNU6-1272P | NBEAL2 | -0.365 | 0.035830687 |
| RNU6-1272P | AP3D1 | -0.365 | 0.035830687 |
| RNU6-1272P | SPRYD3 | -0.364 | 0.036347188 |
| RNU6-1272P | CDKN2D | -0.364 | 0.036347188 |
| RNU6-1272P | GMIP | -0.364 | 0.036347188 |
| RNU6-1272P | TCTA | -0.364 | 0.036347188 |
| RNU6-1272P | TRADD | -0.363 | 0.03687002 |
| RNU6-1272P | KLF13 | -0.363 | 0.03687002 |
| RNU6-1272P | DNASE1L1 | -0.363 | 0.03687002 |
| RNU6-1272P | GBA2 | -0.363 | 0.03687002 |
| RNU6-1272P | PSMF1 | -0.363 | 0.03687002 |
| RNU6-1272P | SLC43A2 | -0.363 | 0.03687002 |
| RNU6-1272P | CTSD | -0.362 | 0.037399244 |
| RNU6-1272P | BAK1 | -0.362 | 0.037399244 |
| RNU6-1272P | CRAT | -0.362 | 0.037399244 |
| RNU6-1272P | CPQ | -0.361 | 0.037934919 |
| RNU6-1272P | IRF2BPL | -0.361 | 0.037934919 |
| RNU6-1272P | CARS | -0.36 | 0.038477106 |
| RNU6-1272P | FAM89B | -0.36 | 0.038477106 |
| RNU6-1272P | TBC1D22A | -0.36 | 0.038477106 |
| RNU6-1272P | MAP3K10 | -0.359 | 0.039025866 |
| RNU6-1272P | STAT6 | -0.359 | 0.039025866 |
| RNU6-1272P | POR | -0.358 | 0.039581259 |
| RNU6-1272P | FAAP100 | -0.358 | 0.039581259 |
| RNU6-1272P | AP2A1 | -0.358 | 0.039581259 |
| RNU6-1272P | RAB1B | -0.358 | 0.039581259 |
| RNU6-1272P | KIF1C | -0.358 | 0.039581259 |
| RNU6-1272P | DCTN1 | -0.357 | 0.040143346 |
| RNU6-1272P | FTH1 | -0.357 | 0.040143346 |
| RNU6-1272P | STUB1 | -0.357 | 0.040143346 |
| RNU6-1272P | PMM1 | -0.357 | 0.040143346 |
| RNU6-1272P | TYK2 | -0.357 | 0.040143346 |
| RNU6-1272P | JOSD2 | -0.357 | 0.040143346 |
| RNU6-1272P | CHMP2A | -0.357 | 0.040143346 |
| RNU6-1272P | PARP10 | -0.356 | 0.040712189 |
| RNU6-1272P | KCTD17 | -0.356 | 0.040712189 |
| RNU6-1272P | PPP6R2 | -0.356 | 0.040712189 |
| RNU6-1272P | WDFY4 | -0.356 | 0.040712189 |
| RNU6-1272P | GPKOW | -0.356 | 0.040712189 |
| RNU6-1272P | SIRT6 | -0.356 | 0.040712189 |
| RNU6-1272P | PCNX3 | -0.355 | 0.041287849 |
| RNU6-1272P | LAT2 | -0.355 | 0.041287849 |
| RNU6-1272P | PSMB8 | -0.355 | 0.041287849 |
| RNU6-1272P | LHPP | -0.355 | 0.041287849 |
| RNU6-1272P | ST6GALNAC4 | -0.355 | 0.041287849 |
| RNU6-1272P | PSMB3 | -0.354 | 0.041870389 |
| RNU6-1272P | PPM1M | -0.354 | 0.041870389 |
| RNU6-1272P | UPF1 | -0.354 | 0.041870389 |
| RNU6-1272P | TSEN34 | -0.354 | 0.041870389 |
| RNU6-1272P | OGFR | -0.354 | 0.041870389 |
| RNU6-1272P | ATG2A | -0.353 | 0.042459871 |
| RNU6-1272P | ABHD8 | -0.353 | 0.042459871 |
| RNU6-1272P | VPS16 | -0.353 | 0.042459871 |
| RNU6-1272P | MVP | -0.353 | 0.042459871 |
| RNU6-1272P | MFSD7 | -0.353 | 0.042459871 |
| RNU6-1272P | CNPY3 | -0.353 | 0.042459871 |
| RNU6-1272P | DBNL | -0.353 | 0.042459871 |
| RNU6-1272P | TSPAN14 | -0.352 | 0.043056357 |
| RNU6-1272P | EXTL3 | -0.352 | 0.043056357 |
| RNU6-1272P | C11orf21 | -0.352 | 0.043056357 |
| RNU6-1272P | RNF187 | -0.352 | 0.043056357 |
| RNU6-1272P | ARHGAP30 | -0.352 | 0.043056357 |
| RNU6-1272P | GMPPA | -0.352 | 0.043056357 |
| RNU6-1272P | FAM50A | -0.352 | 0.043056357 |
| RNU6-1272P | C9orf142 | -0.352 | 0.043056357 |
| RNU6-1272P | TINF2 | -0.351 | 0.043659909 |
| RNU6-1272P | PI4KA | -0.351 | 0.043659909 |
| RNU6-1272P | FGR | -0.351 | 0.043659909 |
| RNU6-1272P | HINT2 | -0.351 | 0.043659909 |
| RNU6-1272P | MAP3K14 | -0.351 | 0.043659909 |
| RNU6-1272P | KIAA0513 | -0.35 | 0.044270592 |
| RNU6-1272P | NDUFB10 | -0.35 | 0.044270592 |
| RNU6-1272P | SURF1 | -0.35 | 0.044270592 |
| RNU6-1272P | SRP14 | -0.35 | 0.044270592 |
| RNU6-1272P | CYB561D2 | -0.35 | 0.044270592 |
| RNU6-1272P | PTPN1 | -0.349 | 0.044888468 |
| RNU6-1272P | RGS19 | -0.349 | 0.044888468 |
| RNU6-1272P | CDH23 | -0.349 | 0.044888468 |
| RNU6-1272P | PNPLA6 | -0.349 | 0.044888468 |
| RNU6-1272P | COPS7A | -0.348 | 0.045513601 |
| RNU6-1272P | RAC2 | -0.348 | 0.045513601 |
| RNU6-1272P | SEMA4D | -0.348 | 0.045513601 |
| RNU6-1272P | PLEC | -0.348 | 0.045513601 |
| RNU6-1272P | EME2 | -0.347 | 0.046146054 |
| RNU6-1272P | AP2M1 | -0.347 | 0.046146054 |
| RNU6-1272P | PARVG | -0.347 | 0.046146054 |
| RNU6-1272P | RIPOR1 | -0.347 | 0.046146054 |
| RNU6-1272P | DENND4B | -0.347 | 0.046146054 |
| RNU6-1272P | ADCK1 | -0.347 | 0.046146054 |
| RNU6-1272P | CNOT3 | -0.346 | 0.046785892 |
| RNU6-1272P | PLCB2 | -0.346 | 0.046785892 |
| RNU6-1272P | PSMC4 | -0.346 | 0.046785892 |
| RNU6-1272P | THBD | -0.346 | 0.046785892 |
| RNU6-1272P | RINL | -0.346 | 0.046785892 |
| RNU6-1272P | TOMM40L | -0.346 | 0.046785892 |
| RNU6-1272P | EML3 | -0.345 | 0.047433179 |
| RNU6-1272P | FAM219A | -0.345 | 0.047433179 |
| RNU6-1272P | MYO9B | -0.345 | 0.047433179 |
| RNU6-1272P | CDK9 | -0.345 | 0.047433179 |
| RNU6-1272P | SLC43A3 | -0.345 | 0.047433179 |
| RNU6-1272P | DNAJB2 | -0.345 | 0.047433179 |
| RNU6-1272P | SDHA | -0.345 | 0.047433179 |
| RNU6-1272P | DNAJB12 | -0.345 | 0.047433179 |
| RNU6-1272P | CLPTM1 | -0.345 | 0.047433179 |
| RNU6-1272P | SBNO2 | -0.344 | 0.04808798 |
| RNU6-1272P | PEA15 | -0.344 | 0.04808798 |
| RNU6-1272P | EIF2B1 | -0.344 | 0.04808798 |
| RNU6-1272P | CXorf40A | -0.344 | 0.04808798 |
| RNU6-1272P | INPP4A | -0.344 | 0.04808798 |
| RNU6-1272P | NDUFS2 | -0.344 | 0.04808798 |
| RNU6-1272P | TSSC4 | -0.344 | 0.04808798 |
| RNU6-1272P | CAPN10 | -0.343 | 0.048750358 |
| RNU6-1272P | PLOD1 | -0.343 | 0.048750358 |
| RNU6-1272P | PSMD13 | -0.343 | 0.048750358 |
| RNU6-1272P | ABHD17A | -0.343 | 0.048750358 |
| RNU6-1272P | LFNG | -0.343 | 0.048750358 |
| RNU6-1272P | PARP3 | -0.343 | 0.048750358 |
| RNU6-1272P | ANAPC11 | -0.343 | 0.048750358 |
| RNU6-1272P | FBXO44 | -0.343 | 0.048750358 |
| RNU6-1272P | COX14 | -0.343 | 0.048750358 |
| RNU6-1272P | PSMD8 | -0.343 | 0.048750358 |
| RNU6-1272P | MRPL28 | -0.342 | 0.04942038 |
| RNU6-1272P | TOLLIP | -0.342 | 0.04942038 |
| RNU6-1272P | CHD3 | -0.342 | 0.04942038 |
| RNU6-1272P | SARS | -0.342 | 0.04942038 |
| RNU6-1272P | DHRS4 | -0.342 | 0.04942038 |
| RNU6-1272P | GDI1 | -0.342 | 0.04942038 |
| RNU6-1272P | SELENOO | -0.342 | 0.04942038 |
| RNU6-1272P | PACS2 | -0.342 | 0.04942038 |
| RNU6-1272P | PLPPR2 | -0.342 | 0.04942038 |
| RNU6-1272P | INO80D | 0.333 | 0.049457065 |
| RNU6-1272P | ZNF182 | 0.333 | 0.049457065 |
| RNU6-1272P | ZNF852 | 0.333 | 0.049457065 |
| RNU6-1272P | DPY19L4 | 0.333 | 0.049457065 |
| RNU6-1272P | FAM120C | 0.333 | 0.049457065 |
| RNU6-1272P | ZNF700 | 0.333 | 0.049457065 |
| RNU6-1272P | ZNF836 | 0.334 | 0.048786626 |
| RNU6-1272P | TOMM20L | 0.334 | 0.048786626 |
| RNU6-1272P | MTM1 | 0.334 | 0.048786626 |
| RNU6-1272P | CDK8 | 0.334 | 0.048786626 |
| RNU6-1272P | ZNF461 | 0.334 | 0.048786626 |
| RNU6-1272P | ZNF436 | 0.334 | 0.048786626 |
| RNU6-1272P | TRAF3IP2 | 0.335 | 0.048123833 |
| RNU6-1272P | RC3H1 | 0.335 | 0.048123833 |
| RNU6-1272P | TCERG1 | 0.335 | 0.048123833 |
| RNU6-1272P | ZNF713 | 0.336 | 0.047468621 |
| RNU6-1272P | ZNF493 | 0.336 | 0.047468621 |
| RNU6-1272P | TYW1 | 0.336 | 0.047468621 |
| RNU6-1272P | LSM11 | 0.336 | 0.047468621 |
| RNU6-1272P | MRPL45 | 0.337 | 0.046820927 |
| RNU6-1272P | DLG1 | 0.337 | 0.046820927 |
| RNU6-1272P | SRSF12 | 0.337 | 0.046820927 |
| RNU6-1272P | PPP2R5E | 0.337 | 0.046820927 |
| RNU6-1272P | SEC63 | 0.337 | 0.046820927 |
| RNU6-1272P | ZNF93 | 0.337 | 0.046820927 |
| RNU6-1272P | MSH6 | 0.337 | 0.046820927 |
| RNU6-1272P | SCAMP1 | 0.338 | 0.046180686 |
| RNU6-1272P | TPP2 | 0.338 | 0.046180686 |
| RNU6-1272P | TBL1XR1 | 0.338 | 0.046180686 |
| RNU6-1272P | TAMM41 | 0.339 | 0.045547832 |
| RNU6-1272P | R3HDM1 | 0.339 | 0.045547832 |
| RNU6-1272P | CEP97 | 0.339 | 0.045547832 |
| RNU6-1272P | FAM210A | 0.339 | 0.045547832 |
| RNU6-1272P | NKIRAS1 | 0.34 | 0.044922302 |
| RNU6-1272P | CHRNA7 | 0.34 | 0.044922302 |
| RNU6-1272P | PAPOLB | 0.34 | 0.044922302 |
| RNU6-1272P | TSN | 0.34 | 0.044922302 |
| RNU6-1272P | ZNF140 | 0.34 | 0.044922302 |
| RNU6-1272P | ZNF624 | 0.341 | 0.044304033 |
| RNU6-1272P | ZCCHC4 | 0.341 | 0.044304033 |
| RNU6-1272P | CCDC125 | 0.341 | 0.044304033 |
| RNU6-1272P | RRP15 | 0.341 | 0.044304033 |
| RNU6-1272P | SECISBP2L | 0.341 | 0.044304033 |
| RNU6-1272P | ADSS | 0.341 | 0.044304033 |
| RNU6-1272P | PPIL4 | 0.342 | 0.043692961 |
| RNU6-1272P | PHAX | 0.342 | 0.043692961 |
| RNU6-1272P | TIA1 | 0.342 | 0.043692961 |
| RNU6-1272P | WIPF3 | 0.342 | 0.043692961 |
| RNU6-1272P | FAM149B1 | 0.342 | 0.043692961 |
| RNU6-1272P | EIF2A | 0.342 | 0.043692961 |
| RNU6-1272P | CSPP1 | 0.342 | 0.043692961 |
| RNU6-1272P | SCO1 | 0.342 | 0.043692961 |
| RNU6-1272P | IREB2 | 0.343 | 0.043089021 |
| RNU6-1272P | ORAI2 | 0.343 | 0.043089021 |
| RNU6-1272P | DNAAF4 | 0.343 | 0.043089021 |
| RNU6-1272P | UTP23 | 0.343 | 0.043089021 |
| RNU6-1272P | ZNF790 | 0.343 | 0.043089021 |
| RNU6-1272P | MIER3 | 0.343 | 0.043089021 |
| RNU6-1272P | ZNF549 | 0.343 | 0.043089021 |
| RNU6-1272P | TMEM200B | 0.343 | 0.043089021 |
| RNU6-1272P | AQP6 | 0.343 | 0.043089021 |
| RNU6-1272P | KLHL24 | 0.343 | 0.043089021 |
| RNU6-1272P | WAPL | 0.343 | 0.043089021 |
| RNU6-1272P | ANKRD46 | 0.343 | 0.043089021 |
| RNU6-1272P | UTP15 | 0.344 | 0.042492153 |
| RNU6-1272P | SYNCRIP | 0.344 | 0.042492153 |
| RNU6-1272P | ARFGEF1 | 0.344 | 0.042492153 |
| RNU6-1272P | SENP8 | 0.344 | 0.042492153 |
| RNU6-1272P | ZMYND11 | 0.344 | 0.042492153 |
| RNU6-1272P | STT3B | 0.344 | 0.042492153 |
| RNU6-1272P | ZNF726 | 0.344 | 0.042492153 |
| RNU6-1272P | PIK3CA | 0.345 | 0.041902291 |
| RNU6-1272P | TMEM260 | 0.345 | 0.041902291 |
| RNU6-1272P | ZG16B | 0.345 | 0.041902291 |
| RNU6-1272P | MRPL30 | 0.345 | 0.041902291 |
| RNU6-1272P | RSBN1L | 0.345 | 0.041902291 |
| RNU6-1272P | PRIMPOL | 0.345 | 0.041902291 |
| RNU6-1272P | ITGB4 | 0.346 | 0.041319375 |
| RNU6-1272P | ATAD5 | 0.346 | 0.041319375 |
| RNU6-1272P | GOLPH3L | 0.346 | 0.041319375 |
| RNU6-1272P | ZNF84 | 0.346 | 0.041319375 |
| RNU6-1272P | ATF2 | 0.346 | 0.041319375 |
| RNU6-1272P | CDH26 | 0.346 | 0.041319375 |
| RNU6-1272P | CRLF3 | 0.346 | 0.041319375 |
| RNU6-1272P | DCAF17 | 0.346 | 0.041319375 |
| RNU6-1272P | ZNF708 | 0.346 | 0.041319375 |
| RNU6-1272P | PCDH15 | 0.346 | 0.041319375 |
| RNU6-1272P | ZNF66 | 0.347 | 0.040743342 |
| RNU6-1272P | SLC44A5 | 0.347 | 0.040743342 |
| RNU6-1272P | ZNF682 | 0.347 | 0.040743342 |
| RNU6-1272P | PIKFYVE | 0.347 | 0.040743342 |
| RNU6-1272P | THOC1 | 0.348 | 0.04017413 |
| RNU6-1272P | ADNP | 0.348 | 0.04017413 |
| RNU6-1272P | TRPV3 | 0.348 | 0.04017413 |
| RNU6-1272P | DMC1 | 0.348 | 0.04017413 |
| RNU6-1272P | AC020915.2 | 0.348 | 0.04017413 |
| RNU6-1272P | YES1 | 0.348 | 0.04017413 |
| RNU6-1272P | TEFM | 0.348 | 0.04017413 |
| RNU6-1272P | AQP11 | 0.349 | 0.039611676 |
| RNU6-1272P | TRPM7 | 0.349 | 0.039611676 |
| RNU6-1272P | ZNF280B | 0.349 | 0.039611676 |
| RNU6-1272P | ZNF197 | 0.349 | 0.039611676 |
| RNU6-1272P | ZNF816 | 0.349 | 0.039611676 |
| RNU6-1272P | CENPK | 0.349 | 0.039611676 |
| RNU6-1272P | ETFRF1 | 0.349 | 0.039611676 |
| RNU6-1272P | ZFP14 | 0.35 | 0.039055921 |
| RNU6-1272P | EPCAM | 0.35 | 0.039055921 |
| RNU6-1272P | PHIP | 0.351 | 0.038506802 |
| RNU6-1272P | BRMS1L | 0.351 | 0.038506802 |
| RNU6-1272P | RBBP4 | 0.352 | 0.037964258 |
| RNU6-1272P | SOCS6 | 0.352 | 0.037964258 |
| RNU6-1272P | UBFD1 | 0.352 | 0.037964258 |
| RNU6-1272P | BPTF | 0.352 | 0.037964258 |
| RNU6-1272P | ZXDB | 0.352 | 0.037964258 |
| RNU6-1272P | MSH2 | 0.352 | 0.037964258 |
| RNU6-1272P | APPBP2 | 0.352 | 0.037964258 |
| RNU6-1272P | ZNF492 | 0.353 | 0.03742823 |
| RNU6-1272P | ZNF345 | 0.353 | 0.03742823 |
| RNU6-1272P | GPALPP1 | 0.353 | 0.03742823 |
| RNU6-1272P | IBTK | 0.353 | 0.03742823 |
| RNU6-1272P | FAM216A | 0.353 | 0.03742823 |
| RNU6-1272P | HELQ | 0.354 | 0.036898657 |
| RNU6-1272P | FAM60A | 0.354 | 0.036898657 |
| RNU6-1272P | LRRC37B | 0.354 | 0.036898657 |
| RNU6-1272P | SNX16 | 0.354 | 0.036898657 |
| RNU6-1272P | SLC25A36 | 0.354 | 0.036898657 |
| RNU6-1272P | ATP2C1 | 0.354 | 0.036898657 |
| RNU6-1272P | AC006030.1 | 0.354 | 0.036898657 |
| RNU6-1272P | MED28 | 0.354 | 0.036898657 |
| RNU6-1272P | SPIN1 | 0.355 | 0.036375478 |
| RNU6-1272P | FAM160B1 | 0.355 | 0.036375478 |
| RNU6-1272P | NFXL1 | 0.355 | 0.036375478 |
| RNU6-1272P | ZNF440 | 0.355 | 0.036375478 |
| RNU6-1272P | L2HGDH | 0.356 | 0.035858635 |
| RNU6-1272P | ZNF480 | 0.356 | 0.035858635 |
| RNU6-1272P | TLK1 | 0.356 | 0.035858635 |
| RNU6-1272P | KLRG2 | 0.356 | 0.035858635 |
| RNU6-1272P | UGDH | 0.356 | 0.035858635 |
| RNU6-1272P | NARS2 | 0.356 | 0.035858635 |
| RNU6-1272P | COIL | 0.356 | 0.035858635 |
| RNU6-1272P | SAP30L | 0.356 | 0.035858635 |
| RNU6-1272P | RNU6-1143P | 0.356 | 0.035858635 |
| RNU6-1272P | ZMYND8 | 0.357 | 0.035348067 |
| RNU6-1272P | DOCK7 | 0.357 | 0.035348067 |
| RNU6-1272P | CDK12 | 0.357 | 0.035348067 |
| RNU6-1272P | RIF1 | 0.357 | 0.035348067 |
| RNU6-1272P | C1orf112 | 0.358 | 0.034843716 |
| RNU6-1272P | ZZZ3 | 0.358 | 0.034843716 |
| RNU6-1272P | PUM2 | 0.358 | 0.034843716 |
| RNU6-1272P | AGO3 | 0.358 | 0.034843716 |
| RNU6-1272P | SMAD5 | 0.358 | 0.034843716 |
| RNU6-1272P | CCNJ | 0.359 | 0.034345523 |
| RNU6-1272P | TRMT10A | 0.359 | 0.034345523 |
| RNU6-1272P | BRAF | 0.359 | 0.034345523 |
| RNU6-1272P | NANOS1 | 0.359 | 0.034345523 |
| RNU6-1272P | REV3L | 0.359 | 0.034345523 |
| RNU6-1272P | CEP170 | 0.359 | 0.034345523 |
| RNU6-1272P | SCMH1 | 0.359 | 0.034345523 |
| RNU6-1272P | ZKSCAN7 | 0.36 | 0.033853429 |
| RNU6-1272P | CDK6 | 0.361 | 0.033367376 |
| RNU6-1272P | METTL2A | 0.361 | 0.033367376 |
| RNU6-1272P | ATAD1 | 0.361 | 0.033367376 |
| RNU6-1272P | ZNF439 | 0.361 | 0.033367376 |
| RNU6-1272P | ZNF813 | 0.361 | 0.033367376 |
| RNU6-1272P | OTUD6B | 0.361 | 0.033367376 |
| RNU6-1272P | ITGB3BP | 0.361 | 0.033367376 |
| RNU6-1272P | LYSMD3 | 0.361 | 0.033367376 |
| RNU6-1272P | RAD17 | 0.362 | 0.032887307 |
| RNU6-1272P | ZNF235 | 0.362 | 0.032887307 |
| RNU6-1272P | NT5C2 | 0.362 | 0.032887307 |
| RNU6-1272P | ZNF551 | 0.362 | 0.032887307 |
| RNU6-1272P | LRIG2 | 0.363 | 0.032413163 |
| RNU6-1272P | ZMAT3 | 0.363 | 0.032413163 |
| RNU6-1272P | ZNF430 | 0.363 | 0.032413163 |
| RNU6-1272P | SAMD13 | 0.363 | 0.032413163 |
| RNU6-1272P | ULK4 | 0.364 | 0.031944887 |
| RNU6-1272P | PUS7 | 0.364 | 0.031944887 |
| RNU6-1272P | ZNF280D | 0.364 | 0.031944887 |
| RNU6-1272P | SDCCAG8 | 0.364 | 0.031944887 |
| RNU6-1272P | VMA21 | 0.364 | 0.031944887 |
| RNU6-1272P | SEPSECS | 0.364 | 0.031944887 |
| RNU6-1272P | ZNF431 | 0.364 | 0.031944887 |
| RNU6-1272P | CEP41 | 0.364 | 0.031944887 |
| RNU6-1272P | POC1B | 0.365 | 0.031482423 |
| RNU6-1272P | LARP1B | 0.365 | 0.031482423 |
| RNU6-1272P | MFSD8 | 0.365 | 0.031482423 |
| RNU6-1272P | ORC2 | 0.365 | 0.031482423 |
| RNU6-1272P | RUNX1T1 | 0.365 | 0.031482423 |
| RNU6-1272P | ZNF608 | 0.365 | 0.031482423 |
| RNU6-1272P | TOMM70 | 0.365 | 0.031482423 |
| RNU6-1272P | ATE1 | 0.365 | 0.031482423 |
| RNU6-1272P | STXBP5 | 0.365 | 0.031482423 |
| RNU6-1272P | NCAN | 0.366 | 0.031025713 |
| RNU6-1272P | PTGDR2 | 0.366 | 0.031025713 |
| RNU6-1272P | ZNF382 | 0.366 | 0.031025713 |
| RNU6-1272P | INTS13 | 0.366 | 0.031025713 |
| RNU6-1272P | C2CD4C | 0.366 | 0.031025713 |
| RNU6-1272P | ARL1 | 0.366 | 0.031025713 |
| RNU6-1272P | NUP54 | 0.366 | 0.031025713 |
| RNU6-1272P | GPBP1 | 0.367 | 0.030574702 |
| RNU6-1272P | BEND3 | 0.367 | 0.030574702 |
| RNU6-1272P | MYNN | 0.367 | 0.030574702 |
| RNU6-1272P | TERB2 | 0.368 | 0.030129334 |
| RNU6-1272P | TRUB1 | 0.368 | 0.030129334 |
| RNU6-1272P | CENPC | 0.368 | 0.030129334 |
| RNU6-1272P | CLINT1 | 0.368 | 0.030129334 |
| RNU6-1272P | AIRE | 0.368 | 0.030129334 |
| RNU6-1272P | ZNF766 | 0.37 | 0.029255302 |
| RNU6-1272P | TMEFF1 | 0.37 | 0.029255302 |
| RNU6-1272P | HAPLN4 | 0.37 | 0.029255302 |
| RNU6-1272P | UBXN2A | 0.37 | 0.029255302 |
| RNU6-1272P | APOOL | 0.371 | 0.028826528 |
| RNU6-1272P | TRMT13 | 0.371 | 0.028826528 |
| RNU6-1272P | LRIF1 | 0.371 | 0.028826528 |
| RNU6-1272P | ZNF569 | 0.371 | 0.028826528 |
| RNU6-1272P | POU4F1 | 0.371 | 0.028826528 |
| RNU6-1272P | PIRT | 0.372 | 0.028403175 |
| RNU6-1272P | TMEM44 | 0.372 | 0.028403175 |
| RNU6-1272P | C17orf78 | 0.373 | 0.02798519 |
| RNU6-1272P | FBXL13 | 0.373 | 0.02798519 |
| RNU6-1272P | ZNF92 | 0.373 | 0.02798519 |
| RNU6-1272P | OR6J1 | 0.373 | 0.02798519 |
| RNU6-1272P | ZRANB3 | 0.373 | 0.02798519 |
| RNU6-1272P | DIMT1 | 0.373 | 0.02798519 |
| RNU6-1272P | IPO11 | 0.374 | 0.027572518 |
| RNU6-1272P | CARNMT1 | 0.374 | 0.027572518 |
| RNU6-1272P | PPP4R2 | 0.374 | 0.027572518 |
| RNU6-1272P | RRAGD | 0.374 | 0.027572518 |
| RNU6-1272P | WFDC1 | 0.374 | 0.027572518 |
| RNU6-1272P | ZNF506 | 0.375 | 0.027165105 |
| RNU6-1272P | POLR3F | 0.375 | 0.027165105 |
| RNU6-1272P | HS2ST1 | 0.375 | 0.027165105 |
| RNU6-1272P | ZNF518A | 0.375 | 0.027165105 |
| RNU6-1272P | ZKSCAN3 | 0.376 | 0.026762899 |
| RNU6-1272P | GBE1 | 0.376 | 0.026762899 |
| RNU6-1272P | ZNF146 | 0.377 | 0.026365845 |
| RNU6-1272P | XPO4 | 0.377 | 0.026365845 |
| RNU6-1272P | ZSCAN31 | 0.378 | 0.025973892 |
| RNU6-1272P | SMARCC1 | 0.378 | 0.025973892 |
| RNU6-1272P | DBT | 0.379 | 0.025586986 |
| RNU6-1272P | MIGA1 | 0.379 | 0.025586986 |
| RNU6-1272P | TRMT61B | 0.38 | 0.025205076 |
| RNU6-1272P | CDCA7 | 0.38 | 0.025205076 |
| RNU6-1272P | G3BP1 | 0.38 | 0.025205076 |
| RNU6-1272P | ZNF529 | 0.38 | 0.025205076 |
| RNU6-1272P | MED17 | 0.381 | 0.024828111 |
| RNU6-1272P | EEF2K | 0.381 | 0.024828111 |
| RNU6-1272P | ALS2 | 0.381 | 0.024828111 |
| RNU6-1272P | TEX10 | 0.381 | 0.024828111 |
| RNU6-1272P | GNPNAT1 | 0.381 | 0.024828111 |
| RNU6-1272P | RUFY2 | 0.381 | 0.024828111 |
| RNU6-1272P | SLC35F5 | 0.382 | 0.024456038 |
| RNU6-1272P | C1orf186 | 0.382 | 0.024456038 |
| RNU6-1272P | STK26 | 0.383 | 0.024088806 |
| RNU6-1272P | RCN2 | 0.383 | 0.024088806 |
| RNU6-1272P | ZCCHC11 | 0.383 | 0.024088806 |
| RNU6-1272P | KAT6B | 0.384 | 0.023726366 |
| RNU6-1272P | GPR12 | 0.384 | 0.023726366 |
| RNU6-1272P | GLS | 0.385 | 0.023368666 |
| RNU6-1272P | TMEM181 | 0.385 | 0.023368666 |
| RNU6-1272P | ZNF69 | 0.386 | 0.023015656 |
| RNU6-1272P | LRRC34 | 0.386 | 0.023015656 |
| RNU6-1272P | ETV6 | 0.386 | 0.023015656 |
| RNU6-1272P | ZNF681 | 0.386 | 0.023015656 |
| RNU6-1272P | ZNF85 | 0.387 | 0.022667287 |
| RNU6-1272P | HPGDS | 0.387 | 0.022667287 |
| RNU6-1272P | FAM227B | 0.387 | 0.022667287 |
| RNU6-1272P | SPDYE3 | 0.388 | 0.02232351 |
| RNU6-1272P | ZNF610 | 0.388 | 0.02232351 |
| RNU6-1272P | BCKDHB | 0.39 | 0.021649534 |
| RNU6-1272P | CAAP1 | 0.391 | 0.021319238 |
| RNU6-1272P | GPSM2 | 0.391 | 0.021319238 |
| RNU6-1272P | EFCAB13 | 0.391 | 0.021319238 |
| RNU6-1272P | ZNF320 | 0.391 | 0.021319238 |
| RNU6-1272P | ZFP1 | 0.391 | 0.021319238 |
| RNU6-1272P | NFAT5 | 0.392 | 0.020993339 |
| RNU6-1272P | RNF125 | 0.392 | 0.020993339 |
| RNU6-1272P | ZNF566 | 0.392 | 0.020993339 |
| RNU6-1272P | ZNF260 | 0.392 | 0.020993339 |
| RNU6-1272P | HSD17B11 | 0.392 | 0.020993339 |
| RNU6-1272P | TOGARAM1 | 0.393 | 0.020671791 |
| RNU6-1272P | CCDC138 | 0.393 | 0.020671791 |
| RNU6-1272P | DYDC2 | 0.394 | 0.020354544 |
| RNU6-1272P | KLHL23 | 0.394 | 0.020354544 |
| RNU6-1272P | GFPT1 | 0.394 | 0.020354544 |
| RNU6-1272P | CEP290 | 0.394 | 0.020354544 |
| RNU6-1272P | AKAP9 | 0.395 | 0.020041553 |
| RNU6-1272P | RAB7B | 0.395 | 0.020041553 |
| RNU6-1272P | ZNF445 | 0.396 | 0.019732771 |
| RNU6-1272P | ALDH6A1 | 0.396 | 0.019732771 |
| RNU6-1272P | METTL14 | 0.396 | 0.019732771 |
| RNU6-1272P | PGPEP1L | 0.397 | 0.019428152 |
| RNU6-1272P | SERINC5 | 0.397 | 0.019428152 |
| RNU6-1272P | MYOZ3 | 0.397 | 0.019428152 |
| RNU6-1272P | SUZ12 | 0.398 | 0.019127649 |
| RNU6-1272P | LMAN1 | 0.398 | 0.019127649 |
| RNU6-1272P | PDS5A | 0.398 | 0.019127649 |
| RNU6-1272P | MTMR2 | 0.398 | 0.019127649 |
| RNU6-1272P | BDP1 | 0.399 | 0.018831218 |
| RNU6-1272P | ZSCAN23 | 0.4 | 0.018538812 |
| RNU6-1272P | ARC | 0.401 | 0.018250387 |
| RNU6-1272P | NUDT13 | 0.401 | 0.018250387 |
| RNU6-1272P | KIAA1958 | 0.401 | 0.018250387 |
| RNU6-1272P | MAPKAPK5 | 0.402 | 0.017965899 |
| RNU6-1272P | KLHL28 | 0.402 | 0.017965899 |
| RNU6-1272P | ZMYM4 | 0.402 | 0.017965899 |
| RNU6-1272P | CTDSPL2 | 0.403 | 0.017685302 |
| RNU6-1272P | ZKSCAN2 | 0.403 | 0.017685302 |
| RNU6-1272P | ZNF660 | 0.403 | 0.017685302 |
| RNU6-1272P | ZNF720 | 0.403 | 0.017685302 |
| RNU6-1272P | ZNF26 | 0.404 | 0.017408554 |
| RNU6-1272P | DENR | 0.404 | 0.017408554 |
| RNU6-1272P | ANKRD26 | 0.405 | 0.017135611 |
| RNU6-1272P | KCNQ5 | 0.405 | 0.017135611 |
| RNU6-1272P | ZNF107 | 0.405 | 0.017135611 |
| RNU6-1272P | LPCAT2 | 0.406 | 0.016866429 |
| RNU6-1272P | ZNF280C | 0.406 | 0.016866429 |
| RNU6-1272P | ZNF233 | 0.406 | 0.016866429 |
| RNU6-1272P | RPAP2 | 0.406 | 0.016866429 |
| RNU6-1272P | MTF2 | 0.406 | 0.016866429 |
| RNU6-1272P | CEP57 | 0.407 | 0.016600966 |
| RNU6-1272P | ZNF124 | 0.407 | 0.016600966 |
| RNU6-1272P | ZNF138 | 0.408 | 0.01633918 |
| RNU6-1272P | ZNF227 | 0.408 | 0.01633918 |
| RNU6-1272P | GTPBP10 | 0.41 | 0.015826467 |
| RNU6-1272P | MGA | 0.411 | 0.015575457 |
| RNU6-1272P | ZSCAN12 | 0.411 | 0.015575457 |
| RNU6-1272P | ZNF253 | 0.411 | 0.015575457 |
| RNU6-1272P | SBK2 | 0.416 | 0.014372242 |
| RNU6-1272P | INA | 0.417 | 0.014141685 |
| RNU6-1272P | KIAA1841 | 0.417 | 0.014141685 |
| RNU6-1272P | ZNF680 | 0.418 | 0.013914398 |
| RNU6-1272P | PTGR2 | 0.418 | 0.013914398 |
| RNU6-1272P | KDM5B | 0.418 | 0.013914398 |
| RNU6-1272P | SBK3 | 0.419 | 0.013690342 |
| RNU6-1272P | ANKRD33B | 0.42 | 0.013469479 |
| RNU6-1272P | CNOT6 | 0.425 | 0.012411729 |
| RNU6-1272P | ZNF184 | 0.426 | 0.012209233 |
| RNU6-1272P | ZNF443 | 0.426 | 0.012209233 |
| RNU6-1272P | NEDD4 | 0.426 | 0.012209233 |
| RNU6-1272P | GALNT7 | 0.427 | 0.012009669 |
| RNU6-1272P | FAM175A | 0.427 | 0.012009669 |
| RNU6-1272P | PRELID2 | 0.428 | 0.011813003 |
| RNU6-1272P | ZNF730 | 0.428 | 0.011813003 |
| RNU6-1272P | ZNF670 | 0.429 | 0.011619198 |
| RNU6-1272P | ZNF100 | 0.43 | 0.011428219 |
| RNU6-1272P | MTPAP | 0.432 | 0.011054602 |
| RNU6-1272P | ZC3HAV1L | 0.433 | 0.010871894 |
| RNU6-1272P | ATXN2 | 0.434 | 0.010691876 |
| RNU6-1272P | ZNF675 | 0.434 | 0.010691876 |
| RNU6-1272P | SSC5D | 0.435 | 0.010514512 |
| RNU6-1272P | TMIE | 0.436 | 0.010339771 |
| RNU6-1272P | ZFP30 | 0.438 | 0.009998024 |
| RNU6-1272P | ZBTB8A | 0.438 | 0.009998024 |
| RNU6-1272P | TCAIM | 0.439 | 0.009830953 |
| RNU6-1272P | FNDC3A | 0.439 | 0.009830953 |
| RNU6-1272P | GALNT1 | 0.441 | 0.009504255 |
| RNU6-1272P | ZNF43 | 0.441 | 0.009504255 |
| RNU6-1272P | GMCL1 | 0.441 | 0.009504255 |
| RNU6-1272P | TM7SF3 | 0.442 | 0.009344566 |
| RNU6-1272P | C11orf54 | 0.443 | 0.009187275 |
| RNU6-1272P | MYO9A | 0.444 | 0.009032352 |
| RNU6-1272P | MYB | 0.444 | 0.009032352 |
| RNU6-1272P | MBTD1 | 0.445 | 0.008879765 |
| RNU6-1272P | USO1 | 0.446 | 0.008729485 |
| RNU6-1272P | MBLAC2 | 0.447 | 0.008581482 |
| RNU6-1272P | LYRM7 | 0.45 | 0.00815084 |
| RNU6-1272P | PIBF1 | 0.452 | 0.007874595 |
| RNU6-1272P | SLC35A3 | 0.453 | 0.007739643 |
| RNU6-1272P | GLMN | 0.453 | 0.007739643 |
| RNU6-1272P | DENND1B | 0.455 | 0.00747594 |
| RNU6-1272P | AGBL3 | 0.456 | 0.007347134 |
| RNU6-1272P | TRIM24 | 0.458 | 0.007095479 |
| RNU6-1272P | SMARCAD1 | 0.458 | 0.007095479 |
| RNU6-1272P | THAP9 | 0.459 | 0.006972577 |
| RNU6-1272P | UBA6 | 0.46 | 0.006851591 |
| RNU6-1272P | TRNT1 | 0.462 | 0.006615261 |
| RNU6-1272P | SERF1B | 0.463 | 0.006499867 |
| RNU6-1272P | C5orf24 | 0.466 | 0.006164469 |
| RNU6-1272P | DEPTOR | 0.47 | 0.005741528 |
| RNU6-1272P | REST | 0.47 | 0.005741528 |
| RNU6-1272P | MEST | 0.471 | 0.005639967 |
| RNU6-1272P | ZNF254 | 0.472 | 0.00554003 |
| RNU6-1272P | GOSR1 | 0.474 | 0.005344935 |
| RNU6-1272P | ZNF195 | 0.474 | 0.005344935 |
| RNU6-1272P | DEPDC7 | 0.478 | 0.004973248 |
| RNU6-1272P | MED21 | 0.478 | 0.004973248 |
| RNU6-1272P | B3GLCT | 0.48 | 0.004796312 |
| RNU6-1272P | MIB1 | 0.504 | 0.003075163 |
| RNU6-1272P | PRRC1 | 0.51 | 0.002743976 |
| RNU6-1272P | DPY19L3 | 0.515 | 0.002493241 |
| RNU6-1272P | ZMYM1 | 0.526 | 0.002013742 |
| RNU6-1272P | SVOPL | 0.53 | 0.001861493 |
| RNU6-1272P | KIAA1549 | 0.6 | 0.000433183 |
| RNU6-1272P | RNU6-1272P | 1 | 4.99E-09 |
| RNU6-1143P | UNC45A | -0.468 | 0.007475802 |
| RNU6-1143P | AAMP | -0.463 | 0.008137204 |
| RNU6-1143P | AP2M1 | -0.455 | 0.009304825 |
| RNU6-1143P | TRADD | -0.442 | 0.011523089 |
| RNU6-1143P | TRAPPC4 | -0.442 | 0.011523089 |
| RNU6-1143P | STUB1 | -0.442 | 0.011523089 |
| RNU6-1143P | MCRS1 | -0.439 | 0.01209729 |
| RNU6-1143P | OXLD1 | -0.434 | 0.013110786 |
| RNU6-1143P | FBXW5 | -0.433 | 0.013322268 |
| RNU6-1143P | HPS1 | -0.432 | 0.01353676 |
| RNU6-1143P | TMEM187 | -0.43 | 0.013974917 |
| RNU6-1143P | E2F4 | -0.427 | 0.014655629 |
| RNU6-1143P | PYCR2 | -0.425 | 0.01512552 |
| RNU6-1143P | STK25 | -0.425 | 0.01512552 |
| RNU6-1143P | NUDT16L1 | -0.423 | 0.015608628 |
| RNU6-1143P | KXD1 | -0.422 | 0.015855235 |
| RNU6-1143P | SSNA1 | -0.419 | 0.016615731 |
| RNU6-1143P | RPP25L | -0.419 | 0.016615731 |
| RNU6-1143P | CYB561D2 | -0.418 | 0.016876253 |
| RNU6-1143P | APEX2 | -0.417 | 0.017140354 |
| RNU6-1143P | TMEM141 | -0.417 | 0.017140354 |
| RNU6-1143P | PORCN | -0.415 | 0.017679454 |
| RNU6-1143P | PLEKHJ1 | -0.415 | 0.017679454 |
| RNU6-1143P | NUDT22 | -0.414 | 0.017954534 |
| RNU6-1143P | NELFB | -0.412 | 0.018515963 |
| RNU6-1143P | FAM96B | -0.411 | 0.018802395 |
| RNU6-1143P | ATG101 | -0.41 | 0.019092695 |
| RNU6-1143P | TMEM222 | -0.407 | 0.019987232 |
| RNU6-1143P | TSR3 | -0.406 | 0.020293434 |
| RNU6-1143P | RBCK1 | -0.406 | 0.020293434 |
| RNU6-1143P | DHRS4 | -0.402 | 0.021559533 |
| RNU6-1143P | BET1L | -0.402 | 0.021559533 |
| RNU6-1143P | PUSL1 | -0.401 | 0.021886603 |
| RNU6-1143P | USP5 | -0.4 | 0.022217983 |
| RNU6-1143P | FAAP100 | -0.4 | 0.022217983 |
| RNU6-1143P | MON1A | -0.4 | 0.022217983 |
| RNU6-1143P | MRPS34 | -0.399 | 0.022553717 |
| RNU6-1143P | TSTA3 | -0.398 | 0.022893852 |
| RNU6-1143P | RNF25 | -0.398 | 0.022893852 |
| RNU6-1143P | TMEM129 | -0.398 | 0.022893852 |
| RNU6-1143P | ZNF213 | -0.398 | 0.022893852 |
| RNU6-1143P | LLGL1 | -0.397 | 0.023238434 |
| RNU6-1143P | SCAMP3 | -0.396 | 0.023587511 |
| RNU6-1143P | NDUFB10 | -0.396 | 0.023587511 |
| RNU6-1143P | PSMD2 | -0.395 | 0.023941128 |
| RNU6-1143P | TAB1 | -0.395 | 0.023941128 |
| RNU6-1143P | TJAP1 | -0.395 | 0.023941128 |
| RNU6-1143P | CNOT3 | -0.394 | 0.024299334 |
| RNU6-1143P | C19orf60 | -0.394 | 0.024299334 |
| RNU6-1143P | COQ9 | -0.394 | 0.024299334 |
| RNU6-1143P | PGAP3 | -0.394 | 0.024299334 |
| RNU6-1143P | TEX261 | -0.392 | 0.025029703 |
| RNU6-1143P | URM1 | -0.392 | 0.025029703 |
| RNU6-1143P | VPS16 | -0.391 | 0.025401963 |
| RNU6-1143P | RNF187 | -0.391 | 0.025401963 |
| RNU6-1143P | FKBP8 | -0.39 | 0.025779003 |
| RNU6-1143P | TRMT2A | -0.39 | 0.025779003 |
| RNU6-1143P | USP19 | -0.39 | 0.025779003 |
| RNU6-1143P | STK11 | -0.39 | 0.025779003 |
| RNU6-1143P | RABGGTA | -0.389 | 0.026160874 |
| RNU6-1143P | ANAPC11 | -0.387 | 0.026939304 |
| RNU6-1143P | FIBP | -0.387 | 0.026939304 |
| RNU6-1143P | ZMAT5 | -0.387 | 0.026939304 |
| RNU6-1143P | BTBD2 | -0.387 | 0.026939304 |
| RNU6-1143P | APEH | -0.386 | 0.027335962 |
| RNU6-1143P | WDR46 | -0.385 | 0.02773765 |
| RNU6-1143P | PTGES2 | -0.385 | 0.02773765 |
| RNU6-1143P | FAM234A | -0.385 | 0.02773765 |
| RNU6-1143P | DUSP23 | -0.385 | 0.02773765 |
| RNU6-1143P | MUS81 | -0.385 | 0.02773765 |
| RNU6-1143P | NUDT18 | -0.384 | 0.028144417 |
| RNU6-1143P | ATP13A1 | -0.384 | 0.028144417 |
| RNU6-1143P | RILP | -0.384 | 0.028144417 |
| RNU6-1143P | UCK1 | -0.384 | 0.028144417 |
| RNU6-1143P | CYB5R3 | -0.383 | 0.028556315 |
| RNU6-1143P | CNPPD1 | -0.382 | 0.028973395 |
| RNU6-1143P | DHX16 | -0.381 | 0.029395707 |
| RNU6-1143P | JMJD8 | -0.381 | 0.029395707 |
| RNU6-1143P | TAF6 | -0.381 | 0.029395707 |
| RNU6-1143P | TCTA | -0.381 | 0.029395707 |
| RNU6-1143P | MRPL43 | -0.38 | 0.029823304 |
| RNU6-1143P | CXXC1 | -0.38 | 0.029823304 |
| RNU6-1143P | IRAK1 | -0.38 | 0.029823304 |
| RNU6-1143P | NPRL2 | -0.38 | 0.029823304 |
| RNU6-1143P | PSMB6 | -0.38 | 0.029823304 |
| RNU6-1143P | CAPN10 | -0.379 | 0.030256238 |
| RNU6-1143P | C2CD2L | -0.379 | 0.030256238 |
| RNU6-1143P | NME3 | -0.378 | 0.030694562 |
| RNU6-1143P | SYNGR2 | -0.378 | 0.030694562 |
| RNU6-1143P | SLC52A2 | -0.377 | 0.031138326 |
| RNU6-1143P | IDH3B | -0.377 | 0.031138326 |
| RNU6-1143P | SMPD1 | -0.377 | 0.031138326 |
| RNU6-1143P | FTSJ1 | -0.377 | 0.031138326 |
| RNU6-1143P | ZFYVE19 | -0.376 | 0.031587586 |
| RNU6-1143P | HECTD3 | -0.376 | 0.031587586 |
| RNU6-1143P | MLST8 | -0.375 | 0.032042393 |
| RNU6-1143P | MIGA2 | -0.375 | 0.032042393 |
| RNU6-1143P | HSD17B10 | -0.375 | 0.032042393 |
| RNU6-1143P | NSMCE1 | -0.374 | 0.032502802 |
| RNU6-1143P | LAGE3 | -0.374 | 0.032502802 |
| RNU6-1143P | C19orf24 | -0.374 | 0.032502802 |
| RNU6-1143P | TKFC | -0.374 | 0.032502802 |
| RNU6-1143P | LMF2 | -0.373 | 0.032968865 |
| RNU6-1143P | TXNRD2 | -0.373 | 0.032968865 |
| RNU6-1143P | ENTPD6 | -0.373 | 0.032968865 |
| RNU6-1143P | CDPF1 | -0.373 | 0.032968865 |
| RNU6-1143P | HDDC3 | -0.372 | 0.033440638 |
| RNU6-1143P | CENPT | -0.372 | 0.033440638 |
| RNU6-1143P | STX4 | -0.372 | 0.033440638 |
| RNU6-1143P | NDUFS2 | -0.372 | 0.033440638 |
| RNU6-1143P | CUEDC2 | -0.371 | 0.033918175 |
| RNU6-1143P | PUF60 | -0.371 | 0.033918175 |
| RNU6-1143P | INTS5 | -0.371 | 0.033918175 |
| RNU6-1143P | YIF1A | -0.371 | 0.033918175 |
| RNU6-1143P | TBCB | -0.371 | 0.033918175 |
| RNU6-1143P | TRAPPC1 | -0.371 | 0.033918175 |
| RNU6-1143P | RASSF7 | -0.37 | 0.034401529 |
| RNU6-1143P | SCYL1 | -0.37 | 0.034401529 |
| RNU6-1143P | PSMG3 | -0.37 | 0.034401529 |
| RNU6-1143P | MEN1 | -0.37 | 0.034401529 |
| RNU6-1143P | LSM10 | -0.37 | 0.034401529 |
| RNU6-1143P | CLPTM1 | -0.37 | 0.034401529 |
| RNU6-1143P | HSD17B8 | -0.37 | 0.034401529 |
| RNU6-1143P | SLC38A10 | -0.369 | 0.034890757 |
| RNU6-1143P | POMGNT1 | -0.369 | 0.034890757 |
| RNU6-1143P | SLC25A28 | -0.369 | 0.034890757 |
| RNU6-1143P | ZFPL1 | -0.369 | 0.034890757 |
| RNU6-1143P | TOR2A | -0.369 | 0.034890757 |
| RNU6-1143P | PDCD6 | -0.368 | 0.035385913 |
| RNU6-1143P | POLR2E | -0.368 | 0.035385913 |
| RNU6-1143P | ZNF319 | -0.368 | 0.035385913 |
| RNU6-1143P | CALCOCO1 | -0.368 | 0.035385913 |
| RNU6-1143P | PCBP1 | -0.368 | 0.035385913 |
| RNU6-1143P | EIF3I | -0.367 | 0.035887053 |
| RNU6-1143P | GPS1 | -0.367 | 0.035887053 |
| RNU6-1143P | FBXL15 | -0.367 | 0.035887053 |
| RNU6-1143P | GUK1 | -0.367 | 0.035887053 |
| RNU6-1143P | MBD1 | -0.366 | 0.036394233 |
| RNU6-1143P | C1orf123 | -0.366 | 0.036394233 |
| RNU6-1143P | C11orf68 | -0.365 | 0.036907509 |
| RNU6-1143P | DVL1 | -0.365 | 0.036907509 |
| RNU6-1143P | PQBP1 | -0.365 | 0.036907509 |
| RNU6-1143P | DNAJB2 | -0.365 | 0.036907509 |
| RNU6-1143P | NDUFAF8 | -0.365 | 0.036907509 |
| RNU6-1143P | SLC29A3 | -0.365 | 0.036907509 |
| RNU6-1143P | ACBD4 | -0.365 | 0.036907509 |
| RNU6-1143P | SLC10A3 | -0.364 | 0.037426936 |
| RNU6-1143P | TUFM | -0.364 | 0.037426936 |
| RNU6-1143P | MRPL23 | -0.364 | 0.037426936 |
| RNU6-1143P | POLR2G | -0.364 | 0.037426936 |
| RNU6-1143P | MIB2 | -0.363 | 0.037952572 |
| RNU6-1143P | RPS19BP1 | -0.363 | 0.037952572 |
| RNU6-1143P | ACADS | -0.363 | 0.037952572 |
| RNU6-1143P | BAG6 | -0.363 | 0.037952572 |
| RNU6-1143P | PPOX | -0.362 | 0.038484474 |
| RNU6-1143P | ATG2A | -0.362 | 0.038484474 |
| RNU6-1143P | FAM50A | -0.362 | 0.038484474 |
| RNU6-1143P | CRTC1 | -0.362 | 0.038484474 |
| RNU6-1143P | PSMC5 | -0.361 | 0.039022699 |
| RNU6-1143P | RFNG | -0.361 | 0.039022699 |
| RNU6-1143P | INTS11 | -0.361 | 0.039022699 |
| RNU6-1143P | CCS | -0.361 | 0.039022699 |
| RNU6-1143P | SDHA | -0.361 | 0.039022699 |
| RNU6-1143P | VPS28 | -0.361 | 0.039022699 |
| RNU6-1143P | C16orf86 | -0.361 | 0.039022699 |
| RNU6-1143P | DCAF11 | -0.361 | 0.039022699 |
| RNU6-1143P | POLRMT | -0.361 | 0.039022699 |
| RNU6-1143P | DUS1L | -0.361 | 0.039022699 |
| RNU6-1143P | L3MBTL2 | -0.361 | 0.039022699 |
| RNU6-1143P | COASY | -0.36 | 0.039567303 |
| RNU6-1143P | VPS25 | -0.36 | 0.039567303 |
| RNU6-1143P | NUBP2 | -0.36 | 0.039567303 |
| RNU6-1143P | GTPBP2 | -0.36 | 0.039567303 |
| RNU6-1143P | TSPAN17 | -0.36 | 0.039567303 |
| RNU6-1143P | CCDC22 | -0.36 | 0.039567303 |
| RNU6-1143P | CCDC130 | -0.36 | 0.039567303 |
| RNU6-1143P | THAP7 | -0.36 | 0.039567303 |
| RNU6-1143P | CREB3 | -0.36 | 0.039567303 |
| RNU6-1143P | CCDC137 | -0.36 | 0.039567303 |
| RNU6-1143P | SDF4 | -0.36 | 0.039567303 |
| RNU6-1143P | RPUSD1 | -0.359 | 0.040118345 |
| RNU6-1143P | RHOT2 | -0.359 | 0.040118345 |
| RNU6-1143P | GBA2 | -0.359 | 0.040118345 |
| RNU6-1143P | STK11IP | -0.359 | 0.040118345 |
| RNU6-1143P | SLC25A22 | -0.359 | 0.040118345 |
| RNU6-1143P | DDX41 | -0.358 | 0.040675883 |
| RNU6-1143P | C1orf122 | -0.358 | 0.040675883 |
| RNU6-1143P | AKR1A1 | -0.357 | 0.041239975 |
| RNU6-1143P | CDC37 | -0.357 | 0.041239975 |
| RNU6-1143P | ANAPC2 | -0.357 | 0.041239975 |
| RNU6-1143P | WDR13 | -0.357 | 0.041239975 |
| RNU6-1143P | APBA3 | -0.356 | 0.041810679 |
| RNU6-1143P | GPAA1 | -0.356 | 0.041810679 |
| RNU6-1143P | IRF3 | -0.356 | 0.041810679 |
| RNU6-1143P | PCYT2 | -0.356 | 0.041810679 |
| RNU6-1143P | MRPS12 | -0.356 | 0.041810679 |
| RNU6-1143P | PHB | -0.356 | 0.041810679 |
| RNU6-1143P | CDK10 | -0.356 | 0.041810679 |
| RNU6-1143P | APRT | -0.355 | 0.042388054 |
| RNU6-1143P | YIPF3 | -0.355 | 0.042388054 |
| RNU6-1143P | PSMF1 | -0.355 | 0.042388054 |
| RNU6-1143P | LZTR1 | -0.354 | 0.042972158 |
| RNU6-1143P | TRMT61A | -0.354 | 0.042972158 |
| RNU6-1143P | LIMK1 | -0.354 | 0.042972158 |
| RNU6-1143P | EIF2B1 | -0.354 | 0.042972158 |
| RNU6-1143P | WDR83OS | -0.354 | 0.042972158 |
| RNU6-1143P | UQCRC1 | -0.354 | 0.042972158 |
| RNU6-1143P | TMEM42 | -0.354 | 0.042972158 |
| RNU6-1143P | PI4KB | -0.354 | 0.042972158 |
| RNU6-1143P | ZNF524 | -0.354 | 0.042972158 |
| RNU6-1143P | CPTP | -0.353 | 0.043563052 |
| RNU6-1143P | TBC1D17 | -0.353 | 0.043563052 |
| RNU6-1143P | MRPS2 | -0.353 | 0.043563052 |
| RNU6-1143P | ARHGEF19 | -0.353 | 0.043563052 |
| RNU6-1143P | NOSIP | -0.353 | 0.043563052 |
| RNU6-1143P | ZFAND2B | -0.353 | 0.043563052 |
| RNU6-1143P | OSBPL7 | -0.353 | 0.043563052 |
| RNU6-1143P | ASB6 | -0.353 | 0.043563052 |
| RNU6-1143P | TBL3 | -0.353 | 0.043563052 |
| RNU6-1143P | GPKOW | -0.353 | 0.043563052 |
| RNU6-1143P | EML3 | -0.352 | 0.044160795 |
| RNU6-1143P | SCRN2 | -0.352 | 0.044160795 |
| RNU6-1143P | FASTK | -0.352 | 0.044160795 |
| RNU6-1143P | ULK3 | -0.352 | 0.044160795 |
| RNU6-1143P | TMEM250 | -0.352 | 0.044160795 |
| RNU6-1143P | AP2A1 | -0.352 | 0.044160795 |
| RNU6-1143P | CXorf40A | -0.352 | 0.044160795 |
| RNU6-1143P | TMEM8A | -0.352 | 0.044160795 |
| RNU6-1143P | AURKAIP1 | -0.351 | 0.044765445 |
| RNU6-1143P | SIGIRR | -0.351 | 0.044765445 |
| RNU6-1143P | RAB11B | -0.351 | 0.044765445 |
| RNU6-1143P | COA3 | -0.351 | 0.044765445 |
| RNU6-1143P | COMMD4 | -0.351 | 0.044765445 |
| RNU6-1143P | ACTR1B | -0.35 | 0.045377064 |
| RNU6-1143P | G6PC3 | -0.35 | 0.045377064 |
| RNU6-1143P | TEX264 | -0.35 | 0.045377064 |
| RNU6-1143P | ALDOA | -0.35 | 0.045377064 |
| RNU6-1143P | SPOUT1 | -0.35 | 0.045377064 |
| RNU6-1143P | RNF40 | -0.35 | 0.045377064 |
| RNU6-1143P | THAP11 | -0.349 | 0.045995711 |
| RNU6-1143P | CCDC51 | -0.349 | 0.045995711 |
| RNU6-1143P | YIF1B | -0.349 | 0.045995711 |
| RNU6-1143P | KCTD2 | -0.349 | 0.045995711 |
| RNU6-1143P | SURF1 | -0.349 | 0.045995711 |
| RNU6-1143P | GPX1 | -0.349 | 0.045995711 |
| RNU6-1143P | MVK | -0.349 | 0.045995711 |
| RNU6-1143P | DCXR | -0.349 | 0.045995711 |
| RNU6-1143P | ISOC2 | -0.349 | 0.045995711 |
| RNU6-1143P | ZNHIT2 | -0.348 | 0.046621446 |
| RNU6-1143P | ZNF513 | -0.347 | 0.047254331 |
| RNU6-1143P | FRS3 | -0.347 | 0.047254331 |
| RNU6-1143P | CD2BP2 | -0.347 | 0.047254331 |
| RNU6-1143P | TMUB2 | -0.347 | 0.047254331 |
| RNU6-1143P | GDI1 | -0.347 | 0.047254331 |
| RNU6-1143P | PMVK | -0.346 | 0.047894425 |
| RNU6-1143P | STK16 | -0.346 | 0.047894425 |
| RNU6-1143P | IGSF8 | -0.346 | 0.047894425 |
| RNU6-1143P | MRPL2 | -0.346 | 0.047894425 |
| RNU6-1143P | STOML2 | -0.346 | 0.047894425 |
| RNU6-1143P | NOC4L | -0.346 | 0.047894425 |
| RNU6-1143P | PEMT | -0.345 | 0.048541791 |
| RNU6-1143P | CHMP1A | -0.345 | 0.048541791 |
| RNU6-1143P | CIAPIN1 | -0.345 | 0.048541791 |
| RNU6-1143P | HDHD3 | -0.345 | 0.048541791 |
| RNU6-1143P | NDUFS3 | -0.345 | 0.048541791 |
| RNU6-1143P | WDR77 | -0.344 | 0.049196488 |
| RNU6-1143P | PSMD3 | -0.344 | 0.049196488 |
| RNU6-1143P | METTL26 | -0.344 | 0.049196488 |
| RNU6-1143P | NOL6 | -0.344 | 0.049196488 |
| RNU6-1143P | ZNF783 | -0.344 | 0.049196488 |
| RNU6-1143P | ABHD17A | -0.344 | 0.049196488 |
| RNU6-1143P | RAB1B | -0.344 | 0.049196488 |
| RNU6-1143P | CISD3 | -0.344 | 0.049196488 |
| RNU6-1143P | MRPL4 | -0.344 | 0.049196488 |
| RNU6-1143P | NIT1 | -0.343 | 0.04985858 |
| RNU6-1143P | SH2B1 | -0.343 | 0.04985858 |
| RNU6-1143P | ZNF865 | -0.343 | 0.04985858 |
| RNU6-1143P | SBF1 | -0.343 | 0.04985858 |
| RNU6-1143P | TMEM185A | -0.343 | 0.04985858 |
| RNU6-1143P | ENKD1 | -0.343 | 0.04985858 |
| RNU6-1143P | CCDC107 | -0.343 | 0.04985858 |
| RNU6-1143P | FAM173A | -0.343 | 0.04985858 |
| RNU6-1143P | FLAD1 | -0.343 | 0.04985858 |
| RNU6-1143P | THAP5 | 0.344 | 0.049844475 |
| RNU6-1143P | TMPO | 0.344 | 0.049844475 |
| RNU6-1143P | GPR52 | 0.344 | 0.049844475 |
| RNU6-1143P | LRCH1 | 0.344 | 0.049844475 |
| RNU6-1143P | FCHSD2 | 0.344 | 0.049844475 |
| RNU6-1143P | ANAPC10 | 0.345 | 0.049182541 |
| RNU6-1143P | NPAT | 0.345 | 0.049182541 |
| RNU6-1143P | INTS2 | 0.345 | 0.049182541 |
| RNU6-1143P | ANGPT2 | 0.345 | 0.049182541 |
| RNU6-1143P | CSNK2A3 | 0.345 | 0.049182541 |
| RNU6-1143P | RSBN1L | 0.345 | 0.049182541 |
| RNU6-1143P | MBTD1 | 0.345 | 0.049182541 |
| RNU6-1143P | PPM1B | 0.345 | 0.049182541 |
| RNU6-1143P | FRY | 0.345 | 0.049182541 |
| RNU6-1143P | PRSS56 | 0.345 | 0.049182541 |
| RNU6-1143P | PCDHB1 | 0.346 | 0.048527999 |
| RNU6-1143P | CYB5R4 | 0.346 | 0.048527999 |
| RNU6-1143P | AP4E1 | 0.346 | 0.048527999 |
| RNU6-1143P | KIF11 | 0.346 | 0.048527999 |
| RNU6-1143P | MSH3 | 0.346 | 0.048527999 |
| RNU6-1143P | RGS20 | 0.346 | 0.048527999 |
| RNU6-1143P | BIRC6 | 0.346 | 0.048527999 |
| RNU6-1143P | TEFM | 0.346 | 0.048527999 |
| RNU6-1143P | RUNDC3B | 0.346 | 0.048527999 |
| RNU6-1143P | UBN2 | 0.347 | 0.047880788 |
| RNU6-1143P | ANKS1B | 0.347 | 0.047880788 |
| RNU6-1143P | PDZD9 | 0.347 | 0.047880788 |
| RNU6-1143P | CEP290 | 0.347 | 0.047880788 |
| RNU6-1143P | CKMT2 | 0.348 | 0.047240847 |
| RNU6-1143P | NKX6-2 | 0.348 | 0.047240847 |
| RNU6-1143P | MAK | 0.348 | 0.047240847 |
| RNU6-1143P | PKD2L2 | 0.348 | 0.047240847 |
| RNU6-1143P | TMTC3 | 0.348 | 0.047240847 |
| RNU6-1143P | ZNF506 | 0.348 | 0.047240847 |
| RNU6-1143P | HELZ | 0.348 | 0.047240847 |
| RNU6-1143P | ZFP14 | 0.348 | 0.047240847 |
| RNU6-1143P | UBR3 | 0.348 | 0.047240847 |
| RNU6-1143P | POLQ | 0.348 | 0.047240847 |
| RNU6-1143P | ACSM2B | 0.348 | 0.047240847 |
| RNU6-1143P | KLHL20 | 0.348 | 0.047240847 |
| RNU6-1143P | FAM208A | 0.348 | 0.047240847 |
| RNU6-1143P | SECISBP2 | 0.348 | 0.047240847 |
| RNU6-1143P | HNRNPA3 | 0.348 | 0.047240847 |
| RNU6-1143P | CCDC88A | 0.349 | 0.046608115 |
| RNU6-1143P | RFTN2 | 0.349 | 0.046608115 |
| RNU6-1143P | LNPEP | 0.349 | 0.046608115 |
| RNU6-1143P | EIF4E | 0.349 | 0.046608115 |
| RNU6-1143P | MTFR1 | 0.349 | 0.046608115 |
| RNU6-1143P | POLE2 | 0.349 | 0.046608115 |
| RNU6-1143P | CA5A | 0.349 | 0.046608115 |
| RNU6-1143P | COL4A4 | 0.35 | 0.04598253 |
| RNU6-1143P | AC013394.1 | 0.35 | 0.04598253 |
| RNU6-1143P | AQP11 | 0.35 | 0.04598253 |
| RNU6-1143P | SMAD2 | 0.35 | 0.04598253 |
| RNU6-1143P | LIN9 | 0.35 | 0.04598253 |
| RNU6-1143P | PROCR | 0.35 | 0.04598253 |
| RNU6-1143P | PPP1R12A | 0.351 | 0.045364033 |
| RNU6-1143P | STAG1 | 0.351 | 0.045364033 |
| RNU6-1143P | KLHL28 | 0.351 | 0.045364033 |
| RNU6-1143P | C1QTNF7 | 0.351 | 0.045364033 |
| RNU6-1143P | BRCA1 | 0.351 | 0.045364033 |
| RNU6-1143P | CDK19 | 0.351 | 0.045364033 |
| RNU6-1143P | C11orf65 | 0.351 | 0.045364033 |
| RNU6-1143P | PPIAL4G | 0.351 | 0.045364033 |
| RNU6-1143P | ZNF700 | 0.351 | 0.045364033 |
| RNU6-1143P | PPIL4 | 0.352 | 0.044752563 |
| RNU6-1143P | TEX12 | 0.352 | 0.044752563 |
| RNU6-1143P | MTRNR2L4 | 0.352 | 0.044752563 |
| RNU6-1143P | SENP1 | 0.352 | 0.044752563 |
| RNU6-1143P | PHF20L1 | 0.352 | 0.044752563 |
| RNU6-1143P | UBR2 | 0.352 | 0.044752563 |
| RNU6-1143P | WAPL | 0.352 | 0.044752563 |
| RNU6-1143P | MCPH1 | 0.352 | 0.044752563 |
| RNU6-1143P | TAOK1 | 0.353 | 0.044148059 |
| RNU6-1143P | CC2D2A | 0.353 | 0.044148059 |
| RNU6-1143P | GOSR1 | 0.353 | 0.044148059 |
| RNU6-1143P | ANKRD36 | 0.353 | 0.044148059 |
| RNU6-1143P | ACSM2A | 0.353 | 0.044148059 |
| RNU6-1143P | INSL6 | 0.353 | 0.044148059 |
| RNU6-1143P | ZNF236 | 0.353 | 0.044148059 |
| RNU6-1143P | EEF1AKMT2 | 0.354 | 0.043550462 |
| RNU6-1143P | NT5E | 0.354 | 0.043550462 |
| RNU6-1143P | ZBTB20 | 0.354 | 0.043550462 |
| RNU6-1143P | SPRTN | 0.354 | 0.043550462 |
| RNU6-1143P | KIAA0391 | 0.355 | 0.042959713 |
| RNU6-1143P | MT-ATP8 | 0.355 | 0.042959713 |
| RNU6-1143P | CHORDC1 | 0.355 | 0.042959713 |
| RNU6-1143P | JPH3 | 0.355 | 0.042959713 |
| RNU6-1143P | ZNF267 | 0.355 | 0.042959713 |
| RNU6-1143P | S100G | 0.355 | 0.042959713 |
| RNU6-1143P | THOC1 | 0.356 | 0.042375751 |
| RNU6-1143P | RABEP1 | 0.356 | 0.042375751 |
| RNU6-1143P | ZNF675 | 0.356 | 0.042375751 |
| RNU6-1143P | UTRN | 0.356 | 0.042375751 |
| RNU6-1143P | EPC1 | 0.356 | 0.042375751 |
| RNU6-1143P | RNU6-1272P | 0.356 | 0.042375751 |
| RNU6-1143P | ANKRD30BL | 0.357 | 0.041798518 |
| RNU6-1143P | DNAJC24 | 0.357 | 0.041798518 |
| RNU6-1143P | EHF | 0.357 | 0.041798518 |
| RNU6-1143P | PTPN4 | 0.357 | 0.041798518 |
| RNU6-1143P | NEDD1 | 0.357 | 0.041798518 |
| RNU6-1143P | ZNF37A | 0.357 | 0.041798518 |
| RNU6-1143P | FANCD2OS | 0.357 | 0.041798518 |
| RNU6-1143P | C5orf58 | 0.357 | 0.041798518 |
| RNU6-1143P | ZNF788 | 0.357 | 0.041798518 |
| RNU6-1143P | TAF1 | 0.358 | 0.041227955 |
| RNU6-1143P | TEX10 | 0.358 | 0.041227955 |
| RNU6-1143P | CCDC38 | 0.358 | 0.041227955 |
| RNU6-1143P | LMBR1 | 0.358 | 0.041227955 |
| RNU6-1143P | ERC1 | 0.358 | 0.041227955 |
| RNU6-1143P | ZNF350 | 0.359 | 0.040664003 |
| RNU6-1143P | XPA | 0.359 | 0.040664003 |
| RNU6-1143P | ZNF20 | 0.359 | 0.040664003 |
| RNU6-1143P | PFN4 | 0.359 | 0.040664003 |
| RNU6-1143P | PLEKHG7 | 0.359 | 0.040664003 |
| RNU6-1143P | KRR1 | 0.359 | 0.040664003 |
| RNU6-1143P | ERCC6 | 0.359 | 0.040664003 |
| RNU6-1143P | 7-Mar | 0.36 | 0.040106603 |
| RNU6-1143P | CENPI | 0.36 | 0.040106603 |
| RNU6-1143P | SLC4A7 | 0.36 | 0.040106603 |
| RNU6-1143P | AL110118.2 | 0.36 | 0.040106603 |
| RNU6-1143P | SLC10A7 | 0.36 | 0.040106603 |
| RNU6-1143P | SLC38A11 | 0.36 | 0.040106603 |
| RNU6-1143P | ZCCHC11 | 0.36 | 0.040106603 |
| RNU6-1143P | TRAPPC13 | 0.36 | 0.040106603 |
| RNU6-1143P | OR1L3 | 0.36 | 0.040106603 |
| RNU6-1143P | CNOT6 | 0.36 | 0.040106603 |
| RNU6-1143P | USP33 | 0.361 | 0.039555698 |
| RNU6-1143P | ZNF680 | 0.361 | 0.039555698 |
| RNU6-1143P | SCAF11 | 0.361 | 0.039555698 |
| RNU6-1143P | AC079447.1 | 0.361 | 0.039555698 |
| RNU6-1143P | TBC1D19 | 0.361 | 0.039555698 |
| RNU6-1143P | COL10A1 | 0.361 | 0.039555698 |
| RNU6-1143P | IFNK | 0.362 | 0.039011229 |
| RNU6-1143P | SGO1 | 0.362 | 0.039011229 |
| RNU6-1143P | SPICE1 | 0.362 | 0.039011229 |
| RNU6-1143P | C3orf49 | 0.363 | 0.03847314 |
| RNU6-1143P | STPG4 | 0.363 | 0.03847314 |
| RNU6-1143P | XIAP | 0.363 | 0.03847314 |
| RNU6-1143P | EXOC6 | 0.363 | 0.03847314 |
| RNU6-1143P | CEP295NL | 0.363 | 0.03847314 |
| RNU6-1143P | KLHL32 | 0.363 | 0.03847314 |
| RNU6-1143P | NVL | 0.364 | 0.037941371 |
| RNU6-1143P | SLC25A16 | 0.364 | 0.037941371 |
| RNU6-1143P | LCTL | 0.364 | 0.037941371 |
| RNU6-1143P | NUP58 | 0.364 | 0.037941371 |
| RNU6-1143P | SUCO | 0.364 | 0.037941371 |
| RNU6-1143P | TRIM13 | 0.365 | 0.037415867 |
| RNU6-1143P | ZFP69B | 0.365 | 0.037415867 |
| RNU6-1143P | AC012309.1 | 0.365 | 0.037415867 |
| RNU6-1143P | PHKB | 0.366 | 0.03689657 |
| RNU6-1143P | PDE12 | 0.366 | 0.03689657 |
| RNU6-1143P | STK3 | 0.366 | 0.03689657 |
| RNU6-1143P | RNF115 | 0.366 | 0.03689657 |
| RNU6-1143P | ERCC6L | 0.366 | 0.03689657 |
| RNU6-1143P | ANKHD1-EIF4EBP3 | 0.367 | 0.036383425 |
| RNU6-1143P | SGK3 | 0.367 | 0.036383425 |
| RNU6-1143P | C14orf105 | 0.367 | 0.036383425 |
| RNU6-1143P | SBNO1 | 0.367 | 0.036383425 |
| RNU6-1143P | L2HGDH | 0.368 | 0.035876373 |
| RNU6-1143P | FBXL20 | 0.368 | 0.035876373 |
| RNU6-1143P | UHRF1BP1 | 0.368 | 0.035876373 |
| RNU6-1143P | ZNF433 | 0.368 | 0.035876373 |
| RNU6-1143P | VCPKMT | 0.368 | 0.035876373 |
| RNU6-1143P | MDM4 | 0.368 | 0.035876373 |
| RNU6-1143P | ACMSD | 0.368 | 0.035876373 |
| RNU6-1143P | SENP5 | 0.368 | 0.035876373 |
| RNU6-1143P | KIAA2026 | 0.368 | 0.035876373 |
| RNU6-1143P | ZNF227 | 0.368 | 0.035876373 |
| RNU6-1143P | DHX36 | 0.369 | 0.035375361 |
| RNU6-1143P | CCDC125 | 0.369 | 0.035375361 |
| RNU6-1143P | APC | 0.369 | 0.035375361 |
| RNU6-1143P | FCGBP | 0.369 | 0.035375361 |
| RNU6-1143P | TMOD3 | 0.369 | 0.035375361 |
| RNU6-1143P | SLC35A3 | 0.37 | 0.034880331 |
| RNU6-1143P | ATRX | 0.37 | 0.034880331 |
| RNU6-1143P | NF1 | 0.371 | 0.034391228 |
| RNU6-1143P | MPHOSPH9 | 0.371 | 0.034391228 |
| RNU6-1143P | TERF1 | 0.371 | 0.034391228 |
| RNU6-1143P | AP002495.2 | 0.371 | 0.034391228 |
| RNU6-1143P | NAP1L5 | 0.371 | 0.034391228 |
| RNU6-1143P | PRPF40A | 0.372 | 0.033907997 |
| RNU6-1143P | PLEKHA3 | 0.372 | 0.033907997 |
| RNU6-1143P | WASL | 0.372 | 0.033907997 |
| RNU6-1143P | CXCR6 | 0.372 | 0.033907997 |
| RNU6-1143P | PIAS2 | 0.372 | 0.033907997 |
| RNU6-1143P | KANSL1 | 0.372 | 0.033907997 |
| RNU6-1143P | PSMA3 | 0.373 | 0.033430583 |
| RNU6-1143P | ANGPTL3 | 0.373 | 0.033430583 |
| RNU6-1143P | CCSAP | 0.373 | 0.033430583 |
| RNU6-1143P | CHD1 | 0.374 | 0.032958932 |
| RNU6-1143P | ATP13A3 | 0.374 | 0.032958932 |
| RNU6-1143P | ZNF519 | 0.374 | 0.032958932 |
| RNU6-1143P | PDGFC | 0.374 | 0.032958932 |
| RNU6-1143P | ERGIC2 | 0.375 | 0.032492989 |
| RNU6-1143P | ARID4B | 0.375 | 0.032492989 |
| RNU6-1143P | HERC4 | 0.375 | 0.032492989 |
| RNU6-1143P | ATP8B1 | 0.375 | 0.032492989 |
| RNU6-1143P | GPR22 | 0.375 | 0.032492989 |
| RNU6-1143P | ZNF292 | 0.375 | 0.032492989 |
| RNU6-1143P | COBLL1 | 0.376 | 0.032032699 |
| RNU6-1143P | RGPD5 | 0.376 | 0.032032699 |
| RNU6-1143P | RBAK | 0.376 | 0.032032699 |
| RNU6-1143P | AVL9 | 0.376 | 0.032032699 |
| RNU6-1143P | MED13 | 0.377 | 0.03157801 |
| RNU6-1143P | IGF2BP1 | 0.377 | 0.03157801 |
| RNU6-1143P | NUP155 | 0.377 | 0.03157801 |
| RNU6-1143P | ATP9B | 0.377 | 0.03157801 |
| RNU6-1143P | MSH6 | 0.377 | 0.03157801 |
| RNU6-1143P | GFPT1 | 0.377 | 0.03157801 |
| RNU6-1143P | MTRNR2L13 | 0.377 | 0.03157801 |
| RNU6-1143P | PARPBP | 0.378 | 0.031128868 |
| RNU6-1143P | KNTC1 | 0.379 | 0.030685219 |
| RNU6-1143P | ORC2 | 0.379 | 0.030685219 |
| RNU6-1143P | C1orf27 | 0.379 | 0.030685219 |
| RNU6-1143P | INSM2 | 0.379 | 0.030685219 |
| RNU6-1143P | KLB | 0.379 | 0.030685219 |
| RNU6-1143P | TRDMT1 | 0.379 | 0.030685219 |
| RNU6-1143P | RNGTT | 0.38 | 0.03024701 |
| RNU6-1143P | MCC | 0.38 | 0.03024701 |
| RNU6-1143P | ZNF235 | 0.38 | 0.03024701 |
| RNU6-1143P | DENND4A | 0.38 | 0.03024701 |
| RNU6-1143P | ZMYM2 | 0.38 | 0.03024701 |
| RNU6-1143P | BLM | 0.381 | 0.02981419 |
| RNU6-1143P | DNAJB14 | 0.381 | 0.02981419 |
| RNU6-1143P | ZNF638 | 0.381 | 0.02981419 |
| RNU6-1143P | ATXN7 | 0.381 | 0.02981419 |
| RNU6-1143P | ATF7IP | 0.382 | 0.029386705 |
| RNU6-1143P | CTAGE5 | 0.382 | 0.029386705 |
| RNU6-1143P | WDR76 | 0.382 | 0.029386705 |
| RNU6-1143P | SLC30A7 | 0.383 | 0.028964504 |
| RNU6-1143P | MCM9 | 0.383 | 0.028964504 |
| RNU6-1143P | ARHGEF38 | 0.383 | 0.028964504 |
| RNU6-1143P | ZNF43 | 0.383 | 0.028964504 |
| RNU6-1143P | NSUN3 | 0.384 | 0.028547535 |
| RNU6-1143P | EEA1 | 0.384 | 0.028547535 |
| RNU6-1143P | ACKR4 | 0.384 | 0.028547535 |
| RNU6-1143P | C9orf3 | 0.384 | 0.028547535 |
| RNU6-1143P | MTBP | 0.384 | 0.028547535 |
| RNU6-1143P | JMJD1C | 0.384 | 0.028547535 |
| RNU6-1143P | ARID4A | 0.385 | 0.028135746 |
| RNU6-1143P | PIAS1 | 0.385 | 0.028135746 |
| RNU6-1143P | CEP78 | 0.385 | 0.028135746 |
| RNU6-1143P | PTPN13 | 0.385 | 0.028135746 |
| RNU6-1143P | RALGAPA1 | 0.385 | 0.028135746 |
| RNU6-1143P | ARIH1 | 0.386 | 0.027729087 |
| RNU6-1143P | SUZ12 | 0.386 | 0.027729087 |
| RNU6-1143P | SLC35G3 | 0.386 | 0.027729087 |
| RNU6-1143P | RBM41 | 0.386 | 0.027729087 |
| RNU6-1143P | AP003108.2 | 0.387 | 0.027327507 |
| RNU6-1143P | ZNF254 | 0.387 | 0.027327507 |
| RNU6-1143P | RLIM | 0.388 | 0.026930954 |
| RNU6-1143P | ZNF562 | 0.388 | 0.026930954 |
| RNU6-1143P | TLK2 | 0.388 | 0.026930954 |
| RNU6-1143P | PDC | 0.389 | 0.02653938 |
| RNU6-1143P | CYP3A5 | 0.389 | 0.02653938 |
| RNU6-1143P | USP34 | 0.389 | 0.02653938 |
| RNU6-1143P | UBE2V1 | 0.39 | 0.026152733 |
| RNU6-1143P | ZGRF1 | 0.39 | 0.026152733 |
| RNU6-1143P | ZNF326 | 0.39 | 0.026152733 |
| RNU6-1143P | AC008575.2 | 0.39 | 0.026152733 |
| RNU6-1143P | AC005520.1 | 0.39 | 0.026152733 |
| RNU6-1143P | FBXL4 | 0.39 | 0.026152733 |
| RNU6-1143P | POLK | 0.39 | 0.026152733 |
| RNU6-1143P | KSR2 | 0.391 | 0.025770965 |
| RNU6-1143P | B3GALT2 | 0.391 | 0.025770965 |
| RNU6-1143P | AL049844.1 | 0.391 | 0.025770965 |
| RNU6-1143P | RNF169 | 0.391 | 0.025770965 |
| RNU6-1143P | SPIN2A | 0.392 | 0.025394026 |
| RNU6-1143P | NOSTRIN | 0.392 | 0.025394026 |
| RNU6-1143P | ANKRD36C | 0.392 | 0.025394026 |
| RNU6-1143P | ZNF26 | 0.392 | 0.025394026 |
| RNU6-1143P | DPP8 | 0.392 | 0.025394026 |
| RNU6-1143P | PRH1 | 0.392 | 0.025394026 |
| RNU6-1143P | NSMCE2 | 0.392 | 0.025394026 |
| RNU6-1143P | HDAC9 | 0.393 | 0.025021868 |
| RNU6-1143P | RASA1 | 0.393 | 0.025021868 |
| RNU6-1143P | TROVE2 | 0.393 | 0.025021868 |
| RNU6-1143P | SUDS3 | 0.393 | 0.025021868 |
| RNU6-1143P | AP3B1 | 0.393 | 0.025021868 |
| RNU6-1143P | TVP23C | 0.393 | 0.025021868 |
| RNU6-1143P | CD1B | 0.393 | 0.025021868 |
| RNU6-1143P | MAPKAPK5 | 0.394 | 0.024654441 |
| RNU6-1143P | SLF2 | 0.394 | 0.024654441 |
| RNU6-1143P | GJA9 | 0.394 | 0.024654441 |
| RNU6-1143P | SCGB3A2 | 0.394 | 0.024654441 |
| RNU6-1143P | EMSY | 0.395 | 0.024291697 |
| RNU6-1143P | KMT2C | 0.395 | 0.024291697 |
| RNU6-1143P | SETD2 | 0.395 | 0.024291697 |
| RNU6-1143P | TMEM161B | 0.396 | 0.023933589 |
| RNU6-1143P | ALMS1 | 0.396 | 0.023933589 |
| RNU6-1143P | TMF1 | 0.397 | 0.023580068 |
| RNU6-1143P | C5orf42 | 0.397 | 0.023580068 |
| RNU6-1143P | MTMR7 | 0.397 | 0.023580068 |
| RNU6-1143P | UBR1 | 0.397 | 0.023580068 |
| RNU6-1143P | AKAP9 | 0.397 | 0.023580068 |
| RNU6-1143P | ITCH | 0.397 | 0.023580068 |
| RNU6-1143P | ECD | 0.398 | 0.023231087 |
| RNU6-1143P | XRN1 | 0.398 | 0.023231087 |
| RNU6-1143P | ATXN2 | 0.398 | 0.023231087 |
| RNU6-1143P | CPNE6 | 0.398 | 0.023231087 |
| RNU6-1143P | NKIRAS1 | 0.399 | 0.0228866 |
| RNU6-1143P | RARS2 | 0.399 | 0.0228866 |
| RNU6-1143P | MYO9A | 0.399 | 0.0228866 |
| RNU6-1143P | VPS13B | 0.399 | 0.0228866 |
| RNU6-1143P | TAS2R43 | 0.399 | 0.0228866 |
| RNU6-1143P | TUBGCP3 | 0.399 | 0.0228866 |
| RNU6-1143P | FER1L6 | 0.399 | 0.0228866 |
| RNU6-1143P | PP2D1 | 0.4 | 0.022546559 |
| RNU6-1143P | CASP8AP2 | 0.4 | 0.022546559 |
| RNU6-1143P | CNNM2 | 0.4 | 0.022546559 |
| RNU6-1143P | KNL1 | 0.4 | 0.022546559 |
| RNU6-1143P | RBL1 | 0.4 | 0.022546559 |
| RNU6-1143P | TDRD15 | 0.4 | 0.022546559 |
| RNU6-1143P | CHD9 | 0.401 | 0.022210917 |
| RNU6-1143P | CNTRL | 0.401 | 0.022210917 |
| RNU6-1143P | SCFD1 | 0.401 | 0.022210917 |
| RNU6-1143P | A1CF | 0.401 | 0.022210917 |
| RNU6-1143P | XPO4 | 0.401 | 0.022210917 |
| RNU6-1143P | ZNF410 | 0.401 | 0.022210917 |
| RNU6-1143P | WDPCP | 0.402 | 0.021879629 |
| RNU6-1143P | BBIP1 | 0.402 | 0.021879629 |
| RNU6-1143P | WDR33 | 0.402 | 0.021879629 |
| RNU6-1143P | SNX13 | 0.402 | 0.021879629 |
| RNU6-1143P | CEP192 | 0.403 | 0.02155265 |
| RNU6-1143P | USP8 | 0.403 | 0.02155265 |
| RNU6-1143P | FAM217A | 0.403 | 0.02155265 |
| RNU6-1143P | BRCA2 | 0.403 | 0.02155265 |
| RNU6-1143P | ABHD18 | 0.404 | 0.021229932 |
| RNU6-1143P | BRIP1 | 0.404 | 0.021229932 |
| RNU6-1143P | ANKRD12 | 0.404 | 0.021229932 |
| RNU6-1143P | ELF2 | 0.404 | 0.021229932 |
| RNU6-1143P | ZNF708 | 0.404 | 0.021229932 |
| RNU6-1143P | TSPYL6 | 0.404 | 0.021229932 |
| RNU6-1143P | REV3L | 0.405 | 0.020911432 |
| RNU6-1143P | CCDC66 | 0.405 | 0.020911432 |
| RNU6-1143P | TNRC6B | 0.405 | 0.020911432 |
| RNU6-1143P | MIS18BP1 | 0.406 | 0.020597105 |
| RNU6-1143P | BPTF | 0.406 | 0.020597105 |
| RNU6-1143P | TNFRSF19 | 0.406 | 0.020597105 |
| RNU6-1143P | PWWP2A | 0.406 | 0.020597105 |
| RNU6-1143P | UBE2W | 0.407 | 0.020286905 |
| RNU6-1143P | DENND1B | 0.407 | 0.020286905 |
| RNU6-1143P | TAF1L | 0.407 | 0.020286905 |
| RNU6-1143P | ZNF100 | 0.408 | 0.019980788 |
| RNU6-1143P | ESCO1 | 0.408 | 0.019980788 |
| RNU6-1143P | ZNF808 | 0.408 | 0.019980788 |
| RNU6-1143P | SMIM18 | 0.408 | 0.019980788 |
| RNU6-1143P | NFATC3 | 0.409 | 0.019678712 |
| RNU6-1143P | ZNF724 | 0.409 | 0.019678712 |
| RNU6-1143P | CEP135 | 0.409 | 0.019678712 |
| RNU6-1143P | ZNF670-ZNF695 | 0.409 | 0.019678712 |
| RNU6-1143P | PRR16 | 0.41 | 0.019380631 |
| RNU6-1143P | FAM133B | 0.41 | 0.019380631 |
| RNU6-1143P | VPS54 | 0.411 | 0.019086504 |
| RNU6-1143P | PHF3 | 0.411 | 0.019086504 |
| RNU6-1143P | FANCM | 0.411 | 0.019086504 |
| RNU6-1143P | EFCAB13 | 0.411 | 0.019086504 |
| RNU6-1143P | ZNF720 | 0.411 | 0.019086504 |
| RNU6-1143P | PAPD4 | 0.411 | 0.019086504 |
| RNU6-1143P | CC2D2B | 0.412 | 0.018796286 |
| RNU6-1143P | BRWD3 | 0.412 | 0.018796286 |
| RNU6-1143P | ARHGAP15 | 0.412 | 0.018796286 |
| RNU6-1143P | ZNF407 | 0.412 | 0.018796286 |
| RNU6-1143P | HMCN2 | 0.413 | 0.018509936 |
| RNU6-1143P | HECTD1 | 0.413 | 0.018509936 |
| RNU6-1143P | DPP6 | 0.413 | 0.018509936 |
| RNU6-1143P | CEP97 | 0.414 | 0.01822741 |
| RNU6-1143P | ANKAR | 0.414 | 0.01822741 |
| RNU6-1143P | PSIP1 | 0.414 | 0.01822741 |
| RNU6-1143P | PPP2R5E | 0.415 | 0.017948667 |
| RNU6-1143P | NAA15 | 0.415 | 0.017948667 |
| RNU6-1143P | CUL3 | 0.415 | 0.017948667 |
| RNU6-1143P | COA1 | 0.416 | 0.017673666 |
| RNU6-1143P | AHCTF1 | 0.416 | 0.017673666 |
| RNU6-1143P | BARD1 | 0.418 | 0.017134721 |
| RNU6-1143P | CRLF3 | 0.418 | 0.017134721 |
| RNU6-1143P | FAM227B | 0.418 | 0.017134721 |
| RNU6-1143P | RAD51B | 0.419 | 0.016870697 |
| RNU6-1143P | ZNF674 | 0.419 | 0.016870697 |
| RNU6-1143P | ZNF148 | 0.419 | 0.016870697 |
| RNU6-1143P | ERCC6L2 | 0.419 | 0.016870697 |
| RNU6-1143P | PVRIG | 0.421 | 0.01635334 |
| RNU6-1143P | FAM196B | 0.421 | 0.01635334 |
| RNU6-1143P | SYCP3 | 0.421 | 0.01635334 |
| RNU6-1143P | AL138752.2 | 0.421 | 0.01635334 |
| RNU6-1143P | SLC35F5 | 0.421 | 0.01635334 |
| RNU6-1143P | AP001931.1 | 0.421 | 0.01635334 |
| RNU6-1143P | SASS6 | 0.421 | 0.01635334 |
| RNU6-1143P | CEP350 | 0.421 | 0.01635334 |
| RNU6-1143P | SMIM11B | 0.422 | 0.016099928 |
| RNU6-1143P | N4BP2 | 0.422 | 0.016099928 |
| RNU6-1143P | SRFBP1 | 0.423 | 0.015849974 |
| RNU6-1143P | RAPGEF6 | 0.423 | 0.015849974 |
| RNU6-1143P | ZNF91 | 0.423 | 0.015849974 |
| RNU6-1143P | MATR3 | 0.423 | 0.015849974 |
| RNU6-1143P | SUPT3H | 0.423 | 0.015849974 |
| RNU6-1143P | HTD2 | 0.425 | 0.015360286 |
| RNU6-1143P | CENPK | 0.425 | 0.015360286 |
| RNU6-1143P | ANKUB1 | 0.426 | 0.015120473 |
| RNU6-1143P | PIK3C2A | 0.426 | 0.015120473 |
| RNU6-1143P | RARRES1 | 0.426 | 0.015120473 |
| RNU6-1143P | RPS6KB1 | 0.426 | 0.015120473 |
| RNU6-1143P | AGO3 | 0.427 | 0.014883964 |
| RNU6-1143P | NME9 | 0.427 | 0.014883964 |
| RNU6-1143P | SLF1 | 0.428 | 0.014650721 |
| RNU6-1143P | TYW5 | 0.428 | 0.014650721 |
| RNU6-1143P | FOXP1 | 0.428 | 0.014650721 |
| RNU6-1143P | N4BP2L2 | 0.429 | 0.014420706 |
| RNU6-1143P | ATL1 | 0.43 | 0.014193882 |
| RNU6-1143P | POU2F1 | 0.43 | 0.014193882 |
| RNU6-1143P | ZNF430 | 0.43 | 0.014193882 |
| RNU6-1143P | PDS5A | 0.431 | 0.013970211 |
| RNU6-1143P | MAP4K5 | 0.431 | 0.013970211 |
| RNU6-1143P | ASB14 | 0.431 | 0.013970211 |
| RNU6-1143P | PMM2 | 0.431 | 0.013970211 |
| RNU6-1143P | ULK4 | 0.432 | 0.013749657 |
| RNU6-1143P | GPSM2 | 0.432 | 0.013749657 |
| RNU6-1143P | TLK1 | 0.432 | 0.013749657 |
| RNU6-1143P | POC1B | 0.433 | 0.013532184 |
| RNU6-1143P | SPDYA | 0.433 | 0.013532184 |
| RNU6-1143P | CUL5 | 0.434 | 0.013317756 |
| RNU6-1143P | MON2 | 0.434 | 0.013317756 |
| RNU6-1143P | TRIM33 | 0.435 | 0.013106337 |
| RNU6-1143P | GIGYF2 | 0.435 | 0.013106337 |
| RNU6-1143P | BRAF | 0.436 | 0.012897892 |
| RNU6-1143P | YY2 | 0.436 | 0.012897892 |
| RNU6-1143P | MKLN1 | 0.436 | 0.012897892 |
| RNU6-1143P | TAS2R38 | 0.436 | 0.012897892 |
| RNU6-1143P | FAM177A1 | 0.437 | 0.012692385 |
| RNU6-1143P | FRYL | 0.437 | 0.012692385 |
| RNU6-1143P | SLC38A9 | 0.437 | 0.012692385 |
| RNU6-1143P | USP37 | 0.437 | 0.012692385 |
| RNU6-1143P | DENND6A | 0.438 | 0.012489781 |
| RNU6-1143P | ZNF66 | 0.438 | 0.012489781 |
| RNU6-1143P | NEMP2 | 0.438 | 0.012489781 |
| RNU6-1143P | ZNF644 | 0.438 | 0.012489781 |
| RNU6-1143P | GK3P | 0.438 | 0.012489781 |
| RNU6-1143P | NIPBL | 0.438 | 0.012489781 |
| RNU6-1143P | ACRV1 | 0.439 | 0.012290047 |
| RNU6-1143P | SPATA5 | 0.439 | 0.012290047 |
| RNU6-1143P | ANGPTL1 | 0.44 | 0.012093148 |
| RNU6-1143P | PHC3 | 0.44 | 0.012093148 |
| RNU6-1143P | TRPM7 | 0.441 | 0.011899049 |
| RNU6-1143P | MTRNR2L10 | 0.441 | 0.011899049 |
| RNU6-1143P | MTRNR2L5 | 0.441 | 0.011899049 |
| RNU6-1143P | YIPF4 | 0.442 | 0.011707718 |
| RNU6-1143P | AC087632.1 | 0.442 | 0.011707718 |
| RNU6-1143P | PIK3CA | 0.442 | 0.011707718 |
| RNU6-1143P | CNOT4 | 0.442 | 0.011707718 |
| RNU6-1143P | NAA16 | 0.442 | 0.011707718 |
| RNU6-1143P | ITGB3BP | 0.442 | 0.011707718 |
| RNU6-1143P | RTTN | 0.444 | 0.011333225 |
| RNU6-1143P | RIF1 | 0.445 | 0.011149998 |
| RNU6-1143P | MTRNR2L12 | 0.446 | 0.010969406 |
| RNU6-1143P | GPR18 | 0.447 | 0.010791418 |
| RNU6-1143P | LRRTM2 | 0.447 | 0.010791418 |
| RNU6-1143P | RC3H1 | 0.447 | 0.010791418 |
| RNU6-1143P | CEP57L1 | 0.447 | 0.010791418 |
| RNU6-1143P | RSF1 | 0.449 | 0.010443127 |
| RNU6-1143P | AKAP5 | 0.449 | 0.010443127 |
| RNU6-1143P | RAPGEF4 | 0.45 | 0.010272761 |
| RNU6-1143P | MPP5 | 0.45 | 0.010272761 |
| RNU6-1143P | SMCHD1 | 0.45 | 0.010272761 |
| RNU6-1143P | QSER1 | 0.45 | 0.010272761 |
| RNU6-1143P | LRRC19 | 0.451 | 0.010104873 |
| RNU6-1143P | SCAF8 | 0.451 | 0.010104873 |
| RNU6-1143P | TTLL6 | 0.452 | 0.009939433 |
| RNU6-1143P | CENPP | 0.452 | 0.009939433 |
| RNU6-1143P | GAPVD1 | 0.453 | 0.00977641 |
| RNU6-1143P | DNA2 | 0.453 | 0.00977641 |
| RNU6-1143P | F2RL2 | 0.454 | 0.009615774 |
| RNU6-1143P | CCDC18 | 0.455 | 0.009457495 |
| RNU6-1143P | CHM | 0.455 | 0.009457495 |
| RNU6-1143P | MUC5AC | 0.456 | 0.009301545 |
| RNU6-1143P | FHOD3 | 0.456 | 0.009301545 |
| RNU6-1143P | KRIT1 | 0.457 | 0.009147893 |
| RNU6-1143P | MTRNR2L11 | 0.458 | 0.008996511 |
| RNU6-1143P | DLG1 | 0.46 | 0.008700441 |
| RNU6-1143P | BAZ2B | 0.461 | 0.008555697 |
| RNU6-1143P | SERPIND1 | 0.462 | 0.008413109 |
| RNU6-1143P | LIN54 | 0.462 | 0.008413109 |
| RNU6-1143P | CENPC | 0.463 | 0.008272651 |
| RNU6-1143P | RICTOR | 0.464 | 0.008134295 |
| RNU6-1143P | ZNF750 | 0.465 | 0.007998013 |
| RNU6-1143P | MTRNR2L6 | 0.465 | 0.007998013 |
| RNU6-1143P | PRH2 | 0.465 | 0.007998013 |
| RNU6-1143P | EDDM13 | 0.466 | 0.00786378 |
| RNU6-1143P | ZRSR1 | 0.467 | 0.007731568 |
| RNU6-1143P | CEP295 | 0.467 | 0.007731568 |
| RNU6-1143P | TAS2R19 | 0.467 | 0.007731568 |
| RNU6-1143P | SPDYE5 | 0.467 | 0.007731568 |
| RNU6-1143P | PLG | 0.468 | 0.007601352 |
| RNU6-1143P | TAS2R30 | 0.468 | 0.007601352 |
| RNU6-1143P | CEP128 | 0.468 | 0.007601352 |
| RNU6-1143P | MEMO1 | 0.469 | 0.007473105 |
| RNU6-1143P | NUDT13 | 0.47 | 0.007346802 |
| RNU6-1143P | MTRNR2L7 | 0.471 | 0.007222417 |
| RNU6-1143P | HTR2B | 0.471 | 0.007222417 |
| RNU6-1143P | YAF2 | 0.471 | 0.007222417 |
| RNU6-1143P | GPR89A | 0.472 | 0.007099926 |
| RNU6-1143P | DLEU1 | 0.472 | 0.007099926 |
| RNU6-1143P | SCLT1 | 0.472 | 0.007099926 |
| RNU6-1143P | ZNF92 | 0.473 | 0.006979303 |
| RNU6-1143P | MTRNR2L3 | 0.473 | 0.006979303 |
| RNU6-1143P | LCORL | 0.473 | 0.006979303 |
| RNU6-1143P | AC093155.3 | 0.474 | 0.006860524 |
| RNU6-1143P | ARID2 | 0.475 | 0.006743564 |
| RNU6-1143P | RUFY2 | 0.475 | 0.006743564 |
| RNU6-1143P | BDP1 | 0.476 | 0.0066284 |
| RNU6-1143P | G2E3 | 0.476 | 0.0066284 |
| RNU6-1143P | SUMO4 | 0.477 | 0.006515007 |
| RNU6-1143P | KCNS2 | 0.478 | 0.006403361 |
| RNU6-1143P | KCTD4 | 0.48 | 0.006185221 |
| RNU6-1143P | NEMF | 0.481 | 0.00607868 |
| RNU6-1143P | METTL2B | 0.481 | 0.00607868 |
| RNU6-1143P | CCDC7 | 0.482 | 0.005973795 |
| RNU6-1143P | SPINK9 | 0.483 | 0.005870544 |
| RNU6-1143P | CDK12 | 0.485 | 0.005668853 |
| RNU6-1143P | GCNT7 | 0.486 | 0.00557037 |
| RNU6-1143P | CREB1 | 0.488 | 0.005378023 |
| RNU6-1143P | FNIP1 | 0.49 | 0.005191693 |
| RNU6-1143P | NAA25 | 0.491 | 0.005100733 |
| RNU6-1143P | PKN2 | 0.491 | 0.005100733 |
| RNU6-1143P | PHIP | 0.492 | 0.005011215 |
| RNU6-1143P | CTDSPL2 | 0.495 | 0.004751122 |
| RNU6-1143P | THADA | 0.498 | 0.004503308 |
| RNU6-1143P | SPATA1 | 0.501 | 0.004267263 |
| RNU6-1143P | OCLM | 0.502 | 0.00419111 |
| RNU6-1143P | LRCH3 | 0.503 | 0.004116193 |
| RNU6-1143P | MMS22L | 0.504 | 0.004042493 |
| RNU6-1143P | WDR20 | 0.508 | 0.003759519 |
| RNU6-1143P | ZNF136 | 0.51 | 0.003624893 |
| RNU6-1143P | RBM34 | 0.511 | 0.003559237 |
| RNU6-1143P | PDS5B | 0.515 | 0.003307294 |
| RNU6-1143P | FAM126B | 0.515 | 0.003307294 |
| RNU6-1143P | GLRB | 0.516 | 0.0032469 |
| RNU6-1143P | TAS2R20 | 0.517 | 0.003187513 |
| RNU6-1143P | FBXO11 | 0.517 | 0.003187513 |
| RNU6-1143P | ZNF124 | 0.519 | 0.003071698 |
| RNU6-1143P | SNURF | 0.521 | 0.002959733 |
| RNU6-1143P | MIGA1 | 0.521 | 0.002959733 |
| RNU6-1143P | ATAD5 | 0.521 | 0.002959733 |
| RNU6-1143P | FRMD5 | 0.524 | 0.002798756 |
| RNU6-1143P | TAS2R13 | 0.524 | 0.002798756 |
| RNU6-1143P | GOLGA4 | 0.525 | 0.0027469 |
| RNU6-1143P | SLMAP | 0.525 | 0.0027469 |
| RNU6-1143P | ANGPTL7 | 0.527 | 0.002645813 |
| RNU6-1143P | PRR4 | 0.527 | 0.002645813 |
| RNU6-1143P | GREB1L | 0.527 | 0.002645813 |
| RNU6-1143P | CDKAL1 | 0.531 | 0.002453769 |
| RNU6-1143P | ZNF611 | 0.531 | 0.002453769 |
| RNU6-1143P | ASH1L | 0.532 | 0.002407794 |
| RNU6-1143P | ZNF564 | 0.533 | 0.002362609 |
| RNU6-1143P | TAS2R31 | 0.536 | 0.002231672 |
| RNU6-1143P | DNAJB7 | 0.538 | 0.002148111 |
| RNU6-1143P | ATAD2B | 0.54 | 0.002067428 |
| RNU6-1143P | ANKHD1 | 0.544 | 0.001914342 |
| RNU6-1143P | SPDYE1 | 0.548 | 0.001771729 |
| RNU6-1143P | PIBF1 | 0.55 | 0.001704146 |
| RNU6-1143P | TAS2R14 | 0.554 | 0.001576041 |
| RNU6-1143P | OMG | 0.557 | 0.001485847 |
| RNU6-1143P | POU5F2 | 0.562 | 0.001346026 |
| RNU6-1143P | ZNF782 | 0.562 | 0.001346026 |
| RNU6-1143P | KCNJ13 | 0.565 | 0.001268067 |
| RNU6-1143P | TAS2R46 | 0.577 | 0.000996103 |
| RNU6-1143P | SLC16A4 | 0.579 | 0.000956413 |
| RNU6-1143P | TAS2R50 | 0.584 | 0.000863508 |
| RNU6-1143P | OGN | 0.621 | 0.000395834 |
| RNU6-1143P | ECM2 | 0.629 | 0.000332557 |
| RNU6-1143P | ASPN | 0.665 | 0.0001482 |
| RNU6-1143P | OMD | 0.666 | 0.000144827 |
| RNU6-1143P | RNU6-1143P | 1 | 1.15E-08 |
| RNU6-946P | DDX23 | -0.485 | 0.001011479 |
| RNU6-946P | PRPF4 | -0.481 | 0.001110829 |
| RNU6-946P | CSTF2 | -0.444 | 0.002561771 |
| RNU6-946P | CIAO1 | -0.44 | 0.002794588 |
| RNU6-946P | SRP68 | -0.428 | 0.003613719 |
| RNU6-946P | XRCC6 | -0.427 | 0.003690989 |
| RNU6-946P | EIF2B1 | -0.426 | 0.003769758 |
| RNU6-946P | SAMM50 | -0.411 | 0.005149474 |
| RNU6-946P | UNC45A | -0.408 | 0.005474927 |
| RNU6-946P | DCAF11 | -0.408 | 0.005474927 |
| RNU6-946P | RTCB | -0.401 | 0.006307646 |
| RNU6-946P | DCTN5 | -0.4 | 0.006435487 |
| RNU6-946P | STOML2 | -0.399 | 0.006565654 |
| RNU6-946P | SEC61A1 | -0.398 | 0.006698184 |
| RNU6-946P | VPS33B | -0.398 | 0.006698184 |
| RNU6-946P | COG4 | -0.397 | 0.006833114 |
| RNU6-946P | NDUFS2 | -0.396 | 0.006970481 |
| RNU6-946P | MIEF1 | -0.395 | 0.007110323 |
| RNU6-946P | ZNF622 | -0.394 | 0.007252679 |
| RNU6-946P | TUBA1B | -0.394 | 0.007252679 |
| RNU6-946P | EIF2B5 | -0.393 | 0.007397588 |
| RNU6-946P | EXO5 | -0.393 | 0.007397588 |
| RNU6-946P | COQ9 | -0.391 | 0.00769522 |
| RNU6-946P | DRG1 | -0.388 | 0.008161809 |
| RNU6-946P | CCT3 | -0.387 | 0.008322874 |
| RNU6-946P | SSRP1 | -0.387 | 0.008322874 |
| RNU6-946P | DHX16 | -0.385 | 0.00865356 |
| RNU6-946P | CINP | -0.385 | 0.00865356 |
| RNU6-946P | PHB | -0.383 | 0.008995941 |
| RNU6-946P | LAS1L | -0.382 | 0.009171626 |
| RNU6-946P | CYB5R1 | -0.38 | 0.00953221 |
| RNU6-946P | DCTN1 | -0.379 | 0.0097172 |
| RNU6-946P | APEH | -0.379 | 0.0097172 |
| RNU6-946P | EIF3I | -0.375 | 0.01048956 |
| RNU6-946P | ZNF32 | -0.375 | 0.01048956 |
| RNU6-946P | CIAPIN1 | -0.374 | 0.010690988 |
| RNU6-946P | CCT7 | -0.374 | 0.010690988 |
| RNU6-946P | ADSL | -0.372 | 0.011104189 |
| RNU6-946P | METTL13 | -0.372 | 0.011104189 |
| RNU6-946P | ROGDI | -0.37 | 0.011531516 |
| RNU6-946P | SNRNP200 | -0.369 | 0.011750603 |
| RNU6-946P | ASCC2 | -0.369 | 0.011750603 |
| RNU6-946P | AARS2 | -0.369 | 0.011750603 |
| RNU6-946P | TMEM203 | -0.369 | 0.011750603 |
| RNU6-946P | BCS1L | -0.368 | 0.011973374 |
| RNU6-946P | ALG1 | -0.365 | 0.012664315 |
| RNU6-946P | NOC2L | -0.365 | 0.012664315 |
| RNU6-946P | POP4 | -0.365 | 0.012664315 |
| RNU6-946P | MED8 | -0.363 | 0.013144332 |
| RNU6-946P | USP5 | -0.362 | 0.01339032 |
| RNU6-946P | VPS52 | -0.361 | 0.013640367 |
| RNU6-946P | RPN2 | -0.359 | 0.014152865 |
| RNU6-946P | PPP1R7 | -0.358 | 0.014415429 |
| RNU6-946P | EXOC7 | -0.357 | 0.01468228 |
| RNU6-946P | COPG1 | -0.356 | 0.014953476 |
| RNU6-946P | EXOSC10 | -0.356 | 0.014953476 |
| RNU6-946P | IKBKAP | -0.356 | 0.014953476 |
| RNU6-946P | SDHA | -0.355 | 0.015229075 |
| RNU6-946P | NME6 | -0.355 | 0.015229075 |
| RNU6-946P | HIRA | -0.354 | 0.015509137 |
| RNU6-946P | PDHA1 | -0.352 | 0.016082887 |
| RNU6-946P | ATG4A | -0.352 | 0.016082887 |
| RNU6-946P | IPO13 | -0.351 | 0.016376698 |
| RNU6-946P | HDLBP | -0.349 | 0.016978496 |
| RNU6-946P | GEMIN4 | -0.345 | 0.018240562 |
| RNU6-946P | HNRNPF | -0.345 | 0.018240562 |
| RNU6-946P | RRP36 | -0.344 | 0.018568632 |
| RNU6-946P | SUCLG1 | -0.344 | 0.018568632 |
| RNU6-946P | C6orf89 | -0.344 | 0.018568632 |
| RNU6-946P | CCDC51 | -0.343 | 0.018901854 |
| RNU6-946P | PREB | -0.343 | 0.018901854 |
| RNU6-946P | UBL4A | -0.343 | 0.018901854 |
| RNU6-946P | MBD1 | -0.343 | 0.018901854 |
| RNU6-946P | AHSA1 | -0.343 | 0.018901854 |
| RNU6-946P | FCHO1 | -0.342 | 0.019240294 |
| RNU6-946P | SARS | -0.342 | 0.019240294 |
| RNU6-946P | HDAC1 | -0.34 | 0.019933093 |
| RNU6-946P | PRMT5 | -0.34 | 0.019933093 |
| RNU6-946P | ENTPD6 | -0.339 | 0.020287588 |
| RNU6-946P | ACAD9 | -0.338 | 0.02064757 |
| RNU6-946P | PSMD2 | -0.338 | 0.02064757 |
| RNU6-946P | MED24 | -0.337 | 0.02101311 |
| RNU6-946P | VPS16 | -0.336 | 0.021384275 |
| RNU6-946P | GCN1 | -0.336 | 0.021384275 |
| RNU6-946P | NUDC | -0.335 | 0.021761137 |
| RNU6-946P | TUFM | -0.335 | 0.021761137 |
| RNU6-946P | AIFM1 | -0.335 | 0.021761137 |
| RNU6-946P | PSMD13 | -0.334 | 0.022143765 |
| RNU6-946P | ATP13A1 | -0.333 | 0.022532232 |
| RNU6-946P | MTFR1L | -0.333 | 0.022532232 |
| RNU6-946P | COPB2 | -0.333 | 0.022532232 |
| RNU6-946P | RNF40 | -0.333 | 0.022532232 |
| RNU6-946P | AP3D1 | -0.333 | 0.022532232 |
| RNU6-946P | RPA1 | -0.332 | 0.022926609 |
| RNU6-946P | TSC2 | -0.332 | 0.022926609 |
| RNU6-946P | RNASEH1 | -0.33 | 0.023733382 |
| RNU6-946P | RNF185 | -0.329 | 0.024145925 |
| RNU6-946P | ECHS1 | -0.329 | 0.024145925 |
| RNU6-946P | ALAD | -0.329 | 0.024145925 |
| RNU6-946P | PES1 | -0.329 | 0.024145925 |
| RNU6-946P | POLR1E | -0.328 | 0.024564671 |
| RNU6-946P | SF3B3 | -0.328 | 0.024564671 |
| RNU6-946P | FIBP | -0.328 | 0.024564671 |
| RNU6-946P | GLE1 | -0.328 | 0.024564671 |
| RNU6-946P | MORC2 | -0.328 | 0.024564671 |
| RNU6-946P | ATP5A1 | -0.328 | 0.024564671 |
| RNU6-946P | PEX19 | -0.328 | 0.024564671 |
| RNU6-946P | THYN1 | -0.327 | 0.024989694 |
| RNU6-946P | PPM1G | -0.327 | 0.024989694 |
| RNU6-946P | PLRG1 | -0.327 | 0.024989694 |
| RNU6-946P | NOL6 | -0.326 | 0.025421071 |
| RNU6-946P | THOP1 | -0.326 | 0.025421071 |
| RNU6-946P | ECH1 | -0.326 | 0.025421071 |
| RNU6-946P | FAM210B | -0.326 | 0.025421071 |
| RNU6-946P | APEX2 | -0.324 | 0.026303185 |
| RNU6-946P | TMEM214 | -0.324 | 0.026303185 |
| RNU6-946P | ATP1A1 | -0.324 | 0.026303185 |
| RNU6-946P | GOT2 | -0.324 | 0.026303185 |
| RNU6-946P | PRKAB1 | -0.323 | 0.026754076 |
| RNU6-946P | ELAC2 | -0.322 | 0.027211626 |
| RNU6-946P | TBRG4 | -0.322 | 0.027211626 |
| RNU6-946P | SF3A3 | -0.322 | 0.027211626 |
| RNU6-946P | ATP5B | -0.322 | 0.027211626 |
| RNU6-946P | PYCR2 | -0.32 | 0.028147017 |
| RNU6-946P | AKR1A1 | -0.32 | 0.028147017 |
| RNU6-946P | NMT1 | -0.32 | 0.028147017 |
| RNU6-946P | MRPL37 | -0.32 | 0.028147017 |
| RNU6-946P | RNF25 | -0.32 | 0.028147017 |
| RNU6-946P | PSMC4 | -0.32 | 0.028147017 |
| RNU6-946P | KARS | -0.32 | 0.028147017 |
| RNU6-946P | L3MBTL2 | -0.32 | 0.028147017 |
| RNU6-946P | HPS1 | -0.319 | 0.028625015 |
| RNU6-946P | DDX56 | -0.319 | 0.028625015 |
| RNU6-946P | PSMD4 | -0.319 | 0.028625015 |
| RNU6-946P | DHRS4 | -0.319 | 0.028625015 |
| RNU6-946P | PPIL1 | -0.319 | 0.028625015 |
| RNU6-946P | NELFCD | -0.318 | 0.029109988 |
| RNU6-946P | ERCC3 | -0.317 | 0.029602015 |
| RNU6-946P | SELENON | -0.317 | 0.029602015 |
| RNU6-946P | NUP93 | -0.317 | 0.029602015 |
| RNU6-946P | PSMC3 | -0.316 | 0.030101178 |
| RNU6-946P | CCDC22 | -0.316 | 0.030101178 |
| RNU6-946P | MAEA | -0.316 | 0.030101178 |
| RNU6-946P | TOE1 | -0.316 | 0.030101178 |
| RNU6-946P | NOP56 | -0.315 | 0.030607558 |
| RNU6-946P | NSDHL | -0.315 | 0.030607558 |
| RNU6-946P | ABCC5 | -0.314 | 0.031121236 |
| RNU6-946P | BATF | -0.314 | 0.031121236 |
| RNU6-946P | UQCRC1 | -0.314 | 0.031121236 |
| RNU6-946P | COG2 | -0.313 | 0.031642296 |
| RNU6-946P | CEP131 | -0.313 | 0.031642296 |
| RNU6-946P | CPT2 | -0.313 | 0.031642296 |
| RNU6-946P | MSTO1 | -0.313 | 0.031642296 |
| RNU6-946P | GGA3 | -0.312 | 0.032170819 |
| RNU6-946P | TRMU | -0.311 | 0.032706889 |
| RNU6-946P | SLC29A1 | -0.31 | 0.033250591 |
| RNU6-946P | POLG | -0.31 | 0.033250591 |
| RNU6-946P | ZNF343 | -0.31 | 0.033250591 |
| RNU6-946P | ZNF691 | -0.31 | 0.033250591 |
| RNU6-946P | MKS1 | -0.31 | 0.033250591 |
| RNU6-946P | CAD | -0.31 | 0.033250591 |
| RNU6-946P | ERCC2 | -0.309 | 0.033802009 |
| RNU6-946P | EIF2B2 | -0.308 | 0.034361227 |
| RNU6-946P | PRPF6 | -0.307 | 0.034928332 |
| RNU6-946P | IVD | -0.307 | 0.034928332 |
| RNU6-946P | HADHA | -0.307 | 0.034928332 |
| RNU6-946P | ENO1 | -0.307 | 0.034928332 |
| RNU6-946P | EMC3 | -0.307 | 0.034928332 |
| RNU6-946P | LETM1 | -0.306 | 0.035503409 |
| RNU6-946P | ZNF672 | -0.306 | 0.035503409 |
| RNU6-946P | KAT14 | -0.306 | 0.035503409 |
| RNU6-946P | POMGNT1 | -0.306 | 0.035503409 |
| RNU6-946P | DPAGT1 | -0.306 | 0.035503409 |
| RNU6-946P | MRPS11 | -0.306 | 0.035503409 |
| RNU6-946P | MUTYH | -0.306 | 0.035503409 |
| RNU6-946P | CCDC137 | -0.305 | 0.036086544 |
| RNU6-946P | LRRC47 | -0.304 | 0.036677826 |
| RNU6-946P | NCF4 | -0.304 | 0.036677826 |
| RNU6-946P | RNASEH2A | -0.304 | 0.036677826 |
| RNU6-946P | BECN1 | -0.303 | 0.037277341 |
| RNU6-946P | DNAJC8 | -0.303 | 0.037277341 |
| RNU6-946P | ALKBH3 | -0.303 | 0.037277341 |
| RNU6-946P | PARP2 | -0.303 | 0.037277341 |
| RNU6-946P | CCT5 | -0.303 | 0.037277341 |
| RNU6-946P | SART3 | -0.303 | 0.037277341 |
| RNU6-946P | TOMM34 | -0.302 | 0.037885177 |
| RNU6-946P | RPL4 | -0.302 | 0.037885177 |
| RNU6-946P | PRPF19 | -0.301 | 0.038501424 |
| RNU6-946P | AP2M1 | -0.301 | 0.038501424 |
| RNU6-946P | ODF2 | -0.301 | 0.038501424 |
| RNU6-946P | RUVBL2 | -0.301 | 0.038501424 |
| RNU6-946P | UROD | -0.301 | 0.038501424 |
| RNU6-946P | HDAC6 | -0.3 | 0.039126169 |
| RNU6-946P | RANGAP1 | -0.3 | 0.039126169 |
| RNU6-946P | GBF1 | -0.3 | 0.039126169 |
| RNU6-946P | VCP | -0.3 | 0.039126169 |
| RNU6-946P | OXSM | -0.3 | 0.039126169 |
| RNU6-946P | FH | -0.3 | 0.039126169 |
| RNU6-946P | FTSJ1 | -0.3 | 0.039126169 |
| RNU6-946P | DLST | -0.299 | 0.039759502 |
| RNU6-946P | SRRD | -0.299 | 0.039759502 |
| RNU6-946P | MPI | -0.299 | 0.039759502 |
| RNU6-946P | PSMC5 | -0.298 | 0.040401514 |
| RNU6-946P | VPS39 | -0.298 | 0.040401514 |
| RNU6-946P | ZNF646 | -0.298 | 0.040401514 |
| RNU6-946P | LRRC45 | -0.298 | 0.040401514 |
| RNU6-946P | ACO2 | -0.298 | 0.040401514 |
| RNU6-946P | RBM10 | -0.298 | 0.040401514 |
| RNU6-946P | KLHL12 | -0.298 | 0.040401514 |
| RNU6-946P | NELFE | -0.297 | 0.041052295 |
| RNU6-946P | RTFDC1 | -0.297 | 0.041052295 |
| RNU6-946P | GTF3C1 | -0.297 | 0.041052295 |
| RNU6-946P | DHX38 | -0.296 | 0.041711935 |
| RNU6-946P | SPOUT1 | -0.296 | 0.041711935 |
| RNU6-946P | TELO2 | -0.295 | 0.042380526 |
| RNU6-946P | PEF1 | -0.295 | 0.042380526 |
| RNU6-946P | HECTD3 | -0.295 | 0.042380526 |
| RNU6-946P | PARP1 | -0.295 | 0.042380526 |
| RNU6-946P | SMARCAL1 | -0.294 | 0.04305816 |
| RNU6-946P | MRPS9 | -0.294 | 0.04305816 |
| RNU6-946P | DMAP1 | -0.294 | 0.04305816 |
| RNU6-946P | P3H1 | -0.293 | 0.043744928 |
| RNU6-946P | XPO6 | -0.293 | 0.043744928 |
| RNU6-946P | FDPS | -0.292 | 0.044440925 |
| RNU6-946P | MRPS7 | -0.292 | 0.044440925 |
| RNU6-946P | CNDP2 | -0.292 | 0.044440925 |
| RNU6-946P | TRAP1 | -0.292 | 0.044440925 |
| RNU6-946P | EBNA1BP2 | -0.291 | 0.045146241 |
| RNU6-946P | POLD1 | -0.291 | 0.045146241 |
| RNU6-946P | PRPF31 | -0.291 | 0.045146241 |
| RNU6-946P | PITRM1 | -0.291 | 0.045146241 |
| RNU6-946P | TACC3 | -0.29 | 0.045860972 |
| RNU6-946P | PDIA6 | -0.29 | 0.045860972 |
| RNU6-946P | TTC31 | -0.29 | 0.045860972 |
| RNU6-946P | MECR | -0.29 | 0.045860972 |
| RNU6-946P | ABCF2 | -0.29 | 0.045860972 |
| RNU6-946P | CLUH | -0.29 | 0.045860972 |
| RNU6-946P | KIF22 | -0.29 | 0.045860972 |
| RNU6-946P | WDR61 | -0.29 | 0.045860972 |
| RNU6-946P | SEPHS2 | -0.29 | 0.045860972 |
| RNU6-946P | RNF123 | -0.289 | 0.046585211 |
| RNU6-946P | EIF2D | -0.289 | 0.046585211 |
| RNU6-946P | USP19 | -0.289 | 0.046585211 |
| RNU6-946P | METTL17 | -0.289 | 0.046585211 |
| RNU6-946P | CHID1 | -0.289 | 0.046585211 |
| RNU6-946P | INF2 | -0.288 | 0.047319053 |
| RNU6-946P | ATIC | -0.288 | 0.047319053 |
| RNU6-946P | EEF2KMT | -0.288 | 0.047319053 |
| RNU6-946P | PPOX | -0.288 | 0.047319053 |
| RNU6-946P | WRAP73 | -0.288 | 0.047319053 |
| RNU6-946P | NUP188 | -0.287 | 0.048062591 |
| RNU6-946P | ACOX3 | -0.287 | 0.048062591 |
| RNU6-946P | PELO | -0.287 | 0.048062591 |
| RNU6-946P | SAE1 | -0.287 | 0.048062591 |
| RNU6-946P | NSMCE1 | -0.286 | 0.048815922 |
| RNU6-946P | CLK2 | -0.286 | 0.048815922 |
| RNU6-946P | TSSC1 | -0.286 | 0.048815922 |
| RNU6-946P | EDC4 | -0.286 | 0.048815922 |
| RNU6-946P | PPP6R1 | -0.286 | 0.048815922 |
| RNU6-946P | SBF1 | -0.286 | 0.048815922 |
| RNU6-946P | ANAPC5 | -0.286 | 0.048815922 |
| RNU6-946P | SLC5A6 | -0.286 | 0.048815922 |
| RNU6-946P | MCM3 | -0.285 | 0.04957914 |
| RNU6-946P | EIF3D | -0.285 | 0.04957914 |
| RNU6-946P | TXNDC17 | 0.308 | 0.049843692 |
| RNU6-946P | HSPE1 | 0.308 | 0.049843692 |
| RNU6-946P | TTC19 | 0.308 | 0.049843692 |
| RNU6-946P | MCM9 | 0.308 | 0.049843692 |
| RNU6-946P | DTD2 | 0.308 | 0.049843692 |
| RNU6-946P | CORIN | 0.308 | 0.049843692 |
| RNU6-946P | CELA2A | 0.308 | 0.049843692 |
| RNU6-946P | PTP4A1 | 0.309 | 0.049077053 |
| RNU6-946P | CTU1 | 0.309 | 0.049077053 |
| RNU6-946P | NEMP2 | 0.309 | 0.049077053 |
| RNU6-946P | TMPRSS2 | 0.309 | 0.049077053 |
| RNU6-946P | GOLPH3 | 0.309 | 0.049077053 |
| RNU6-946P | SCAF8 | 0.309 | 0.049077053 |
| RNU6-946P | QSER1 | 0.309 | 0.049077053 |
| RNU6-946P | LEPROTL1 | 0.309 | 0.049077053 |
| RNU6-946P | DAD1 | 0.31 | 0.048320336 |
| RNU6-946P | PPP3R1 | 0.31 | 0.048320336 |
| RNU6-946P | CSPP1 | 0.31 | 0.048320336 |
| RNU6-946P | TATDN1 | 0.31 | 0.048320336 |
| RNU6-946P | UBE2E2 | 0.31 | 0.048320336 |
| RNU6-946P | USP44 | 0.311 | 0.047573443 |
| RNU6-946P | CCDC65 | 0.311 | 0.047573443 |
| RNU6-946P | ZNF66 | 0.311 | 0.047573443 |
| RNU6-946P | ZBED3 | 0.311 | 0.047573443 |
| RNU6-946P | FOXJ3 | 0.311 | 0.047573443 |
| RNU6-946P | C3orf35 | 0.311 | 0.047573443 |
| RNU6-946P | C1QTNF7 | 0.311 | 0.047573443 |
| RNU6-946P | TCERG1 | 0.311 | 0.047573443 |
| RNU6-946P | RAP2B | 0.311 | 0.047573443 |
| RNU6-946P | WDR44 | 0.311 | 0.047573443 |
| RNU6-946P | MED6 | 0.312 | 0.046836281 |
| RNU6-946P | KIAA0408 | 0.312 | 0.046836281 |
| RNU6-946P | ZNF383 | 0.312 | 0.046836281 |
| RNU6-946P | TMEM242 | 0.312 | 0.046836281 |
| RNU6-946P | KHSRP | 0.312 | 0.046836281 |
| RNU6-946P | LYPD8 | 0.312 | 0.046836281 |
| RNU6-946P | SLC22A12 | 0.312 | 0.046836281 |
| RNU6-946P | RSBN1L | 0.312 | 0.046836281 |
| RNU6-946P | SMIM4 | 0.312 | 0.046836281 |
| RNU6-946P | INSL6 | 0.312 | 0.046836281 |
| RNU6-946P | G2E3 | 0.312 | 0.046836281 |
| RNU6-946P | ING3 | 0.312 | 0.046836281 |
| RNU6-946P | KRT10 | 0.313 | 0.046108753 |
| RNU6-946P | RFWD2 | 0.313 | 0.046108753 |
| RNU6-946P | DYNLL2 | 0.313 | 0.046108753 |
| RNU6-946P | B9D2 | 0.313 | 0.046108753 |
| RNU6-946P | SHPRH | 0.313 | 0.046108753 |
| RNU6-946P | BIRC6 | 0.313 | 0.046108753 |
| RNU6-946P | OARD1 | 0.313 | 0.046108753 |
| RNU6-946P | CLEC18A | 0.313 | 0.046108753 |
| RNU6-946P | ZNF292 | 0.313 | 0.046108753 |
| RNU6-946P | RDH14 | 0.314 | 0.045390765 |
| RNU6-946P | ELOC | 0.314 | 0.045390765 |
| RNU6-946P | SUMO4 | 0.314 | 0.045390765 |
| RNU6-946P | KANSL1L | 0.314 | 0.045390765 |
| RNU6-946P | TMEM253 | 0.314 | 0.045390765 |
| RNU6-946P | SSBP3 | 0.314 | 0.045390765 |
| RNU6-946P | RTN4 | 0.314 | 0.045390765 |
| RNU6-946P | RPL30 | 0.314 | 0.045390765 |
| RNU6-946P | OR7D2 | 0.314 | 0.045390765 |
| RNU6-946P | SEM1 | 0.314 | 0.045390765 |
| RNU6-946P | MSL2 | 0.315 | 0.044682224 |
| RNU6-946P | NF1 | 0.315 | 0.044682224 |
| RNU6-946P | AL136531.2 | 0.315 | 0.044682224 |
| RNU6-946P | CREB1 | 0.315 | 0.044682224 |
| RNU6-946P | MAP4K5 | 0.315 | 0.044682224 |
| RNU6-946P | MRPL54 | 0.315 | 0.044682224 |
| RNU6-946P | SNRNP27 | 0.315 | 0.044682224 |
| RNU6-946P | CEP295NL | 0.315 | 0.044682224 |
| RNU6-946P | CNOT2 | 0.315 | 0.044682224 |
| RNU6-946P | JMJD1C | 0.315 | 0.044682224 |
| RNU6-946P | MAGOHB | 0.315 | 0.044682224 |
| RNU6-946P | GRIK1 | 0.315 | 0.044682224 |
| RNU6-946P | PCDHGA9 | 0.316 | 0.043983036 |
| RNU6-946P | FOXQ1 | 0.316 | 0.043983036 |
| RNU6-946P | BNIP3 | 0.316 | 0.043983036 |
| RNU6-946P | UPRT | 0.316 | 0.043983036 |
| RNU6-946P | SLC30A7 | 0.316 | 0.043983036 |
| RNU6-946P | AUTS2 | 0.316 | 0.043983036 |
| RNU6-946P | SLC35G2 | 0.316 | 0.043983036 |
| RNU6-946P | KCNJ14 | 0.316 | 0.043983036 |
| RNU6-946P | LEMD3 | 0.316 | 0.043983036 |
| RNU6-946P | KATNBL1 | 0.317 | 0.043293107 |
| RNU6-946P | TAS2R39 | 0.317 | 0.043293107 |
| RNU6-946P | SDHAF3 | 0.317 | 0.043293107 |
| RNU6-946P | FUT11 | 0.317 | 0.043293107 |
| RNU6-946P | NAA30 | 0.317 | 0.043293107 |
| RNU6-946P | POLR2J2 | 0.317 | 0.043293107 |
| RNU6-946P | CDK17 | 0.317 | 0.043293107 |
| RNU6-946P | OR13C9 | 0.317 | 0.043293107 |
| RNU6-946P | FAM83C | 0.317 | 0.043293107 |
| RNU6-946P | NR2E1 | 0.318 | 0.042612345 |
| RNU6-946P | PKD2L2 | 0.318 | 0.042612345 |
| RNU6-946P | FAM204A | 0.318 | 0.042612345 |
| RNU6-946P | AC010325.1 | 0.318 | 0.042612345 |
| RNU6-946P | ZNF780A | 0.318 | 0.042612345 |
| RNU6-946P | MKLN1 | 0.318 | 0.042612345 |
| RNU6-946P | ATXN2 | 0.318 | 0.042612345 |
| RNU6-946P | CNFN | 0.318 | 0.042612345 |
| RNU6-946P | RGSL1 | 0.319 | 0.041940657 |
| RNU6-946P | ABHD13 | 0.319 | 0.041940657 |
| RNU6-946P | NBEAL1 | 0.319 | 0.041940657 |
| RNU6-946P | PRB1 | 0.319 | 0.041940657 |
| RNU6-946P | AKIRIN2 | 0.319 | 0.041940657 |
| RNU6-946P | PTS | 0.319 | 0.041940657 |
| RNU6-946P | COX7A1 | 0.319 | 0.041940657 |
| RNU6-946P | SFT2D1 | 0.32 | 0.041277952 |
| RNU6-946P | UNC50 | 0.32 | 0.041277952 |
| RNU6-946P | FOXC2 | 0.32 | 0.041277952 |
| RNU6-946P | UPK3B | 0.32 | 0.041277952 |
| RNU6-946P | UBE2R2 | 0.321 | 0.040624137 |
| RNU6-946P | NAA15 | 0.321 | 0.040624137 |
| RNU6-946P | ORAI3 | 0.321 | 0.040624137 |
| RNU6-946P | ZFYVE16 | 0.321 | 0.040624137 |
| RNU6-946P | NAF1 | 0.322 | 0.039979123 |
| RNU6-946P | SUSD5 | 0.322 | 0.039979123 |
| RNU6-946P | PIBF1 | 0.322 | 0.039979123 |
| RNU6-946P | PIK3CA | 0.322 | 0.039979123 |
| RNU6-946P | C18orf25 | 0.322 | 0.039979123 |
| RNU6-946P | CASC4 | 0.322 | 0.039979123 |
| RNU6-946P | COX7A2L | 0.322 | 0.039979123 |
| RNU6-946P | CTDSPL2 | 0.322 | 0.039979123 |
| RNU6-946P | PBLD | 0.322 | 0.039979123 |
| RNU6-946P | KLHL32 | 0.322 | 0.039979123 |
| RNU6-946P | FXR2 | 0.322 | 0.039979123 |
| RNU6-946P | HUS1B | 0.322 | 0.039979123 |
| RNU6-946P | PIGP | 0.322 | 0.039979123 |
| RNU6-946P | RNASEH2B | 0.323 | 0.039342817 |
| RNU6-946P | MORF4L1 | 0.323 | 0.039342817 |
| RNU6-946P | BRMS1L | 0.323 | 0.039342817 |
| RNU6-946P | MEPCE | 0.323 | 0.039342817 |
| RNU6-946P | KCNS2 | 0.324 | 0.038715131 |
| RNU6-946P | ATP5J | 0.324 | 0.038715131 |
| RNU6-946P | SPINK4 | 0.324 | 0.038715131 |
| RNU6-946P | CACUL1 | 0.324 | 0.038715131 |
| RNU6-946P | KHDRBS3 | 0.324 | 0.038715131 |
| RNU6-946P | DNAH7 | 0.324 | 0.038715131 |
| RNU6-946P | ZNF428 | 0.324 | 0.038715131 |
| RNU6-946P | RNU6-1143P | 0.324 | 0.038715131 |
| RNU6-946P | TMEM50A | 0.325 | 0.038095974 |
| RNU6-946P | SLC45A2 | 0.325 | 0.038095974 |
| RNU6-946P | AL138752.2 | 0.325 | 0.038095974 |
| RNU6-946P | TRMT112 | 0.325 | 0.038095974 |
| RNU6-946P | TMEM251 | 0.325 | 0.038095974 |
| RNU6-946P | FOXF1 | 0.325 | 0.038095974 |
| RNU6-946P | CEBPZOS | 0.326 | 0.037485258 |
| RNU6-946P | LIX1L | 0.326 | 0.037485258 |
| RNU6-946P | RPS25 | 0.326 | 0.037485258 |
| RNU6-946P | KANSL1 | 0.326 | 0.037485258 |
| RNU6-946P | GNPDA2 | 0.327 | 0.036882894 |
| RNU6-946P | PTPN4 | 0.327 | 0.036882894 |
| RNU6-946P | MTMR7 | 0.327 | 0.036882894 |
| RNU6-946P | NUDT13 | 0.327 | 0.036882894 |
| RNU6-946P | EFCAB5 | 0.327 | 0.036882894 |
| RNU6-946P | TM4SF20 | 0.328 | 0.036288793 |
| RNU6-946P | ERICH6 | 0.328 | 0.036288793 |
| RNU6-946P | ING1 | 0.328 | 0.036288793 |
| RNU6-946P | SPPL3 | 0.328 | 0.036288793 |
| RNU6-946P | EPOP | 0.328 | 0.036288793 |
| RNU6-946P | BTBD18 | 0.328 | 0.036288793 |
| RNU6-946P | CRHR1 | 0.328 | 0.036288793 |
| RNU6-946P | WDR20 | 0.329 | 0.035702868 |
| RNU6-946P | ZNF451 | 0.329 | 0.035702868 |
| RNU6-946P | SUMO1 | 0.329 | 0.035702868 |
| RNU6-946P | EBPL | 0.329 | 0.035702868 |
| RNU6-946P | CCDC91 | 0.329 | 0.035702868 |
| RNU6-946P | ZNF652 | 0.33 | 0.035125032 |
| RNU6-946P | INO80D | 0.33 | 0.035125032 |
| RNU6-946P | CPSF4L | 0.33 | 0.035125032 |
| RNU6-946P | RBBP6 | 0.33 | 0.035125032 |
| RNU6-946P | ADORA2A | 0.33 | 0.035125032 |
| RNU6-946P | C5orf15 | 0.33 | 0.035125032 |
| RNU6-946P | FRS2 | 0.331 | 0.034555198 |
| RNU6-946P | ELK4 | 0.331 | 0.034555198 |
| RNU6-946P | YIPF4 | 0.331 | 0.034555198 |
| RNU6-946P | TSPYL1 | 0.331 | 0.034555198 |
| RNU6-946P | ETV3 | 0.331 | 0.034555198 |
| RNU6-946P | ALX4 | 0.331 | 0.034555198 |
| RNU6-946P | DUSP28 | 0.331 | 0.034555198 |
| RNU6-946P | AC068896.1 | 0.332 | 0.033993279 |
| RNU6-946P | ZBTB8B | 0.332 | 0.033993279 |
| RNU6-946P | WNT4 | 0.332 | 0.033993279 |
| RNU6-946P | VPS13C | 0.332 | 0.033993279 |
| RNU6-946P | TMEM60 | 0.332 | 0.033993279 |
| RNU6-946P | AL139353.1 | 0.333 | 0.033439191 |
| RNU6-946P | CSNK1G3 | 0.333 | 0.033439191 |
| RNU6-946P | GNRHR | 0.333 | 0.033439191 |
| RNU6-946P | PKN2 | 0.333 | 0.033439191 |
| RNU6-946P | ARL5B | 0.333 | 0.033439191 |
| RNU6-946P | IER3IP1 | 0.333 | 0.033439191 |
| RNU6-946P | DNAAF2 | 0.333 | 0.033439191 |
| RNU6-946P | ARF3 | 0.333 | 0.033439191 |
| RNU6-946P | TMSB15B | 0.334 | 0.032892847 |
| RNU6-946P | RAD51B | 0.334 | 0.032892847 |
| RNU6-946P | KMT2E | 0.334 | 0.032892847 |
| RNU6-946P | SLC39A5 | 0.334 | 0.032892847 |
| RNU6-946P | KPNA4 | 0.334 | 0.032892847 |
| RNU6-946P | ATG12 | 0.334 | 0.032892847 |
| RNU6-946P | CREBRF | 0.335 | 0.032354164 |
| RNU6-946P | DUSP10 | 0.335 | 0.032354164 |
| RNU6-946P | REV3L | 0.335 | 0.032354164 |
| RNU6-946P | CHM | 0.335 | 0.032354164 |
| RNU6-946P | ODF3 | 0.335 | 0.032354164 |
| RNU6-946P | LMO3 | 0.335 | 0.032354164 |
| RNU6-946P | RPS6KB1 | 0.335 | 0.032354164 |
| RNU6-946P | ZBTB41 | 0.335 | 0.032354164 |
| RNU6-946P | ENSA | 0.335 | 0.032354164 |
| RNU6-946P | RPL27 | 0.336 | 0.031823057 |
| RNU6-946P | MOB4 | 0.336 | 0.031823057 |
| RNU6-946P | DHX36 | 0.336 | 0.031823057 |
| RNU6-946P | DOLPP1 | 0.336 | 0.031823057 |
| RNU6-946P | AC036214.3 | 0.336 | 0.031823057 |
| RNU6-946P | OR2AK2 | 0.336 | 0.031823057 |
| RNU6-946P | CDK2 | 0.336 | 0.031823057 |
| RNU6-946P | UQCRHL | 0.336 | 0.031823057 |
| RNU6-946P | ACPT | 0.337 | 0.031299442 |
| RNU6-946P | SNRPF | 0.337 | 0.031299442 |
| RNU6-946P | PAFAH1B2 | 0.337 | 0.031299442 |
| RNU6-946P | SUGT1 | 0.337 | 0.031299442 |
| RNU6-946P | MSANTD1 | 0.337 | 0.031299442 |
| RNU6-946P | HIVEP1 | 0.337 | 0.031299442 |
| RNU6-946P | ATG10 | 0.337 | 0.031299442 |
| RNU6-946P | NSMCE3 | 0.337 | 0.031299442 |
| RNU6-946P | ZNRF2 | 0.337 | 0.031299442 |
| RNU6-946P | COX6C | 0.337 | 0.031299442 |
| RNU6-946P | DPY30 | 0.338 | 0.030783237 |
| RNU6-946P | TMCC1 | 0.338 | 0.030783237 |
| RNU6-946P | C9orf3 | 0.338 | 0.030783237 |
| RNU6-946P | BCLAF1 | 0.338 | 0.030783237 |
| RNU6-946P | MPP5 | 0.339 | 0.030274359 |
| RNU6-946P | SENP3 | 0.339 | 0.030274359 |
| RNU6-946P | PHF14 | 0.34 | 0.029772725 |
| RNU6-946P | PLG | 0.34 | 0.029772725 |
| RNU6-946P | EPHX4 | 0.34 | 0.029772725 |
| RNU6-946P | SDHAF4 | 0.34 | 0.029772725 |
| RNU6-946P | GABPB1 | 0.34 | 0.029772725 |
| RNU6-946P | TFAP4 | 0.34 | 0.029772725 |
| RNU6-946P | PAN3 | 0.34 | 0.029772725 |
| RNU6-946P | TBR1 | 0.34 | 0.029772725 |
| RNU6-946P | CAMTA1 | 0.341 | 0.029278255 |
| RNU6-946P | CERS2 | 0.341 | 0.029278255 |
| RNU6-946P | DDX52 | 0.341 | 0.029278255 |
| RNU6-946P | HIGD1B | 0.341 | 0.029278255 |
| RNU6-946P | NEK7 | 0.341 | 0.029278255 |
| RNU6-946P | C1GALT1 | 0.341 | 0.029278255 |
| RNU6-946P | SKP1 | 0.341 | 0.029278255 |
| RNU6-946P | MRPS28 | 0.342 | 0.028790867 |
| RNU6-946P | RAB21 | 0.342 | 0.028790867 |
| RNU6-946P | CHORDC1 | 0.342 | 0.028790867 |
| RNU6-946P | KRT15 | 0.342 | 0.028790867 |
| RNU6-946P | ZNF91 | 0.342 | 0.028790867 |
| RNU6-946P | ISCA2 | 0.342 | 0.028790867 |
| RNU6-946P | PIAS2 | 0.342 | 0.028790867 |
| RNU6-946P | POMZP3 | 0.342 | 0.028790867 |
| RNU6-946P | ANKHD1 | 0.342 | 0.028790867 |
| RNU6-946P | RASL11B | 0.342 | 0.028790867 |
| RNU6-946P | RAB33B | 0.342 | 0.028790867 |
| RNU6-946P | NXPH3 | 0.342 | 0.028790867 |
| RNU6-946P | COX7A2 | 0.343 | 0.028310482 |
| RNU6-946P | AC134669.2 | 0.343 | 0.028310482 |
| RNU6-946P | NREP | 0.344 | 0.027837018 |
| RNU6-946P | DCUN1D1 | 0.344 | 0.027837018 |
| RNU6-946P | COA1 | 0.344 | 0.027837018 |
| RNU6-946P | C1D | 0.344 | 0.027837018 |
| RNU6-946P | MED13 | 0.345 | 0.027370398 |
| RNU6-946P | RNF11 | 0.345 | 0.027370398 |
| RNU6-946P | ZNF205 | 0.345 | 0.027370398 |
| RNU6-946P | GLP2R | 0.345 | 0.027370398 |
| RNU6-946P | RICTOR | 0.346 | 0.026910541 |
| RNU6-946P | DSC3 | 0.346 | 0.026910541 |
| RNU6-946P | C20orf173 | 0.346 | 0.026910541 |
| RNU6-946P | SERTAD2 | 0.346 | 0.026910541 |
| RNU6-946P | SMAD2 | 0.346 | 0.026910541 |
| RNU6-946P | CRLF1 | 0.346 | 0.026910541 |
| RNU6-946P | MON2 | 0.346 | 0.026910541 |
| RNU6-946P | ROPN1B | 0.347 | 0.026457371 |
| RNU6-946P | TMEM106A | 0.347 | 0.026457371 |
| RNU6-946P | RAB11FIP2 | 0.348 | 0.026010808 |
| RNU6-946P | HTD2 | 0.348 | 0.026010808 |
| RNU6-946P | SFT2D2 | 0.348 | 0.026010808 |
| RNU6-946P | PABPN1 | 0.348 | 0.026010808 |
| RNU6-946P | TLK1 | 0.348 | 0.026010808 |
| RNU6-946P | SLC25A33 | 0.348 | 0.026010808 |
| RNU6-946P | APH1A | 0.349 | 0.025570777 |
| RNU6-946P | CHGA | 0.349 | 0.025570777 |
| RNU6-946P | GNG5 | 0.349 | 0.025570777 |
| RNU6-946P | RUNX1 | 0.349 | 0.025570777 |
| RNU6-946P | GMDS | 0.349 | 0.025570777 |
| RNU6-946P | SCLT1 | 0.349 | 0.025570777 |
| RNU6-946P | DNAJB7 | 0.35 | 0.025137201 |
| RNU6-946P | PP2D1 | 0.351 | 0.024710003 |
| RNU6-946P | RPS29 | 0.351 | 0.024710003 |
| RNU6-946P | PIK3C2A | 0.351 | 0.024710003 |
| RNU6-946P | MBD5 | 0.351 | 0.024710003 |
| RNU6-946P | WDSUB1 | 0.351 | 0.024710003 |
| RNU6-946P | ZNHIT3 | 0.351 | 0.024710003 |
| RNU6-946P | RPL37 | 0.351 | 0.024710003 |
| RNU6-946P | COX7B | 0.352 | 0.024289108 |
| RNU6-946P | ASIP | 0.353 | 0.023874442 |
| RNU6-946P | CRX | 0.353 | 0.023874442 |
| RNU6-946P | F2RL2 | 0.353 | 0.023874442 |
| RNU6-946P | MOB1B | 0.353 | 0.023874442 |
| RNU6-946P | NAB2 | 0.354 | 0.02346593 |
| RNU6-946P | ZNF713 | 0.354 | 0.02346593 |
| RNU6-946P | ZNF430 | 0.354 | 0.02346593 |
| RNU6-946P | ZFAND6 | 0.354 | 0.02346593 |
| RNU6-946P | RPS27L | 0.354 | 0.02346593 |
| RNU6-946P | RBP2 | 0.354 | 0.02346593 |
| RNU6-946P | ERCC6L2 | 0.354 | 0.02346593 |
| RNU6-946P | RNF32 | 0.354 | 0.02346593 |
| RNU6-946P | C1QTNF3-AMACR | 0.355 | 0.023063499 |
| RNU6-946P | CDK12 | 0.355 | 0.023063499 |
| RNU6-946P | C7orf26 | 0.355 | 0.023063499 |
| RNU6-946P | MBLAC1 | 0.355 | 0.023063499 |
| RNU6-946P | GJD4 | 0.355 | 0.023063499 |
| RNU6-946P | CLK1 | 0.356 | 0.022667075 |
| RNU6-946P | PCDHGB4 | 0.356 | 0.022667075 |
| RNU6-946P | NDUFB3 | 0.356 | 0.022667075 |
| RNU6-946P | CMC1 | 0.356 | 0.022667075 |
| RNU6-946P | SLC10A7 | 0.356 | 0.022667075 |
| RNU6-946P | CCNI | 0.357 | 0.022276585 |
| RNU6-946P | HINT1 | 0.357 | 0.022276585 |
| RNU6-946P | CCDC36 | 0.357 | 0.022276585 |
| RNU6-946P | CDC73 | 0.358 | 0.021891959 |
| RNU6-946P | ABHD18 | 0.358 | 0.021891959 |
| RNU6-946P | NR1D2 | 0.358 | 0.021891959 |
| RNU6-946P | MT-ND3 | 0.358 | 0.021891959 |
| RNU6-946P | PCGF6 | 0.358 | 0.021891959 |
| RNU6-946P | FAM217A | 0.358 | 0.021891959 |
| RNU6-946P | INAFM2 | 0.358 | 0.021891959 |
| RNU6-946P | KDM6A | 0.358 | 0.021891959 |
| RNU6-946P | NAT14 | 0.359 | 0.021513123 |
| RNU6-946P | TMEM64 | 0.359 | 0.021513123 |
| RNU6-946P | IQCA1L | 0.36 | 0.021140009 |
| RNU6-946P | PET100 | 0.36 | 0.021140009 |
| RNU6-946P | AEBP2 | 0.36 | 0.021140009 |
| RNU6-946P | RHEB | 0.36 | 0.021140009 |
| RNU6-946P | PEX13 | 0.361 | 0.020772544 |
| RNU6-946P | MLLT11 | 0.361 | 0.020772544 |
| RNU6-946P | TBC1D19 | 0.361 | 0.020772544 |
| RNU6-946P | GPR18 | 0.362 | 0.02041066 |
| RNU6-946P | CDC14A | 0.362 | 0.02041066 |
| RNU6-946P | PCDHGA8 | 0.362 | 0.02041066 |
| RNU6-946P | KLHL11 | 0.362 | 0.02041066 |
| RNU6-946P | LHX9 | 0.362 | 0.02041066 |
| RNU6-946P | MIER1 | 0.363 | 0.020054287 |
| RNU6-946P | ZNF254 | 0.363 | 0.020054287 |
| RNU6-946P | RNF44 | 0.363 | 0.020054287 |
| RNU6-946P | C14orf28 | 0.364 | 0.019703357 |
| RNU6-946P | AKAP5 | 0.364 | 0.019703357 |
| RNU6-946P | NME2 | 0.364 | 0.019703357 |
| RNU6-946P | RNF5 | 0.365 | 0.019357802 |
| RNU6-946P | NDUFB1 | 0.365 | 0.019357802 |
| RNU6-946P | UBE2E1 | 0.365 | 0.019357802 |
| RNU6-946P | PPP3CB | 0.365 | 0.019357802 |
| RNU6-946P | SNRPC | 0.365 | 0.019357802 |
| RNU6-946P | C6orf48 | 0.365 | 0.019357802 |
| RNU6-946P | RAB18 | 0.366 | 0.019017554 |
| RNU6-946P | ZBTB10 | 0.366 | 0.019017554 |
| RNU6-946P | AC009014.1 | 0.366 | 0.019017554 |
| RNU6-946P | BBS9 | 0.366 | 0.019017554 |
| RNU6-946P | SNX3 | 0.366 | 0.019017554 |
| RNU6-946P | TRIM33 | 0.367 | 0.018682547 |
| RNU6-946P | LNPEP | 0.367 | 0.018682547 |
| RNU6-946P | PDE6D | 0.367 | 0.018682547 |
| RNU6-946P | RAPGEF6 | 0.367 | 0.018682547 |
| RNU6-946P | TMEM258 | 0.367 | 0.018682547 |
| RNU6-946P | HERC4 | 0.367 | 0.018682547 |
| RNU6-946P | DNAL1 | 0.367 | 0.018682547 |
| RNU6-946P | NDUFS4 | 0.367 | 0.018682547 |
| RNU6-946P | MAX | 0.367 | 0.018682547 |
| RNU6-946P | MYLPF | 0.368 | 0.018352714 |
| RNU6-946P | ANKRD37 | 0.368 | 0.018352714 |
| RNU6-946P | ERF | 0.368 | 0.018352714 |
| RNU6-946P | EGR1 | 0.369 | 0.01802799 |
| RNU6-946P | FAM172A | 0.369 | 0.01802799 |
| RNU6-946P | SEH1L | 0.369 | 0.01802799 |
| RNU6-946P | ALG13 | 0.37 | 0.017708309 |
| RNU6-946P | NDUFA1 | 0.37 | 0.017708309 |
| RNU6-946P | LSM8 | 0.371 | 0.017393607 |
| RNU6-946P | ULK4 | 0.371 | 0.017393607 |
| RNU6-946P | C4orf46 | 0.371 | 0.017393607 |
| RNU6-946P | TGIF2-C20orf24 | 0.371 | 0.017393607 |
| RNU6-946P | JPH3 | 0.372 | 0.01708382 |
| RNU6-946P | PTEN | 0.372 | 0.01708382 |
| RNU6-946P | CHD1 | 0.372 | 0.01708382 |
| RNU6-946P | MCTS1 | 0.372 | 0.01708382 |
| RNU6-946P | RNF38 | 0.372 | 0.01708382 |
| RNU6-946P | OGN | 0.372 | 0.01708382 |
| RNU6-946P | SMURF2 | 0.373 | 0.016778885 |
| RNU6-946P | ACYP2 | 0.373 | 0.016778885 |
| RNU6-946P | VPREB1 | 0.373 | 0.016778885 |
| RNU6-946P | ZC2HC1C | 0.373 | 0.016778885 |
| RNU6-946P | MT-ATP8 | 0.374 | 0.016478738 |
| RNU6-946P | CKS2 | 0.374 | 0.016478738 |
| RNU6-946P | MED31 | 0.374 | 0.016478738 |
| RNU6-946P | MTRNR2L12 | 0.374 | 0.016478738 |
| RNU6-946P | VPS13B | 0.374 | 0.016478738 |
| RNU6-946P | SS18 | 0.375 | 0.016183318 |
| RNU6-946P | MRPS18C | 0.375 | 0.016183318 |
| RNU6-946P | ZBTB22 | 0.375 | 0.016183318 |
| RNU6-946P | MT-ND6 | 0.375 | 0.016183318 |
| RNU6-946P | CMC2 | 0.376 | 0.015892562 |
| RNU6-946P | YTHDF3 | 0.376 | 0.015892562 |
| RNU6-946P | MAP6D1 | 0.376 | 0.015892562 |
| RNU6-946P | GPR61 | 0.376 | 0.015892562 |
| RNU6-946P | CRIPT | 0.377 | 0.01560641 |
| RNU6-946P | CNTN2 | 0.377 | 0.01560641 |
| RNU6-946P | PEX26 | 0.377 | 0.01560641 |
| RNU6-946P | HSPB11 | 0.378 | 0.015324801 |
| RNU6-946P | MAPK8 | 0.378 | 0.015324801 |
| RNU6-946P | DNAJB8 | 0.378 | 0.015324801 |
| RNU6-946P | FBXL4 | 0.378 | 0.015324801 |
| RNU6-946P | COX7C | 0.378 | 0.015324801 |
| RNU6-946P | PAPD4 | 0.378 | 0.015324801 |
| RNU6-946P | CCDC7 | 0.379 | 0.015047676 |
| RNU6-946P | PDS5B | 0.379 | 0.015047676 |
| RNU6-946P | KLB | 0.379 | 0.015047676 |
| RNU6-946P | PSPC1 | 0.379 | 0.015047676 |
| RNU6-946P | ATP5L2 | 0.379 | 0.015047676 |
| RNU6-946P | CHTOP | 0.38 | 0.014774974 |
| RNU6-946P | NRL | 0.38 | 0.014774974 |
| RNU6-946P | SIAH1 | 0.38 | 0.014774974 |
| RNU6-946P | SYPL1 | 0.38 | 0.014774974 |
| RNU6-946P | PRH1 | 0.38 | 0.014774974 |
| RNU6-946P | SMN1 | 0.38 | 0.014774974 |
| RNU6-946P | RNF115 | 0.38 | 0.014774974 |
| RNU6-946P | CDKL4 | 0.382 | 0.014242606 |
| RNU6-946P | ZBTB11 | 0.383 | 0.013982824 |
| RNU6-946P | SPDYE5 | 0.383 | 0.013982824 |
| RNU6-946P | CDK13 | 0.383 | 0.013982824 |
| RNU6-946P | CDKAL1 | 0.384 | 0.013727234 |
| RNU6-946P | ZNF407 | 0.384 | 0.013727234 |
| RNU6-946P | SOX4 | 0.384 | 0.013727234 |
| RNU6-946P | MTRNR2L11 | 0.385 | 0.013475778 |
| RNU6-946P | DLEU1 | 0.385 | 0.013475778 |
| RNU6-946P | MTRNR2L13 | 0.385 | 0.013475778 |
| RNU6-946P | ANGPTL7 | 0.386 | 0.013228402 |
| RNU6-946P | RARB | 0.386 | 0.013228402 |
| RNU6-946P | CAMKMT | 0.386 | 0.013228402 |
| RNU6-946P | AFF4 | 0.386 | 0.013228402 |
| RNU6-946P | EPC1 | 0.386 | 0.013228402 |
| RNU6-946P | RBM34 | 0.387 | 0.012985049 |
| RNU6-946P | ZCCHC3 | 0.387 | 0.012985049 |
| RNU6-946P | RPL39 | 0.387 | 0.012985049 |
| RNU6-946P | PDE12 | 0.388 | 0.012745665 |
| RNU6-946P | KLHL24 | 0.388 | 0.012745665 |
| RNU6-946P | KMT2C | 0.388 | 0.012745665 |
| RNU6-946P | MTRNR2L5 | 0.388 | 0.012745665 |
| RNU6-946P | STRN3 | 0.388 | 0.012745665 |
| RNU6-946P | TLK2 | 0.389 | 0.012510194 |
| RNU6-946P | RNF138 | 0.389 | 0.012510194 |
| RNU6-946P | EIF4E | 0.39 | 0.012278584 |
| RNU6-946P | ASB14 | 0.39 | 0.012278584 |
| RNU6-946P | ATXN7 | 0.39 | 0.012278584 |
| RNU6-946P | DNAL4 | 0.391 | 0.012050781 |
| RNU6-946P | SMIM27 | 0.391 | 0.012050781 |
| RNU6-946P | KIAA0825 | 0.391 | 0.012050781 |
| RNU6-946P | SCUBE3 | 0.392 | 0.011826732 |
| RNU6-946P | LINC00238 | 0.392 | 0.011826732 |
| RNU6-946P | MTRNR2L6 | 0.392 | 0.011826732 |
| RNU6-946P | PRH2 | 0.392 | 0.011826732 |
| RNU6-946P | RAX2 | 0.392 | 0.011826732 |
| RNU6-946P | MTRNR2L10 | 0.393 | 0.011606385 |
| RNU6-946P | NAP1L5 | 0.393 | 0.011606385 |
| RNU6-946P | ARL6IP6 | 0.394 | 0.011389688 |
| RNU6-946P | TMEM170A | 0.394 | 0.011389688 |
| RNU6-946P | PHF5A | 0.394 | 0.011389688 |
| RNU6-946P | ZNF92 | 0.394 | 0.011389688 |
| RNU6-946P | IRX5 | 0.394 | 0.011389688 |
| RNU6-946P | ZNRF1 | 0.395 | 0.01117659 |
| RNU6-946P | SPDYA | 0.395 | 0.01117659 |
| RNU6-946P | IMMP2L | 0.395 | 0.01117659 |
| RNU6-946P | TAS2R14 | 0.395 | 0.01117659 |
| RNU6-946P | TRA2A | 0.396 | 0.010967041 |
| RNU6-946P | TXNL1 | 0.396 | 0.010967041 |
| RNU6-946P | C15orf61 | 0.397 | 0.010760991 |
| RNU6-946P | MRPS36 | 0.397 | 0.010760991 |
| RNU6-946P | ZNF148 | 0.397 | 0.010760991 |
| RNU6-946P | OAZ2 | 0.397 | 0.010760991 |
| RNU6-946P | ZNF709 | 0.397 | 0.010760991 |
| RNU6-946P | CDH2 | 0.397 | 0.010760991 |
| RNU6-946P | H3F3A | 0.397 | 0.010760991 |
| RNU6-946P | RFX3 | 0.397 | 0.010760991 |
| RNU6-946P | CYP26C1 | 0.398 | 0.010558389 |
| RNU6-946P | KLHL15 | 0.398 | 0.010558389 |
| RNU6-946P | LMBR1 | 0.398 | 0.010558389 |
| RNU6-946P | TGIF2 | 0.399 | 0.010359187 |
| RNU6-946P | ASB3 | 0.399 | 0.010359187 |
| RNU6-946P | PHF23 | 0.4 | 0.010163337 |
| RNU6-946P | SFPQ | 0.4 | 0.010163337 |
| RNU6-946P | RPL22 | 0.4 | 0.010163337 |
| RNU6-946P | TMEM165 | 0.4 | 0.010163337 |
| RNU6-946P | FOSB | 0.401 | 0.00997079 |
| RNU6-946P | ODF4 | 0.402 | 0.009781499 |
| RNU6-946P | TXN | 0.402 | 0.009781499 |
| RNU6-946P | TAS2R43 | 0.402 | 0.009781499 |
| RNU6-946P | RPL41 | 0.402 | 0.009781499 |
| RNU6-946P | USP12 | 0.403 | 0.009595418 |
| RNU6-946P | GJB7 | 0.403 | 0.009595418 |
| RNU6-946P | EIF4EBP3 | 0.404 | 0.009412499 |
| RNU6-946P | MEX3B | 0.404 | 0.009412499 |
| RNU6-946P | ZNF410 | 0.404 | 0.009412499 |
| RNU6-946P | METTL25 | 0.405 | 0.009232697 |
| RNU6-946P | RASA1 | 0.405 | 0.009232697 |
| RNU6-946P | IL20RB | 0.405 | 0.009232697 |
| RNU6-946P | KDELR1 | 0.405 | 0.009232697 |
| RNU6-946P | RBM39 | 0.405 | 0.009232697 |
| RNU6-946P | SPCS2 | 0.406 | 0.009055967 |
| RNU6-946P | LRRTM2 | 0.406 | 0.009055967 |
| RNU6-946P | EFNA3 | 0.406 | 0.009055967 |
| RNU6-946P | ATP4A | 0.407 | 0.008882263 |
| RNU6-946P | KPNA5 | 0.407 | 0.008882263 |
| RNU6-946P | CBLB | 0.407 | 0.008882263 |
| RNU6-946P | DYNLL1 | 0.408 | 0.008711542 |
| RNU6-946P | CENPC | 0.408 | 0.008711542 |
| RNU6-946P | SLF1 | 0.408 | 0.008711542 |
| RNU6-946P | RPL35A | 0.408 | 0.008711542 |
| RNU6-946P | PCBP2 | 0.409 | 0.008543759 |
| RNU6-946P | SARNP | 0.409 | 0.008543759 |
| RNU6-946P | FGF11 | 0.409 | 0.008543759 |
| RNU6-946P | SREK1 | 0.41 | 0.008378871 |
| RNU6-946P | MARCKSL1 | 0.41 | 0.008378871 |
| RNU6-946P | ADM5 | 0.41 | 0.008378871 |
| RNU6-946P | TRA2B | 0.411 | 0.008216836 |
| RNU6-946P | TMEM41B | 0.411 | 0.008216836 |
| RNU6-946P | MT-ND5 | 0.412 | 0.00805761 |
| RNU6-946P | KLHL35 | 0.412 | 0.00805761 |
| RNU6-946P | C12orf40 | 0.412 | 0.00805761 |
| RNU6-946P | GCNT7 | 0.413 | 0.007901152 |
| RNU6-946P | ZNF124 | 0.413 | 0.007901152 |
| RNU6-946P | CCNH | 0.413 | 0.007901152 |
| RNU6-946P | STARD3NL | 0.413 | 0.007901152 |
| RNU6-946P | LINC00854 | 0.413 | 0.007901152 |
| RNU6-946P | ERO1B | 0.414 | 0.007747422 |
| RNU6-946P | SLC4A7 | 0.414 | 0.007747422 |
| RNU6-946P | EEF1AKMT2 | 0.414 | 0.007747422 |
| RNU6-946P | AC023055.1 | 0.414 | 0.007747422 |
| RNU6-946P | SLMAP | 0.414 | 0.007747422 |
| RNU6-946P | NDUFA4 | 0.414 | 0.007747422 |
| RNU6-946P | TRDMT1 | 0.414 | 0.007747422 |
| RNU6-946P | LCOR | 0.415 | 0.007596376 |
| RNU6-946P | BAZ2B | 0.415 | 0.007596376 |
| RNU6-946P | AUH | 0.415 | 0.007596376 |
| RNU6-946P | ZMYM2 | 0.416 | 0.007447976 |
| RNU6-946P | MIDN | 0.417 | 0.007302182 |
| RNU6-946P | SEMA5A | 0.417 | 0.007302182 |
| RNU6-946P | LCORL | 0.417 | 0.007302182 |
| RNU6-946P | UBE2V1 | 0.418 | 0.007158953 |
| RNU6-946P | IFRD1 | 0.418 | 0.007158953 |
| RNU6-946P | ASPN | 0.418 | 0.007158953 |
| RNU6-946P | FRYL | 0.418 | 0.007158953 |
| RNU6-946P | IPMK | 0.418 | 0.007158953 |
| RNU6-946P | ZNF782 | 0.418 | 0.007158953 |
| RNU6-946P | NKX6-2 | 0.42 | 0.006880038 |
| RNU6-946P | GSK3B | 0.42 | 0.006880038 |
| RNU6-946P | EID3 | 0.42 | 0.006880038 |
| RNU6-946P | TIAL1 | 0.42 | 0.006880038 |
| RNU6-946P | PBX2 | 0.42 | 0.006880038 |
| RNU6-946P | LRRC18 | 0.42 | 0.006880038 |
| RNU6-946P | ZNF708 | 0.421 | 0.006744275 |
| RNU6-946P | SEC61G | 0.421 | 0.006744275 |
| RNU6-946P | SLC38A9 | 0.421 | 0.006744275 |
| RNU6-946P | FAM196B | 0.422 | 0.006610925 |
| RNU6-946P | ERH | 0.422 | 0.006610925 |
| RNU6-946P | AC069503.2 | 0.422 | 0.006610925 |
| RNU6-946P | PAIP1 | 0.422 | 0.006610925 |
| RNU6-946P | PIN4 | 0.422 | 0.006610925 |
| RNU6-946P | WDR33 | 0.423 | 0.00647995 |
| RNU6-946P | KCNJ1 | 0.424 | 0.006351314 |
| RNU6-946P | ZNF460 | 0.424 | 0.006351314 |
| RNU6-946P | SPINK9 | 0.424 | 0.006351314 |
| RNU6-946P | UBE2D2 | 0.424 | 0.006351314 |
| RNU6-946P | METTL21A | 0.425 | 0.006224981 |
| RNU6-946P | SLC35G6 | 0.425 | 0.006224981 |
| RNU6-946P | PHC3 | 0.425 | 0.006224981 |
| RNU6-946P | COX17 | 0.425 | 0.006224981 |
| RNU6-946P | LMOD2 | 0.426 | 0.006100914 |
| RNU6-946P | BTF3L4 | 0.426 | 0.006100914 |
| RNU6-946P | BMT2 | 0.426 | 0.006100914 |
| RNU6-946P | ZNF800 | 0.426 | 0.006100914 |
| RNU6-946P | TMED6 | 0.427 | 0.00597908 |
| RNU6-946P | GGT6 | 0.427 | 0.00597908 |
| RNU6-946P | C16orf72 | 0.427 | 0.00597908 |
| RNU6-946P | PLEKHG7 | 0.429 | 0.005741966 |
| RNU6-946P | EXOSC6 | 0.429 | 0.005741966 |
| RNU6-946P | TP53TG5 | 0.429 | 0.005741966 |
| RNU6-946P | AC012309.1 | 0.429 | 0.005741966 |
| RNU6-946P | MT-ND4L | 0.43 | 0.005626618 |
| RNU6-946P | TMEM128 | 0.43 | 0.005626618 |
| RNU6-946P | DNAJC19 | 0.43 | 0.005626618 |
| RNU6-946P | CPNE6 | 0.43 | 0.005626618 |
| RNU6-946P | VAMP2 | 0.431 | 0.005513365 |
| RNU6-946P | LSM6 | 0.432 | 0.005402173 |
| RNU6-946P | HNRNPA0 | 0.432 | 0.005402173 |
| RNU6-946P | PPM1A | 0.432 | 0.005402173 |
| RNU6-946P | RLIM | 0.432 | 0.005402173 |
| RNU6-946P | KCNJ6 | 0.433 | 0.00529301 |
| RNU6-946P | DTWD2 | 0.433 | 0.00529301 |
| RNU6-946P | STPG2 | 0.433 | 0.00529301 |
| RNU6-946P | SERPIND1 | 0.434 | 0.005185843 |
| RNU6-946P | RBM14-RBM4 | 0.434 | 0.005185843 |
| RNU6-946P | RCOR3 | 0.434 | 0.005185843 |
| RNU6-946P | PFDN4 | 0.435 | 0.00508064 |
| RNU6-946P | GK3P | 0.435 | 0.00508064 |
| RNU6-946P | TAS2R20 | 0.435 | 0.00508064 |
| RNU6-946P | OCIAD1 | 0.436 | 0.004977371 |
| RNU6-946P | IL31 | 0.437 | 0.004876004 |
| RNU6-946P | ELF2 | 0.437 | 0.004876004 |
| RNU6-946P | HHEX | 0.438 | 0.004776507 |
| RNU6-946P | EFNA4 | 0.439 | 0.004678852 |
| RNU6-946P | GPR27 | 0.44 | 0.004583008 |
| RNU6-946P | RBM4 | 0.44 | 0.004583008 |
| RNU6-946P | RUNDC3B | 0.44 | 0.004583008 |
| RNU6-946P | TSC22D2 | 0.441 | 0.004488945 |
| RNU6-946P | TBCA | 0.441 | 0.004488945 |
| RNU6-946P | ARIH1 | 0.442 | 0.004396635 |
| RNU6-946P | VHL | 0.442 | 0.004396635 |
| RNU6-946P | MT1A | 0.442 | 0.004396635 |
| RNU6-946P | UBE2D3 | 0.442 | 0.004396635 |
| RNU6-946P | METTL21C | 0.443 | 0.004306049 |
| RNU6-946P | MED10 | 0.443 | 0.004306049 |
| RNU6-946P | LRRN4CL | 0.444 | 0.004217158 |
| RNU6-946P | RSRC2 | 0.445 | 0.004129935 |
| RNU6-946P | TAS2R13 | 0.445 | 0.004129935 |
| RNU6-946P | FBXL20 | 0.446 | 0.004044353 |
| RNU6-946P | SETD9 | 0.446 | 0.004044353 |
| RNU6-946P | CASD1 | 0.447 | 0.003960383 |
| RNU6-946P | ZBED6 | 0.449 | 0.003797175 |
| RNU6-946P | GREB1L | 0.449 | 0.003797175 |
| RNU6-946P | SNRPE | 0.449 | 0.003797175 |
| RNU6-946P | LRRC19 | 0.449 | 0.003797175 |
| RNU6-946P | FAM135A | 0.449 | 0.003797175 |
| RNU6-946P | CBX8 | 0.45 | 0.003717885 |
| RNU6-946P | TM2D1 | 0.452 | 0.003563803 |
| RNU6-946P | ZNF644 | 0.452 | 0.003563803 |
| RNU6-946P | ARID2 | 0.453 | 0.003488961 |
| RNU6-946P | SNRPG | 0.453 | 0.003488961 |
| RNU6-946P | TMEM161B | 0.453 | 0.003488961 |
| RNU6-946P | RC3H1 | 0.454 | 0.003415552 |
| RNU6-946P | C16orf87 | 0.454 | 0.003415552 |
| RNU6-946P | COMMD6 | 0.455 | 0.003343552 |
| RNU6-946P | AC005520.1 | 0.456 | 0.003272936 |
| RNU6-946P | MGST2 | 0.458 | 0.003135766 |
| RNU6-946P | MEMO1 | 0.458 | 0.003135766 |
| RNU6-946P | AC093155.3 | 0.458 | 0.003135766 |
| RNU6-946P | RALGAPA1 | 0.459 | 0.003069165 |
| RNU6-946P | MSL1 | 0.46 | 0.003003856 |
| RNU6-946P | MTRNR2L7 | 0.46 | 0.003003856 |
| RNU6-946P | NUP210L | 0.461 | 0.002939817 |
| RNU6-946P | SF3B6 | 0.461 | 0.002939817 |
| RNU6-946P | OMD | 0.462 | 0.002877027 |
| RNU6-946P | ZP2 | 0.462 | 0.002877027 |
| RNU6-946P | SPATA21 | 0.462 | 0.002877027 |
| RNU6-946P | CTAGE5 | 0.462 | 0.002877027 |
| RNU6-946P | ST7L | 0.462 | 0.002877027 |
| RNU6-946P | CDKL2 | 0.464 | 0.002755104 |
| RNU6-946P | NDUFB4 | 0.465 | 0.00269593 |
| RNU6-946P | COX20 | 0.465 | 0.00269593 |
| RNU6-946P | FILIP1L | 0.465 | 0.00269593 |
| RNU6-946P | HACE1 | 0.465 | 0.00269593 |
| RNU6-946P | SUB1 | 0.466 | 0.002637919 |
| RNU6-946P | PABPC4L | 0.466 | 0.002637919 |
| RNU6-946P | RBM7 | 0.466 | 0.002637919 |
| RNU6-946P | ASB15 | 0.468 | 0.002525306 |
| RNU6-946P | INTS6 | 0.468 | 0.002525306 |
| RNU6-946P | LRCH3 | 0.469 | 0.002470665 |
| RNU6-946P | KCNJ9 | 0.47 | 0.002417107 |
| RNU6-946P | SLC35F5 | 0.47 | 0.002417107 |
| RNU6-946P | TTC29 | 0.47 | 0.002417107 |
| RNU6-946P | BBIP1 | 0.472 | 0.002313167 |
| RNU6-946P | GPR75 | 0.472 | 0.002313167 |
| RNU6-946P | GATAD2B | 0.473 | 0.002262747 |
| RNU6-946P | AC013394.1 | 0.476 | 0.002117467 |
| RNU6-946P | BRAF | 0.476 | 0.002117467 |
| RNU6-946P | SLC35G3 | 0.477 | 0.002070974 |
| RNU6-946P | RPS15A | 0.478 | 0.00202542 |
| RNU6-946P | WDPCP | 0.48 | 0.001937058 |
| RNU6-946P | BRWD3 | 0.48 | 0.001937058 |
| RNU6-946P | C8orf59 | 0.482 | 0.001852248 |
| RNU6-946P | C18orf32 | 0.483 | 0.001811135 |
| RNU6-946P | MDFI | 0.485 | 0.001731414 |
| RNU6-946P | MPLKIP | 0.487 | 0.001654931 |
| RNU6-946P | ABRA | 0.487 | 0.001654931 |
| RNU6-946P | SMIM18 | 0.487 | 0.001654931 |
| RNU6-946P | USMG5 | 0.489 | 0.001581568 |
| RNU6-946P | C7orf73 | 0.489 | 0.001581568 |
| RNU6-946P | SUMO2 | 0.492 | 0.001477122 |
| RNU6-946P | TOMM5 | 0.492 | 0.001477122 |
| RNU6-946P | RAB28 | 0.492 | 0.001477122 |
| RNU6-946P | MED30 | 0.493 | 0.001443745 |
| RNU6-946P | CCNG2 | 0.493 | 0.001443745 |
| RNU6-946P | LAPTM4A | 0.494 | 0.001411064 |
| RNU6-946P | DBI | 0.494 | 0.001411064 |
| RNU6-946P | FOXP1 | 0.494 | 0.001411064 |
| RNU6-946P | SIRT1 | 0.496 | 0.001347738 |
| RNU6-946P | MED17 | 0.498 | 0.001287044 |
| RNU6-946P | SMNDC1 | 0.498 | 0.001287044 |
| RNU6-946P | MEX3C | 0.5 | 0.001228881 |
| RNU6-946P | FAM174A | 0.505 | 0.00109393 |
| RNU6-946P | AP002373.1 | 0.505 | 0.00109393 |
| RNU6-946P | KCNJ13 | 0.507 | 0.001043895 |
| RNU6-946P | C11orf95 | 0.507 | 0.001043895 |
| RNU6-946P | TAS2R19 | 0.508 | 0.001019679 |
| RNU6-946P | MAP4K3 | 0.509 | 0.000995984 |
| RNU6-946P | ATP5L | 0.509 | 0.000995984 |
| RNU6-946P | FNIP1 | 0.51 | 0.0009728 |
| RNU6-946P | ZNF738 | 0.511 | 0.000950116 |
| RNU6-946P | SHBG | 0.511 | 0.000950116 |
| RNU6-946P | ANAPC10 | 0.512 | 0.000927924 |
| RNU6-946P | ANKUB1 | 0.513 | 0.000906212 |
| RNU6-946P | TRPC5 | 0.513 | 0.000906212 |
| RNU6-946P | RNU6-202P | 0.513 | 0.000906212 |
| RNU6-946P | ZNF564 | 0.515 | 0.000864194 |
| RNU6-946P | SMIM26 | 0.518 | 0.000804544 |
| RNU6-946P | MTRNR2L3 | 0.518 | 0.000804544 |
| RNU6-946P | TAS2R30 | 0.518 | 0.000804544 |
| RNU6-946P | MAGOH | 0.519 | 0.000785525 |
| RNU6-946P | TOB2 | 0.52 | 0.000766924 |
| RNU6-946P | LRRN3 | 0.52 | 0.000766924 |
| RNU6-946P | MDM4 | 0.521 | 0.000748733 |
| RNU6-946P | C2orf76 | 0.522 | 0.000730944 |
| RNU6-946P | RPS27 | 0.523 | 0.000713547 |
| RNU6-946P | INSM2 | 0.523 | 0.000713547 |
| RNU6-946P | OMG | 0.523 | 0.000713547 |
| RNU6-946P | TAS2R46 | 0.525 | 0.000679903 |
| RNU6-946P | AAMDC | 0.528 | 0.000632191 |
| RNU6-946P | IMMP1L | 0.529 | 0.000616993 |
| RNU6-946P | PNRC1 | 0.533 | 0.000559534 |
| RNU6-946P | CNOT4 | 0.535 | 0.000532712 |
| RNU6-946P | RGS20 | 0.535 | 0.000532712 |
| RNU6-946P | PCF11 | 0.536 | 0.000519755 |
| RNU6-946P | PLEKHA3 | 0.538 | 0.000494717 |
| RNU6-946P | RUFY2 | 0.538 | 0.000494717 |
| RNU6-946P | UBE2B | 0.538 | 0.000494717 |
| RNU6-946P | RAB2B | 0.54 | 0.000470808 |
| RNU6-946P | OCLM | 0.542 | 0.00044798 |
| RNU6-946P | EYS | 0.545 | 0.000415668 |
| RNU6-946P | DENND6A | 0.546 | 0.00040539 |
| RNU6-946P | MAP2K5 | 0.549 | 0.000375963 |
| RNU6-946P | SMIM11B | 0.551 | 0.000357467 |
| RNU6-946P | WASL | 0.56 | 0.000284288 |
| RNU6-946P | KCTD4 | 0.562 | 0.000270057 |
| RNU6-946P | SNX13 | 0.563 | 0.000263194 |
| RNU6-946P | TAS2R31 | 0.566 | 0.000243574 |
| RNU6-946P | ATAD2B | 0.568 | 0.000231265 |
| RNU6-946P | HTR2B | 0.57 | 0.000219542 |
| RNU6-946P | STX19 | 0.571 | 0.000213892 |
| RNU6-946P | FOXF2 | 0.575 | 0.000192629 |
| RNU6-946P | PRR4 | 0.577 | 0.000182758 |
| RNU6-946P | LSM12 | 0.578 | 0.000178003 |
| RNU6-946P | SPIN2A | 0.581 | 0.000164426 |
| RNU6-946P | ANGPTL1 | 0.586 | 0.00014394 |
| RNU6-946P | TAS2R50 | 0.589 | 0.000132829 |
| RNU6-946P | AP003108.2 | 0.59 | 0.000129308 |
| RNU6-946P | YAF2 | 0.591 | 0.000125876 |
| RNU6-946P | PTBP2 | 0.6 | 9.86E-05 |
| RNU6-946P | FOXC1 | 0.611 | 7.29E-05 |
| RNU6-946P | COL10A1 | 0.611 | 7.29E-05 |
| RNU6-946P | CHIC2 | 0.625 | 4.92E-05 |
| RNU6-946P | ACKR4 | 0.631 | 4.15E-05 |
| RNU6-946P | POU5F2 | 0.636 | 3.59E-05 |
| RNU6-946P | MATR3 | 0.64 | 3.20E-05 |
| RNU6-946P | TEX101 | 0.66 | 1.78E-05 |
| RNU6-946P | GPR52 | 0.701 | 5.06E-06 |
| RNU6-946P | GPR22 | 0.706 | 4.32E-06 |
| RNU6-946P | B3GALT2 | 0.707 | 4.18E-06 |
| RNU6-946P | ANGPTL3 | 0.734 | 1.75E-06 |
| RNU6-946P | RNU6-946P | 1 | 6.09E-11 |
| RNU6-761P | EBNA1BP2 | -0.425 | 0.003537245 |
| RNU6-761P | EEF1AKMT1 | -0.418 | 0.004127768 |
| RNU6-761P | MRPS7 | -0.414 | 0.00450407 |
| RNU6-761P | PDIA6 | -0.413 | 0.004602878 |
| RNU6-761P | RANBP1 | -0.41 | 0.004911191 |
| RNU6-761P | MORF4L2 | -0.407 | 0.005238066 |
| RNU6-761P | ILF2 | -0.404 | 0.005584471 |
| RNU6-761P | PARK7 | -0.402 | 0.005826753 |
| RNU6-761P | UCK2 | -0.399 | 0.006207969 |
| RNU6-761P | RAN | -0.392 | 0.007186184 |
| RNU6-761P | TIMM23 | -0.391 | 0.007336688 |
| RNU6-761P | RPL6 | -0.388 | 0.007805316 |
| RNU6-761P | HSP90B1 | -0.388 | 0.007805316 |
| RNU6-761P | RPN2 | -0.387 | 0.007967382 |
| RNU6-761P | METTL1 | -0.387 | 0.007967382 |
| RNU6-761P | CETN2 | -0.386 | 0.008132456 |
| RNU6-761P | COPS3 | -0.382 | 0.008823785 |
| RNU6-761P | RAB32 | -0.382 | 0.008823785 |
| RNU6-761P | RPL4 | -0.38 | 0.009188761 |
| RNU6-761P | MCAT | -0.379 | 0.009376253 |
| RNU6-761P | EIF3I | -0.379 | 0.009376253 |
| RNU6-761P | UTP11 | -0.379 | 0.009376253 |
| RNU6-761P | FDFT1 | -0.378 | 0.009567151 |
| RNU6-761P | ALG5 | -0.376 | 0.009959373 |
| RNU6-761P | PDIA3 | -0.375 | 0.010160804 |
| RNU6-761P | HMGN1 | -0.375 | 0.010160804 |
| RNU6-761P | NOP56 | -0.374 | 0.010365855 |
| RNU6-761P | SNRPD2 | -0.373 | 0.01057458 |
| RNU6-761P | ISOC2 | -0.373 | 0.01057458 |
| RNU6-761P | LTV1 | -0.37 | 0.01122336 |
| RNU6-761P | APEX1 | -0.37 | 0.01122336 |
| RNU6-761P | POLD2 | -0.369 | 0.011447345 |
| RNU6-761P | QPCTL | -0.369 | 0.011447345 |
| RNU6-761P | ALKBH3 | -0.368 | 0.011675289 |
| RNU6-761P | MORN2 | -0.367 | 0.01190725 |
| RNU6-761P | SSR4 | -0.365 | 0.012383463 |
| RNU6-761P | ADSL | -0.364 | 0.012627837 |
| RNU6-761P | CFAP20 | -0.363 | 0.01287647 |
| RNU6-761P | NUDC | -0.363 | 0.01287647 |
| RNU6-761P | C1orf123 | -0.363 | 0.01287647 |
| RNU6-761P | UFC1 | -0.362 | 0.013129424 |
| RNU6-761P | MRPL47 | -0.361 | 0.013386763 |
| RNU6-761P | IFT52 | -0.361 | 0.013386763 |
| RNU6-761P | CWC15 | -0.36 | 0.01364855 |
| RNU6-761P | DRG1 | -0.359 | 0.013914848 |
| RNU6-761P | PSMB7 | -0.357 | 0.01446124 |
| RNU6-761P | CHID1 | -0.357 | 0.01446124 |
| RNU6-761P | MRPL48 | -0.356 | 0.014741466 |
| RNU6-761P | MYDGF | -0.355 | 0.015026466 |
| RNU6-761P | NACA | -0.355 | 0.015026466 |
| RNU6-761P | NDUFS5 | -0.354 | 0.015316308 |
| RNU6-761P | PDCL3 | -0.354 | 0.015316308 |
| RNU6-761P | EMC4 | -0.352 | 0.015910793 |
| RNU6-761P | PHPT1 | -0.351 | 0.016215574 |
| RNU6-761P | PPIA | -0.35 | 0.016525474 |
| RNU6-761P | SRPRB | -0.349 | 0.016840564 |
| RNU6-761P | CCT2 | -0.348 | 0.017160915 |
| RNU6-761P | STT3A | -0.348 | 0.017160915 |
| RNU6-761P | HMGB3 | -0.347 | 0.0174866 |
| RNU6-761P | MMAB | -0.347 | 0.0174866 |
| RNU6-761P | H2AFZ | -0.347 | 0.0174866 |
| RNU6-761P | EEF1B2 | -0.346 | 0.017817691 |
| RNU6-761P | MRPS15 | -0.344 | 0.018496387 |
| RNU6-761P | NONO | -0.343 | 0.018844142 |
| RNU6-761P | IFT122 | -0.342 | 0.019197601 |
| RNU6-761P | SSRP1 | -0.342 | 0.019197601 |
| RNU6-761P | STOML2 | -0.342 | 0.019197601 |
| RNU6-761P | MRPL40 | -0.342 | 0.019197601 |
| RNU6-761P | PLPPR3 | -0.341 | 0.019556842 |
| RNU6-761P | NDUFS3 | -0.341 | 0.019556842 |
| RNU6-761P | CENPW | -0.34 | 0.019921941 |
| RNU6-761P | SF3B5 | -0.34 | 0.019921941 |
| RNU6-761P | ATRAID | -0.34 | 0.019921941 |
| RNU6-761P | RUVBL2 | -0.34 | 0.019921941 |
| RNU6-761P | CINP | -0.34 | 0.019921941 |
| RNU6-761P | MRPL58 | -0.339 | 0.020292976 |
| RNU6-761P | PDHA1 | -0.339 | 0.020292976 |
| RNU6-761P | SEC13 | -0.338 | 0.020670026 |
| RNU6-761P | EXOSC7 | -0.337 | 0.021053169 |
| RNU6-761P | MRPL51 | -0.337 | 0.021053169 |
| RNU6-761P | IQCK | -0.336 | 0.021442486 |
| RNU6-761P | HNRNPA1 | -0.335 | 0.021838056 |
| RNU6-761P | PIWIL4 | -0.335 | 0.021838056 |
| RNU6-761P | RPS8 | -0.335 | 0.021838056 |
| RNU6-761P | ING2 | -0.335 | 0.021838056 |
| RNU6-761P | TMEM147 | -0.335 | 0.021838056 |
| RNU6-761P | HYOU1 | -0.334 | 0.022239961 |
| RNU6-761P | NSMCE1 | -0.333 | 0.022648283 |
| RNU6-761P | ANAPC15 | -0.333 | 0.022648283 |
| RNU6-761P | PPM1G | -0.333 | 0.022648283 |
| RNU6-761P | SRSF7 | -0.332 | 0.023063105 |
| RNU6-761P | CCT7 | -0.332 | 0.023063105 |
| RNU6-761P | RPS6 | -0.331 | 0.023484509 |
| RNU6-761P | MRPL17 | -0.331 | 0.023484509 |
| RNU6-761P | PRDX6 | -0.331 | 0.023484509 |
| RNU6-761P | RPL36AL | -0.331 | 0.023484509 |
| RNU6-761P | ODF2 | -0.33 | 0.02391258 |
| RNU6-761P | MRPL2 | -0.33 | 0.02391258 |
| RNU6-761P | RPL7A | -0.329 | 0.024347401 |
| RNU6-761P | COQ3 | -0.328 | 0.024789059 |
| RNU6-761P | FAM136A | -0.328 | 0.024789059 |
| RNU6-761P | TIMM13 | -0.328 | 0.024789059 |
| RNU6-761P | SLC25A3 | -0.328 | 0.024789059 |
| RNU6-761P | PFDN2 | -0.327 | 0.02523764 |
| RNU6-761P | ERP29 | -0.327 | 0.02523764 |
| RNU6-761P | DNAJB11 | -0.327 | 0.02523764 |
| RNU6-761P | SRSF2 | -0.326 | 0.025693229 |
| RNU6-761P | ERGIC3 | -0.325 | 0.026155915 |
| RNU6-761P | MRFAP1 | -0.325 | 0.026155915 |
| RNU6-761P | COPRS | -0.325 | 0.026155915 |
| RNU6-761P | UBA52 | -0.325 | 0.026155915 |
| RNU6-761P | PPIH | -0.325 | 0.026155915 |
| RNU6-761P | POLE3 | -0.325 | 0.026155915 |
| RNU6-761P | COMT | -0.324 | 0.026625784 |
| RNU6-761P | GPN1 | -0.324 | 0.026625784 |
| RNU6-761P | TMEM138 | -0.324 | 0.026625784 |
| RNU6-761P | TUBA1B | -0.324 | 0.026625784 |
| RNU6-761P | CLEC11A | -0.323 | 0.027102927 |
| RNU6-761P | TIMM50 | -0.323 | 0.027102927 |
| RNU6-761P | MRPL21 | -0.323 | 0.027102927 |
| RNU6-761P | MRPL3 | -0.322 | 0.027587432 |
| RNU6-761P | RPS16 | -0.321 | 0.028079389 |
| RNU6-761P | GCDH | -0.321 | 0.028079389 |
| RNU6-761P | WDR61 | -0.321 | 0.028079389 |
| RNU6-761P | POLR1C | -0.321 | 0.028079389 |
| RNU6-761P | ATP5A1 | -0.321 | 0.028079389 |
| RNU6-761P | ERI3 | -0.32 | 0.028578889 |
| RNU6-761P | LAGE3 | -0.319 | 0.029086023 |
| RNU6-761P | NCBP2-AS2 | -0.319 | 0.029086023 |
| RNU6-761P | RUVBL1 | -0.319 | 0.029086023 |
| RNU6-761P | YBX1 | -0.319 | 0.029086023 |
| RNU6-761P | PSMC3 | -0.318 | 0.029600883 |
| RNU6-761P | FBL | -0.318 | 0.029600883 |
| RNU6-761P | NSDHL | -0.318 | 0.029600883 |
| RNU6-761P | RPS3A | -0.318 | 0.029600883 |
| RNU6-761P | RPL14 | -0.318 | 0.029600883 |
| RNU6-761P | ALG3 | -0.318 | 0.029600883 |
| RNU6-761P | NPM3 | -0.318 | 0.029600883 |
| RNU6-761P | HDDC2 | -0.317 | 0.030123561 |
| RNU6-761P | P3H1 | -0.317 | 0.030123561 |
| RNU6-761P | TECR | -0.317 | 0.030123561 |
| RNU6-761P | PAK1IP1 | -0.317 | 0.030123561 |
| RNU6-761P | DDX27 | -0.317 | 0.030123561 |
| RNU6-761P | PARL | -0.316 | 0.030654152 |
| RNU6-761P | UQCRFS1 | -0.316 | 0.030654152 |
| RNU6-761P | NUCB2 | -0.316 | 0.030654152 |
| RNU6-761P | GGH | -0.316 | 0.030654152 |
| RNU6-761P | FARSA | -0.315 | 0.031192748 |
| RNU6-761P | CANX | -0.315 | 0.031192748 |
| RNU6-761P | UQCRH | -0.315 | 0.031192748 |
| RNU6-761P | MIF4GD | -0.314 | 0.031739445 |
| RNU6-761P | CHCHD2 | -0.314 | 0.031739445 |
| RNU6-761P | SMS | -0.314 | 0.031739445 |
| RNU6-761P | PPA2 | -0.313 | 0.032294338 |
| RNU6-761P | ST3GAL3 | -0.313 | 0.032294338 |
| RNU6-761P | ECHS1 | -0.313 | 0.032294338 |
| RNU6-761P | USE1 | -0.313 | 0.032294338 |
| RNU6-761P | TOMM22 | -0.313 | 0.032294338 |
| RNU6-761P | RPL5 | -0.313 | 0.032294338 |
| RNU6-761P | NOP58 | -0.312 | 0.032857521 |
| RNU6-761P | MRPL57 | -0.312 | 0.032857521 |
| RNU6-761P | MRPS9 | -0.312 | 0.032857521 |
| RNU6-761P | CCDC189 | -0.312 | 0.032857521 |
| RNU6-761P | RPSA | -0.312 | 0.032857521 |
| RNU6-761P | POLR2I | -0.311 | 0.033429093 |
| RNU6-761P | MTX2 | -0.311 | 0.033429093 |
| RNU6-761P | SNRPD3 | -0.31 | 0.03400915 |
| RNU6-761P | EXOSC1 | -0.31 | 0.03400915 |
| RNU6-761P | PA2G4 | -0.31 | 0.03400915 |
| RNU6-761P | RPL19 | -0.309 | 0.034597789 |
| RNU6-761P | OSTC | -0.309 | 0.034597789 |
| RNU6-761P | C21orf59 | -0.309 | 0.034597789 |
| RNU6-761P | GPI | -0.309 | 0.034597789 |
| RNU6-761P | NDUFB9 | -0.308 | 0.03519511 |
| RNU6-761P | XRCC6 | -0.308 | 0.03519511 |
| RNU6-761P | DNAJC8 | -0.308 | 0.03519511 |
| RNU6-761P | RNASEH2A | -0.308 | 0.03519511 |
| RNU6-761P | SLC35F2 | -0.308 | 0.03519511 |
| RNU6-761P | NDUFAF3 | -0.308 | 0.03519511 |
| RNU6-761P | PRMT1 | -0.308 | 0.03519511 |
| RNU6-761P | NME1 | -0.307 | 0.03580121 |
| RNU6-761P | NELFE | -0.307 | 0.03580121 |
| RNU6-761P | NASP | -0.307 | 0.03580121 |
| RNU6-761P | NOP2 | -0.307 | 0.03580121 |
| RNU6-761P | SSU72 | -0.307 | 0.03580121 |
| RNU6-761P | WDR83OS | -0.307 | 0.03580121 |
| RNU6-761P | GLA | -0.306 | 0.03641619 |
| RNU6-761P | RIOK1 | -0.306 | 0.03641619 |
| RNU6-761P | CUTA | -0.306 | 0.03641619 |
| RNU6-761P | IFRD2 | -0.306 | 0.03641619 |
| RNU6-761P | PHKG2 | -0.306 | 0.03641619 |
| RNU6-761P | C16orf74 | -0.306 | 0.03641619 |
| RNU6-761P | KTI12 | -0.306 | 0.03641619 |
| RNU6-761P | ADI1 | -0.306 | 0.03641619 |
| RNU6-761P | KPNA2 | -0.306 | 0.03641619 |
| RNU6-761P | BOLA2B | -0.305 | 0.037040149 |
| RNU6-761P | RPLP0 | -0.305 | 0.037040149 |
| RNU6-761P | C19orf24 | -0.305 | 0.037040149 |
| RNU6-761P | PNP | -0.305 | 0.037040149 |
| RNU6-761P | POP5 | -0.304 | 0.037673189 |
| RNU6-761P | SNRPD1 | -0.304 | 0.037673189 |
| RNU6-761P | IMP4 | -0.304 | 0.037673189 |
| RNU6-761P | SLC2A4RG | -0.304 | 0.037673189 |
| RNU6-761P | ELP5 | -0.304 | 0.037673189 |
| RNU6-761P | ATP23 | -0.304 | 0.037673189 |
| RNU6-761P | MRPL11 | -0.303 | 0.03831541 |
| RNU6-761P | MRPL20 | -0.303 | 0.03831541 |
| RNU6-761P | MLEC | -0.303 | 0.03831541 |
| RNU6-761P | MEA1 | -0.303 | 0.03831541 |
| RNU6-761P | MRPS22 | -0.302 | 0.038966915 |
| RNU6-761P | RPL11 | -0.302 | 0.038966915 |
| RNU6-761P | CCER2 | -0.302 | 0.038966915 |
| RNU6-761P | RPS21 | -0.302 | 0.038966915 |
| RNU6-761P | MRTO4 | -0.302 | 0.038966915 |
| RNU6-761P | RFC4 | -0.302 | 0.038966915 |
| RNU6-761P | NMB | -0.302 | 0.038966915 |
| RNU6-761P | ATP5O | -0.302 | 0.038966915 |
| RNU6-761P | PSMB6 | -0.302 | 0.038966915 |
| RNU6-761P | HNRNPDL | -0.301 | 0.039627806 |
| RNU6-761P | BDH1 | -0.301 | 0.039627806 |
| RNU6-761P | NDUFAF8 | -0.301 | 0.039627806 |
| RNU6-761P | PEBP1 | -0.301 | 0.039627806 |
| RNU6-761P | MIS18A | -0.301 | 0.039627806 |
| RNU6-761P | FDPS | -0.3 | 0.040298186 |
| RNU6-761P | SNRPF | -0.3 | 0.040298186 |
| RNU6-761P | DKC1 | -0.3 | 0.040298186 |
| RNU6-761P | SRP68 | -0.3 | 0.040298186 |
| RNU6-761P | TMX2 | -0.3 | 0.040298186 |
| RNU6-761P | CDK4 | -0.299 | 0.040978159 |
| RNU6-761P | SRSF3 | -0.299 | 0.040978159 |
| RNU6-761P | SH3KBP1 | -0.299 | 0.040978159 |
| RNU6-761P | ADPRHL2 | -0.299 | 0.040978159 |
| RNU6-761P | GNL2 | -0.299 | 0.040978159 |
| RNU6-761P | TMEM216 | -0.298 | 0.041667828 |
| RNU6-761P | FRG1 | -0.298 | 0.041667828 |
| RNU6-761P | DARS | -0.298 | 0.041667828 |
| RNU6-761P | RPL15 | -0.298 | 0.041667828 |
| RNU6-761P | APOO | -0.298 | 0.041667828 |
| RNU6-761P | C19orf48 | -0.297 | 0.042367299 |
| RNU6-761P | MRPL27 | -0.297 | 0.042367299 |
| RNU6-761P | TUFM | -0.297 | 0.042367299 |
| RNU6-761P | UQCC3 | -0.297 | 0.042367299 |
| RNU6-761P | C1orf228 | -0.297 | 0.042367299 |
| RNU6-761P | RPL10A | -0.297 | 0.042367299 |
| RNU6-761P | KIF22 | -0.297 | 0.042367299 |
| RNU6-761P | TIMMDC1 | -0.296 | 0.043076676 |
| RNU6-761P | GADD45GIP1 | -0.296 | 0.043076676 |
| RNU6-761P | ALKBH7 | -0.296 | 0.043076676 |
| RNU6-761P | UQCRQ | -0.296 | 0.043076676 |
| RNU6-761P | RPS7 | -0.296 | 0.043076676 |
| RNU6-761P | AAMP | -0.296 | 0.043076676 |
| RNU6-761P | NCOA4 | -0.295 | 0.043796066 |
| RNU6-761P | ARMCX6 | -0.295 | 0.043796066 |
| RNU6-761P | RPL29 | -0.295 | 0.043796066 |
| RNU6-761P | PKP4 | -0.295 | 0.043796066 |
| RNU6-761P | TIMM17A | -0.294 | 0.044525575 |
| RNU6-761P | NOP16 | -0.294 | 0.044525575 |
| RNU6-761P | BTF3 | -0.294 | 0.044525575 |
| RNU6-761P | SUCLG1 | -0.294 | 0.044525575 |
| RNU6-761P | GORASP2 | -0.294 | 0.044525575 |
| RNU6-761P | HSD17B10 | -0.294 | 0.044525575 |
| RNU6-761P | METTL5 | -0.294 | 0.044525575 |
| RNU6-761P | CCT5 | -0.294 | 0.044525575 |
| RNU6-761P | UXT | -0.294 | 0.044525575 |
| RNU6-761P | RNASE2 | -0.294 | 0.044525575 |
| RNU6-761P | ATP5B | -0.294 | 0.044525575 |
| RNU6-761P | GAPDH | -0.294 | 0.044525575 |
| RNU6-761P | RPL21 | -0.293 | 0.04526531 |
| RNU6-761P | STX18 | -0.293 | 0.04526531 |
| RNU6-761P | ILF3 | -0.293 | 0.04526531 |
| RNU6-761P | TOE1 | -0.293 | 0.04526531 |
| RNU6-761P | TCP1 | -0.292 | 0.046015377 |
| RNU6-761P | RPL32 | -0.292 | 0.046015377 |
| RNU6-761P | CITED4 | -0.292 | 0.046015377 |
| RNU6-761P | JMJD8 | -0.292 | 0.046015377 |
| RNU6-761P | RNF113A | -0.292 | 0.046015377 |
| RNU6-761P | SAPCD1 | -0.292 | 0.046015377 |
| RNU6-761P | SMIM19 | -0.292 | 0.046015377 |
| RNU6-761P | PDCD5 | -0.292 | 0.046015377 |
| RNU6-761P | RPS5 | -0.292 | 0.046015377 |
| RNU6-761P | TMEM5 | -0.292 | 0.046015377 |
| RNU6-761P | B4GALT2 | -0.292 | 0.046015377 |
| RNU6-761P | EIF4A3 | -0.292 | 0.046015377 |
| RNU6-761P | EIF6 | -0.292 | 0.046015377 |
| RNU6-761P | CALR | -0.291 | 0.046775885 |
| RNU6-761P | BAG1 | -0.291 | 0.046775885 |
| RNU6-761P | FANCG | -0.291 | 0.046775885 |
| RNU6-761P | REEP6 | -0.291 | 0.046775885 |
| RNU6-761P | PRSS57 | -0.291 | 0.046775885 |
| RNU6-761P | MBD4 | -0.291 | 0.046775885 |
| RNU6-761P | PRPF4 | -0.29 | 0.047546943 |
| RNU6-761P | ZNHIT2 | -0.29 | 0.047546943 |
| RNU6-761P | GNL3 | -0.29 | 0.047546943 |
| RNU6-761P | RPL23A | -0.29 | 0.047546943 |
| RNU6-761P | APRT | -0.289 | 0.048328658 |
| RNU6-761P | SNU13 | -0.289 | 0.048328658 |
| RNU6-761P | APMAP | -0.289 | 0.048328658 |
| RNU6-761P | TOMM40 | -0.289 | 0.048328658 |
| RNU6-761P | CENPV | -0.289 | 0.048328658 |
| RNU6-761P | POP4 | -0.289 | 0.048328658 |
| RNU6-761P | SARS2 | -0.289 | 0.048328658 |
| RNU6-761P | TCTN2 | -0.288 | 0.049121141 |
| RNU6-761P | C19orf44 | -0.288 | 0.049121141 |
| RNU6-761P | PEMT | -0.288 | 0.049121141 |
| RNU6-761P | HNRNPL | -0.288 | 0.049121141 |
| RNU6-761P | TIMM21 | -0.288 | 0.049121141 |
| RNU6-761P | DPM3 | -0.288 | 0.049121141 |
| RNU6-761P | EEF1G | -0.288 | 0.049121141 |
| RNU6-761P | HNRNPD | -0.288 | 0.049121141 |
| RNU6-761P | CKS1B | -0.287 | 0.0499245 |
| RNU6-761P | LMAN2L | -0.287 | 0.0499245 |
| RNU6-761P | SRSF9 | -0.287 | 0.0499245 |
| RNU6-761P | RPLP2 | -0.287 | 0.0499245 |
| RNU6-761P | SAP18 | -0.287 | 0.0499245 |
| RNU6-761P | NARS | -0.287 | 0.0499245 |
| RNU6-761P | TST | -0.287 | 0.0499245 |
| RNU6-761P | FUNDC2 | -0.287 | 0.0499245 |
| RNU6-761P | MZT2B | -0.287 | 0.0499245 |
| RNU6-761P | H2AFX | -0.287 | 0.0499245 |
| RNU6-761P | REPS2 | 0.279 | 0.049895777 |
| RNU6-761P | GRIA4 | 0.279 | 0.049895777 |
| RNU6-761P | ABRA | 0.279 | 0.049895777 |
| RNU6-761P | SYT11 | 0.279 | 0.049895777 |
| RNU6-761P | FAM126A | 0.279 | 0.049895777 |
| RNU6-761P | IL27RA | 0.279 | 0.049895777 |
| RNU6-761P | ZYG11B | 0.279 | 0.049895777 |
| RNU6-761P | ZNF510 | 0.279 | 0.049895777 |
| RNU6-761P | RNF145 | 0.279 | 0.049895777 |
| RNU6-761P | SECISBP2L | 0.279 | 0.049895777 |
| RNU6-761P | DICER1 | 0.279 | 0.049895777 |
| RNU6-761P | UBE2V1 | 0.28 | 0.049092806 |
| RNU6-761P | TRAT1 | 0.28 | 0.049092806 |
| RNU6-761P | ARG2 | 0.28 | 0.049092806 |
| RNU6-761P | SEC14L1 | 0.28 | 0.049092806 |
| RNU6-761P | TCAF2 | 0.28 | 0.049092806 |
| RNU6-761P | GRIP2 | 0.28 | 0.049092806 |
| RNU6-761P | AANAT | 0.281 | 0.048300707 |
| RNU6-761P | SENP7 | 0.281 | 0.048300707 |
| RNU6-761P | PPP2R5C | 0.281 | 0.048300707 |
| RNU6-761P | ZNF808 | 0.281 | 0.048300707 |
| RNU6-761P | WIPF1 | 0.281 | 0.048300707 |
| RNU6-761P | ZNF821 | 0.281 | 0.048300707 |
| RNU6-761P | NFAT5 | 0.282 | 0.047519372 |
| RNU6-761P | POU5F2 | 0.282 | 0.047519372 |
| RNU6-761P | RFTN1 | 0.282 | 0.047519372 |
| RNU6-761P | ARID2 | 0.282 | 0.047519372 |
| RNU6-761P | TAS2R31 | 0.282 | 0.047519372 |
| RNU6-761P | IFIH1 | 0.282 | 0.047519372 |
| RNU6-761P | ZNF148 | 0.282 | 0.047519372 |
| RNU6-761P | SLFN5 | 0.282 | 0.047519372 |
| RNU6-761P | CLMP | 0.282 | 0.047519372 |
| RNU6-761P | DISP1 | 0.282 | 0.047519372 |
| RNU6-761P | AC020636.2 | 0.283 | 0.046748692 |
| RNU6-761P | ROCK1 | 0.283 | 0.046748692 |
| RNU6-761P | CDK17 | 0.283 | 0.046748692 |
| RNU6-761P | MYOT | 0.283 | 0.046748692 |
| RNU6-761P | HAPLN3 | 0.283 | 0.046748692 |
| RNU6-761P | ZNF490 | 0.283 | 0.046748692 |
| RNU6-761P | TXNRD3 | 0.283 | 0.046748692 |
| RNU6-761P | FNIP2 | 0.284 | 0.045988556 |
| RNU6-761P | CLEC9A | 0.284 | 0.045988556 |
| RNU6-761P | STK32B | 0.284 | 0.045988556 |
| RNU6-761P | ZNF101 | 0.284 | 0.045988556 |
| RNU6-761P | SPINK9 | 0.284 | 0.045988556 |
| RNU6-761P | STK4 | 0.284 | 0.045988556 |
| RNU6-761P | FNDC5 | 0.284 | 0.045988556 |
| RNU6-761P | AFF4 | 0.284 | 0.045988556 |
| RNU6-761P | CDKL1 | 0.285 | 0.045238857 |
| RNU6-761P | MERTK | 0.285 | 0.045238857 |
| RNU6-761P | CEP135 | 0.285 | 0.045238857 |
| RNU6-761P | LRBA | 0.285 | 0.045238857 |
| RNU6-761P | RNF111 | 0.285 | 0.045238857 |
| RNU6-761P | TAS2R30 | 0.285 | 0.045238857 |
| RNU6-761P | EZH1 | 0.285 | 0.045238857 |
| RNU6-761P | SLC5A11 | 0.286 | 0.044499488 |
| RNU6-761P | ATL1 | 0.286 | 0.044499488 |
| RNU6-761P | SBF2 | 0.286 | 0.044499488 |
| RNU6-761P | HCFC2 | 0.286 | 0.044499488 |
| RNU6-761P | KAT6A | 0.286 | 0.044499488 |
| RNU6-761P | GIMAP6 | 0.286 | 0.044499488 |
| RNU6-761P | KAT2B | 0.286 | 0.044499488 |
| RNU6-761P | PSD3 | 0.287 | 0.04377034 |
| RNU6-761P | TMEM63A | 0.287 | 0.04377034 |
| RNU6-761P | PVRIG | 0.287 | 0.04377034 |
| RNU6-761P | CPEB3 | 0.287 | 0.04377034 |
| RNU6-761P | OR2AK2 | 0.287 | 0.04377034 |
| RNU6-761P | ABLIM2 | 0.287 | 0.04377034 |
| RNU6-761P | AKAP13 | 0.287 | 0.04377034 |
| RNU6-761P | TNFSF4 | 0.288 | 0.043051308 |
| RNU6-761P | PANK3 | 0.288 | 0.043051308 |
| RNU6-761P | ROBO4 | 0.288 | 0.043051308 |
| RNU6-761P | ADGRV1 | 0.288 | 0.043051308 |
| RNU6-761P | STX7 | 0.288 | 0.043051308 |
| RNU6-761P | TAP2 | 0.289 | 0.042342284 |
| RNU6-761P | VWA3A | 0.289 | 0.042342284 |
| RNU6-761P | MAP3K5 | 0.289 | 0.042342284 |
| RNU6-761P | ARHGAP20 | 0.29 | 0.041643163 |
| RNU6-761P | ZNF677 | 0.29 | 0.041643163 |
| RNU6-761P | ERGIC1 | 0.29 | 0.041643163 |
| RNU6-761P | AC119396.1 | 0.29 | 0.041643163 |
| RNU6-761P | SNAPC3 | 0.291 | 0.04095384 |
| RNU6-761P | TRPM7 | 0.291 | 0.04095384 |
| RNU6-761P | SLC25A30 | 0.291 | 0.04095384 |
| RNU6-761P | ZNF91 | 0.291 | 0.04095384 |
| RNU6-761P | SGPL1 | 0.291 | 0.04095384 |
| RNU6-761P | NPC1 | 0.291 | 0.04095384 |
| RNU6-761P | OR2L3 | 0.291 | 0.04095384 |
| RNU6-761P | ULK2 | 0.291 | 0.04095384 |
| RNU6-761P | VWC2L | 0.291 | 0.04095384 |
| RNU6-761P | KANSL1 | 0.291 | 0.04095384 |
| RNU6-761P | ATF7IP | 0.292 | 0.04027421 |
| RNU6-761P | PITPNC1 | 0.292 | 0.04027421 |
| RNU6-761P | XPR1 | 0.292 | 0.04027421 |
| RNU6-761P | ITCH | 0.292 | 0.04027421 |
| RNU6-761P | CLEC16A | 0.292 | 0.04027421 |
| RNU6-761P | TAS2R13 | 0.292 | 0.04027421 |
| RNU6-761P | IL6ST | 0.293 | 0.039604169 |
| RNU6-761P | ASCC1 | 0.293 | 0.039604169 |
| RNU6-761P | MMP28 | 0.293 | 0.039604169 |
| RNU6-761P | CSRNP2 | 0.293 | 0.039604169 |
| RNU6-761P | KCTD4 | 0.293 | 0.039604169 |
| RNU6-761P | TCF12 | 0.293 | 0.039604169 |
| RNU6-761P | ATAD2B | 0.294 | 0.038943613 |
| RNU6-761P | GLIPR1L1 | 0.294 | 0.038943613 |
| RNU6-761P | TAS2R19 | 0.294 | 0.038943613 |
| RNU6-761P | MAOA | 0.294 | 0.038943613 |
| RNU6-761P | CEMIP | 0.294 | 0.038943613 |
| RNU6-761P | CD163L1 | 0.295 | 0.03829244 |
| RNU6-761P | ASH1L | 0.295 | 0.03829244 |
| RNU6-761P | ATF7 | 0.295 | 0.03829244 |
| RNU6-761P | CMKLR1 | 0.295 | 0.03829244 |
| RNU6-761P | NPR3 | 0.295 | 0.03829244 |
| RNU6-761P | ZNF652 | 0.296 | 0.037650547 |
| RNU6-761P | FAM214A | 0.296 | 0.037650547 |
| RNU6-761P | BAZ2A | 0.297 | 0.037017831 |
| RNU6-761P | FHOD3 | 0.297 | 0.037017831 |
| RNU6-761P | PRH2 | 0.297 | 0.037017831 |
| RNU6-761P | FAM171B | 0.297 | 0.037017831 |
| RNU6-761P | STX17 | 0.297 | 0.037017831 |
| RNU6-761P | RALGAPB | 0.297 | 0.037017831 |
| RNU6-761P | TFPI | 0.297 | 0.037017831 |
| RNU6-761P | DAPK1 | 0.298 | 0.036394192 |
| RNU6-761P | SUSD4 | 0.298 | 0.036394192 |
| RNU6-761P | SAMD9 | 0.298 | 0.036394192 |
| RNU6-761P | XYLT1 | 0.298 | 0.036394192 |
| RNU6-761P | XIAP | 0.298 | 0.036394192 |
| RNU6-761P | SLC35F4 | 0.298 | 0.036394192 |
| RNU6-761P | KLHL29 | 0.298 | 0.036394192 |
| RNU6-761P | ANGPTL7 | 0.299 | 0.03577953 |
| RNU6-761P | SPINT1 | 0.299 | 0.03577953 |
| RNU6-761P | SCN3A | 0.299 | 0.03577953 |
| RNU6-761P | TRPS1 | 0.299 | 0.03577953 |
| RNU6-761P | ZBTB16 | 0.299 | 0.03577953 |
| RNU6-761P | RIC1 | 0.299 | 0.03577953 |
| RNU6-761P | TMLHE | 0.299 | 0.03577953 |
| RNU6-761P | ZNF611 | 0.299 | 0.03577953 |
| RNU6-761P | STAC | 0.3 | 0.035173743 |
| RNU6-761P | BCAS4 | 0.3 | 0.035173743 |
| RNU6-761P | GAN | 0.3 | 0.035173743 |
| RNU6-761P | MAP4K5 | 0.3 | 0.035173743 |
| RNU6-761P | KCNJ2 | 0.3 | 0.035173743 |
| RNU6-761P | USF3 | 0.301 | 0.034576733 |
| RNU6-761P | PTTG2 | 0.301 | 0.034576733 |
| RNU6-761P | HERC3 | 0.301 | 0.034576733 |
| RNU6-761P | CNOT6L | 0.301 | 0.034576733 |
| RNU6-761P | SH3BP5 | 0.301 | 0.034576733 |
| RNU6-761P | SP4 | 0.301 | 0.034576733 |
| RNU6-761P | ADAMTS6 | 0.301 | 0.034576733 |
| RNU6-761P | OR2L5 | 0.301 | 0.034576733 |
| RNU6-761P | APC | 0.301 | 0.034576733 |
| RNU6-761P | C4orf22 | 0.301 | 0.034576733 |
| RNU6-761P | SP110 | 0.302 | 0.0339884 |
| RNU6-761P | SH3TC1 | 0.302 | 0.0339884 |
| RNU6-761P | KIAA1109 | 0.302 | 0.0339884 |
| RNU6-761P | MBP | 0.303 | 0.033408646 |
| RNU6-761P | RICTOR | 0.303 | 0.033408646 |
| RNU6-761P | THSD7A | 0.303 | 0.033408646 |
| RNU6-761P | MBD5 | 0.303 | 0.033408646 |
| RNU6-761P | USP15 | 0.303 | 0.033408646 |
| RNU6-761P | SCAF8 | 0.303 | 0.033408646 |
| RNU6-761P | AC131097.2 | 0.303 | 0.033408646 |
| RNU6-761P | TIMP4 | 0.303 | 0.033408646 |
| RNU6-761P | FBXO11 | 0.303 | 0.033408646 |
| RNU6-761P | LIMS1 | 0.303 | 0.033408646 |
| RNU6-761P | SLC9A7 | 0.304 | 0.032837374 |
| RNU6-761P | UST | 0.304 | 0.032837374 |
| RNU6-761P | FMN1 | 0.304 | 0.032837374 |
| RNU6-761P | P2RY12 | 0.304 | 0.032837374 |
| RNU6-761P | CPED1 | 0.304 | 0.032837374 |
| RNU6-761P | SEMA4F | 0.305 | 0.032274486 |
| RNU6-761P | ZNF81 | 0.305 | 0.032274486 |
| RNU6-761P | CEP350 | 0.305 | 0.032274486 |
| RNU6-761P | FER1L6 | 0.305 | 0.032274486 |
| RNU6-761P | RCAN1 | 0.305 | 0.032274486 |
| RNU6-761P | DIRC3 | 0.306 | 0.031719886 |
| RNU6-761P | AIDA | 0.306 | 0.031719886 |
| RNU6-761P | FAM212B | 0.306 | 0.031719886 |
| RNU6-761P | PHF2 | 0.306 | 0.031719886 |
| RNU6-761P | PREX1 | 0.307 | 0.031173479 |
| RNU6-761P | GIMAP2 | 0.307 | 0.031173479 |
| RNU6-761P | CYP3A5 | 0.307 | 0.031173479 |
| RNU6-761P | MINDY4B | 0.307 | 0.031173479 |
| RNU6-761P | ST3GAL1 | 0.307 | 0.031173479 |
| RNU6-761P | MED13 | 0.308 | 0.030635168 |
| RNU6-761P | CCDC186 | 0.308 | 0.030635168 |
| RNU6-761P | LRRTM2 | 0.308 | 0.030635168 |
| RNU6-761P | OR5C1 | 0.308 | 0.030635168 |
| RNU6-761P | AVPR1B | 0.309 | 0.03010486 |
| RNU6-761P | COL24A1 | 0.309 | 0.03010486 |
| RNU6-761P | MED13L | 0.309 | 0.03010486 |
| RNU6-761P | HELZ | 0.309 | 0.03010486 |
| RNU6-761P | DMXL1 | 0.309 | 0.03010486 |
| RNU6-761P | PLXNC1 | 0.309 | 0.03010486 |
| RNU6-761P | ITSN2 | 0.309 | 0.03010486 |
| RNU6-761P | ADAMTS2 | 0.309 | 0.03010486 |
| RNU6-761P | GRK3 | 0.309 | 0.03010486 |
| RNU6-761P | TCF20 | 0.309 | 0.03010486 |
| RNU6-761P | MLLT3 | 0.31 | 0.029582461 |
| RNU6-761P | SNTB2 | 0.31 | 0.029582461 |
| RNU6-761P | SERPIND1 | 0.31 | 0.029582461 |
| RNU6-761P | ATP7A | 0.31 | 0.029582461 |
| RNU6-761P | HIPK2 | 0.31 | 0.029582461 |
| RNU6-761P | RNU6-1143P | 0.31 | 0.029582461 |
| RNU6-761P | INPP5F | 0.311 | 0.029067877 |
| RNU6-761P | RASA2 | 0.311 | 0.029067877 |
| RNU6-761P | PZP | 0.311 | 0.029067877 |
| RNU6-761P | ARHGEF3 | 0.311 | 0.029067877 |
| RNU6-761P | TTPAL | 0.311 | 0.029067877 |
| RNU6-761P | SMAP2 | 0.311 | 0.029067877 |
| RNU6-761P | BIRC6 | 0.311 | 0.029067877 |
| RNU6-761P | ELK3 | 0.311 | 0.029067877 |
| RNU6-761P | CDYL2 | 0.312 | 0.028561016 |
| RNU6-761P | ZNF235 | 0.312 | 0.028561016 |
| RNU6-761P | MECP2 | 0.313 | 0.028061786 |
| RNU6-761P | TAB2 | 0.313 | 0.028061786 |
| RNU6-761P | COL5A1 | 0.313 | 0.028061786 |
| RNU6-761P | MARF1 | 0.313 | 0.028061786 |
| RNU6-761P | KMT2C | 0.313 | 0.028061786 |
| RNU6-761P | SYNE1 | 0.313 | 0.028061786 |
| RNU6-761P | MEF2A | 0.314 | 0.027570095 |
| RNU6-761P | KIAA1462 | 0.314 | 0.027570095 |
| RNU6-761P | BORCS5 | 0.314 | 0.027570095 |
| RNU6-761P | FBXL4 | 0.314 | 0.027570095 |
| RNU6-761P | LINC00854 | 0.314 | 0.027570095 |
| RNU6-761P | ZNF782 | 0.314 | 0.027570095 |
| RNU6-761P | CLCC1 | 0.315 | 0.027085853 |
| RNU6-761P | GBP4 | 0.315 | 0.027085853 |
| RNU6-761P | PTPN4 | 0.315 | 0.027085853 |
| RNU6-761P | PRKCE | 0.315 | 0.027085853 |
| RNU6-761P | APPL2 | 0.315 | 0.027085853 |
| RNU6-761P | SLC12A6 | 0.315 | 0.027085853 |
| RNU6-761P | USP9X | 0.316 | 0.02660897 |
| RNU6-761P | UBR3 | 0.316 | 0.02660897 |
| RNU6-761P | STAG1 | 0.316 | 0.02660897 |
| RNU6-761P | GIMAP5 | 0.316 | 0.02660897 |
| RNU6-761P | TPST1 | 0.316 | 0.02660897 |
| RNU6-761P | TFCP2L1 | 0.317 | 0.026139357 |
| RNU6-761P | PAG1 | 0.317 | 0.026139357 |
| RNU6-761P | DNAH9 | 0.317 | 0.026139357 |
| RNU6-761P | SRGAP1 | 0.317 | 0.026139357 |
| RNU6-761P | RNF38 | 0.317 | 0.026139357 |
| RNU6-761P | GAB1 | 0.317 | 0.026139357 |
| RNU6-761P | DLG3 | 0.318 | 0.025676925 |
| RNU6-761P | MAML2 | 0.318 | 0.025676925 |
| RNU6-761P | SCRG1 | 0.318 | 0.025676925 |
| RNU6-761P | SETX | 0.318 | 0.025676925 |
| RNU6-761P | CREBBP | 0.318 | 0.025676925 |
| RNU6-761P | HFE2 | 0.318 | 0.025676925 |
| RNU6-761P | CRTC3 | 0.318 | 0.025676925 |
| RNU6-761P | NUDT16 | 0.319 | 0.025221586 |
| RNU6-761P | CNOT4 | 0.319 | 0.025221586 |
| RNU6-761P | TXNIP | 0.319 | 0.025221586 |
| RNU6-761P | RASGRP1 | 0.319 | 0.025221586 |
| RNU6-761P | TMEM106A | 0.319 | 0.025221586 |
| RNU6-761P | NAT8 | 0.32 | 0.024773253 |
| RNU6-761P | ARMC2 | 0.32 | 0.024773253 |
| RNU6-761P | RARRES1 | 0.32 | 0.024773253 |
| RNU6-761P | KCNE1 | 0.32 | 0.024773253 |
| RNU6-761P | RUBCN | 0.321 | 0.02433184 |
| RNU6-761P | RGP1 | 0.321 | 0.02433184 |
| RNU6-761P | PHTF2 | 0.321 | 0.02433184 |
| RNU6-761P | KMT2A | 0.321 | 0.02433184 |
| RNU6-761P | LMOD3 | 0.321 | 0.02433184 |
| RNU6-761P | ATM | 0.322 | 0.023897259 |
| RNU6-761P | GIF | 0.322 | 0.023897259 |
| RNU6-761P | VAV3 | 0.322 | 0.023897259 |
| RNU6-761P | HERC4 | 0.322 | 0.023897259 |
| RNU6-761P | TCTEX1D1 | 0.322 | 0.023897259 |
| RNU6-761P | CCDC7 | 0.323 | 0.023469427 |
| RNU6-761P | PARP14 | 0.323 | 0.023469427 |
| RNU6-761P | OR1K1 | 0.323 | 0.023469427 |
| RNU6-761P | FCGBP | 0.324 | 0.023048258 |
| RNU6-761P | ELMO1 | 0.325 | 0.022633669 |
| RNU6-761P | DTHD1 | 0.325 | 0.022633669 |
| RNU6-761P | NIPBL | 0.325 | 0.022633669 |
| RNU6-761P | CXCR6 | 0.326 | 0.022225576 |
| RNU6-761P | NCOA3 | 0.326 | 0.022225576 |
| RNU6-761P | OPALIN | 0.326 | 0.022225576 |
| RNU6-761P | PTAR1 | 0.327 | 0.021823897 |
| RNU6-761P | BRWD3 | 0.327 | 0.021823897 |
| RNU6-761P | NPAT | 0.328 | 0.02142855 |
| RNU6-761P | MFGE8 | 0.328 | 0.02142855 |
| RNU6-761P | MKLN1 | 0.328 | 0.02142855 |
| RNU6-761P | LPAR6 | 0.328 | 0.02142855 |
| RNU6-761P | SUN1 | 0.328 | 0.02142855 |
| RNU6-761P | GATAD2B | 0.328 | 0.02142855 |
| RNU6-761P | CTAGE6 | 0.328 | 0.02142855 |
| RNU6-761P | TMCC1 | 0.329 | 0.021039454 |
| RNU6-761P | DOCK11 | 0.329 | 0.021039454 |
| RNU6-761P | AC135178.2 | 0.329 | 0.021039454 |
| RNU6-761P | SUFU | 0.329 | 0.021039454 |
| RNU6-761P | SEC24B | 0.329 | 0.021039454 |
| RNU6-761P | LMBR1 | 0.329 | 0.021039454 |
| RNU6-761P | MB21D2 | 0.329 | 0.021039454 |
| RNU6-761P | MAST4 | 0.329 | 0.021039454 |
| RNU6-761P | ABCA12 | 0.33 | 0.020656529 |
| RNU6-761P | SAMD3 | 0.33 | 0.020656529 |
| RNU6-761P | AFF1 | 0.33 | 0.020656529 |
| RNU6-761P | STARD9 | 0.331 | 0.020279694 |
| RNU6-761P | ELK4 | 0.332 | 0.019908871 |
| RNU6-761P | FCMR | 0.332 | 0.019908871 |
| RNU6-761P | ADRA1D | 0.332 | 0.019908871 |
| RNU6-761P | C12orf42 | 0.332 | 0.019908871 |
| RNU6-761P | GPX2 | 0.333 | 0.019543982 |
| RNU6-761P | ZCCHC6 | 0.333 | 0.019543982 |
| RNU6-761P | ANKRD6 | 0.333 | 0.019543982 |
| RNU6-761P | ERCC6L2 | 0.333 | 0.019543982 |
| RNU6-761P | TMEM140 | 0.334 | 0.019184947 |
| RNU6-761P | SPANXB1 | 0.334 | 0.019184947 |
| RNU6-761P | SERPINB9 | 0.334 | 0.019184947 |
| RNU6-761P | RNF213 | 0.334 | 0.019184947 |
| RNU6-761P | TLL2 | 0.335 | 0.018831692 |
| RNU6-761P | CA13 | 0.335 | 0.018831692 |
| RNU6-761P | ZCCHC2 | 0.335 | 0.018831692 |
| RNU6-761P | LATS1 | 0.336 | 0.018484138 |
| RNU6-761P | CELF2 | 0.336 | 0.018484138 |
| RNU6-761P | LPP | 0.336 | 0.018484138 |
| RNU6-761P | TAOK1 | 0.336 | 0.018484138 |
| RNU6-761P | MCC | 0.336 | 0.018484138 |
| RNU6-761P | C3orf30 | 0.336 | 0.018484138 |
| RNU6-761P | OR2M3 | 0.337 | 0.018142212 |
| RNU6-761P | ERCC6 | 0.337 | 0.018142212 |
| RNU6-761P | GFOD1 | 0.338 | 0.017805837 |
| RNU6-761P | CNST | 0.338 | 0.017805837 |
| RNU6-761P | PHC3 | 0.338 | 0.017805837 |
| RNU6-761P | SV2C | 0.339 | 0.017474939 |
| RNU6-761P | ATP8B1 | 0.339 | 0.017474939 |
| RNU6-761P | CLASP1 | 0.34 | 0.017149445 |
| RNU6-761P | JADE2 | 0.34 | 0.017149445 |
| RNU6-761P | DIP2B | 0.34 | 0.017149445 |
| RNU6-761P | MTSS1 | 0.341 | 0.016829282 |
| RNU6-761P | ADD3 | 0.341 | 0.016829282 |
| RNU6-761P | TCN2 | 0.341 | 0.016829282 |
| RNU6-761P | XRN1 | 0.342 | 0.016514378 |
| RNU6-761P | PARP12 | 0.342 | 0.016514378 |
| RNU6-761P | IL1R2 | 0.343 | 0.016204661 |
| RNU6-761P | ST8SIA5 | 0.343 | 0.016204661 |
| RNU6-761P | DAAM2 | 0.344 | 0.015900061 |
| RNU6-761P | CASP10 | 0.344 | 0.015900061 |
| RNU6-761P | CYP1A2 | 0.345 | 0.015600506 |
| RNU6-761P | INSL5 | 0.346 | 0.015305929 |
| RNU6-761P | ARAP2 | 0.346 | 0.015305929 |
| RNU6-761P | ESR1 | 0.346 | 0.015305929 |
| RNU6-761P | BANP | 0.346 | 0.015305929 |
| RNU6-761P | ACTR3C | 0.347 | 0.01501626 |
| RNU6-761P | TCF7L2 | 0.347 | 0.01501626 |
| RNU6-761P | KIF1B | 0.347 | 0.01501626 |
| RNU6-761P | ZNF619 | 0.347 | 0.01501626 |
| RNU6-761P | WDR44 | 0.347 | 0.01501626 |
| RNU6-761P | CREG2 | 0.348 | 0.01473143 |
| RNU6-761P | PRKCH | 0.348 | 0.01473143 |
| RNU6-761P | GPATCH8 | 0.349 | 0.014451374 |
| RNU6-761P | PLCG1 | 0.349 | 0.014451374 |
| RNU6-761P | VSIG4 | 0.35 | 0.014176023 |
| RNU6-761P | MYBPC1 | 0.35 | 0.014176023 |
| RNU6-761P | C5orf56 | 0.35 | 0.014176023 |
| RNU6-761P | NRBP2 | 0.351 | 0.013905311 |
| RNU6-761P | KCNE1B | 0.351 | 0.013905311 |
| RNU6-761P | DOCK9 | 0.352 | 0.013639174 |
| RNU6-761P | PLG | 0.352 | 0.013639174 |
| RNU6-761P | DNMT3L | 0.353 | 0.013377547 |
| RNU6-761P | RASA1 | 0.355 | 0.012867565 |
| RNU6-761P | BCAS3 | 0.355 | 0.012867565 |
| RNU6-761P | RCSD1 | 0.355 | 0.012867565 |
| RNU6-761P | DYRK2 | 0.355 | 0.012867565 |
| RNU6-761P | ZBTB37 | 0.355 | 0.012867565 |
| RNU6-761P | ARNTL | 0.356 | 0.012619084 |
| RNU6-761P | CFAP58 | 0.356 | 0.012619084 |
| RNU6-761P | ERICH1 | 0.356 | 0.012619084 |
| RNU6-761P | IQGAP2 | 0.356 | 0.012619084 |
| RNU6-761P | ARPP21 | 0.357 | 0.012374861 |
| RNU6-761P | PPP3CC | 0.357 | 0.012374861 |
| RNU6-761P | IL24 | 0.357 | 0.012374861 |
| RNU6-761P | SNURF | 0.358 | 0.012134833 |
| RNU6-761P | UCP1 | 0.358 | 0.012134833 |
| RNU6-761P | INPP4B | 0.358 | 0.012134833 |
| RNU6-761P | SH2D4A | 0.358 | 0.012134833 |
| RNU6-761P | SYNRG | 0.358 | 0.012134833 |
| RNU6-761P | GLI1 | 0.359 | 0.011898941 |
| RNU6-761P | COBL | 0.359 | 0.011898941 |
| RNU6-761P | ZNF154 | 0.359 | 0.011898941 |
| RNU6-761P | UPK1B | 0.36 | 0.011667123 |
| RNU6-761P | EBI3 | 0.36 | 0.011667123 |
| RNU6-761P | ADGRF1 | 0.36 | 0.011667123 |
| RNU6-761P | KATNAL1 | 0.361 | 0.011439321 |
| RNU6-761P | HERC1 | 0.361 | 0.011439321 |
| RNU6-761P | DLEU1 | 0.361 | 0.011439321 |
| RNU6-761P | AOX1 | 0.363 | 0.01099553 |
| RNU6-761P | RHCG | 0.365 | 0.010567103 |
| RNU6-761P | SLC35E3 | 0.365 | 0.010567103 |
| RNU6-761P | VPS13D | 0.365 | 0.010567103 |
| RNU6-761P | TRHR | 0.365 | 0.010567103 |
| RNU6-761P | F2RL2 | 0.366 | 0.010358509 |
| RNU6-761P | WWP1 | 0.367 | 0.010153588 |
| RNU6-761P | PIK3IP1 | 0.368 | 0.009952284 |
| RNU6-761P | ISG20 | 0.368 | 0.009952284 |
| RNU6-761P | GPR82 | 0.37 | 0.009560311 |
| RNU6-761P | LNPEP | 0.37 | 0.009560311 |
| RNU6-761P | WASF2 | 0.37 | 0.009560311 |
| RNU6-761P | LIMK2 | 0.37 | 0.009560311 |
| RNU6-761P | RFX3 | 0.37 | 0.009560311 |
| RNU6-761P | REC114 | 0.371 | 0.009369535 |
| RNU6-761P | CTTNBP2NL | 0.371 | 0.009369535 |
| RNU6-761P | HECA | 0.372 | 0.009182163 |
| RNU6-761P | TAS2R50 | 0.372 | 0.009182163 |
| RNU6-761P | STK24 | 0.372 | 0.009182163 |
| RNU6-761P | NCOA2 | 0.373 | 0.008998142 |
| RNU6-761P | DENND4C | 0.374 | 0.008817422 |
| RNU6-761P | TAS2R46 | 0.374 | 0.008817422 |
| RNU6-761P | DLGAP2 | 0.374 | 0.008817422 |
| RNU6-761P | RNF32 | 0.374 | 0.008817422 |
| RNU6-761P | ABLIM3 | 0.375 | 0.008639952 |
| RNU6-761P | TRAF6 | 0.375 | 0.008639952 |
| RNU6-761P | CD163 | 0.375 | 0.008639952 |
| RNU6-761P | TMEM236 | 0.376 | 0.008465681 |
| RNU6-761P | TCP11L2 | 0.376 | 0.008465681 |
| RNU6-761P | SCGB3A2 | 0.376 | 0.008465681 |
| RNU6-761P | CYP1A1 | 0.377 | 0.00829456 |
| RNU6-761P | ANKUB1 | 0.378 | 0.008126541 |
| RNU6-761P | GRK5 | 0.378 | 0.008126541 |
| RNU6-761P | SMAD1 | 0.379 | 0.007961575 |
| RNU6-761P | SUMO4 | 0.379 | 0.007961575 |
| RNU6-761P | USP34 | 0.379 | 0.007961575 |
| RNU6-761P | KLHL6 | 0.38 | 0.007799614 |
| RNU6-761P | KCNRG | 0.38 | 0.007799614 |
| RNU6-761P | LRCH1 | 0.38 | 0.007799614 |
| RNU6-761P | CXorf23 | 0.38 | 0.007799614 |
| RNU6-761P | ECHDC3 | 0.38 | 0.007799614 |
| RNU6-761P | TNRC6B | 0.381 | 0.007640611 |
| RNU6-761P | DDR1 | 0.382 | 0.00748452 |
| RNU6-761P | REL | 0.383 | 0.007331294 |
| RNU6-761P | RIPOR2 | 0.383 | 0.007331294 |
| RNU6-761P | RIPPLY3 | 0.384 | 0.007180889 |
| RNU6-761P | RETREG3 | 0.384 | 0.007180889 |
| RNU6-761P | GPR34 | 0.385 | 0.007033259 |
| RNU6-761P | BICRAL | 0.385 | 0.007033259 |
| RNU6-761P | COBLL1 | 0.387 | 0.00674615 |
| RNU6-761P | SCN4B | 0.387 | 0.00674615 |
| RNU6-761P | THBS1 | 0.387 | 0.00674615 |
| RNU6-761P | FNDC9 | 0.387 | 0.00674615 |
| RNU6-761P | MAP1LC3B2 | 0.388 | 0.006606584 |
| RNU6-761P | ZBTB25 | 0.39 | 0.006335214 |
| RNU6-761P | TLDC1 | 0.39 | 0.006335214 |
| RNU6-761P | ZNF699 | 0.391 | 0.006203327 |
| RNU6-761P | SNX25 | 0.393 | 0.005946945 |
| RNU6-761P | RC3H2 | 0.393 | 0.005946945 |
| RNU6-761P | HCRTR1 | 0.394 | 0.005822369 |
| RNU6-761P | UBR2 | 0.394 | 0.005822369 |
| RNU6-761P | FBXO32 | 0.395 | 0.005700151 |
| RNU6-761P | RTL5 | 0.396 | 0.005580251 |
| RNU6-761P | GK3P | 0.396 | 0.005580251 |
| RNU6-761P | TMOD2 | 0.397 | 0.005462632 |
| RNU6-761P | NCOA6 | 0.398 | 0.005347255 |
| RNU6-761P | DDX58 | 0.399 | 0.005234083 |
| RNU6-761P | ARID5B | 0.399 | 0.005234083 |
| RNU6-761P | PIK3C2A | 0.4 | 0.00512308 |
| RNU6-761P | C5orf46 | 0.403 | 0.004802719 |
| RNU6-761P | EMP2 | 0.403 | 0.004802719 |
| RNU6-761P | GLYATL1 | 0.404 | 0.004700031 |
| RNU6-761P | CFAP221 | 0.404 | 0.004700031 |
| RNU6-761P | TMIGD3 | 0.412 | 0.003947438 |
| RNU6-761P | FMO5 | 0.412 | 0.003947438 |
| RNU6-761P | ZNF484 | 0.414 | 0.003777254 |
| RNU6-761P | SEMA4D | 0.414 | 0.003777254 |
| RNU6-761P | SSH2 | 0.416 | 0.003613763 |
| RNU6-761P | WDR20 | 0.417 | 0.003534456 |
| RNU6-761P | TNFRSF21 | 0.42 | 0.00330594 |
| RNU6-761P | LRP2BP | 0.426 | 0.002888802 |
| RNU6-761P | UTRN | 0.428 | 0.002760815 |
| RNU6-761P | CBLB | 0.433 | 0.002463183 |
| RNU6-761P | MAML3 | 0.435 | 0.002352582 |
| RNU6-761P | PDGFC | 0.435 | 0.002352582 |
| RNU6-761P | LRCH3 | 0.436 | 0.002299004 |
| RNU6-761P | ABHD6 | 0.438 | 0.002195186 |
| RNU6-761P | CLIP4 | 0.441 | 0.002047496 |
| RNU6-761P | OLAH | 0.444 | 0.001908974 |
| RNU6-761P | SLC38A11 | 0.448 | 0.001737656 |
| RNU6-761P | TAF8 | 0.451 | 0.001618573 |
| RNU6-761P | SH3PXD2B | 0.455 | 0.001471468 |
| RNU6-761P | RLN3 | 0.46 | 0.001304925 |
| RNU6-761P | PALLD | 0.463 | 0.001213536 |
| RNU6-761P | RBMS3 | 0.467 | 0.001100867 |
| RNU6-761P | FKBP5 | 0.471 | 0.000997941 |
| RNU6-761P | C14orf105 | 0.476 | 0.000881815 |
| RNU6-761P | SESN1 | 0.485 | 0.000703776 |
| RNU6-761P | AKAP5 | 0.491 | 0.000604306 |
| RNU6-761P | MEGF11 | 0.499 | 0.000491951 |
| RNU6-761P | USP6NL | 0.514 | 0.000331914 |
| RNU6-761P | ALK | 0.528 | 0.000227784 |
| RNU6-761P | RNU6-761P | 1 | 3.50E-12 |
| RNU6-202P | AIFM1 | -0.475 | 0.001227287 |
| RNU6-202P | ECHS1 | -0.457 | 0.001864003 |
| RNU6-202P | NDUFS2 | -0.453 | 0.002041592 |
| RNU6-202P | XRCC6 | -0.445 | 0.002444162 |
| RNU6-202P | ATP5A1 | -0.441 | 0.002671589 |
| RNU6-202P | ATP5B | -0.44 | 0.002731389 |
| RNU6-202P | ATP5C1 | -0.436 | 0.002983019 |
| RNU6-202P | STOML2 | -0.435 | 0.003049147 |
| RNU6-202P | CCT7 | -0.435 | 0.003049147 |
| RNU6-202P | APEH | -0.428 | 0.003550759 |
| RNU6-202P | ZCCHC17 | -0.427 | 0.003628248 |
| RNU6-202P | GOT2 | -0.427 | 0.003628248 |
| RNU6-202P | IMMT | -0.426 | 0.003707272 |
| RNU6-202P | TIMMDC1 | -0.425 | 0.003787857 |
| RNU6-202P | CCDC51 | -0.421 | 0.004126363 |
| RNU6-202P | PPP1R7 | -0.419 | 0.004305709 |
| RNU6-202P | PRPF4 | -0.417 | 0.004492095 |
| RNU6-202P | COQ9 | -0.415 | 0.004685762 |
| RNU6-202P | CCT3 | -0.414 | 0.004785403 |
| RNU6-202P | CINP | -0.414 | 0.004785403 |
| RNU6-202P | C1orf123 | -0.413 | 0.004886957 |
| RNU6-202P | DNAJC8 | -0.413 | 0.004886957 |
| RNU6-202P | FH | -0.413 | 0.004886957 |
| RNU6-202P | EIF3I | -0.404 | 0.005892491 |
| RNU6-202P | MRM3 | -0.403 | 0.006015017 |
| RNU6-202P | MRPL44 | -0.398 | 0.006662755 |
| RNU6-202P | MRPL37 | -0.398 | 0.006662755 |
| RNU6-202P | HNRNPF | -0.398 | 0.006662755 |
| RNU6-202P | AHCY | -0.397 | 0.00679959 |
| RNU6-202P | CPT2 | -0.395 | 0.007080861 |
| RNU6-202P | FDFT1 | -0.394 | 0.007225377 |
| RNU6-202P | RWDD2B | -0.393 | 0.007372535 |
| RNU6-202P | PSMD4 | -0.393 | 0.007372535 |
| RNU6-202P | RTCB | -0.392 | 0.007522376 |
| RNU6-202P | USP5 | -0.391 | 0.007674942 |
| RNU6-202P | NDUFA8 | -0.391 | 0.007674942 |
| RNU6-202P | ADI1 | -0.39 | 0.007830275 |
| RNU6-202P | TUFM | -0.389 | 0.007988419 |
| RNU6-202P | TMEM203 | -0.389 | 0.007988419 |
| RNU6-202P | UQCRC2 | -0.388 | 0.008149417 |
| RNU6-202P | NUDC | -0.386 | 0.008480151 |
| RNU6-202P | SUCLG1 | -0.386 | 0.008480151 |
| RNU6-202P | NUP93 | -0.385 | 0.008649978 |
| RNU6-202P | DRG1 | -0.383 | 0.008998777 |
| RNU6-202P | NSDHL | -0.383 | 0.008998777 |
| RNU6-202P | PARK7 | -0.383 | 0.008998777 |
| RNU6-202P | MRPS18B | -0.381 | 0.009360083 |
| RNU6-202P | AHSA1 | -0.381 | 0.009360083 |
| RNU6-202P | MRM2 | -0.378 | 0.009926324 |
| RNU6-202P | POLDIP2 | -0.378 | 0.009926324 |
| RNU6-202P | KARS | -0.378 | 0.009926324 |
| RNU6-202P | TOMM34 | -0.377 | 0.010121742 |
| RNU6-202P | RRP36 | -0.376 | 0.010320578 |
| RNU6-202P | CBR1 | -0.376 | 0.010320578 |
| RNU6-202P | OGFOD1 | -0.374 | 0.010728707 |
| RNU6-202P | SLC29A1 | -0.374 | 0.010728707 |
| RNU6-202P | GOT1 | -0.372 | 0.011151124 |
| RNU6-202P | ALG1 | -0.372 | 0.011151124 |
| RNU6-202P | PPM1G | -0.372 | 0.011151124 |
| RNU6-202P | SRP68 | -0.372 | 0.011151124 |
| RNU6-202P | NSMCE1 | -0.371 | 0.011367821 |
| RNU6-202P | HSPA8 | -0.37 | 0.011588249 |
| RNU6-202P | GSS | -0.37 | 0.011588249 |
| RNU6-202P | CIAPIN1 | -0.37 | 0.011588249 |
| RNU6-202P | POP4 | -0.37 | 0.011588249 |
| RNU6-202P | NETO2 | -0.369 | 0.01181246 |
| RNU6-202P | MRPS15 | -0.367 | 0.012272456 |
| RNU6-202P | ASB13 | -0.365 | 0.012748252 |
| RNU6-202P | BST2 | -0.364 | 0.012992216 |
| RNU6-202P | MTFR1L | -0.364 | 0.012992216 |
| RNU6-202P | PSMB5 | -0.364 | 0.012992216 |
| RNU6-202P | RPL4 | -0.362 | 0.013492565 |
| RNU6-202P | VDAC3 | -0.362 | 0.013492565 |
| RNU6-202P | PLA2G4A | -0.361 | 0.013749066 |
| RNU6-202P | DHRS4 | -0.361 | 0.013749066 |
| RNU6-202P | PHB | -0.361 | 0.013749066 |
| RNU6-202P | MRPL15 | -0.361 | 0.013749066 |
| RNU6-202P | MRPS16 | -0.361 | 0.013749066 |
| RNU6-202P | SEPHS2 | -0.36 | 0.014009864 |
| RNU6-202P | C16orf62 | -0.359 | 0.014275019 |
| RNU6-202P | TTC1 | -0.359 | 0.014275019 |
| RNU6-202P | PRDX3 | -0.359 | 0.014275019 |
| RNU6-202P | PCYOX1L | -0.359 | 0.014275019 |
| RNU6-202P | MRPL35 | -0.359 | 0.014275019 |
| RNU6-202P | TACO1 | -0.358 | 0.014544591 |
| RNU6-202P | GLOD4 | -0.358 | 0.014544591 |
| RNU6-202P | RPA1 | -0.357 | 0.014818642 |
| RNU6-202P | PLPBP | -0.357 | 0.014818642 |
| RNU6-202P | MRPS7 | -0.355 | 0.015380426 |
| RNU6-202P | UQCRC1 | -0.355 | 0.015380426 |
| RNU6-202P | UTP14A | -0.354 | 0.015668285 |
| RNU6-202P | CHCHD4 | -0.354 | 0.015668285 |
| RNU6-202P | ATP5F1 | -0.354 | 0.015668285 |
| RNU6-202P | PSMA5 | -0.354 | 0.015668285 |
| RNU6-202P | GTSF1 | -0.353 | 0.015960873 |
| RNU6-202P | PRMT5 | -0.352 | 0.016258255 |
| RNU6-202P | PPIL1 | -0.352 | 0.016258255 |
| RNU6-202P | AIMP2 | -0.352 | 0.016258255 |
| RNU6-202P | PES1 | -0.351 | 0.016560494 |
| RNU6-202P | ELAC2 | -0.35 | 0.016867657 |
| RNU6-202P | UNC45A | -0.35 | 0.016867657 |
| RNU6-202P | CPSF3 | -0.349 | 0.017179809 |
| RNU6-202P | CSTF2 | -0.349 | 0.017179809 |
| RNU6-202P | SERPINB6 | -0.348 | 0.017497017 |
| RNU6-202P | NHLRC1 | -0.347 | 0.017819348 |
| RNU6-202P | SLC50A1 | -0.346 | 0.018146871 |
| RNU6-202P | KLHL12 | -0.346 | 0.018146871 |
| RNU6-202P | DOLK | -0.342 | 0.01951025 |
| RNU6-202P | PYM1 | -0.342 | 0.01951025 |
| RNU6-202P | MDH1 | -0.342 | 0.01951025 |
| RNU6-202P | ATIC | -0.341 | 0.019864768 |
| RNU6-202P | SARS | -0.341 | 0.019864768 |
| RNU6-202P | SFXN4 | -0.341 | 0.019864768 |
| RNU6-202P | ZNF185 | -0.341 | 0.019864768 |
| RNU6-202P | HADHA | -0.341 | 0.019864768 |
| RNU6-202P | FAM162A | -0.34 | 0.020224897 |
| RNU6-202P | EEF2KMT | -0.339 | 0.020590709 |
| RNU6-202P | EBNA1BP2 | -0.339 | 0.020590709 |
| RNU6-202P | SDHB | -0.339 | 0.020590709 |
| RNU6-202P | PCBD1 | -0.339 | 0.020590709 |
| RNU6-202P | DAP3 | -0.338 | 0.020962277 |
| RNU6-202P | FIBP | -0.338 | 0.020962277 |
| RNU6-202P | ECH1 | -0.338 | 0.020962277 |
| RNU6-202P | PDHA1 | -0.337 | 0.021339675 |
| RNU6-202P | NFS1 | -0.337 | 0.021339675 |
| RNU6-202P | TPI1 | -0.337 | 0.021339675 |
| RNU6-202P | ERCC2 | -0.336 | 0.021722976 |
| RNU6-202P | C1orf131 | -0.336 | 0.021722976 |
| RNU6-202P | STIP1 | -0.335 | 0.022112256 |
| RNU6-202P | SAMM50 | -0.334 | 0.022507589 |
| RNU6-202P | TXN2 | -0.334 | 0.022507589 |
| RNU6-202P | MRPS10 | -0.334 | 0.022507589 |
| RNU6-202P | SNRNP40 | -0.334 | 0.022507589 |
| RNU6-202P | RTFDC1 | -0.334 | 0.022507589 |
| RNU6-202P | COA7 | -0.333 | 0.022909052 |
| RNU6-202P | CASP9 | -0.333 | 0.022909052 |
| RNU6-202P | QARS | -0.333 | 0.022909052 |
| RNU6-202P | GLA | -0.331 | 0.023730673 |
| RNU6-202P | RILP | -0.331 | 0.023730673 |
| RNU6-202P | GPN1 | -0.331 | 0.023730673 |
| RNU6-202P | CYB5R1 | -0.33 | 0.024150985 |
| RNU6-202P | LDLRAD2 | -0.33 | 0.024150985 |
| RNU6-202P | NDUFB10 | -0.33 | 0.024150985 |
| RNU6-202P | CCT5 | -0.33 | 0.024150985 |
| RNU6-202P | TSSC1 | -0.329 | 0.024577737 |
| RNU6-202P | ALDH1B1 | -0.329 | 0.024577737 |
| RNU6-202P | FAHD1 | -0.329 | 0.024577737 |
| RNU6-202P | ADSL | -0.329 | 0.024577737 |
| RNU6-202P | MPI | -0.329 | 0.024577737 |
| RNU6-202P | SLC25A5 | -0.329 | 0.024577737 |
| RNU6-202P | HSD17B10 | -0.328 | 0.025011006 |
| RNU6-202P | APEX1 | -0.328 | 0.025011006 |
| RNU6-202P | ZNF691 | -0.328 | 0.025011006 |
| RNU6-202P | MDH2 | -0.327 | 0.025450874 |
| RNU6-202P | LGALS3BP | -0.327 | 0.025450874 |
| RNU6-202P | MRPS9 | -0.327 | 0.025450874 |
| RNU6-202P | HDDC3 | -0.326 | 0.025897419 |
| RNU6-202P | SELENON | -0.326 | 0.025897419 |
| RNU6-202P | TRAP1 | -0.326 | 0.025897419 |
| RNU6-202P | MED8 | -0.326 | 0.025897419 |
| RNU6-202P | RNF25 | -0.325 | 0.026350722 |
| RNU6-202P | CCT8 | -0.325 | 0.026350722 |
| RNU6-202P | CDC123 | -0.325 | 0.026350722 |
| RNU6-202P | TARS2 | -0.324 | 0.026810865 |
| RNU6-202P | NANS | -0.324 | 0.026810865 |
| RNU6-202P | ZNF622 | -0.323 | 0.027277931 |
| RNU6-202P | MED20 | -0.323 | 0.027277931 |
| RNU6-202P | CHCHD10 | -0.323 | 0.027277931 |
| RNU6-202P | DPP3 | -0.323 | 0.027277931 |
| RNU6-202P | H2AFY2 | -0.323 | 0.027277931 |
| RNU6-202P | MRPL58 | -0.322 | 0.027752001 |
| RNU6-202P | OXSM | -0.322 | 0.027752001 |
| RNU6-202P | HDAC3 | -0.321 | 0.028233158 |
| RNU6-202P | TBCE | -0.321 | 0.028233158 |
| RNU6-202P | PAK1IP1 | -0.321 | 0.028233158 |
| RNU6-202P | SNX17 | -0.32 | 0.028721487 |
| RNU6-202P | SIL1 | -0.32 | 0.028721487 |
| RNU6-202P | RUVBL1 | -0.32 | 0.028721487 |
| RNU6-202P | PDHB | -0.32 | 0.028721487 |
| RNU6-202P | NUP37 | -0.319 | 0.029217071 |
| RNU6-202P | DDX28 | -0.319 | 0.029217071 |
| RNU6-202P | GHITM | -0.319 | 0.029217071 |
| RNU6-202P | APOBEC3C | -0.319 | 0.029217071 |
| RNU6-202P | QDPR | -0.318 | 0.029719996 |
| RNU6-202P | DPAGT1 | -0.318 | 0.029719996 |
| RNU6-202P | MYCBP | -0.318 | 0.029719996 |
| RNU6-202P | MRPL18 | -0.317 | 0.030230347 |
| RNU6-202P | RPA2 | -0.317 | 0.030230347 |
| RNU6-202P | RPLP0 | -0.317 | 0.030230347 |
| RNU6-202P | PRKAG1 | -0.317 | 0.030230347 |
| RNU6-202P | FAH | -0.316 | 0.030748211 |
| RNU6-202P | APTX | -0.316 | 0.030748211 |
| RNU6-202P | MTFP1 | -0.316 | 0.030748211 |
| RNU6-202P | IDH1 | -0.316 | 0.030748211 |
| RNU6-202P | SMIM15 | -0.315 | 0.031273673 |
| RNU6-202P | APEX2 | -0.315 | 0.031273673 |
| RNU6-202P | PMVK | -0.315 | 0.031273673 |
| RNU6-202P | GTF2H3 | -0.315 | 0.031273673 |
| RNU6-202P | MSRB2 | -0.315 | 0.031273673 |
| RNU6-202P | NDUFV3 | -0.314 | 0.031806822 |
| RNU6-202P | ATP6V1E1 | -0.314 | 0.031806822 |
| RNU6-202P | RAN | -0.314 | 0.031806822 |
| RNU6-202P | MRTO4 | -0.314 | 0.031806822 |
| RNU6-202P | AP2M1 | -0.313 | 0.032347744 |
| RNU6-202P | ATP1B3 | -0.312 | 0.03289653 |
| RNU6-202P | EHHADH | -0.312 | 0.03289653 |
| RNU6-202P | THYN1 | -0.312 | 0.03289653 |
| RNU6-202P | LYAR | -0.312 | 0.03289653 |
| RNU6-202P | ACO2 | -0.312 | 0.03289653 |
| RNU6-202P | MRPS18A | -0.312 | 0.03289653 |
| RNU6-202P | TCTN3 | -0.312 | 0.03289653 |
| RNU6-202P | CNBP | -0.311 | 0.033453266 |
| RNU6-202P | NELFE | -0.311 | 0.033453266 |
| RNU6-202P | IFI27L2 | -0.31 | 0.034018044 |
| RNU6-202P | MUL1 | -0.31 | 0.034018044 |
| RNU6-202P | RNASEH2A | -0.31 | 0.034018044 |
| RNU6-202P | MRPL13 | -0.31 | 0.034018044 |
| RNU6-202P | HINT2 | -0.31 | 0.034018044 |
| RNU6-202P | PARP1 | -0.31 | 0.034018044 |
| RNU6-202P | L3MBTL2 | -0.31 | 0.034018044 |
| RNU6-202P | RALBP1 | -0.309 | 0.034590953 |
| RNU6-202P | KBTBD8 | -0.309 | 0.034590953 |
| RNU6-202P | LPCAT3 | -0.309 | 0.034590953 |
| RNU6-202P | SRPRB | -0.308 | 0.035172083 |
| RNU6-202P | MRPS23 | -0.308 | 0.035172083 |
| RNU6-202P | HAUS4 | -0.308 | 0.035172083 |
| RNU6-202P | BLMH | -0.308 | 0.035172083 |
| RNU6-202P | MVK | -0.308 | 0.035172083 |
| RNU6-202P | IMP3 | -0.308 | 0.035172083 |
| RNU6-202P | ATPAF1 | -0.307 | 0.035761526 |
| RNU6-202P | BBS12 | -0.307 | 0.035761526 |
| RNU6-202P | FDPS | -0.307 | 0.035761526 |
| RNU6-202P | RGS10 | -0.306 | 0.036359374 |
| RNU6-202P | HIRA | -0.306 | 0.036359374 |
| RNU6-202P | RNASEH1 | -0.306 | 0.036359374 |
| RNU6-202P | BCKDK | -0.306 | 0.036359374 |
| RNU6-202P | SSRP1 | -0.306 | 0.036359374 |
| RNU6-202P | USP39 | -0.305 | 0.036965719 |
| RNU6-202P | PEF1 | -0.305 | 0.036965719 |
| RNU6-202P | DOK1 | -0.304 | 0.037580653 |
| RNU6-202P | PSMD13 | -0.304 | 0.037580653 |
| RNU6-202P | RRS1 | -0.304 | 0.037580653 |
| RNU6-202P | TDP2 | -0.304 | 0.037580653 |
| RNU6-202P | PRDX6 | -0.304 | 0.037580653 |
| RNU6-202P | PABPC1L | -0.303 | 0.038204271 |
| RNU6-202P | PSMC5 | -0.303 | 0.038204271 |
| RNU6-202P | LRRC42 | -0.303 | 0.038204271 |
| RNU6-202P | AKR7A2 | -0.303 | 0.038204271 |
| RNU6-202P | SAE1 | -0.303 | 0.038204271 |
| RNU6-202P | RPL6 | -0.303 | 0.038204271 |
| RNU6-202P | NABP2 | -0.303 | 0.038204271 |
| RNU6-202P | TOMM40L | -0.303 | 0.038204271 |
| RNU6-202P | RNF41 | -0.303 | 0.038204271 |
| RNU6-202P | IPO13 | -0.303 | 0.038204271 |
| RNU6-202P | HDLBP | -0.303 | 0.038204271 |
| RNU6-202P | C1orf54 | -0.303 | 0.038204271 |
| RNU6-202P | HSP90AB1 | -0.303 | 0.038204271 |
| RNU6-202P | CCT4 | -0.302 | 0.038836665 |
| RNU6-202P | VCP | -0.302 | 0.038836665 |
| RNU6-202P | GART | -0.302 | 0.038836665 |
| RNU6-202P | S100A13 | -0.302 | 0.038836665 |
| RNU6-202P | SHMT2 | -0.302 | 0.038836665 |
| RNU6-202P | ENO1 | -0.302 | 0.038836665 |
| RNU6-202P | ATP5O | -0.302 | 0.038836665 |
| RNU6-202P | CSRP1 | -0.302 | 0.038836665 |
| RNU6-202P | IMPDH2 | -0.302 | 0.038836665 |
| RNU6-202P | COQ5 | -0.301 | 0.03947793 |
| RNU6-202P | MRPL24 | -0.301 | 0.03947793 |
| RNU6-202P | BCS1L | -0.301 | 0.03947793 |
| RNU6-202P | ACADS | -0.3 | 0.040128162 |
| RNU6-202P | TMEM69 | -0.3 | 0.040128162 |
| RNU6-202P | ACAT2 | -0.299 | 0.040787455 |
| RNU6-202P | HTATSF1 | -0.299 | 0.040787455 |
| RNU6-202P | CCRL2 | -0.299 | 0.040787455 |
| RNU6-202P | BSPRY | -0.299 | 0.040787455 |
| RNU6-202P | PSMD7 | -0.299 | 0.040787455 |
| RNU6-202P | METTL13 | -0.299 | 0.040787455 |
| RNU6-202P | TMEM186 | -0.299 | 0.040787455 |
| RNU6-202P | GRPEL1 | -0.299 | 0.040787455 |
| RNU6-202P | MFAP1 | -0.299 | 0.040787455 |
| RNU6-202P | RNF113A | -0.298 | 0.041455905 |
| RNU6-202P | DDX1 | -0.298 | 0.041455905 |
| RNU6-202P | PPCS | -0.298 | 0.041455905 |
| RNU6-202P | CACYBP | -0.298 | 0.041455905 |
| RNU6-202P | ISOC2 | -0.298 | 0.041455905 |
| RNU6-202P | TBRG4 | -0.297 | 0.04213361 |
| RNU6-202P | DNTTIP1 | -0.297 | 0.04213361 |
| RNU6-202P | BCAP31 | -0.297 | 0.04213361 |
| RNU6-202P | PSMD1 | -0.297 | 0.04213361 |
| RNU6-202P | LSM10 | -0.297 | 0.04213361 |
| RNU6-202P | MRPL40 | -0.297 | 0.04213361 |
| RNU6-202P | RNF8 | -0.296 | 0.042820664 |
| RNU6-202P | ELAC1 | -0.296 | 0.042820664 |
| RNU6-202P | SDHA | -0.296 | 0.042820664 |
| RNU6-202P | YWHAE | -0.295 | 0.043517167 |
| RNU6-202P | PYCR2 | -0.295 | 0.043517167 |
| RNU6-202P | DAXX | -0.295 | 0.043517167 |
| RNU6-202P | TRAF7 | -0.295 | 0.043517167 |
| RNU6-202P | ECE2 | -0.295 | 0.043517167 |
| RNU6-202P | C1QBP | -0.294 | 0.044223216 |
| RNU6-202P | KCTD10 | -0.294 | 0.044223216 |
| RNU6-202P | MRPS5 | -0.294 | 0.044223216 |
| RNU6-202P | LDHB | -0.294 | 0.044223216 |
| RNU6-202P | CKM | -0.294 | 0.044223216 |
| RNU6-202P | EIF6 | -0.294 | 0.044223216 |
| RNU6-202P | VDAC1 | -0.294 | 0.044223216 |
| RNU6-202P | ALDH16A1 | -0.294 | 0.044223216 |
| RNU6-202P | SF3A3 | -0.293 | 0.044938908 |
| RNU6-202P | UTF1 | -0.293 | 0.044938908 |
| RNU6-202P | ETFB | -0.293 | 0.044938908 |
| RNU6-202P | KXD1 | -0.293 | 0.044938908 |
| RNU6-202P | NDUFB9 | -0.292 | 0.045664344 |
| RNU6-202P | PSMD2 | -0.292 | 0.045664344 |
| RNU6-202P | GDI2 | -0.292 | 0.045664344 |
| RNU6-202P | AFG3L2 | -0.292 | 0.045664344 |
| RNU6-202P | AFMID | -0.292 | 0.045664344 |
| RNU6-202P | EIF3K | -0.291 | 0.046399621 |
| RNU6-202P | COPS7A | -0.291 | 0.046399621 |
| RNU6-202P | ZFYVE19 | -0.291 | 0.046399621 |
| RNU6-202P | PSMC4 | -0.291 | 0.046399621 |
| RNU6-202P | PCTP | -0.291 | 0.046399621 |
| RNU6-202P | EBP | -0.291 | 0.046399621 |
| RNU6-202P | MRPS34 | -0.291 | 0.046399621 |
| RNU6-202P | FAM83G | -0.29 | 0.04714484 |
| RNU6-202P | TSTA3 | -0.29 | 0.04714484 |
| RNU6-202P | AGMAT | -0.29 | 0.04714484 |
| RNU6-202P | CCNE1 | -0.29 | 0.04714484 |
| RNU6-202P | HPS1 | -0.289 | 0.047900101 |
| RNU6-202P | ARL2 | -0.289 | 0.047900101 |
| RNU6-202P | COG4 | -0.289 | 0.047900101 |
| RNU6-202P | ERAL1 | -0.289 | 0.047900101 |
| RNU6-202P | STARD7 | -0.289 | 0.047900101 |
| RNU6-202P | CHEK2 | -0.289 | 0.047900101 |
| RNU6-202P | RPL3 | -0.288 | 0.048665504 |
| RNU6-202P | KAT14 | -0.288 | 0.048665504 |
| RNU6-202P | HCCS | -0.288 | 0.048665504 |
| RNU6-202P | NUDT12 | -0.288 | 0.048665504 |
| RNU6-202P | STAC3 | -0.288 | 0.048665504 |
| RNU6-202P | PSMC3 | -0.287 | 0.04944115 |
| RNU6-202P | PPID | -0.287 | 0.04944115 |
| RNU6-202P | PSMA7 | -0.287 | 0.04944115 |
| RNU6-202P | TAF9 | -0.287 | 0.04944115 |
| RNU6-202P | RPS8 | -0.287 | 0.04944115 |
| RNU6-202P | XPC | -0.287 | 0.04944115 |
| RNU6-202P | PRDM16 | -0.287 | 0.04944115 |
| RNU6-202P | ACOT4 | -0.287 | 0.04944115 |
| RNU6-202P | ZNF18 | -0.287 | 0.04944115 |
| RNU6-202P | RORA | 0.296 | 0.049248706 |
| RNU6-202P | ZBTB25 | 0.296 | 0.049248706 |
| RNU6-202P | RNF169 | 0.296 | 0.049248706 |
| RNU6-202P | AP002495.1 | 0.297 | 0.048475598 |
| RNU6-202P | BCAS4 | 0.297 | 0.048475598 |
| RNU6-202P | ESYT3 | 0.298 | 0.047712708 |
| RNU6-202P | WWP1 | 0.298 | 0.047712708 |
| RNU6-202P | CYP26C1 | 0.298 | 0.047712708 |
| RNU6-202P | GSK3B | 0.298 | 0.047712708 |
| RNU6-202P | HOXC11 | 0.298 | 0.047712708 |
| RNU6-202P | PPP2R3A | 0.298 | 0.047712708 |
| RNU6-202P | TLK1 | 0.298 | 0.047712708 |
| RNU6-202P | SPSB1 | 0.298 | 0.047712708 |
| RNU6-202P | NR2C2 | 0.298 | 0.047712708 |
| RNU6-202P | TTC21B | 0.299 | 0.046959936 |
| RNU6-202P | LRCOL1 | 0.299 | 0.046959936 |
| RNU6-202P | CBLB | 0.299 | 0.046959936 |
| RNU6-202P | OTX1 | 0.299 | 0.046959936 |
| RNU6-202P | WNT3A | 0.299 | 0.046959936 |
| RNU6-202P | DLG1 | 0.3 | 0.04621718 |
| RNU6-202P | NEFM | 0.3 | 0.04621718 |
| RNU6-202P | ZNF81 | 0.3 | 0.04621718 |
| RNU6-202P | LRRC28 | 0.3 | 0.04621718 |
| RNU6-202P | FOXP1 | 0.3 | 0.04621718 |
| RNU6-202P | RDH16 | 0.301 | 0.045484342 |
| RNU6-202P | LRRC4 | 0.301 | 0.045484342 |
| RNU6-202P | BAZ2B | 0.301 | 0.045484342 |
| RNU6-202P | TCAF2 | 0.301 | 0.045484342 |
| RNU6-202P | CHST9 | 0.301 | 0.045484342 |
| RNU6-202P | CD160 | 0.301 | 0.045484342 |
| RNU6-202P | ZNF227 | 0.301 | 0.045484342 |
| RNU6-202P | C14orf105 | 0.302 | 0.044761321 |
| RNU6-202P | SUGT1 | 0.302 | 0.044761321 |
| RNU6-202P | AP001931.1 | 0.302 | 0.044761321 |
| RNU6-202P | FABP6 | 0.302 | 0.044761321 |
| RNU6-202P | TOX | 0.303 | 0.044048018 |
| RNU6-202P | SLC35G3 | 0.303 | 0.044048018 |
| RNU6-202P | HIVEP2 | 0.303 | 0.044048018 |
| RNU6-202P | ZNF805 | 0.303 | 0.044048018 |
| RNU6-202P | SMG6 | 0.303 | 0.044048018 |
| RNU6-202P | BOD1L1 | 0.303 | 0.044048018 |
| RNU6-202P | RUNDC3B | 0.303 | 0.044048018 |
| RNU6-202P | OLFM2 | 0.304 | 0.043344335 |
| RNU6-202P | AWAT1 | 0.304 | 0.043344335 |
| RNU6-202P | GPX2 | 0.304 | 0.043344335 |
| RNU6-202P | TACR1 | 0.304 | 0.043344335 |
| RNU6-202P | KCNS2 | 0.304 | 0.043344335 |
| RNU6-202P | PHGR1 | 0.304 | 0.043344335 |
| RNU6-202P | IGFL4 | 0.304 | 0.043344335 |
| RNU6-202P | VTN | 0.304 | 0.043344335 |
| RNU6-202P | GNPDA2 | 0.305 | 0.042650174 |
| RNU6-202P | PIBF1 | 0.305 | 0.042650174 |
| RNU6-202P | KDELR3 | 0.305 | 0.042650174 |
| RNU6-202P | ZNF43 | 0.305 | 0.042650174 |
| RNU6-202P | LRRC37A2 | 0.305 | 0.042650174 |
| RNU6-202P | ALMS1 | 0.305 | 0.042650174 |
| RNU6-202P | RNF115 | 0.305 | 0.042650174 |
| RNU6-202P | FRY | 0.305 | 0.042650174 |
| RNU6-202P | FAM228A | 0.305 | 0.042650174 |
| RNU6-202P | KIAA1328 | 0.306 | 0.041965437 |
| RNU6-202P | TNRC6C | 0.306 | 0.041965437 |
| RNU6-202P | ODF2L | 0.306 | 0.041965437 |
| RNU6-202P | KIAA2026 | 0.306 | 0.041965437 |
| RNU6-202P | SYT14 | 0.306 | 0.041965437 |
| RNU6-202P | CEPT1 | 0.306 | 0.041965437 |
| RNU6-202P | SNURF | 0.307 | 0.041290026 |
| RNU6-202P | SH3RF1 | 0.307 | 0.041290026 |
| RNU6-202P | RGPD1 | 0.307 | 0.041290026 |
| RNU6-202P | PBLD | 0.307 | 0.041290026 |
| RNU6-202P | MLLT11 | 0.307 | 0.041290026 |
| RNU6-202P | SP7 | 0.307 | 0.041290026 |
| RNU6-202P | THADA | 0.307 | 0.041290026 |
| RNU6-202P | VEGFD | 0.308 | 0.040623845 |
| RNU6-202P | SOCS7 | 0.308 | 0.040623845 |
| RNU6-202P | ASCL4 | 0.308 | 0.040623845 |
| RNU6-202P | RASGRP1 | 0.308 | 0.040623845 |
| RNU6-202P | FAM217A | 0.308 | 0.040623845 |
| RNU6-202P | VWC2L | 0.308 | 0.040623845 |
| RNU6-202P | SEBOX | 0.309 | 0.039966798 |
| RNU6-202P | EMX1 | 0.309 | 0.039966798 |
| RNU6-202P | CA5A | 0.309 | 0.039966798 |
| RNU6-202P | ZNF853 | 0.309 | 0.039966798 |
| RNU6-202P | DRC3 | 0.309 | 0.039966798 |
| RNU6-202P | CDK17 | 0.309 | 0.039966798 |
| RNU6-202P | DPF1 | 0.309 | 0.039966798 |
| RNU6-202P | GLYATL2 | 0.309 | 0.039966798 |
| RNU6-202P | PDX1 | 0.309 | 0.039966798 |
| RNU6-202P | SMIM18 | 0.309 | 0.039966798 |
| RNU6-202P | TRIM50 | 0.31 | 0.039318789 |
| RNU6-202P | SMIM11B | 0.31 | 0.039318789 |
| RNU6-202P | PXYLP1 | 0.31 | 0.039318789 |
| RNU6-202P | ANKRD20A4 | 0.31 | 0.039318789 |
| RNU6-202P | LMTK2 | 0.311 | 0.038679722 |
| RNU6-202P | MSX1 | 0.311 | 0.038679722 |
| RNU6-202P | CHD6 | 0.311 | 0.038679722 |
| RNU6-202P | ARHGEF7 | 0.311 | 0.038679722 |
| RNU6-202P | EPC1 | 0.311 | 0.038679722 |
| RNU6-202P | SETD5 | 0.312 | 0.038049504 |
| RNU6-202P | RSPH4A | 0.312 | 0.038049504 |
| RNU6-202P | FOXJ3 | 0.312 | 0.038049504 |
| RNU6-202P | SHC3 | 0.312 | 0.038049504 |
| RNU6-202P | ERO1B | 0.313 | 0.037428039 |
| RNU6-202P | MCM9 | 0.313 | 0.037428039 |
| RNU6-202P | SIAH1 | 0.313 | 0.037428039 |
| RNU6-202P | VASH2 | 0.313 | 0.037428039 |
| RNU6-202P | SEC24B | 0.313 | 0.037428039 |
| RNU6-202P | SOX13 | 0.313 | 0.037428039 |
| RNU6-202P | LRIG2 | 0.314 | 0.036815234 |
| RNU6-202P | KIAA1217 | 0.314 | 0.036815234 |
| RNU6-202P | N4BP2L2 | 0.314 | 0.036815234 |
| RNU6-202P | KDM3A | 0.314 | 0.036815234 |
| RNU6-202P | ZNF708 | 0.314 | 0.036815234 |
| RNU6-202P | GNRHR | 0.315 | 0.036210995 |
| RNU6-202P | PNMA2 | 0.315 | 0.036210995 |
| RNU6-202P | OVOL3 | 0.315 | 0.036210995 |
| RNU6-202P | PCDH7 | 0.316 | 0.035615231 |
| RNU6-202P | CCDC82 | 0.316 | 0.035615231 |
| RNU6-202P | C16orf71 | 0.316 | 0.035615231 |
| RNU6-202P | NR1D2 | 0.316 | 0.035615231 |
| RNU6-202P | TMTC4 | 0.316 | 0.035615231 |
| RNU6-202P | PLEKHA3 | 0.316 | 0.035615231 |
| RNU6-202P | NPIPB9 | 0.316 | 0.035615231 |
| RNU6-202P | PKN2 | 0.316 | 0.035615231 |
| RNU6-202P | ATP8A1 | 0.316 | 0.035615231 |
| RNU6-202P | GREB1L | 0.316 | 0.035615231 |
| RNU6-202P | CDH12 | 0.316 | 0.035615231 |
| RNU6-202P | RAB3C | 0.316 | 0.035615231 |
| RNU6-202P | ZFYVE16 | 0.316 | 0.035615231 |
| RNU6-202P | ZNF398 | 0.317 | 0.035027848 |
| RNU6-202P | MAPKAPK5 | 0.317 | 0.035027848 |
| RNU6-202P | CHIC2 | 0.317 | 0.035027848 |
| RNU6-202P | PLG | 0.317 | 0.035027848 |
| RNU6-202P | ZNF207 | 0.317 | 0.035027848 |
| RNU6-202P | MTMR7 | 0.317 | 0.035027848 |
| RNU6-202P | ASPN | 0.317 | 0.035027848 |
| RNU6-202P | CCDC141 | 0.317 | 0.035027848 |
| RNU6-202P | TAS2R4 | 0.317 | 0.035027848 |
| RNU6-202P | FBXO3 | 0.317 | 0.035027848 |
| RNU6-202P | EEPD1 | 0.317 | 0.035027848 |
| RNU6-202P | DGAT2L6 | 0.317 | 0.035027848 |
| RNU6-202P | HGFAC | 0.317 | 0.035027848 |
| RNU6-202P | ELK4 | 0.318 | 0.034448756 |
| RNU6-202P | PIK3C2A | 0.318 | 0.034448756 |
| RNU6-202P | NEMP2 | 0.318 | 0.034448756 |
| RNU6-202P | EYS | 0.318 | 0.034448756 |
| RNU6-202P | RUFY3 | 0.319 | 0.033877863 |
| RNU6-202P | C6orf201 | 0.319 | 0.033877863 |
| RNU6-202P | SLC22A12 | 0.319 | 0.033877863 |
| RNU6-202P | KDM6A | 0.319 | 0.033877863 |
| RNU6-202P | NFATC3 | 0.32 | 0.033315079 |
| RNU6-202P | METTL21C | 0.32 | 0.033315079 |
| RNU6-202P | SNX13 | 0.32 | 0.033315079 |
| RNU6-202P | SCN8A | 0.32 | 0.033315079 |
| RNU6-202P | MGAT5B | 0.321 | 0.032760313 |
| RNU6-202P | ADH6 | 0.321 | 0.032760313 |
| RNU6-202P | DNAJB8 | 0.321 | 0.032760313 |
| RNU6-202P | OR51Q1 | 0.321 | 0.032760313 |
| RNU6-202P | RUFY2 | 0.321 | 0.032760313 |
| RNU6-202P | CCDC146 | 0.321 | 0.032760313 |
| RNU6-202P | SPICE1 | 0.321 | 0.032760313 |
| RNU6-202P | RFX3 | 0.321 | 0.032760313 |
| RNU6-202P | STK17A | 0.322 | 0.032213478 |
| RNU6-202P | ZNRF1 | 0.322 | 0.032213478 |
| RNU6-202P | AC024270.1 | 0.322 | 0.032213478 |
| RNU6-202P | ANKIB1 | 0.322 | 0.032213478 |
| RNU6-202P | TBC1D19 | 0.322 | 0.032213478 |
| RNU6-202P | AC013394.1 | 0.323 | 0.031674482 |
| RNU6-202P | PKD2 | 0.323 | 0.031674482 |
| RNU6-202P | ACMSD | 0.323 | 0.031674482 |
| RNU6-202P | SLC25A53 | 0.323 | 0.031674482 |
| RNU6-202P | KIAA0825 | 0.324 | 0.031143239 |
| RNU6-202P | GDAP1L1 | 0.325 | 0.030619661 |
| RNU6-202P | GCNT7 | 0.325 | 0.030619661 |
| RNU6-202P | RFLNA | 0.325 | 0.030619661 |
| RNU6-202P | ZNF148 | 0.325 | 0.030619661 |
| RNU6-202P | TNRC6B | 0.325 | 0.030619661 |
| RNU6-202P | KIAA0895 | 0.325 | 0.030619661 |
| RNU6-202P | CLDN10 | 0.325 | 0.030619661 |
| RNU6-202P | STXBP4 | 0.325 | 0.030619661 |
| RNU6-202P | BARHL1 | 0.326 | 0.03010366 |
| RNU6-202P | STAMBPL1 | 0.326 | 0.03010366 |
| RNU6-202P | AKAP9 | 0.326 | 0.03010366 |
| RNU6-202P | B3GAT2 | 0.326 | 0.03010366 |
| RNU6-202P | ANKRD39 | 0.326 | 0.03010366 |
| RNU6-202P | UBE2V1 | 0.327 | 0.02959515 |
| RNU6-202P | RAB22A | 0.327 | 0.02959515 |
| RNU6-202P | ZNF644 | 0.327 | 0.02959515 |
| RNU6-202P | KATNAL1 | 0.327 | 0.02959515 |
| RNU6-202P | CYP3A5 | 0.327 | 0.02959515 |
| RNU6-202P | SPRN | 0.328 | 0.029094045 |
| RNU6-202P | LZTS1 | 0.328 | 0.029094045 |
| RNU6-202P | C3orf84 | 0.328 | 0.029094045 |
| RNU6-202P | MED13L | 0.329 | 0.02860026 |
| RNU6-202P | JAKMIP3 | 0.329 | 0.02860026 |
| RNU6-202P | WDR33 | 0.329 | 0.02860026 |
| RNU6-202P | UBE2F-SCLY | 0.329 | 0.02860026 |
| RNU6-202P | KIAA1109 | 0.329 | 0.02860026 |
| RNU6-202P | USP34 | 0.329 | 0.02860026 |
| RNU6-202P | EFCAB5 | 0.329 | 0.02860026 |
| RNU6-202P | LHX1 | 0.33 | 0.028113709 |
| RNU6-202P | FBXO25 | 0.33 | 0.028113709 |
| RNU6-202P | AKAP2 | 0.33 | 0.028113709 |
| RNU6-202P | BBIP1 | 0.331 | 0.02763431 |
| RNU6-202P | KANSL1L | 0.331 | 0.02763431 |
| RNU6-202P | H3F3A | 0.331 | 0.02763431 |
| RNU6-202P | RNF32 | 0.331 | 0.02763431 |
| RNU6-202P | XRN1 | 0.332 | 0.027161977 |
| RNU6-202P | BIRC6 | 0.332 | 0.027161977 |
| RNU6-202P | CRHR1 | 0.332 | 0.027161977 |
| RNU6-202P | ZNF407 | 0.332 | 0.027161977 |
| RNU6-202P | TRAT1 | 0.333 | 0.026696628 |
| RNU6-202P | ZNF669 | 0.333 | 0.026696628 |
| RNU6-202P | CCNH | 0.333 | 0.026696628 |
| RNU6-202P | PPP1R12B | 0.333 | 0.026696628 |
| RNU6-202P | AC110814.1 | 0.333 | 0.026696628 |
| RNU6-202P | FTO | 0.333 | 0.026696628 |
| RNU6-202P | C18orf25 | 0.334 | 0.026238181 |
| RNU6-202P | PTPN14 | 0.334 | 0.026238181 |
| RNU6-202P | LRRC37B | 0.334 | 0.026238181 |
| RNU6-202P | VPREB1 | 0.334 | 0.026238181 |
| RNU6-202P | KIAA1958 | 0.334 | 0.026238181 |
| RNU6-202P | FBXL20 | 0.335 | 0.025786553 |
| RNU6-202P | GPR174 | 0.335 | 0.025786553 |
| RNU6-202P | CCNJL | 0.335 | 0.025786553 |
| RNU6-202P | FBXL4 | 0.335 | 0.025786553 |
| RNU6-202P | YAF2 | 0.335 | 0.025786553 |
| RNU6-202P | KANTR | 0.335 | 0.025786553 |
| RNU6-202P | CCDC7 | 0.336 | 0.025341664 |
| RNU6-202P | REV3L | 0.336 | 0.025341664 |
| RNU6-202P | VPS13B | 0.336 | 0.025341664 |
| RNU6-202P | SPATA21 | 0.336 | 0.025341664 |
| RNU6-202P | VANGL2 | 0.336 | 0.025341664 |
| RNU6-202P | GPRASP1 | 0.336 | 0.025341664 |
| RNU6-202P | GPR18 | 0.337 | 0.024903433 |
| RNU6-202P | BNC2 | 0.337 | 0.024903433 |
| RNU6-202P | STAG1 | 0.337 | 0.024903433 |
| RNU6-202P | CXCR6 | 0.337 | 0.024903433 |
| RNU6-202P | SUGCT | 0.337 | 0.024903433 |
| RNU6-202P | KIAA1147 | 0.338 | 0.02447178 |
| RNU6-202P | C3orf49 | 0.338 | 0.02447178 |
| RNU6-202P | KIAA1468 | 0.338 | 0.02447178 |
| RNU6-202P | GK3P | 0.338 | 0.02447178 |
| RNU6-202P | STPG2 | 0.338 | 0.02447178 |
| RNU6-202P | RAB28 | 0.338 | 0.02447178 |
| RNU6-202P | NAP1L5 | 0.338 | 0.02447178 |
| RNU6-202P | INO80D | 0.339 | 0.024046625 |
| RNU6-202P | AC009119.2 | 0.339 | 0.024046625 |
| RNU6-202P | PP2D1 | 0.34 | 0.023627891 |
| RNU6-202P | KIAA1614 | 0.34 | 0.023627891 |
| RNU6-202P | TMEM161B | 0.34 | 0.023627891 |
| RNU6-202P | SOGA3 | 0.34 | 0.023627891 |
| RNU6-202P | PLCB1 | 0.341 | 0.023215497 |
| RNU6-202P | OMD | 0.341 | 0.023215497 |
| RNU6-202P | ZNF254 | 0.341 | 0.023215497 |
| RNU6-202P | ZBTB20 | 0.341 | 0.023215497 |
| RNU6-202P | AMN1 | 0.342 | 0.022809368 |
| RNU6-202P | TMEM184A | 0.342 | 0.022809368 |
| RNU6-202P | CAVIN4 | 0.342 | 0.022809368 |
| RNU6-202P | GAL3ST4 | 0.342 | 0.022809368 |
| RNU6-202P | LHX9 | 0.342 | 0.022809368 |
| RNU6-202P | PRB1 | 0.343 | 0.022409425 |
| RNU6-202P | KMT5B | 0.343 | 0.022409425 |
| RNU6-202P | RLIM | 0.343 | 0.022409425 |
| RNU6-202P | ADAM20 | 0.343 | 0.022409425 |
| RNU6-202P | EID3 | 0.344 | 0.022015593 |
| RNU6-202P | OTULIN | 0.344 | 0.022015593 |
| RNU6-202P | GPR87 | 0.344 | 0.022015593 |
| RNU6-202P | ANKH | 0.345 | 0.021627796 |
| RNU6-202P | STAG3 | 0.345 | 0.021627796 |
| RNU6-202P | ZNF66 | 0.345 | 0.021627796 |
| RNU6-202P | RAB25 | 0.345 | 0.021627796 |
| RNU6-202P | ATP8B1 | 0.345 | 0.021627796 |
| RNU6-202P | CTHRC1 | 0.345 | 0.021627796 |
| RNU6-202P | CCDC171 | 0.345 | 0.021627796 |
| RNU6-202P | C1GALT1 | 0.345 | 0.021627796 |
| RNU6-202P | RBM4 | 0.345 | 0.021627796 |
| RNU6-202P | CDK19 | 0.346 | 0.021245959 |
| RNU6-202P | AHCYL2 | 0.347 | 0.020870007 |
| RNU6-202P | CDKAL1 | 0.347 | 0.020870007 |
| RNU6-202P | COX20 | 0.347 | 0.020870007 |
| RNU6-202P | SMO | 0.347 | 0.020870007 |
| RNU6-202P | ODF3 | 0.347 | 0.020870007 |
| RNU6-202P | ATG2B | 0.347 | 0.020870007 |
| RNU6-202P | ZNF709 | 0.347 | 0.020870007 |
| RNU6-202P | TMEM52B | 0.347 | 0.020870007 |
| RNU6-202P | NFAT5 | 0.348 | 0.020499867 |
| RNU6-202P | MED13 | 0.348 | 0.020499867 |
| RNU6-202P | HHIP | 0.348 | 0.020499867 |
| RNU6-202P | MFSD11 | 0.348 | 0.020499867 |
| RNU6-202P | CYP2A6 | 0.348 | 0.020499867 |
| RNU6-202P | TBX10 | 0.348 | 0.020499867 |
| RNU6-202P | CLDN20 | 0.349 | 0.020135465 |
| RNU6-202P | BRAF | 0.349 | 0.020135465 |
| RNU6-202P | RPRML | 0.349 | 0.020135465 |
| RNU6-202P | KCNJ13 | 0.349 | 0.020135465 |
| RNU6-202P | VPS13A | 0.349 | 0.020135465 |
| RNU6-202P | VGLL4 | 0.349 | 0.020135465 |
| RNU6-202P | XYLT1 | 0.349 | 0.020135465 |
| RNU6-202P | FGF11 | 0.349 | 0.020135465 |
| RNU6-202P | USP12 | 0.35 | 0.019776728 |
| RNU6-202P | ZNF484 | 0.351 | 0.019423585 |
| RNU6-202P | MYCBP2 | 0.351 | 0.019423585 |
| RNU6-202P | WNK4 | 0.351 | 0.019423585 |
| RNU6-202P | BBS9 | 0.351 | 0.019423585 |
| RNU6-202P | SERINC5 | 0.351 | 0.019423585 |
| RNU6-202P | SMAD2 | 0.351 | 0.019423585 |
| RNU6-202P | ITGA4 | 0.351 | 0.019423585 |
| RNU6-202P | KIF6 | 0.352 | 0.019075964 |
| RNU6-202P | AK9 | 0.352 | 0.019075964 |
| RNU6-202P | CPNE6 | 0.352 | 0.019075964 |
| RNU6-202P | HTR4 | 0.353 | 0.018733794 |
| RNU6-202P | RALGAPA2 | 0.353 | 0.018733794 |
| RNU6-202P | C9orf152 | 0.353 | 0.018733794 |
| RNU6-202P | PTBP2 | 0.353 | 0.018733794 |
| RNU6-202P | TRPC5 | 0.354 | 0.018397005 |
| RNU6-202P | WDR20 | 0.354 | 0.018397005 |
| RNU6-202P | MOB1B | 0.354 | 0.018397005 |
| RNU6-202P | TP53TG5 | 0.355 | 0.018065528 |
| RNU6-202P | TTC29 | 0.355 | 0.018065528 |
| RNU6-202P | SERAC1 | 0.355 | 0.018065528 |
| RNU6-202P | MEX3C | 0.356 | 0.017739293 |
| RNU6-202P | ERCC6 | 0.356 | 0.017739293 |
| RNU6-202P | MEF2A | 0.357 | 0.017418233 |
| RNU6-202P | ATP4A | 0.357 | 0.017418233 |
| RNU6-202P | GATS | 0.357 | 0.017418233 |
| RNU6-202P | SLC14A1 | 0.357 | 0.017418233 |
| RNU6-202P | TSPAN11 | 0.357 | 0.017418233 |
| RNU6-202P | ARHGAP10 | 0.358 | 0.017102279 |
| RNU6-202P | KCNJ1 | 0.358 | 0.017102279 |
| RNU6-202P | MAP4K3 | 0.358 | 0.017102279 |
| RNU6-202P | SKOR1 | 0.358 | 0.017102279 |
| RNU6-202P | BRWD1 | 0.359 | 0.016791365 |
| RNU6-202P | RBM33 | 0.359 | 0.016791365 |
| RNU6-202P | ZNF800 | 0.359 | 0.016791365 |
| RNU6-202P | PRH1 | 0.36 | 0.016485424 |
| RNU6-202P | RALGAPB | 0.36 | 0.016485424 |
| RNU6-202P | HHAT | 0.361 | 0.01618439 |
| RNU6-202P | GGT6 | 0.361 | 0.01618439 |
| RNU6-202P | NUTM2B | 0.361 | 0.01618439 |
| RNU6-202P | SLC38A9 | 0.361 | 0.01618439 |
| RNU6-202P | ANKRD7 | 0.362 | 0.015888198 |
| RNU6-202P | MBD5 | 0.363 | 0.015596783 |
| RNU6-202P | DOCK4 | 0.364 | 0.015310082 |
| RNU6-202P | SHPRH | 0.364 | 0.015310082 |
| RNU6-202P | MGAT4A | 0.365 | 0.015028031 |
| RNU6-202P | MPP5 | 0.365 | 0.015028031 |
| RNU6-202P | KCNJ6 | 0.365 | 0.015028031 |
| RNU6-202P | RLN1 | 0.366 | 0.014750566 |
| RNU6-202P | OCLM | 0.366 | 0.014750566 |
| RNU6-202P | ZBED6 | 0.366 | 0.014750566 |
| RNU6-202P | VPS13D | 0.367 | 0.014477627 |
| RNU6-202P | SPIN2A | 0.368 | 0.014209151 |
| RNU6-202P | FNIP1 | 0.368 | 0.014209151 |
| RNU6-202P | ABCA12 | 0.368 | 0.014209151 |
| RNU6-202P | TUBGCP3 | 0.368 | 0.014209151 |
| RNU6-202P | MATR3 | 0.369 | 0.013945077 |
| RNU6-202P | MFSD4A | 0.369 | 0.013945077 |
| RNU6-202P | GPC2 | 0.369 | 0.013945077 |
| RNU6-202P | ZNF564 | 0.369 | 0.013945077 |
| RNU6-202P | FAM214A | 0.369 | 0.013945077 |
| RNU6-202P | ZNF236 | 0.369 | 0.013945077 |
| RNU6-202P | ZNF91 | 0.37 | 0.013685345 |
| RNU6-202P | UBN2 | 0.37 | 0.013685345 |
| RNU6-202P | FNDC9 | 0.37 | 0.013685345 |
| RNU6-202P | LRRN3 | 0.37 | 0.013685345 |
| RNU6-202P | GPATCH8 | 0.371 | 0.013429896 |
| RNU6-202P | GPR171 | 0.371 | 0.013429896 |
| RNU6-202P | HSD17B1 | 0.372 | 0.013178669 |
| RNU6-202P | F2RL2 | 0.372 | 0.013178669 |
| RNU6-202P | SPDYE5 | 0.372 | 0.013178669 |
| RNU6-202P | TAS2R20 | 0.372 | 0.013178669 |
| RNU6-202P | DYRK2 | 0.373 | 0.012931607 |
| RNU6-202P | MKL2 | 0.373 | 0.012931607 |
| RNU6-202P | BPTF | 0.374 | 0.012688651 |
| RNU6-202P | GABRB2 | 0.375 | 0.012449744 |
| RNU6-202P | KLHL32 | 0.375 | 0.012449744 |
| RNU6-202P | NF1 | 0.376 | 0.01221483 |
| RNU6-202P | ELF2 | 0.376 | 0.01221483 |
| RNU6-202P | SP4 | 0.376 | 0.01221483 |
| RNU6-202P | TBC1D32 | 0.376 | 0.01221483 |
| RNU6-202P | RBM44 | 0.376 | 0.01221483 |
| RNU6-202P | TMEM196 | 0.377 | 0.011983852 |
| RNU6-202P | AJUBA | 0.377 | 0.011983852 |
| RNU6-202P | CDK13 | 0.377 | 0.011983852 |
| RNU6-202P | AC068896.1 | 0.378 | 0.011756754 |
| RNU6-202P | BNIP3 | 0.378 | 0.011756754 |
| RNU6-202P | PIAS2 | 0.378 | 0.011756754 |
| RNU6-202P | TAS2R3 | 0.378 | 0.011756754 |
| RNU6-202P | ADH4 | 0.379 | 0.011533481 |
| RNU6-202P | MLYCD | 0.379 | 0.011533481 |
| RNU6-202P | SLC30A4 | 0.379 | 0.011533481 |
| RNU6-202P | MON2 | 0.379 | 0.011533481 |
| RNU6-202P | DHX36 | 0.381 | 0.011098195 |
| RNU6-202P | SLC35F5 | 0.382 | 0.010886074 |
| RNU6-202P | NR3C2 | 0.383 | 0.010677564 |
| RNU6-202P | ZNF750 | 0.383 | 0.010677564 |
| RNU6-202P | SSBP2 | 0.383 | 0.010677564 |
| RNU6-202P | OR6V1 | 0.383 | 0.010677564 |
| RNU6-202P | MKLN1 | 0.383 | 0.010677564 |
| RNU6-202P | DST | 0.385 | 0.01027117 |
| RNU6-202P | KPNA5 | 0.386 | 0.010073182 |
| RNU6-202P | ASH1L | 0.386 | 0.010073182 |
| RNU6-202P | SENP7 | 0.386 | 0.010073182 |
| RNU6-202P | TPP2 | 0.386 | 0.010073182 |
| RNU6-202P | GPR82 | 0.387 | 0.009878601 |
| RNU6-202P | RTTN | 0.387 | 0.009878601 |
| RNU6-202P | SPINK7 | 0.388 | 0.009687375 |
| RNU6-202P | CDK12 | 0.388 | 0.009687375 |
| RNU6-202P | LINC00238 | 0.388 | 0.009687375 |
| RNU6-202P | AKAP6 | 0.388 | 0.009687375 |
| RNU6-202P | RICTOR | 0.389 | 0.009499456 |
| RNU6-202P | VPS13C | 0.389 | 0.009499456 |
| RNU6-202P | LRP2 | 0.389 | 0.009499456 |
| RNU6-202P | RBP2 | 0.389 | 0.009499456 |
| RNU6-202P | RBM14-RBM4 | 0.39 | 0.009314795 |
| RNU6-202P | LYPD8 | 0.39 | 0.009314795 |
| RNU6-202P | FOXC2 | 0.39 | 0.009314795 |
| RNU6-202P | ZNF124 | 0.391 | 0.009133343 |
| RNU6-202P | FCHSD2 | 0.392 | 0.008955054 |
| RNU6-202P | PPM1A | 0.393 | 0.008779879 |
| RNU6-202P | ERCC6L2 | 0.393 | 0.008779879 |
| RNU6-202P | C1QTNF3-AMACR | 0.395 | 0.008438687 |
| RNU6-202P | CNOT4 | 0.395 | 0.008438687 |
| RNU6-202P | SPINK9 | 0.395 | 0.008438687 |
| RNU6-202P | BTBD11 | 0.395 | 0.008438687 |
| RNU6-202P | KCNK16 | 0.396 | 0.008272579 |
| RNU6-202P | CASD1 | 0.397 | 0.008109403 |
| RNU6-202P | BCL2 | 0.397 | 0.008109403 |
| RNU6-202P | ACVR2B | 0.398 | 0.007949114 |
| RNU6-202P | TAS2R40 | 0.4 | 0.007637022 |
| RNU6-202P | ZNF652 | 0.401 | 0.007485132 |
| RNU6-202P | TAS2R14 | 0.401 | 0.007485132 |
| RNU6-202P | CKMT2 | 0.402 | 0.007335958 |
| RNU6-202P | RASA1 | 0.403 | 0.007189456 |
| RNU6-202P | RALGAPA1 | 0.403 | 0.007189456 |
| RNU6-202P | KIAA0408 | 0.404 | 0.007045586 |
| RNU6-202P | DENND4C | 0.404 | 0.007045586 |
| RNU6-202P | RCOR3 | 0.405 | 0.006904306 |
| RNU6-202P | SLC4A4 | 0.406 | 0.006765576 |
| RNU6-202P | ZMYM2 | 0.406 | 0.006765576 |
| RNU6-202P | LRCH3 | 0.407 | 0.006629357 |
| RNU6-202P | ZNF548 | 0.408 | 0.006495609 |
| RNU6-202P | KCNIP4 | 0.411 | 0.006108805 |
| RNU6-202P | WDPCP | 0.413 | 0.005862591 |
| RNU6-202P | FAM196B | 0.413 | 0.005862591 |
| RNU6-202P | KLF12 | 0.414 | 0.00574287 |
| RNU6-202P | RHOH | 0.414 | 0.00574287 |
| RNU6-202P | SYNRG | 0.414 | 0.00574287 |
| RNU6-202P | LRIG1 | 0.416 | 0.00551002 |
| RNU6-202P | ARHGAP5 | 0.416 | 0.00551002 |
| RNU6-202P | LRRC18 | 0.416 | 0.00551002 |
| RNU6-202P | ANGPTL3 | 0.418 | 0.005285725 |
| RNU6-202P | CENPC | 0.418 | 0.005285725 |
| RNU6-202P | ZNF460 | 0.419 | 0.005176699 |
| RNU6-202P | SLC4A7 | 0.421 | 0.004964723 |
| RNU6-202P | BRWD3 | 0.421 | 0.004964723 |
| RNU6-202P | EMSY | 0.421 | 0.004964723 |
| RNU6-202P | KMT2C | 0.421 | 0.004964723 |
| RNU6-202P | PHC3 | 0.421 | 0.004964723 |
| RNU6-202P | FOXF1 | 0.422 | 0.004861706 |
| RNU6-202P | APPL2 | 0.423 | 0.004760627 |
| RNU6-202P | TRDMT1 | 0.424 | 0.004661454 |
| RNU6-202P | AUH | 0.425 | 0.004564155 |
| RNU6-202P | LMO7 | 0.428 | 0.004283195 |
| RNU6-202P | PRR4 | 0.429 | 0.004193087 |
| RNU6-202P | ACKR4 | 0.429 | 0.004193087 |
| RNU6-202P | KLHL11 | 0.429 | 0.004193087 |
| RNU6-202P | KCNH1 | 0.432 | 0.003932984 |
| RNU6-202P | MDM4 | 0.433 | 0.003849595 |
| RNU6-202P | TAS2R30 | 0.434 | 0.003767816 |
| RNU6-202P | SERPIND1 | 0.435 | 0.003687619 |
| RNU6-202P | KCNJ9 | 0.439 | 0.003382115 |
| RNU6-202P | COA1 | 0.439 | 0.003382115 |
| RNU6-202P | N4BP2 | 0.44 | 0.00330943 |
| RNU6-202P | ATXN7 | 0.441 | 0.00323817 |
| RNU6-202P | KMT2E | 0.444 | 0.003032698 |
| RNU6-202P | TAS2R13 | 0.444 | 0.003032698 |
| RNU6-202P | PRH2 | 0.444 | 0.003032698 |
| RNU6-202P | JPH3 | 0.446 | 0.0029024 |
| RNU6-202P | SECISBP2 | 0.446 | 0.0029024 |
| RNU6-202P | FRYL | 0.448 | 0.002777231 |
| RNU6-202P | CEMIP | 0.448 | 0.002777231 |
| RNU6-202P | ZNF292 | 0.448 | 0.002777231 |
| RNU6-202P | ANGPTL7 | 0.451 | 0.002598702 |
| RNU6-202P | TAS2R19 | 0.453 | 0.002485579 |
| RNU6-202P | AUTS2 | 0.455 | 0.002376979 |
| RNU6-202P | GPR22 | 0.455 | 0.002376979 |
| RNU6-202P | OR9A2 | 0.456 | 0.002324324 |
| RNU6-202P | AL136531.2 | 0.457 | 0.002272739 |
| RNU6-202P | ADORA2A | 0.458 | 0.002222204 |
| RNU6-202P | AKAP5 | 0.458 | 0.002222204 |
| RNU6-202P | KCTD4 | 0.459 | 0.002172702 |
| RNU6-202P | HTR2B | 0.46 | 0.002124212 |
| RNU6-202P | ZP2 | 0.463 | 0.001984635 |
| RNU6-202P | KANSL1 | 0.464 | 0.001940014 |
| RNU6-202P | NKX6-2 | 0.466 | 0.001853522 |
| RNU6-202P | CPSF4L | 0.468 | 0.001770586 |
| RNU6-202P | PTPN4 | 0.469 | 0.00173041 |
| RNU6-202P | B3GALT2 | 0.47 | 0.001691074 |
| RNU6-202P | POU5F2 | 0.47 | 0.001691074 |
| RNU6-202P | WASL | 0.47 | 0.001691074 |
| RNU6-202P | ZNF235 | 0.471 | 0.001652562 |
| RNU6-202P | CD47 | 0.477 | 0.001437942 |
| RNU6-202P | SCUBE3 | 0.478 | 0.001404777 |
| RNU6-202P | ZNF782 | 0.478 | 0.001404777 |
| RNU6-202P | TAS2R46 | 0.481 | 0.001309467 |
| RNU6-202P | STX19 | 0.483 | 0.001249281 |
| RNU6-202P | TMSB15B | 0.484 | 0.001220156 |
| RNU6-202P | SMURF2 | 0.488 | 0.001109815 |
| RNU6-202P | ATAD2B | 0.491 | 0.001033197 |
| RNU6-202P | AP003108.2 | 0.498 | 0.00087306 |
| RNU6-202P | OMG | 0.503 | 0.000773117 |
| RNU6-202P | ANGPTL1 | 0.503 | 0.000773117 |
| RNU6-202P | TAS2R39 | 0.504 | 0.000754449 |
| RNU6-202P | TAS2R50 | 0.505 | 0.000736201 |
| RNU6-202P | RNU6-946P | 0.513 | 0.000604305 |
| RNU6-202P | HACE1 | 0.514 | 0.000589461 |
| RNU6-202P | GPR52 | 0.516 | 0.000560786 |
| RNU6-202P | SRGAP3 | 0.519 | 0.000520201 |
| RNU6-202P | ROPN1B | 0.533 | 0.000364501 |
| RNU6-202P | TAS2R31 | 0.535 | 0.000346205 |
| RNU6-202P | COL10A1 | 0.571 | 0.000133035 |
| RNU6-202P | CASK | 0.656 | 1.11E-05 |
| RNU6-202P | RNU6-202P | 1 | 1.90E-11 |
| RNU6-110P | PSMD2 | -0.355 | 0.005239967 |
| RNU6-110P | PIGC | -0.352 | 0.005641535 |
| RNU6-110P | PGM1 | -0.35 | 0.005924458 |
| RNU6-110P | SRP14 | -0.349 | 0.006070661 |
| RNU6-110P | SH3GLB2 | -0.345 | 0.006688517 |
| RNU6-110P | RTCB | -0.342 | 0.007188402 |
| RNU6-110P | MRPS7 | -0.341 | 0.007362334 |
| RNU6-110P | AHSA1 | -0.338 | 0.007907008 |
| RNU6-110P | SUCLG1 | -0.333 | 0.008895376 |
| RNU6-110P | EMC3 | -0.332 | 0.009105807 |
| RNU6-110P | PARK7 | -0.331 | 0.009320672 |
| RNU6-110P | MAP3K10 | -0.328 | 0.009992661 |
| RNU6-110P | PJA1 | -0.327 | 0.010226061 |
| RNU6-110P | PSMB1 | -0.32 | 0.012000227 |
| RNU6-110P | UQCC2 | -0.317 | 0.012840664 |
| RNU6-110P | CISD3 | -0.313 | 0.014042071 |
| RNU6-110P | DDX24 | -0.309 | 0.015341622 |
| RNU6-110P | RTL8C | -0.307 | 0.016030245 |
| RNU6-110P | SLC25A28 | -0.306 | 0.016384638 |
| RNU6-110P | FAM50A | -0.306 | 0.016384638 |
| RNU6-110P | MRPL58 | -0.304 | 0.017114128 |
| RNU6-110P | DUSP23 | -0.304 | 0.017114128 |
| RNU6-110P | TRAPPC4 | -0.304 | 0.017114128 |
| RNU6-110P | CXorf40B | -0.303 | 0.017489445 |
| RNU6-110P | PRPS1 | -0.303 | 0.017489445 |
| RNU6-110P | BEX4 | -0.302 | 0.017871958 |
| RNU6-110P | PLBD2 | -0.302 | 0.017871958 |
| RNU6-110P | NDUFS6 | -0.301 | 0.018261782 |
| RNU6-110P | PSMC3 | -0.299 | 0.019063818 |
| RNU6-110P | EIF3I | -0.299 | 0.019063818 |
| RNU6-110P | PORCN | -0.297 | 0.01989648 |
| RNU6-110P | PSMC5 | -0.296 | 0.020324593 |
| RNU6-110P | BEX3 | -0.296 | 0.020324593 |
| RNU6-110P | ANAPC11 | -0.296 | 0.020324593 |
| RNU6-110P | UCK1 | -0.296 | 0.020324593 |
| RNU6-110P | PITHD1 | -0.294 | 0.021204981 |
| RNU6-110P | PSMG3 | -0.293 | 0.021657502 |
| RNU6-110P | ARFIP2 | -0.293 | 0.021657502 |
| RNU6-110P | NPRL2 | -0.293 | 0.021657502 |
| RNU6-110P | WDR61 | -0.293 | 0.021657502 |
| RNU6-110P | SDSL | -0.292 | 0.022118405 |
| RNU6-110P | PPP1R7 | -0.292 | 0.022118405 |
| RNU6-110P | RNF113A | -0.291 | 0.022587816 |
| RNU6-110P | CDK11B | -0.291 | 0.022587816 |
| RNU6-110P | VCP | -0.289 | 0.023552669 |
| RNU6-110P | TEX261 | -0.288 | 0.024048368 |
| RNU6-110P | CRAT | -0.288 | 0.024048368 |
| RNU6-110P | C1orf122 | -0.288 | 0.024048368 |
| RNU6-110P | DPP7 | -0.287 | 0.024553088 |
| RNU6-110P | LSM4 | -0.287 | 0.024553088 |
| RNU6-110P | KHK | -0.286 | 0.025066961 |
| RNU6-110P | POP4 | -0.286 | 0.025066961 |
| RNU6-110P | KPTN | -0.285 | 0.025590119 |
| RNU6-110P | ADSL | -0.282 | 0.027216646 |
| RNU6-110P | PSMA7 | -0.281 | 0.027778294 |
| RNU6-110P | GPR108 | -0.281 | 0.027778294 |
| RNU6-110P | DCXR | -0.281 | 0.027778294 |
| RNU6-110P | MRPL20 | -0.28 | 0.028349907 |
| RNU6-110P | HSD17B10 | -0.28 | 0.028349907 |
| RNU6-110P | FUNDC2 | -0.28 | 0.028349907 |
| RNU6-110P | C9orf142 | -0.28 | 0.028349907 |
| RNU6-110P | PGP | -0.28 | 0.028349907 |
| RNU6-110P | RASSF7 | -0.278 | 0.02952359 |
| RNU6-110P | LRRC49 | -0.278 | 0.02952359 |
| RNU6-110P | HRAS | -0.278 | 0.02952359 |
| RNU6-110P | HPS1 | -0.277 | 0.030125943 |
| RNU6-110P | SPECC1L | -0.277 | 0.030125943 |
| RNU6-110P | CYC1 | -0.277 | 0.030125943 |
| RNU6-110P | SLC8B1 | -0.276 | 0.030738828 |
| RNU6-110P | ENO1 | -0.276 | 0.030738828 |
| RNU6-110P | EIF4A3 | -0.276 | 0.030738828 |
| RNU6-110P | HDDC2 | -0.274 | 0.031996769 |
| RNU6-110P | FAM96B | -0.274 | 0.031996769 |
| RNU6-110P | MSRB2 | -0.274 | 0.031996769 |
| RNU6-110P | DDX39A | -0.273 | 0.032642118 |
| RNU6-110P | SSNA1 | -0.273 | 0.032642118 |
| RNU6-110P | MOSPD3 | -0.273 | 0.032642118 |
| RNU6-110P | VPS16 | -0.273 | 0.032642118 |
| RNU6-110P | PIH1D1 | -0.273 | 0.032642118 |
| RNU6-110P | ATP1A1 | -0.273 | 0.032642118 |
| RNU6-110P | PSMB6 | -0.273 | 0.032642118 |
| RNU6-110P | TOE1 | -0.273 | 0.032642118 |
| RNU6-110P | BOP1 | -0.272 | 0.033298581 |
| RNU6-110P | JTB | -0.272 | 0.033298581 |
| RNU6-110P | ZMYND19 | -0.272 | 0.033298581 |
| RNU6-110P | NDUFAF8 | -0.272 | 0.033298581 |
| RNU6-110P | NUDC | -0.271 | 0.033966309 |
| RNU6-110P | STK16 | -0.27 | 0.034645451 |
| RNU6-110P | ARHGEF19 | -0.27 | 0.034645451 |
| RNU6-110P | SOD1 | -0.27 | 0.034645451 |
| RNU6-110P | BATF | -0.27 | 0.034645451 |
| RNU6-110P | PEPD | -0.27 | 0.034645451 |
| RNU6-110P | DHRS4L2 | -0.269 | 0.035336158 |
| RNU6-110P | TMEM214 | -0.269 | 0.035336158 |
| RNU6-110P | FAM207A | -0.269 | 0.035336158 |
| RNU6-110P | SPR | -0.269 | 0.035336158 |
| RNU6-110P | TCEAL3 | -0.268 | 0.036038582 |
| RNU6-110P | DCTN1 | -0.268 | 0.036038582 |
| RNU6-110P | MRPS12 | -0.268 | 0.036038582 |
| RNU6-110P | SMPD1 | -0.268 | 0.036038582 |
| RNU6-110P | SIRT6 | -0.268 | 0.036038582 |
| RNU6-110P | ACAT2 | -0.267 | 0.036752876 |
| RNU6-110P | PLOD1 | -0.267 | 0.036752876 |
| RNU6-110P | PUF60 | -0.267 | 0.036752876 |
| RNU6-110P | TMEM205 | -0.267 | 0.036752876 |
| RNU6-110P | MESP1 | -0.267 | 0.036752876 |
| RNU6-110P | UBB | -0.266 | 0.037479194 |
| RNU6-110P | IDH3B | -0.266 | 0.037479194 |
| RNU6-110P | NDUFS7 | -0.266 | 0.037479194 |
| RNU6-110P | PIDD1 | -0.266 | 0.037479194 |
| RNU6-110P | ACOT7 | -0.266 | 0.037479194 |
| RNU6-110P | PRELID1 | -0.266 | 0.037479194 |
| RNU6-110P | TOR2A | -0.266 | 0.037479194 |
| RNU6-110P | PHPT1 | -0.266 | 0.037479194 |
| RNU6-110P | DCTN3 | -0.264 | 0.038968525 |
| RNU6-110P | NCBP2-AS2 | -0.264 | 0.038968525 |
| RNU6-110P | POFUT2 | -0.264 | 0.038968525 |
| RNU6-110P | POLR2G | -0.264 | 0.038968525 |
| RNU6-110P | MED27 | -0.263 | 0.039731852 |
| RNU6-110P | BBC3 | -0.263 | 0.039731852 |
| RNU6-110P | SARS | -0.263 | 0.039731852 |
| RNU6-110P | SNRNP25 | -0.263 | 0.039731852 |
| RNU6-110P | RPS6KA1 | -0.263 | 0.039731852 |
| RNU6-110P | ABCF1 | -0.263 | 0.039731852 |
| RNU6-110P | MED8 | -0.263 | 0.039731852 |
| RNU6-110P | ZPR1 | -0.262 | 0.04050783 |
| RNU6-110P | CCND3 | -0.262 | 0.04050783 |
| RNU6-110P | KIAA0895L | -0.262 | 0.04050783 |
| RNU6-110P | SNRNP200 | -0.262 | 0.04050783 |
| RNU6-110P | FAAP24 | -0.262 | 0.04050783 |
| RNU6-110P | AATF | -0.262 | 0.04050783 |
| RNU6-110P | CENPX | -0.262 | 0.04050783 |
| RNU6-110P | PSMD7 | -0.262 | 0.04050783 |
| RNU6-110P | FBXW5 | -0.262 | 0.04050783 |
| RNU6-110P | SIVA1 | -0.262 | 0.04050783 |
| RNU6-110P | PLEKHJ1 | -0.262 | 0.04050783 |
| RNU6-110P | PAF1 | -0.261 | 0.041296618 |
| RNU6-110P | NDUFA11 | -0.261 | 0.041296618 |
| RNU6-110P | CXXC1 | -0.26 | 0.042098377 |
| RNU6-110P | SUPT5H | -0.26 | 0.042098377 |
| RNU6-110P | RAN | -0.26 | 0.042098377 |
| RNU6-110P | HDLBP | -0.26 | 0.042098377 |
| RNU6-110P | SRP68 | -0.26 | 0.042098377 |
| RNU6-110P | EXOSC7 | -0.259 | 0.042913269 |
| RNU6-110P | GOT1 | -0.259 | 0.042913269 |
| RNU6-110P | HIRA | -0.259 | 0.042913269 |
| RNU6-110P | PSMB3 | -0.259 | 0.042913269 |
| RNU6-110P | RPS19BP1 | -0.259 | 0.042913269 |
| RNU6-110P | NAPA | -0.259 | 0.042913269 |
| RNU6-110P | WDR83OS | -0.259 | 0.042913269 |
| RNU6-110P | SRF | -0.258 | 0.043741455 |
| RNU6-110P | ABCA7 | -0.258 | 0.043741455 |
| RNU6-110P | CCT3 | -0.258 | 0.043741455 |
| RNU6-110P | ZC3H12A | -0.258 | 0.043741455 |
| RNU6-110P | MFSD7 | -0.258 | 0.043741455 |
| RNU6-110P | COMMD4 | -0.258 | 0.043741455 |
| RNU6-110P | SRXN1 | -0.258 | 0.043741455 |
| RNU6-110P | SRSF4 | -0.257 | 0.044583098 |
| RNU6-110P | CNOT3 | -0.257 | 0.044583098 |
| RNU6-110P | CCDC85B | -0.257 | 0.044583098 |
| RNU6-110P | IRAK1 | -0.257 | 0.044583098 |
| RNU6-110P | DDX27 | -0.257 | 0.044583098 |
| RNU6-110P | NSMF | -0.256 | 0.045438362 |
| RNU6-110P | IFT57 | -0.256 | 0.045438362 |
| RNU6-110P | GUK1 | -0.256 | 0.045438362 |
| RNU6-110P | C19orf47 | -0.255 | 0.046307413 |
| RNU6-110P | C2orf81 | -0.255 | 0.046307413 |
| RNU6-110P | TCEAL9 | -0.255 | 0.046307413 |
| RNU6-110P | COX8A | -0.255 | 0.046307413 |
| RNU6-110P | DPM2 | -0.255 | 0.046307413 |
| RNU6-110P | PUSL1 | -0.255 | 0.046307413 |
| RNU6-110P | LAGE3 | -0.254 | 0.047190417 |
| RNU6-110P | NELFE | -0.254 | 0.047190417 |
| RNU6-110P | SELPLG | -0.254 | 0.047190417 |
| RNU6-110P | KRI1 | -0.254 | 0.047190417 |
| RNU6-110P | ASCC2 | -0.254 | 0.047190417 |
| RNU6-110P | NABP2 | -0.254 | 0.047190417 |
| RNU6-110P | PRDX5 | -0.254 | 0.047190417 |
| RNU6-110P | MZT2B | -0.254 | 0.047190417 |
| RNU6-110P | PI4KB | -0.254 | 0.047190417 |
| RNU6-110P | ACD | -0.254 | 0.047190417 |
| RNU6-110P | SLC27A4 | -0.253 | 0.048087539 |
| RNU6-110P | NDUFB10 | -0.253 | 0.048087539 |
| RNU6-110P | PRADC1 | -0.253 | 0.048087539 |
| RNU6-110P | RING1 | -0.253 | 0.048087539 |
| RNU6-110P | DTD1 | -0.252 | 0.048998947 |
| RNU6-110P | COASY | -0.252 | 0.048998947 |
| RNU6-110P | PSMC4 | -0.252 | 0.048998947 |
| RNU6-110P | PHB | -0.252 | 0.048998947 |
| RNU6-110P | CDK10 | -0.252 | 0.048998947 |
| RNU6-110P | MFN2 | -0.251 | 0.04992481 |
| RNU6-110P | MIEN1 | -0.251 | 0.04992481 |
| RNU6-110P | MIGA2 | -0.251 | 0.04992481 |
| RNU6-110P | MT1F | -0.251 | 0.04992481 |
| RNU6-110P | DGAT1 | -0.251 | 0.04992481 |
| RNU6-110P | SPATA20 | -0.251 | 0.04992481 |
| RNU6-110P | HEBP1 | -0.251 | 0.04992481 |
| RNU6-110P | NAGLU | -0.251 | 0.04992481 |
| RNU6-110P | OTUB1 | -0.251 | 0.04992481 |
| RNU6-110P | COPS6 | -0.251 | 0.04992481 |
| RNU6-110P | SEC61A2 | 0.24 | 0.049524522 |
| RNU6-110P | ENTPD3 | 0.24 | 0.049524522 |
| RNU6-110P | ABHD17B | 0.24 | 0.049524522 |
| RNU6-110P | SERPIND1 | 0.24 | 0.049524522 |
| RNU6-110P | C21orf62 | 0.24 | 0.049524522 |
| RNU6-110P | SERPINE2 | 0.24 | 0.049524522 |
| RNU6-110P | PHLDB3 | 0.24 | 0.049524522 |
| RNU6-110P | ZNF260 | 0.24 | 0.049524522 |
| RNU6-110P | TMEM168 | 0.24 | 0.049524522 |
| RNU6-110P | MYOT | 0.24 | 0.049524522 |
| RNU6-110P | AVL9 | 0.24 | 0.049524522 |
| RNU6-110P | ZNF461 | 0.24 | 0.049524522 |
| RNU6-110P | ZNF431 | 0.24 | 0.049524522 |
| RNU6-110P | CDK13 | 0.24 | 0.049524522 |
| RNU6-110P | LIMS1 | 0.24 | 0.049524522 |
| RNU6-110P | PIPOX | 0.241 | 0.048604901 |
| RNU6-110P | NR3C1 | 0.241 | 0.048604901 |
| RNU6-110P | TMEM263 | 0.241 | 0.048604901 |
| RNU6-110P | COX18 | 0.241 | 0.048604901 |
| RNU6-110P | ZNF320 | 0.241 | 0.048604901 |
| RNU6-110P | TSPYL6 | 0.241 | 0.048604901 |
| RNU6-110P | ATXN3 | 0.242 | 0.047699662 |
| RNU6-110P | SLC51A | 0.242 | 0.047699662 |
| RNU6-110P | CDK15 | 0.242 | 0.047699662 |
| RNU6-110P | GPR20 | 0.242 | 0.047699662 |
| RNU6-110P | SLC25A30 | 0.242 | 0.047699662 |
| RNU6-110P | PDZK1 | 0.242 | 0.047699662 |
| RNU6-110P | PAFAH1B1 | 0.242 | 0.047699662 |
| RNU6-110P | IKZF2 | 0.242 | 0.047699662 |
| RNU6-110P | TMEM243 | 0.242 | 0.047699662 |
| RNU6-110P | WNT7A | 0.242 | 0.047699662 |
| RNU6-110P | CEP68 | 0.242 | 0.047699662 |
| RNU6-110P | MAPKAPK3 | 0.242 | 0.047699662 |
| RNU6-110P | POC1B | 0.243 | 0.046808638 |
| RNU6-110P | NFE2L1 | 0.243 | 0.046808638 |
| RNU6-110P | FUT11 | 0.243 | 0.046808638 |
| RNU6-110P | CDH13 | 0.243 | 0.046808638 |
| RNU6-110P | PBLD | 0.243 | 0.046808638 |
| RNU6-110P | PROSER2 | 0.244 | 0.04593166 |
| RNU6-110P | BUB1B-PAK6 | 0.244 | 0.04593166 |
| RNU6-110P | AC005779.2 | 0.244 | 0.04593166 |
| RNU6-110P | ACTL10 | 0.244 | 0.04593166 |
| RNU6-110P | CENPC | 0.244 | 0.04593166 |
| RNU6-110P | EDDM13 | 0.245 | 0.045068563 |
| RNU6-110P | TAS2R50 | 0.245 | 0.045068563 |
| RNU6-110P | ODF3 | 0.245 | 0.045068563 |
| RNU6-110P | DNAJC14 | 0.245 | 0.045068563 |
| RNU6-110P | SMAD4 | 0.245 | 0.045068563 |
| RNU6-110P | S100Z | 0.246 | 0.044219182 |
| RNU6-110P | ZNF430 | 0.246 | 0.044219182 |
| RNU6-110P | HERPUD2 | 0.246 | 0.044219182 |
| RNU6-110P | SDCCAG8 | 0.246 | 0.044219182 |
| RNU6-110P | CRIP2 | 0.246 | 0.044219182 |
| RNU6-110P | ST6GALNAC2 | 0.246 | 0.044219182 |
| RNU6-110P | DLEU1 | 0.246 | 0.044219182 |
| RNU6-110P | CD58 | 0.246 | 0.044219182 |
| RNU6-110P | HUS1 | 0.247 | 0.043383351 |
| RNU6-110P | TSPAN7 | 0.247 | 0.043383351 |
| RNU6-110P | ADH4 | 0.247 | 0.043383351 |
| RNU6-110P | RNF212B | 0.247 | 0.043383351 |
| RNU6-110P | SCN1B | 0.247 | 0.043383351 |
| RNU6-110P | AC092587.1 | 0.247 | 0.043383351 |
| RNU6-110P | CLSTN1 | 0.247 | 0.043383351 |
| RNU6-110P | FBXL13 | 0.248 | 0.042560907 |
| RNU6-110P | TSSK3 | 0.248 | 0.042560907 |
| RNU6-110P | ZNF85 | 0.248 | 0.042560907 |
| RNU6-110P | DPP8 | 0.248 | 0.042560907 |
| RNU6-110P | ZC3HAV1L | 0.248 | 0.042560907 |
| RNU6-110P | TGFB1I1 | 0.248 | 0.042560907 |
| RNU6-110P | ETNK1 | 0.248 | 0.042560907 |
| RNU6-110P | CHRNB4 | 0.248 | 0.042560907 |
| RNU6-110P | AC006030.1 | 0.248 | 0.042560907 |
| RNU6-110P | GNPTAB | 0.248 | 0.042560907 |
| RNU6-110P | PCDHGA7 | 0.248 | 0.042560907 |
| RNU6-110P | THAP5 | 0.249 | 0.041751688 |
| RNU6-110P | GLS | 0.249 | 0.041751688 |
| RNU6-110P | KLF17 | 0.249 | 0.041751688 |
| RNU6-110P | SIPA1L2 | 0.249 | 0.041751688 |
| RNU6-110P | TCAIM | 0.249 | 0.041751688 |
| RNU6-110P | CYP2B6 | 0.249 | 0.041751688 |
| RNU6-110P | ATP5S | 0.249 | 0.041751688 |
| RNU6-110P | CRAMP1 | 0.249 | 0.041751688 |
| RNU6-110P | KCTD4 | 0.249 | 0.041751688 |
| RNU6-110P | PRSS42 | 0.249 | 0.041751688 |
| RNU6-110P | CCDC7 | 0.25 | 0.040955531 |
| RNU6-110P | NRN1L | 0.25 | 0.040955531 |
| RNU6-110P | HPGDS | 0.25 | 0.040955531 |
| RNU6-110P | ZCCHC7 | 0.25 | 0.040955531 |
| RNU6-110P | RCSD1 | 0.25 | 0.040955531 |
| RNU6-110P | TMEM225B | 0.25 | 0.040955531 |
| RNU6-110P | ATP8B1 | 0.25 | 0.040955531 |
| RNU6-110P | ZNF816 | 0.25 | 0.040955531 |
| RNU6-110P | DNAJB14 | 0.25 | 0.040955531 |
| RNU6-110P | ZNF708 | 0.25 | 0.040955531 |
| RNU6-110P | UBAP1L | 0.251 | 0.040172276 |
| RNU6-110P | SLC4A5 | 0.251 | 0.040172276 |
| RNU6-110P | HPN | 0.251 | 0.040172276 |
| RNU6-110P | PSG4 | 0.251 | 0.040172276 |
| RNU6-110P | HSD11B2 | 0.251 | 0.040172276 |
| RNU6-110P | ZNF433 | 0.251 | 0.040172276 |
| RNU6-110P | CEP295NL | 0.251 | 0.040172276 |
| RNU6-110P | ZRSR1 | 0.252 | 0.039401763 |
| RNU6-110P | TRIM34 | 0.252 | 0.039401763 |
| RNU6-110P | ARPP21 | 0.253 | 0.038643833 |
| RNU6-110P | MDM4 | 0.253 | 0.038643833 |
| RNU6-110P | PCDHGB3 | 0.253 | 0.038643833 |
| RNU6-110P | ZNF154 | 0.253 | 0.038643833 |
| RNU6-110P | ZNF570 | 0.253 | 0.038643833 |
| RNU6-110P | MOCS3 | 0.254 | 0.037898328 |
| RNU6-110P | MT-ND5 | 0.254 | 0.037898328 |
| RNU6-110P | ZNF674 | 0.254 | 0.037898328 |
| RNU6-110P | SERPINB9 | 0.254 | 0.037898328 |
| RNU6-110P | UBE2G1 | 0.254 | 0.037898328 |
| RNU6-110P | CDKN2AIPNL | 0.254 | 0.037898328 |
| RNU6-110P | ZNF141 | 0.255 | 0.037165092 |
| RNU6-110P | ZNF66 | 0.255 | 0.037165092 |
| RNU6-110P | KCTD16 | 0.255 | 0.037165092 |
| RNU6-110P | WDR47 | 0.255 | 0.037165092 |
| RNU6-110P | DAGLB | 0.255 | 0.037165092 |
| RNU6-110P | PCDHGA6 | 0.255 | 0.037165092 |
| RNU6-110P | FBXO28 | 0.255 | 0.037165092 |
| RNU6-110P | BTBD18 | 0.255 | 0.037165092 |
| RNU6-110P | MORN5 | 0.255 | 0.037165092 |
| RNU6-110P | ZNF888 | 0.256 | 0.036443968 |
| RNU6-110P | PELI2 | 0.256 | 0.036443968 |
| RNU6-110P | FSIP2 | 0.256 | 0.036443968 |
| RNU6-110P | APCDD1L | 0.256 | 0.036443968 |
| RNU6-110P | AGO3 | 0.256 | 0.036443968 |
| RNU6-110P | FAM228A | 0.256 | 0.036443968 |
| RNU6-110P | UBFD1 | 0.257 | 0.035734802 |
| RNU6-110P | ZNF383 | 0.257 | 0.035734802 |
| RNU6-110P | MDFIC | 0.257 | 0.035734802 |
| RNU6-110P | ZNF43 | 0.257 | 0.035734802 |
| RNU6-110P | ZHX2 | 0.257 | 0.035734802 |
| RNU6-110P | SVOPL | 0.257 | 0.035734802 |
| RNU6-110P | ZNF587 | 0.257 | 0.035734802 |
| RNU6-110P | DBT | 0.258 | 0.03503744 |
| RNU6-110P | C11orf54 | 0.258 | 0.03503744 |
| RNU6-110P | TRAT1 | 0.258 | 0.03503744 |
| RNU6-110P | HYAL3 | 0.258 | 0.03503744 |
| RNU6-110P | TRIM13 | 0.258 | 0.03503744 |
| RNU6-110P | CSRP2 | 0.258 | 0.03503744 |
| RNU6-110P | KLHL28 | 0.258 | 0.03503744 |
| RNU6-110P | AC008575.2 | 0.258 | 0.03503744 |
| RNU6-110P | GATAD2B | 0.258 | 0.03503744 |
| RNU6-110P | RNU6-761P | 0.258 | 0.03503744 |
| RNU6-110P | TMEM260 | 0.259 | 0.03435173 |
| RNU6-110P | HIST1H1A | 0.259 | 0.03435173 |
| RNU6-110P | PPP3CB | 0.259 | 0.03435173 |
| RNU6-110P | AC018630.6 | 0.259 | 0.03435173 |
| RNU6-110P | HIGD1B | 0.259 | 0.03435173 |
| RNU6-110P | C11orf80 | 0.259 | 0.03435173 |
| RNU6-110P | YIPF4 | 0.26 | 0.033677519 |
| RNU6-110P | ETFBKMT | 0.26 | 0.033677519 |
| RNU6-110P | WIPF3 | 0.26 | 0.033677519 |
| RNU6-110P | TMEM268 | 0.26 | 0.033677519 |
| RNU6-110P | DUSP15 | 0.26 | 0.033677519 |
| RNU6-110P | ZNF486 | 0.261 | 0.033014657 |
| RNU6-110P | CNOT6L | 0.261 | 0.033014657 |
| RNU6-110P | KLHDC7A | 0.262 | 0.032362996 |
| RNU6-110P | CEP104 | 0.262 | 0.032362996 |
| RNU6-110P | RNF219 | 0.262 | 0.032362996 |
| RNU6-110P | ERGIC1 | 0.262 | 0.032362996 |
| RNU6-110P | SLC7A14 | 0.262 | 0.032362996 |
| RNU6-110P | ZFHX2 | 0.262 | 0.032362996 |
| RNU6-110P | SLC4A9 | 0.262 | 0.032362996 |
| RNU6-110P | IL17RD | 0.262 | 0.032362996 |
| RNU6-110P | SECISBP2L | 0.262 | 0.032362996 |
| RNU6-110P | C6orf132 | 0.262 | 0.032362996 |
| RNU6-110P | SDHC | 0.262 | 0.032362996 |
| RNU6-110P | ZNF543 | 0.263 | 0.031722386 |
| RNU6-110P | TMEM220 | 0.263 | 0.031722386 |
| RNU6-110P | FRMD7 | 0.263 | 0.031722386 |
| RNU6-110P | TBC1D31 | 0.263 | 0.031722386 |
| RNU6-110P | RAD51D | 0.263 | 0.031722386 |
| RNU6-110P | TEN1 | 0.264 | 0.03109268 |
| RNU6-110P | FAM200A | 0.264 | 0.03109268 |
| RNU6-110P | MED21 | 0.264 | 0.03109268 |
| RNU6-110P | LSM14B | 0.264 | 0.03109268 |
| RNU6-110P | CD99 | 0.264 | 0.03109268 |
| RNU6-110P | RHBDL2 | 0.264 | 0.03109268 |
| RNU6-110P | SLC45A3 | 0.264 | 0.03109268 |
| RNU6-110P | PIGP | 0.264 | 0.03109268 |
| RNU6-110P | ARL13A | 0.264 | 0.03109268 |
| RNU6-110P | ZC3H6 | 0.264 | 0.03109268 |
| RNU6-110P | C9orf85 | 0.264 | 0.03109268 |
| RNU6-110P | PAPD4 | 0.264 | 0.03109268 |
| RNU6-110P | TBXA2R | 0.264 | 0.03109268 |
| RNU6-110P | ZNF41 | 0.265 | 0.030473733 |
| RNU6-110P | MYH11 | 0.265 | 0.030473733 |
| RNU6-110P | FAM177A1 | 0.265 | 0.030473733 |
| RNU6-110P | C1orf189 | 0.265 | 0.030473733 |
| RNU6-110P | SLC9B1 | 0.265 | 0.030473733 |
| RNU6-110P | REST | 0.265 | 0.030473733 |
| RNU6-110P | ZNF621 | 0.265 | 0.030473733 |
| RNU6-110P | HLA-DPA1 | 0.265 | 0.030473733 |
| RNU6-110P | C3orf49 | 0.266 | 0.029865399 |
| RNU6-110P | SNAPC3 | 0.266 | 0.029865399 |
| RNU6-110P | ROBO1 | 0.266 | 0.029865399 |
| RNU6-110P | SLC35F5 | 0.266 | 0.029865399 |
| RNU6-110P | PALM3 | 0.266 | 0.029865399 |
| RNU6-110P | PLA2G2D | 0.266 | 0.029865399 |
| RNU6-110P | INA | 0.266 | 0.029865399 |
| RNU6-110P | FAM175A | 0.266 | 0.029865399 |
| RNU6-110P | AC131097.2 | 0.266 | 0.029865399 |
| RNU6-110P | CBR4 | 0.266 | 0.029865399 |
| RNU6-110P | SMN2 | 0.266 | 0.029865399 |
| RNU6-110P | SEC14L6 | 0.266 | 0.029865399 |
| RNU6-110P | MAP3K19 | 0.267 | 0.029267535 |
| RNU6-110P | FBXO40 | 0.267 | 0.029267535 |
| RNU6-110P | AP001931.1 | 0.267 | 0.029267535 |
| RNU6-110P | FCHSD2 | 0.267 | 0.029267535 |
| RNU6-110P | RFPL1 | 0.267 | 0.029267535 |
| RNU6-110P | PTX4 | 0.268 | 0.028679997 |
| RNU6-110P | SYCP3 | 0.268 | 0.028679997 |
| RNU6-110P | RSBN1L | 0.268 | 0.028679997 |
| RNU6-110P | RCAN3 | 0.268 | 0.028679997 |
| RNU6-110P | KCNIP3 | 0.268 | 0.028679997 |
| RNU6-110P | A1BG | 0.268 | 0.028679997 |
| RNU6-110P | MTRNR2L3 | 0.268 | 0.028679997 |
| RNU6-110P | MBTD1 | 0.268 | 0.028679997 |
| RNU6-110P | PRB4 | 0.268 | 0.028679997 |
| RNU6-110P | RASAL1 | 0.268 | 0.028679997 |
| RNU6-110P | ANGPTL7 | 0.269 | 0.028102645 |
| RNU6-110P | RAB22A | 0.269 | 0.028102645 |
| RNU6-110P | GLRA1 | 0.269 | 0.028102645 |
| RNU6-110P | CNOT4 | 0.269 | 0.028102645 |
| RNU6-110P | ACOT13 | 0.269 | 0.028102645 |
| RNU6-110P | CCDC125 | 0.269 | 0.028102645 |
| RNU6-110P | PFN4 | 0.269 | 0.028102645 |
| RNU6-110P | RUFY2 | 0.269 | 0.028102645 |
| RNU6-110P | PRB3 | 0.269 | 0.028102645 |
| RNU6-110P | FBXL20 | 0.27 | 0.027535338 |
| RNU6-110P | ZNF727 | 0.27 | 0.027535338 |
| RNU6-110P | NCAN | 0.27 | 0.027535338 |
| RNU6-110P | SERF1B | 0.27 | 0.027535338 |
| RNU6-110P | RUNX1T1 | 0.27 | 0.027535338 |
| RNU6-110P | FBXO47 | 0.27 | 0.027535338 |
| RNU6-110P | ERVFRD-1 | 0.27 | 0.027535338 |
| RNU6-110P | C16orf90 | 0.27 | 0.027535338 |
| RNU6-110P | C7orf49 | 0.27 | 0.027535338 |
| RNU6-110P | CCNG2 | 0.27 | 0.027535338 |
| RNU6-110P | ST3GAL1 | 0.27 | 0.027535338 |
| RNU6-110P | MT-ATP8 | 0.271 | 0.026977938 |
| RNU6-110P | WNT9B | 0.271 | 0.026977938 |
| RNU6-110P | SNX29 | 0.271 | 0.026977938 |
| RNU6-110P | KMT5B | 0.271 | 0.026977938 |
| RNU6-110P | ZNF736 | 0.271 | 0.026977938 |
| RNU6-110P | AC017083.4 | 0.271 | 0.026977938 |
| RNU6-110P | CD200 | 0.271 | 0.026977938 |
| RNU6-110P | SHANK1 | 0.272 | 0.026430305 |
| RNU6-110P | SHC1 | 0.272 | 0.026430305 |
| RNU6-110P | CNST | 0.272 | 0.026430305 |
| RNU6-110P | BTBD8 | 0.273 | 0.025892303 |
| RNU6-110P | TRNP1 | 0.273 | 0.025892303 |
| RNU6-110P | CYP2A7 | 0.273 | 0.025892303 |
| RNU6-110P | ZNF490 | 0.273 | 0.025892303 |
| RNU6-110P | AL139353.1 | 0.274 | 0.025363797 |
| RNU6-110P | ZNF345 | 0.274 | 0.025363797 |
| RNU6-110P | C2orf66 | 0.274 | 0.025363797 |
| RNU6-110P | WIPF1 | 0.274 | 0.025363797 |
| RNU6-110P | AKAP2 | 0.274 | 0.025363797 |
| RNU6-110P | CUL4B | 0.274 | 0.025363797 |
| RNU6-110P | C5orf24 | 0.275 | 0.024844652 |
| RNU6-110P | FZD5 | 0.276 | 0.024334735 |
| RNU6-110P | PARP11 | 0.276 | 0.024334735 |
| RNU6-110P | KLHDC1 | 0.276 | 0.024334735 |
| RNU6-110P | PRH1 | 0.276 | 0.024334735 |
| RNU6-110P | S100PBP | 0.276 | 0.024334735 |
| RNU6-110P | MLYCD | 0.276 | 0.024334735 |
| RNU6-110P | MB21D1 | 0.276 | 0.024334735 |
| RNU6-110P | CAV1 | 0.277 | 0.023833914 |
| RNU6-110P | ZNF257 | 0.277 | 0.023833914 |
| RNU6-110P | AIRE | 0.277 | 0.023833914 |
| RNU6-110P | ZNF585A | 0.277 | 0.023833914 |
| RNU6-110P | AC006978.2 | 0.277 | 0.023833914 |
| RNU6-110P | PMEPA1 | 0.278 | 0.023342058 |
| RNU6-110P | SETDB2 | 0.278 | 0.023342058 |
| RNU6-110P | UNC5D | 0.278 | 0.023342058 |
| RNU6-110P | ZNF610 | 0.278 | 0.023342058 |
| RNU6-110P | DNAAF4 | 0.279 | 0.022859037 |
| RNU6-110P | WTH3DI | 0.279 | 0.022859037 |
| RNU6-110P | TNFAIP8L1 | 0.279 | 0.022859037 |
| RNU6-110P | MCCD1 | 0.279 | 0.022859037 |
| RNU6-110P | TPPP3 | 0.279 | 0.022859037 |
| RNU6-110P | SCML2 | 0.28 | 0.022384723 |
| RNU6-110P | ANKUB1 | 0.28 | 0.022384723 |
| RNU6-110P | R3HDM2 | 0.28 | 0.022384723 |
| RNU6-110P | MED28 | 0.28 | 0.022384723 |
| RNU6-110P | ARL11 | 0.28 | 0.022384723 |
| RNU6-110P | SLC24A1 | 0.281 | 0.02191899 |
| RNU6-110P | SOWAHD | 0.281 | 0.02191899 |
| RNU6-110P | LCORL | 0.281 | 0.02191899 |
| RNU6-110P | TMEM43 | 0.282 | 0.02146171 |
| RNU6-110P | TMEM44 | 0.282 | 0.02146171 |
| RNU6-110P | LRRC29 | 0.282 | 0.02146171 |
| RNU6-110P | NF2 | 0.282 | 0.02146171 |
| RNU6-110P | RNU6-1272P | 0.282 | 0.02146171 |
| RNU6-110P | UPK3BL1 | 0.283 | 0.021012759 |
| RNU6-110P | ALS2CR12 | 0.283 | 0.021012759 |
| RNU6-110P | CD79A | 0.283 | 0.021012759 |
| RNU6-110P | KIF26A | 0.283 | 0.021012759 |
| RNU6-110P | ZNF264 | 0.283 | 0.021012759 |
| RNU6-110P | EEF2K | 0.284 | 0.020572014 |
| RNU6-110P | PRAG1 | 0.284 | 0.020572014 |
| RNU6-110P | ZNF436 | 0.284 | 0.020572014 |
| RNU6-110P | TMPRSS11A | 0.285 | 0.020139351 |
| RNU6-110P | TRAF3IP2 | 0.285 | 0.020139351 |
| RNU6-110P | FP565260.6 | 0.285 | 0.020139351 |
| RNU6-110P | FAM69B | 0.286 | 0.019714651 |
| RNU6-110P | TINAGL1 | 0.286 | 0.019714651 |
| RNU6-110P | PLEKHA3 | 0.286 | 0.019714651 |
| RNU6-110P | TRIM4 | 0.286 | 0.019714651 |
| RNU6-110P | NUDT13 | 0.286 | 0.019714651 |
| RNU6-110P | TEFM | 0.286 | 0.019714651 |
| RNU6-110P | PMS2 | 0.286 | 0.019714651 |
| RNU6-110P | ZNF701 | 0.287 | 0.019297794 |
| RNU6-110P | PAPOLG | 0.287 | 0.019297794 |
| RNU6-110P | PUS10 | 0.287 | 0.019297794 |
| RNU6-110P | SMIM18 | 0.287 | 0.019297794 |
| RNU6-110P | ZNF597 | 0.288 | 0.018888659 |
| RNU6-110P | RBAK | 0.288 | 0.018888659 |
| RNU6-110P | ZNF774 | 0.288 | 0.018888659 |
| RNU6-110P | ZACN | 0.288 | 0.018888659 |
| RNU6-110P | TMEM239 | 0.289 | 0.018487131 |
| RNU6-110P | PPP1R10 | 0.289 | 0.018487131 |
| RNU6-110P | PURB | 0.289 | 0.018487131 |
| RNU6-110P | PCDHGA8 | 0.29 | 0.018093093 |
| RNU6-110P | CPA1 | 0.29 | 0.018093093 |
| RNU6-110P | AGBL3 | 0.29 | 0.018093093 |
| RNU6-110P | NT5C2 | 0.29 | 0.018093093 |
| RNU6-110P | PRDM14 | 0.291 | 0.017706429 |
| RNU6-110P | FBXL2 | 0.291 | 0.017706429 |
| RNU6-110P | APOBEC3F | 0.292 | 0.017327027 |
| RNU6-110P | ZNF790 | 0.292 | 0.017327027 |
| RNU6-110P | DNMT3A | 0.292 | 0.017327027 |
| RNU6-110P | PCDHGA10 | 0.292 | 0.017327027 |
| RNU6-110P | USP10 | 0.293 | 0.016954773 |
| RNU6-110P | CSNK2A3 | 0.293 | 0.016954773 |
| RNU6-110P | EVPL | 0.293 | 0.016954773 |
| RNU6-110P | TAS2R10 | 0.293 | 0.016954773 |
| RNU6-110P | TM4SF19-TCTEX1D2 | 0.293 | 0.016954773 |
| RNU6-110P | FSD2 | 0.293 | 0.016954773 |
| RNU6-110P | AQP11 | 0.294 | 0.016589556 |
| RNU6-110P | TMEM170A | 0.294 | 0.016589556 |
| RNU6-110P | RC3H1 | 0.294 | 0.016589556 |
| RNU6-110P | AC093155.3 | 0.294 | 0.016589556 |
| RNU6-110P | ZNF737 | 0.294 | 0.016589556 |
| RNU6-110P | PMM2 | 0.294 | 0.016589556 |
| RNU6-110P | TMEM179 | 0.295 | 0.016231267 |
| RNU6-110P | RIPPLY3 | 0.295 | 0.016231267 |
| RNU6-110P | AL591806.3 | 0.295 | 0.016231267 |
| RNU6-110P | STPG4 | 0.295 | 0.016231267 |
| RNU6-110P | AC073082.1 | 0.295 | 0.016231267 |
| RNU6-110P | SLC16A4 | 0.295 | 0.016231267 |
| RNU6-110P | CREB1 | 0.296 | 0.015879795 |
| RNU6-110P | NPHP3-ACAD11 | 0.296 | 0.015879795 |
| RNU6-110P | CEP41 | 0.296 | 0.015879795 |
| RNU6-110P | CHRNB2 | 0.297 | 0.015535034 |
| RNU6-110P | CA4 | 0.297 | 0.015535034 |
| RNU6-110P | ZNF100 | 0.297 | 0.015535034 |
| RNU6-110P | GHRH | 0.297 | 0.015535034 |
| RNU6-110P | ZBTB25 | 0.297 | 0.015535034 |
| RNU6-110P | DDR1 | 0.297 | 0.015535034 |
| RNU6-110P | HRG | 0.298 | 0.015196876 |
| RNU6-110P | GNRHR | 0.298 | 0.015196876 |
| RNU6-110P | TMED6 | 0.298 | 0.015196876 |
| RNU6-110P | H1FOO | 0.298 | 0.015196876 |
| RNU6-110P | BTBD7 | 0.298 | 0.015196876 |
| RNU6-110P | MFSD8 | 0.299 | 0.014865217 |
| RNU6-110P | TMEM19 | 0.299 | 0.014865217 |
| RNU6-110P | SPDYE5 | 0.299 | 0.014865217 |
| RNU6-110P | HOMEZ | 0.299 | 0.014865217 |
| RNU6-110P | TERF2 | 0.299 | 0.014865217 |
| RNU6-110P | ATAD3C | 0.3 | 0.014539953 |
| RNU6-110P | 6-Sep | 0.3 | 0.014539953 |
| RNU6-110P | ATCAY | 0.3 | 0.014539953 |
| RNU6-110P | CDH4 | 0.3 | 0.014539953 |
| RNU6-110P | BCL6B | 0.301 | 0.01422098 |
| RNU6-110P | IL5RA | 0.301 | 0.01422098 |
| RNU6-110P | ZKSCAN3 | 0.302 | 0.013908197 |
| RNU6-110P | ZNF26 | 0.302 | 0.013908197 |
| RNU6-110P | PXMP4 | 0.302 | 0.013908197 |
| RNU6-110P | RNF115 | 0.302 | 0.013908197 |
| RNU6-110P | ZNF253 | 0.302 | 0.013908197 |
| RNU6-110P | GPA33 | 0.303 | 0.013601503 |
| RNU6-110P | ZNF681 | 0.303 | 0.013601503 |
| RNU6-110P | HLA-DOA | 0.304 | 0.013300799 |
| RNU6-110P | ZNF124 | 0.304 | 0.013300799 |
| RNU6-110P | ZMYM5 | 0.304 | 0.013300799 |
| RNU6-110P | PRSS50 | 0.305 | 0.013005988 |
| RNU6-110P | BET1 | 0.305 | 0.013005988 |
| RNU6-110P | ZNF573 | 0.305 | 0.013005988 |
| RNU6-110P | S100G | 0.305 | 0.013005988 |
| RNU6-110P | KRIT1 | 0.305 | 0.013005988 |
| RNU6-110P | CCDC188 | 0.305 | 0.013005988 |
| RNU6-110P | ZNF70 | 0.306 | 0.01271697 |
| RNU6-110P | RCOR2 | 0.306 | 0.01271697 |
| RNU6-110P | PCDHGA5 | 0.307 | 0.012433652 |
| RNU6-110P | NFAT5 | 0.308 | 0.012155939 |
| RNU6-110P | PDC | 0.308 | 0.012155939 |
| RNU6-110P | ZNF91 | 0.308 | 0.012155939 |
| RNU6-110P | AL139260.3 | 0.309 | 0.011883736 |
| RNU6-110P | KIN | 0.309 | 0.011883736 |
| RNU6-110P | AGXT | 0.31 | 0.011616952 |
| RNU6-110P | KCNG4 | 0.31 | 0.011616952 |
| RNU6-110P | HYAL2 | 0.31 | 0.011616952 |
| RNU6-110P | ATXN2 | 0.311 | 0.011355495 |
| RNU6-110P | ZNF235 | 0.313 | 0.010848205 |
| RNU6-110P | HERC4 | 0.313 | 0.010848205 |
| RNU6-110P | CARF | 0.313 | 0.010848205 |
| RNU6-110P | C8orf44-SGK3 | 0.314 | 0.010602196 |
| RNU6-110P | AC114490.2 | 0.314 | 0.010602196 |
| RNU6-110P | HS3ST3B1 | 0.314 | 0.010602196 |
| RNU6-110P | GPC1 | 0.314 | 0.010602196 |
| RNU6-110P | ALDH6A1 | 0.314 | 0.010602196 |
| RNU6-110P | PTGR2 | 0.314 | 0.010602196 |
| RNU6-110P | HFE2 | 0.314 | 0.010602196 |
| RNU6-110P | MFAP4 | 0.314 | 0.010602196 |
| RNU6-110P | ZNF624 | 0.315 | 0.01036116 |
| RNU6-110P | ZNRF2 | 0.315 | 0.01036116 |
| RNU6-110P | MEGF11 | 0.316 | 0.010125013 |
| RNU6-110P | PRH2 | 0.316 | 0.010125013 |
| RNU6-110P | ARC | 0.317 | 0.009893671 |
| RNU6-110P | DEPTOR | 0.317 | 0.009893671 |
| RNU6-110P | PGPEP1L | 0.317 | 0.009893671 |
| RNU6-110P | KIAA1841 | 0.317 | 0.009893671 |
| RNU6-110P | ADAD2 | 0.317 | 0.009893671 |
| RNU6-110P | SSUH2 | 0.317 | 0.009893671 |
| RNU6-110P | ZNF680 | 0.318 | 0.009667049 |
| RNU6-110P | RARRES1 | 0.318 | 0.009667049 |
| RNU6-110P | XIAP | 0.318 | 0.009667049 |
| RNU6-110P | BSCL2 | 0.318 | 0.009667049 |
| RNU6-110P | PVRIG | 0.319 | 0.009445067 |
| RNU6-110P | SEZ6 | 0.319 | 0.009445067 |
| RNU6-110P | ZNF808 | 0.319 | 0.009445067 |
| RNU6-110P | SLCO5A1 | 0.32 | 0.009227642 |
| RNU6-110P | CCSAP | 0.32 | 0.009227642 |
| RNU6-110P | CD160 | 0.32 | 0.009227642 |
| RNU6-110P | TAS2R13 | 0.32 | 0.009227642 |
| RNU6-110P | FER1L5 | 0.32 | 0.009227642 |
| RNU6-110P | OR2AE1 | 0.321 | 0.009014696 |
| RNU6-110P | ITM2B | 0.321 | 0.009014696 |
| RNU6-110P | GREB1L | 0.322 | 0.008806148 |
| RNU6-110P | RPEL1 | 0.322 | 0.008806148 |
| RNU6-110P | LEPROTL1 | 0.322 | 0.008806148 |
| RNU6-110P | KIAA0391 | 0.323 | 0.008601922 |
| RNU6-110P | ZC3H10 | 0.323 | 0.008601922 |
| RNU6-110P | MTRNR2L7 | 0.323 | 0.008601922 |
| RNU6-110P | ZNF611 | 0.323 | 0.008601922 |
| RNU6-110P | ZNF233 | 0.323 | 0.008601922 |
| RNU6-110P | CPNE6 | 0.323 | 0.008601922 |
| RNU6-110P | AC068946.1 | 0.324 | 0.00840194 |
| RNU6-110P | ZFP30 | 0.324 | 0.00840194 |
| RNU6-110P | MECP2 | 0.324 | 0.00840194 |
| RNU6-110P | PCDHGB2 | 0.324 | 0.00840194 |
| RNU6-110P | RELL1 | 0.324 | 0.00840194 |
| RNU6-110P | ZNF619 | 0.324 | 0.00840194 |
| RNU6-110P | ORAI2 | 0.325 | 0.008206126 |
| RNU6-110P | MAPKAPK5 | 0.325 | 0.008206126 |
| RNU6-110P | ZNF254 | 0.325 | 0.008206126 |
| RNU6-110P | MTRNR2L8 | 0.325 | 0.008206126 |
| RNU6-110P | L1CAM | 0.325 | 0.008206126 |
| RNU6-110P | NEURL1B | 0.326 | 0.008014407 |
| RNU6-110P | GLIS3 | 0.326 | 0.008014407 |
| RNU6-110P | LRIG2 | 0.327 | 0.007826708 |
| RNU6-110P | TPO | 0.327 | 0.007826708 |
| RNU6-110P | MEST | 0.327 | 0.007826708 |
| RNU6-110P | CCND1 | 0.327 | 0.007826708 |
| RNU6-110P | PLEKHA2 | 0.328 | 0.007642957 |
| RNU6-110P | THBS3 | 0.329 | 0.007463082 |
| RNU6-110P | CIITA | 0.329 | 0.007463082 |
| RNU6-110P | ACYP2 | 0.329 | 0.007463082 |
| RNU6-110P | NAPEPLD | 0.33 | 0.007287013 |
| RNU6-110P | TMIGD2 | 0.33 | 0.007287013 |
| RNU6-110P | ALS2CL | 0.33 | 0.007287013 |
| RNU6-110P | PGAM5 | 0.33 | 0.007287013 |
| RNU6-110P | IRX6 | 0.331 | 0.007114681 |
| RNU6-110P | ABHD18 | 0.332 | 0.006946016 |
| RNU6-110P | MTRNR2L10 | 0.332 | 0.006946016 |
| RNU6-110P | ZFP14 | 0.332 | 0.006946016 |
| RNU6-110P | AC012309.1 | 0.332 | 0.006946016 |
| RNU6-110P | STYX | 0.333 | 0.006780952 |
| RNU6-110P | COG8 | 0.334 | 0.006619421 |
| RNU6-110P | TADA2A | 0.334 | 0.006619421 |
| RNU6-110P | AKAP5 | 0.334 | 0.006619421 |
| RNU6-110P | ZBTB42 | 0.334 | 0.006619421 |
| RNU6-110P | GOSR1 | 0.335 | 0.006461359 |
| RNU6-110P | CCDC89 | 0.336 | 0.0063067 |
| RNU6-110P | FAM46B | 0.337 | 0.006155381 |
| RNU6-110P | SCO1 | 0.337 | 0.006155381 |
| RNU6-110P | PLTP | 0.337 | 0.006155381 |
| RNU6-110P | PTPRH | 0.338 | 0.006007339 |
| RNU6-110P | TMCC1 | 0.339 | 0.005862513 |
| RNU6-110P | ROBO4 | 0.339 | 0.005862513 |
| RNU6-110P | ZFP69B | 0.339 | 0.005862513 |
| RNU6-110P | NCAM1 | 0.34 | 0.005720842 |
| RNU6-110P | FAM180B | 0.341 | 0.005582266 |
| RNU6-110P | SPATA32 | 0.341 | 0.005582266 |
| RNU6-110P | ZG16B | 0.342 | 0.005446725 |
| RNU6-110P | WNT4 | 0.342 | 0.005446725 |
| RNU6-110P | LINC00675 | 0.342 | 0.005446725 |
| RNU6-110P | GPALPP1 | 0.343 | 0.005314163 |
| RNU6-110P | DUSP14 | 0.343 | 0.005314163 |
| RNU6-110P | CCM2 | 0.344 | 0.005184521 |
| RNU6-110P | KLHL36 | 0.345 | 0.005057744 |
| RNU6-110P | KCNH8 | 0.347 | 0.004812563 |
| RNU6-110P | MYO1H | 0.347 | 0.004812563 |
| RNU6-110P | ZNF724 | 0.348 | 0.004694051 |
| RNU6-110P | PTGIR | 0.348 | 0.004694051 |
| RNU6-110P | CSRNP2 | 0.348 | 0.004694051 |
| RNU6-110P | FAM81A | 0.348 | 0.004694051 |
| RNU6-110P | MAVS | 0.349 | 0.004578187 |
| RNU6-110P | ACADSB | 0.351 | 0.004354197 |
| RNU6-110P | RGS20 | 0.351 | 0.004354197 |
| RNU6-110P | LINC00854 | 0.351 | 0.004354197 |
| RNU6-110P | ZNF852 | 0.352 | 0.004245969 |
| RNU6-110P | CNTN2 | 0.353 | 0.004140187 |
| RNU6-110P | GRK5 | 0.353 | 0.004140187 |
| RNU6-110P | C9orf57 | 0.354 | 0.004036802 |
| RNU6-110P | LIX1L | 0.355 | 0.003935765 |
| RNU6-110P | JUP | 0.356 | 0.003837031 |
| RNU6-110P | MMP28 | 0.356 | 0.003837031 |
| RNU6-110P | RGS9 | 0.356 | 0.003837031 |
| RNU6-110P | MTRNR2L4 | 0.357 | 0.003740552 |
| RNU6-110P | FAM120C | 0.357 | 0.003740552 |
| RNU6-110P | CRYGD | 0.358 | 0.003646283 |
| RNU6-110P | SPIN2A | 0.359 | 0.00355418 |
| RNU6-110P | ZNF720 | 0.359 | 0.00355418 |
| RNU6-110P | ZNF675 | 0.361 | 0.003376294 |
| RNU6-110P | DCAF7 | 0.362 | 0.003290425 |
| RNU6-110P | EDEM1 | 0.362 | 0.003290425 |
| RNU6-110P | SSC5D | 0.362 | 0.003290425 |
| RNU6-110P | USH2A | 0.363 | 0.003206551 |
| RNU6-110P | MFAP2 | 0.366 | 0.002966484 |
| RNU6-110P | HIF3A | 0.366 | 0.002966484 |
| RNU6-110P | ATF7 | 0.368 | 0.002815674 |
| RNU6-110P | KLRG2 | 0.368 | 0.002815674 |
| RNU6-110P | RNASE8 | 0.369 | 0.002742925 |
| RNU6-110P | MTRNR2L13 | 0.37 | 0.002671897 |
| RNU6-110P | C16orf95 | 0.372 | 0.002534859 |
| RNU6-110P | TMEM140 | 0.372 | 0.002534859 |
| RNU6-110P | AL033529.1 | 0.374 | 0.002404278 |
| RNU6-110P | GPR12 | 0.374 | 0.002404278 |
| RNU6-110P | BCORL1 | 0.376 | 0.002279881 |
| RNU6-110P | KRT19 | 0.376 | 0.002279881 |
| RNU6-110P | CRLF3 | 0.377 | 0.00221992 |
| RNU6-110P | MAK | 0.378 | 0.002161407 |
| RNU6-110P | SPDYE3 | 0.378 | 0.002161407 |
| RNU6-110P | DHTKD1 | 0.378 | 0.002161407 |
| RNU6-110P | ZSCAN32 | 0.38 | 0.002048601 |
| RNU6-110P | SAP30L | 0.38 | 0.002048601 |
| RNU6-110P | WFDC1 | 0.38 | 0.002048601 |
| RNU6-110P | TNFRSF21 | 0.381 | 0.001994248 |
| RNU6-110P | MTUS1 | 0.381 | 0.001994248 |
| RNU6-110P | MTRNR2L12 | 0.383 | 0.001889491 |
| RNU6-110P | WDR20 | 0.385 | 0.00178981 |
| RNU6-110P | TTLL9 | 0.387 | 0.001694984 |
| RNU6-110P | GRIP2 | 0.387 | 0.001694984 |
| RNU6-110P | MTRNR2L6 | 0.388 | 0.001649324 |
| RNU6-110P | TMEM51 | 0.39 | 0.001561382 |
| RNU6-110P | OR3A2 | 0.391 | 0.001519049 |
| RNU6-110P | TMEM106A | 0.392 | 0.001477776 |
| RNU6-110P | LRCH3 | 0.394 | 0.001398313 |
| RNU6-110P | RD3 | 0.395 | 0.001360076 |
| RNU6-110P | GJA9 | 0.395 | 0.001360076 |
| RNU6-110P | MPL | 0.396 | 0.001322806 |
| RNU6-110P | INMT | 0.396 | 0.001322806 |
| RNU6-110P | PCP4L1 | 0.396 | 0.001322806 |
| RNU6-110P | KDM4B | 0.397 | 0.001286481 |
| RNU6-110P | FNDC5 | 0.397 | 0.001286481 |
| RNU6-110P | CD34 | 0.398 | 0.001251078 |
| RNU6-110P | TPPP | 0.399 | 0.001216576 |
| RNU6-110P | SMAD6 | 0.401 | 0.001150195 |
| RNU6-110P | ZMYM6 | 0.402 | 0.001118275 |
| RNU6-110P | ERICH3 | 0.403 | 0.001087176 |
| RNU6-110P | MAN1A1 | 0.404 | 0.001056878 |
| RNU6-110P | SPDYE1 | 0.404 | 0.001056878 |
| RNU6-110P | MTRNR2L11 | 0.405 | 0.001027363 |
| RNU6-110P | PIRT | 0.408 | 0.000943333 |
| RNU6-110P | PALM | 0.409 | 0.000916769 |
| RNU6-110P | FBLN2 | 0.409 | 0.000916769 |
| RNU6-110P | ZNF506 | 0.412 | 0.00084118 |
| RNU6-110P | SPACA9 | 0.413 | 0.000817296 |
| RNU6-110P | TAMM41 | 0.414 | 0.000794043 |
| RNU6-110P | DOK7 | 0.414 | 0.000794043 |
| RNU6-110P | SOGA1 | 0.416 | 0.000749369 |
| RNU6-110P | ITGB4 | 0.416 | 0.000749369 |
| RNU6-110P | HIGD1C | 0.417 | 0.000727917 |
| RNU6-110P | PLEKHG6 | 0.418 | 0.000707037 |
| RNU6-110P | PDLIM1 | 0.418 | 0.000707037 |
| RNU6-110P | MTRNR2L5 | 0.418 | 0.000707037 |
| RNU6-110P | SRMS | 0.418 | 0.000707037 |
| RNU6-110P | RASA4 | 0.419 | 0.000686715 |
| RNU6-110P | MREG | 0.42 | 0.000666937 |
| RNU6-110P | TTC9 | 0.421 | 0.00064769 |
| RNU6-110P | OR3A1 | 0.424 | 0.000593003 |
| RNU6-110P | PHYHIP | 0.429 | 0.000511309 |
| RNU6-110P | REM1 | 0.432 | 0.000467462 |
| RNU6-110P | TCP11L1 | 0.433 | 0.000453643 |
| RNU6-110P | TP53TG3D | 0.435 | 0.000427143 |
| RNU6-110P | HOMER2 | 0.436 | 0.000414441 |
| RNU6-110P | C17orf78 | 0.438 | 0.000390089 |
| RNU6-110P | LPAR5 | 0.447 | 0.000296157 |
| RNU6-110P | PPL | 0.448 | 0.000287143 |
| RNU6-110P | ADRA2C | 0.45 | 0.00026988 |
| RNU6-110P | ZBTB8A | 0.452 | 0.000253593 |
| RNU6-110P | AQP6 | 0.454 | 0.000238231 |
| RNU6-110P | FAM166B | 0.454 | 0.000238231 |
| RNU6-110P | C2CD4C | 0.457 | 0.000216818 |
| RNU6-110P | POU4F1 | 0.46 | 0.000197221 |
| RNU6-110P | CAMK2B | 0.461 | 0.000191068 |
| RNU6-110P | GRAPL | 0.463 | 0.000179298 |
| RNU6-110P | CD52 | 0.467 | 0.000157775 |
| RNU6-110P | RNF125 | 0.469 | 0.000147948 |
| RNU6-110P | BAIAP3 | 0.474 | 0.000125843 |
| RNU6-110P | PSORS1C1 | 0.475 | 0.000121814 |
| RNU6-110P | TMIE | 0.475 | 0.000121814 |
| RNU6-110P | TERB2 | 0.477 | 0.000114116 |
| RNU6-110P | ZMYM1 | 0.478 | 0.000110442 |
| RNU6-110P | STK32B | 0.48 | 0.000103425 |
| RNU6-110P | RASA4B | 0.482 | 9.68E-05 |
| RNU6-110P | PAFAH2 | 0.506 | 4.31E-05 |
| RNU6-110P | GPR152 | 0.519 | 2.74E-05 |
| RNU6-110P | PTGDR2 | 0.521 | 2.55E-05 |
| RNU6-110P | SLC5A10 | 0.525 | 2.21E-05 |
| RNU6-110P | SLC2A7 | 0.528 | 1.99E-05 |
| RNU6-110P | TRH | 0.53 | 1.85E-05 |
| RNU6-110P | FBLN5 | 0.54 | 1.29E-05 |
| RNU6-110P | CACNA2D2 | 0.549 | 9.25E-06 |
| RNU6-110P | CLEC2L | 0.559 | 6.37E-06 |
| RNU6-110P | C12orf42 | 0.587 | 2.16E-06 |
| RNU6-110P | PCDH15 | 0.605 | 1.05E-06 |
| RNU6-110P | CTNNA2 | 0.609 | 8.94E-07 |
| RNU6-110P | PADI3 | 0.632 | 3.44E-07 |
| RNU6-110P | RNU6-110P | 1 | 9.07E-16 |
| RNU4-8P | NFYC | -0.422 | 0.001872668 |
| RNU4-8P | TMEM199 | -0.405 | 0.002813222 |
| RNU4-8P | DIAPH1 | -0.401 | 0.003089677 |
| RNU4-8P | ZBTB18 | -0.388 | 0.004167877 |
| RNU4-8P | RNF130 | -0.363 | 0.007245319 |
| RNU4-8P | MAP4K4 | -0.361 | 0.007563285 |
| RNU4-8P | JAK1 | -0.361 | 0.007563285 |
| RNU4-8P | TATDN2 | -0.358 | 0.008063717 |
| RNU4-8P | AGTPBP1 | -0.356 | 0.008413604 |
| RNU4-8P | PPP2R2D | -0.355 | 0.008593589 |
| RNU4-8P | EZR | -0.351 | 0.009348434 |
| RNU4-8P | DCAF5 | -0.349 | 0.009747598 |
| RNU4-8P | TNFRSF14 | -0.338 | 0.01222652 |
| RNU4-8P | TBC1D10A | -0.338 | 0.01222652 |
| RNU4-8P | RIOK3 | -0.337 | 0.01247745 |
| RNU4-8P | PPP4R1 | -0.336 | 0.012732929 |
| RNU4-8P | ENTPD4 | -0.332 | 0.013801737 |
| RNU4-8P | CDKN2D | -0.331 | 0.014081017 |
| RNU4-8P | CYTH1 | -0.32 | 0.01749808 |
| RNU4-8P | C16orf70 | -0.32 | 0.01749808 |
| RNU4-8P | AMFR | -0.319 | 0.017842103 |
| RNU4-8P | COPG1 | -0.318 | 0.018192038 |
| RNU4-8P | IQSEC1 | -0.317 | 0.018547968 |
| RNU4-8P | OTULIN | -0.315 | 0.019278148 |
| RNU4-8P | FYN | -0.312 | 0.020420507 |
| RNU4-8P | GRASP | -0.311 | 0.020814198 |
| RNU4-8P | RAP2A | -0.309 | 0.021621469 |
| RNU4-8P | ADAM9 | -0.309 | 0.021621469 |
| RNU4-8P | STK24 | -0.309 | 0.021621469 |
| RNU4-8P | EIF2AK3 | -0.308 | 0.022035228 |
| RNU4-8P | REEP5 | -0.307 | 0.022455858 |
| RNU4-8P | KIAA0922 | -0.307 | 0.022455858 |
| RNU4-8P | MXRA7 | -0.307 | 0.022455858 |
| RNU4-8P | METRNL | -0.307 | 0.022455858 |
| RNU4-8P | RAPGEF1 | -0.306 | 0.022883451 |
| RNU4-8P | MIA3 | -0.305 | 0.023318099 |
| RNU4-8P | SART3 | -0.305 | 0.023318099 |
| RNU4-8P | RAF1 | -0.304 | 0.023759896 |
| RNU4-8P | MAD1L1 | -0.304 | 0.023759896 |
| RNU4-8P | MCUB | -0.304 | 0.023759896 |
| RNU4-8P | CLTC | -0.303 | 0.024208937 |
| RNU4-8P | SNX1 | -0.303 | 0.024208937 |
| RNU4-8P | WIPI1 | -0.303 | 0.024208937 |
| RNU4-8P | TACC1 | -0.303 | 0.024208937 |
| RNU4-8P | COPA | -0.301 | 0.02512913 |
| RNU4-8P | ARFGAP3 | -0.301 | 0.02512913 |
| RNU4-8P | RAB43 | -0.3 | 0.025600474 |
| RNU4-8P | DYNLT1 | -0.299 | 0.026079448 |
| RNU4-8P | CECR6 | -0.299 | 0.026079448 |
| RNU4-8P | GOLGA5 | -0.298 | 0.026566149 |
| RNU4-8P | ELMO2 | -0.297 | 0.027060675 |
| RNU4-8P | PHLPP2 | -0.297 | 0.027060675 |
| RNU4-8P | SENP5 | -0.296 | 0.027563128 |
| RNU4-8P | ST3GAL6 | -0.295 | 0.028073607 |
| RNU4-8P | VPS39 | -0.294 | 0.028592214 |
| RNU4-8P | SPTAN1 | -0.294 | 0.028592214 |
| RNU4-8P | NLRP1 | -0.293 | 0.029119051 |
| RNU4-8P | GNG2 | -0.293 | 0.029119051 |
| RNU4-8P | TADA2B | -0.293 | 0.029119051 |
| RNU4-8P | SRSF4 | -0.292 | 0.029654221 |
| RNU4-8P | RNF19B | -0.292 | 0.029654221 |
| RNU4-8P | PAPD7 | -0.292 | 0.029654221 |
| RNU4-8P | SEC13 | -0.291 | 0.030197828 |
| RNU4-8P | ILKAP | -0.291 | 0.030197828 |
| RNU4-8P | RASSF5 | -0.291 | 0.030197828 |
| RNU4-8P | EPB41 | -0.29 | 0.030749975 |
| RNU4-8P | TNFAIP3 | -0.29 | 0.030749975 |
| RNU4-8P | MAP3K8 | -0.29 | 0.030749975 |
| RNU4-8P | STIM1 | -0.289 | 0.03131077 |
| RNU4-8P | JAK3 | -0.289 | 0.03131077 |
| RNU4-8P | CORO1C | -0.289 | 0.03131077 |
| RNU4-8P | TMX4 | -0.289 | 0.03131077 |
| RNU4-8P | USP7 | -0.288 | 0.031880316 |
| RNU4-8P | ZBTB2 | -0.288 | 0.031880316 |
| RNU4-8P | C18orf8 | -0.287 | 0.032458721 |
| RNU4-8P | HPS3 | -0.287 | 0.032458721 |
| RNU4-8P | CPEB4 | -0.286 | 0.033046093 |
| RNU4-8P | ADIPOR1 | -0.286 | 0.033046093 |
| RNU4-8P | FAM129B | -0.285 | 0.033642539 |
| RNU4-8P | NDRG3 | -0.285 | 0.033642539 |
| RNU4-8P | CDC42EP4 | -0.283 | 0.034863091 |
| RNU4-8P | BRD7 | -0.283 | 0.034863091 |
| RNU4-8P | JMJD6 | -0.282 | 0.035487417 |
| RNU4-8P | CALCB | -0.282 | 0.035487417 |
| RNU4-8P | SLC26A11 | -0.282 | 0.035487417 |
| RNU4-8P | PPIB | -0.282 | 0.035487417 |
| RNU4-8P | STAT3 | -0.281 | 0.036121256 |
| RNU4-8P | ATP1A1 | -0.281 | 0.036121256 |
| RNU4-8P | OS9 | -0.281 | 0.036121256 |
| RNU4-8P | SIPA1L1 | -0.28 | 0.036764722 |
| RNU4-8P | CRYBG1 | -0.28 | 0.036764722 |
| RNU4-8P | SPATA13 | -0.28 | 0.036764722 |
| RNU4-8P | STX6 | -0.279 | 0.037417926 |
| RNU4-8P | MAPK6 | -0.279 | 0.037417926 |
| RNU4-8P | KIF13A | -0.279 | 0.037417926 |
| RNU4-8P | NAPA | -0.278 | 0.038080981 |
| RNU4-8P | ATP2A2 | -0.278 | 0.038080981 |
| RNU4-8P | IL16 | -0.278 | 0.038080981 |
| RNU4-8P | GAB2 | -0.277 | 0.038754001 |
| RNU4-8P | MXD3 | -0.277 | 0.038754001 |
| RNU4-8P | TRAFD1 | -0.277 | 0.038754001 |
| RNU4-8P | NSFL1C | -0.276 | 0.039437101 |
| RNU4-8P | C18orf25 | -0.275 | 0.040130396 |
| RNU4-8P | SYTL3 | -0.275 | 0.040130396 |
| RNU4-8P | STAB1 | -0.275 | 0.040130396 |
| RNU4-8P | GLRX3 | -0.274 | 0.040834 |
| RNU4-8P | NARF | -0.274 | 0.040834 |
| RNU4-8P | GPAT4 | -0.274 | 0.040834 |
| RNU4-8P | TBC1D20 | -0.273 | 0.041548032 |
| RNU4-8P | TADA3 | -0.273 | 0.041548032 |
| RNU4-8P | CCDC85C | -0.272 | 0.042272608 |
| RNU4-8P | MTMR6 | -0.272 | 0.042272608 |
| RNU4-8P | ABCA1 | -0.272 | 0.042272608 |
| RNU4-8P | NUAK2 | -0.272 | 0.042272608 |
| RNU4-8P | ATG9A | -0.271 | 0.043007844 |
| RNU4-8P | LGALS12 | -0.271 | 0.043007844 |
| RNU4-8P | CD3D | -0.27 | 0.043753861 |
| RNU4-8P | TDRD9 | -0.27 | 0.043753861 |
| RNU4-8P | PHF13 | -0.27 | 0.043753861 |
| RNU4-8P | ANKRD44 | -0.27 | 0.043753861 |
| RNU4-8P | TPD52L2 | -0.27 | 0.043753861 |
| RNU4-8P | ANAPC15 | -0.269 | 0.044510776 |
| RNU4-8P | TIMP1 | -0.269 | 0.044510776 |
| RNU4-8P | AREL1 | -0.269 | 0.044510776 |
| RNU4-8P | HLA-G | -0.269 | 0.044510776 |
| RNU4-8P | 3-Mar | -0.269 | 0.044510776 |
| RNU4-8P | VMP1 | -0.269 | 0.044510776 |
| RNU4-8P | GBF1 | -0.268 | 0.04527871 |
| RNU4-8P | CACFD1 | -0.268 | 0.04527871 |
| RNU4-8P | 8-Mar | -0.268 | 0.04527871 |
| RNU4-8P | KCTD6 | -0.267 | 0.046057781 |
| RNU4-8P | PSMD2 | -0.267 | 0.046057781 |
| RNU4-8P | FFAR1 | -0.267 | 0.046057781 |
| RNU4-8P | SKIL | -0.267 | 0.046057781 |
| RNU4-8P | LYSMD4 | -0.267 | 0.046057781 |
| RNU4-8P | 7-Sep | -0.267 | 0.046057781 |
| RNU4-8P | SMOX | -0.266 | 0.046848111 |
| RNU4-8P | WBP11 | -0.266 | 0.046848111 |
| RNU4-8P | NDEL1 | -0.265 | 0.047649822 |
| RNU4-8P | IL17RA | -0.265 | 0.047649822 |
| RNU4-8P | BRPF1 | -0.264 | 0.048463034 |
| RNU4-8P | LDLRAD4 | -0.264 | 0.048463034 |
| RNU4-8P | MKRN1 | -0.264 | 0.048463034 |
| RNU4-8P | CARS | -0.263 | 0.049287871 |
| RNU4-8P | TFIP11 | -0.263 | 0.049287871 |
| RNU4-8P | FYTTD1 | -0.263 | 0.049287871 |
| RNU4-8P | CWF19L1 | -0.263 | 0.049287871 |
| RNU4-8P | C8orf88 | -0.263 | 0.049287871 |
| RNU4-8P | SEC61A1 | -0.263 | 0.049287871 |
| RNU4-8P | TTC7A | -0.263 | 0.049287871 |
| RNU4-8P | COX11 | 0.283 | 0.049871103 |
| RNU4-8P | ZNF2 | 0.283 | 0.049871103 |
| RNU4-8P | ASB3 | 0.283 | 0.049871103 |
| RNU4-8P | C12orf45 | 0.283 | 0.049871103 |
| RNU4-8P | EEF2KMT | 0.284 | 0.049038072 |
| RNU4-8P | ZNF586 | 0.284 | 0.049038072 |
| RNU4-8P | SIRT5 | 0.284 | 0.049038072 |
| RNU4-8P | HSPB7 | 0.284 | 0.049038072 |
| RNU4-8P | HMX3 | 0.284 | 0.049038072 |
| RNU4-8P | ZP1 | 0.284 | 0.049038072 |
| RNU4-8P | CFAP99 | 0.284 | 0.049038072 |
| RNU4-8P | CFAP206 | 0.284 | 0.049038072 |
| RNU4-8P | RPAP2 | 0.284 | 0.049038072 |
| RNU4-8P | ZNF45 | 0.284 | 0.049038072 |
| RNU4-8P | ZNF131 | 0.285 | 0.048216752 |
| RNU4-8P | ZNF286B | 0.285 | 0.048216752 |
| RNU4-8P | TEDDM1 | 0.285 | 0.048216752 |
| RNU4-8P | GOLPH3L | 0.285 | 0.048216752 |
| RNU4-8P | WDR35 | 0.285 | 0.048216752 |
| RNU4-8P | PPEF2 | 0.285 | 0.048216752 |
| RNU4-8P | SMIM20 | 0.286 | 0.047407018 |
| RNU4-8P | XKRX | 0.286 | 0.047407018 |
| RNU4-8P | ERCC2 | 0.286 | 0.047407018 |
| RNU4-8P | SYNPO2L | 0.286 | 0.047407018 |
| RNU4-8P | ZNF671 | 0.286 | 0.047407018 |
| RNU4-8P | RCC1 | 0.286 | 0.047407018 |
| RNU4-8P | UQCC2 | 0.286 | 0.047407018 |
| RNU4-8P | FAM135B | 0.286 | 0.047407018 |
| RNU4-8P | BTBD18 | 0.286 | 0.047407018 |
| RNU4-8P | TMEM186 | 0.286 | 0.047407018 |
| RNU4-8P | ZNF33B | 0.286 | 0.047407018 |
| RNU4-8P | FSBP | 0.287 | 0.04660875 |
| RNU4-8P | ALKBH2 | 0.287 | 0.04660875 |
| RNU4-8P | HHIPL1 | 0.287 | 0.04660875 |
| RNU4-8P | TBC1D26 | 0.287 | 0.04660875 |
| RNU4-8P | ZNF264 | 0.287 | 0.04660875 |
| RNU4-8P | ATG9B | 0.288 | 0.045821826 |
| RNU4-8P | CFAP53 | 0.288 | 0.045821826 |
| RNU4-8P | CASC1 | 0.288 | 0.045821826 |
| RNU4-8P | NME1 | 0.288 | 0.045821826 |
| RNU4-8P | NDUFV3 | 0.288 | 0.045821826 |
| RNU4-8P | PKDREJ | 0.288 | 0.045821826 |
| RNU4-8P | RBPJL | 0.288 | 0.045821826 |
| RNU4-8P | ZNF345 | 0.288 | 0.045821826 |
| RNU4-8P | CDK18 | 0.288 | 0.045821826 |
| RNU4-8P | PTCD2 | 0.288 | 0.045821826 |
| RNU4-8P | PAQR8 | 0.288 | 0.045821826 |
| RNU4-8P | ZNF529 | 0.288 | 0.045821826 |
| RNU4-8P | ZNF846 | 0.289 | 0.045046123 |
| RNU4-8P | LRRC71 | 0.289 | 0.045046123 |
| RNU4-8P | NEURL2 | 0.289 | 0.045046123 |
| RNU4-8P | DCHS2 | 0.289 | 0.045046123 |
| RNU4-8P | MFSD9 | 0.289 | 0.045046123 |
| RNU4-8P | ECT2L | 0.289 | 0.045046123 |
| RNU4-8P | GID4 | 0.289 | 0.045046123 |
| RNU4-8P | ASPHD1 | 0.289 | 0.045046123 |
| RNU4-8P | PHLDB3 | 0.289 | 0.045046123 |
| RNU4-8P | USH2A | 0.289 | 0.045046123 |
| RNU4-8P | SMC4 | 0.289 | 0.045046123 |
| RNU4-8P | SMTNL2 | 0.289 | 0.045046123 |
| RNU4-8P | OR51I2 | 0.289 | 0.045046123 |
| RNU4-8P | PINLYP | 0.289 | 0.045046123 |
| RNU4-8P | C1orf194 | 0.289 | 0.045046123 |
| RNU4-8P | RBM26 | 0.29 | 0.044281523 |
| RNU4-8P | ZNRD1 | 0.29 | 0.044281523 |
| RNU4-8P | C16orf71 | 0.29 | 0.044281523 |
| RNU4-8P | ZNF791 | 0.29 | 0.044281523 |
| RNU4-8P | TNNI3K | 0.29 | 0.044281523 |
| RNU4-8P | WDR5B | 0.29 | 0.044281523 |
| RNU4-8P | SEMA3F | 0.291 | 0.043527905 |
| RNU4-8P | SLC15A1 | 0.291 | 0.043527905 |
| RNU4-8P | CCDC58 | 0.291 | 0.043527905 |
| RNU4-8P | RRS1 | 0.291 | 0.043527905 |
| RNU4-8P | CCDC125 | 0.291 | 0.043527905 |
| RNU4-8P | HCAR1 | 0.291 | 0.043527905 |
| RNU4-8P | FKBP7 | 0.291 | 0.043527905 |
| RNU4-8P | THAP6 | 0.291 | 0.043527905 |
| RNU4-8P | OR51Q1 | 0.291 | 0.043527905 |
| RNU4-8P | ZNF506 | 0.292 | 0.042785149 |
| RNU4-8P | ZNF665 | 0.292 | 0.042785149 |
| RNU4-8P | HEATR4 | 0.292 | 0.042785149 |
| RNU4-8P | SLC35E3 | 0.292 | 0.042785149 |
| RNU4-8P | GOLGA8O | 0.292 | 0.042785149 |
| RNU4-8P | EXOG | 0.292 | 0.042785149 |
| RNU4-8P | C2orf72 | 0.292 | 0.042785149 |
| RNU4-8P | SFXN4 | 0.293 | 0.042053137 |
| RNU4-8P | NANOG | 0.293 | 0.042053137 |
| RNU4-8P | UPF3B | 0.293 | 0.042053137 |
| RNU4-8P | GPATCH4 | 0.293 | 0.042053137 |
| RNU4-8P | HSF2BP | 0.293 | 0.042053137 |
| RNU4-8P | DNAH6 | 0.293 | 0.042053137 |
| RNU4-8P | HEYL | 0.293 | 0.042053137 |
| RNU4-8P | MTX2 | 0.293 | 0.042053137 |
| RNU4-8P | USP54 | 0.294 | 0.041331752 |
| RNU4-8P | PIGF | 0.294 | 0.041331752 |
| RNU4-8P | ADAM11 | 0.294 | 0.041331752 |
| RNU4-8P | CNFN | 0.294 | 0.041331752 |
| RNU4-8P | HELQ | 0.295 | 0.040620874 |
| RNU4-8P | XYLB | 0.295 | 0.040620874 |
| RNU4-8P | FUZ | 0.295 | 0.040620874 |
| RNU4-8P | ZFP2 | 0.295 | 0.040620874 |
| RNU4-8P | IFT74 | 0.295 | 0.040620874 |
| RNU4-8P | DUSP11 | 0.296 | 0.039920389 |
| RNU4-8P | GSTCD | 0.296 | 0.039920389 |
| RNU4-8P | TREML2 | 0.296 | 0.039920389 |
| RNU4-8P | TCEANC2 | 0.296 | 0.039920389 |
| RNU4-8P | HOXA7 | 0.297 | 0.039230179 |
| RNU4-8P | HSPE1 | 0.297 | 0.039230179 |
| RNU4-8P | AC073896.1 | 0.297 | 0.039230179 |
| RNU4-8P | RPS27A | 0.297 | 0.039230179 |
| RNU4-8P | OPA3 | 0.298 | 0.038550128 |
| RNU4-8P | TDRD5 | 0.298 | 0.038550128 |
| RNU4-8P | GLRA2 | 0.298 | 0.038550128 |
| RNU4-8P | ZNF891 | 0.298 | 0.038550128 |
| RNU4-8P | SPACA5 | 0.298 | 0.038550128 |
| RNU4-8P | KIF9 | 0.298 | 0.038550128 |
| RNU4-8P | ZPBP | 0.298 | 0.038550128 |
| RNU4-8P | ALDOB | 0.298 | 0.038550128 |
| RNU4-8P | ANKRD18B | 0.298 | 0.038550128 |
| RNU4-8P | PLPP7 | 0.299 | 0.037880123 |
| RNU4-8P | UPK3BL1 | 0.299 | 0.037880123 |
| RNU4-8P | CKM | 0.299 | 0.037880123 |
| RNU4-8P | SERHL2 | 0.299 | 0.037880123 |
| RNU4-8P | COA4 | 0.299 | 0.037880123 |
| RNU4-8P | MSX2 | 0.299 | 0.037880123 |
| RNU4-8P | L3MBTL1 | 0.299 | 0.037880123 |
| RNU4-8P | MLH3 | 0.299 | 0.037880123 |
| RNU4-8P | MRM1 | 0.3 | 0.037220049 |
| RNU4-8P | MMP21 | 0.3 | 0.037220049 |
| RNU4-8P | NLRP2 | 0.3 | 0.037220049 |
| RNU4-8P | PDYN | 0.3 | 0.037220049 |
| RNU4-8P | ZBED6CL | 0.3 | 0.037220049 |
| RNU4-8P | EEF1E1 | 0.3 | 0.037220049 |
| RNU4-8P | HOMEZ | 0.3 | 0.037220049 |
| RNU4-8P | MYRIP | 0.3 | 0.037220049 |
| RNU4-8P | GOLGA8Q | 0.301 | 0.036569791 |
| RNU4-8P | ZNF780B | 0.301 | 0.036569791 |
| RNU4-8P | TTBK1 | 0.301 | 0.036569791 |
| RNU4-8P | ZC2HC1C | 0.301 | 0.036569791 |
| RNU4-8P | MIS18A | 0.301 | 0.036569791 |
| RNU4-8P | TWNK | 0.301 | 0.036569791 |
| RNU4-8P | TRPV6 | 0.301 | 0.036569791 |
| RNU4-8P | SYT3 | 0.302 | 0.035929238 |
| RNU4-8P | MPZL2 | 0.302 | 0.035929238 |
| RNU4-8P | NMNAT3 | 0.302 | 0.035929238 |
| RNU4-8P | DPH6 | 0.302 | 0.035929238 |
| RNU4-8P | GSPT2 | 0.302 | 0.035929238 |
| RNU4-8P | RPS12 | 0.302 | 0.035929238 |
| RNU4-8P | CCNB1IP1 | 0.303 | 0.035298277 |
| RNU4-8P | LCLAT1 | 0.303 | 0.035298277 |
| RNU4-8P | CDNF | 0.304 | 0.034676797 |
| RNU4-8P | CYP4Z1 | 0.304 | 0.034676797 |
| RNU4-8P | LRRC34 | 0.304 | 0.034676797 |
| RNU4-8P | NUPL2 | 0.304 | 0.034676797 |
| RNU4-8P | DNAJC30 | 0.304 | 0.034676797 |
| RNU4-8P | ACOT2 | 0.304 | 0.034676797 |
| RNU4-8P | GPR75 | 0.304 | 0.034676797 |
| RNU4-8P | KLHL20 | 0.304 | 0.034676797 |
| RNU4-8P | MT-ND2 | 0.305 | 0.034064687 |
| RNU4-8P | BBS10 | 0.305 | 0.034064687 |
| RNU4-8P | TRIM16L | 0.305 | 0.034064687 |
| RNU4-8P | APOLD1 | 0.305 | 0.034064687 |
| RNU4-8P | LBHD1 | 0.305 | 0.034064687 |
| RNU4-8P | FMO4 | 0.305 | 0.034064687 |
| RNU4-8P | NIPAL2 | 0.306 | 0.033461836 |
| RNU4-8P | MT-ATP8 | 0.306 | 0.033461836 |
| RNU4-8P | PCDH9 | 0.306 | 0.033461836 |
| RNU4-8P | SERPINI2 | 0.306 | 0.033461836 |
| RNU4-8P | SEZ6 | 0.306 | 0.033461836 |
| RNU4-8P | RSPH3 | 0.306 | 0.033461836 |
| RNU4-8P | TMEM217 | 0.306 | 0.033461836 |
| RNU4-8P | AHI1 | 0.306 | 0.033461836 |
| RNU4-8P | ZNF830 | 0.306 | 0.033461836 |
| RNU4-8P | BBS5 | 0.307 | 0.032868136 |
| RNU4-8P | WDPCP | 0.307 | 0.032868136 |
| RNU4-8P | WDR78 | 0.307 | 0.032868136 |
| RNU4-8P | DNAH12 | 0.307 | 0.032868136 |
| RNU4-8P | SCLY | 0.307 | 0.032868136 |
| RNU4-8P | PLA2G3 | 0.308 | 0.032283478 |
| RNU4-8P | PEX7 | 0.308 | 0.032283478 |
| RNU4-8P | HNF4G | 0.308 | 0.032283478 |
| RNU4-8P | LDLRAD2 | 0.308 | 0.032283478 |
| RNU4-8P | TOB2 | 0.308 | 0.032283478 |
| RNU4-8P | CELA2B | 0.308 | 0.032283478 |
| RNU4-8P | UBQLN3 | 0.308 | 0.032283478 |
| RNU4-8P | ZNF233 | 0.308 | 0.032283478 |
| RNU4-8P | GOLGA6L7P | 0.308 | 0.032283478 |
| RNU4-8P | RNASE10 | 0.308 | 0.032283478 |
| RNU4-8P | WDR49 | 0.309 | 0.031707754 |
| RNU4-8P | RNLS | 0.309 | 0.031707754 |
| RNU4-8P | PMFBP1 | 0.309 | 0.031707754 |
| RNU4-8P | DKKL1 | 0.309 | 0.031707754 |
| RNU4-8P | SEMA3A | 0.309 | 0.031707754 |
| RNU4-8P | HIST3H3 | 0.309 | 0.031707754 |
| RNU4-8P | WISP2 | 0.309 | 0.031707754 |
| RNU4-8P | MANSC4 | 0.309 | 0.031707754 |
| RNU4-8P | TGDS | 0.309 | 0.031707754 |
| RNU4-8P | ZNF714 | 0.309 | 0.031707754 |
| RNU4-8P | SOX7 | 0.309 | 0.031707754 |
| RNU4-8P | TLCD1 | 0.31 | 0.031140856 |
| RNU4-8P | ZNF140 | 0.31 | 0.031140856 |
| RNU4-8P | RPP14 | 0.31 | 0.031140856 |
| RNU4-8P | CHST5 | 0.31 | 0.031140856 |
| RNU4-8P | ZBTB3 | 0.31 | 0.031140856 |
| RNU4-8P | ZNF19 | 0.31 | 0.031140856 |
| RNU4-8P | GOLGA8H | 0.31 | 0.031140856 |
| RNU4-8P | EFCAB6 | 0.31 | 0.031140856 |
| RNU4-8P | CABYR | 0.31 | 0.031140856 |
| RNU4-8P | CFAP52 | 0.31 | 0.031140856 |
| RNU4-8P | MRPL34 | 0.31 | 0.031140856 |
| RNU4-8P | CATSPER2 | 0.31 | 0.031140856 |
| RNU4-8P | GUCY2F | 0.31 | 0.031140856 |
| RNU4-8P | RNF220 | 0.311 | 0.030582678 |
| RNU4-8P | SSC4D | 0.311 | 0.030582678 |
| RNU4-8P | FAM186B | 0.311 | 0.030582678 |
| RNU4-8P | PMS1 | 0.311 | 0.030582678 |
| RNU4-8P | ZNF843 | 0.311 | 0.030582678 |
| RNU4-8P | HFE2 | 0.311 | 0.030582678 |
| RNU4-8P | TSPYL6 | 0.311 | 0.030582678 |
| RNU4-8P | CATSPERG | 0.312 | 0.030033115 |
| RNU4-8P | ARRDC5 | 0.312 | 0.030033115 |
| RNU4-8P | HOOK2 | 0.312 | 0.030033115 |
| RNU4-8P | IFT80 | 0.312 | 0.030033115 |
| RNU4-8P | CYP4A22 | 0.312 | 0.030033115 |
| RNU4-8P | LPIN3 | 0.312 | 0.030033115 |
| RNU4-8P | SOCS2 | 0.312 | 0.030033115 |
| RNU4-8P | IFT46 | 0.313 | 0.029492062 |
| RNU4-8P | AC011498.5 | 0.313 | 0.029492062 |
| RNU4-8P | LRRC69 | 0.313 | 0.029492062 |
| RNU4-8P | AKR7A3 | 0.313 | 0.029492062 |
| RNU4-8P | NFIC | 0.313 | 0.029492062 |
| RNU4-8P | MACC1 | 0.313 | 0.029492062 |
| RNU4-8P | MAP3K13 | 0.313 | 0.029492062 |
| RNU4-8P | SLC7A9 | 0.314 | 0.028959414 |
| RNU4-8P | ZNF568 | 0.314 | 0.028959414 |
| RNU4-8P | AC003002.1 | 0.314 | 0.028959414 |
| RNU4-8P | IMMP2L | 0.314 | 0.028959414 |
| RNU4-8P | TMEM267 | 0.315 | 0.028435068 |
| RNU4-8P | CLYBL | 0.315 | 0.028435068 |
| RNU4-8P | ZFP14 | 0.315 | 0.028435068 |
| RNU4-8P | DFFA | 0.315 | 0.028435068 |
| RNU4-8P | RPL36 | 0.316 | 0.027918921 |
| RNU4-8P | LRRIQ4 | 0.317 | 0.027410871 |
| RNU4-8P | UNC80 | 0.317 | 0.027410871 |
| RNU4-8P | SLC22A7 | 0.318 | 0.026910818 |
| RNU4-8P | LCMT2 | 0.318 | 0.026910818 |
| RNU4-8P | ACSM5 | 0.318 | 0.026910818 |
| RNU4-8P | ZNF189 | 0.318 | 0.026910818 |
| RNU4-8P | SLC5A12 | 0.318 | 0.026910818 |
| RNU4-8P | KLHL31 | 0.318 | 0.026910818 |
| RNU4-8P | MGST2 | 0.319 | 0.02641866 |
| RNU4-8P | GPR179 | 0.319 | 0.02641866 |
| RNU4-8P | KCNC4 | 0.319 | 0.02641866 |
| RNU4-8P | GLS2 | 0.32 | 0.025934299 |
| RNU4-8P | AC114490.2 | 0.32 | 0.025934299 |
| RNU4-8P | METTL12 | 0.32 | 0.025934299 |
| RNU4-8P | RBM43 | 0.32 | 0.025934299 |
| RNU4-8P | THTPA | 0.32 | 0.025934299 |
| RNU4-8P | LDB3 | 0.32 | 0.025934299 |
[truncated: 1,224,167 more chars]
